# Disulfide re-bridging reagents for single-payload antibody-drug conjugates†

**DOI:** 10.1039/d3cc02980h

**Published:** 2023-07-18

**Authors:** Thomas A. King, Stephen J. Walsh, Mia Kapun, Thomas Wharton, Sona Krajcovicova, Melanie S. Glossop, David R. Spring

**Affiliations:** a Yusuf Hamied Department of Chemistry, University of Cambridge Cambridge CB2 1EW UK spring@ch.cam.ac.uk; b Apollo Therapeutics Ltd 50-60 Station Road Cambridge CB1 2JH UK; c Department of Organic Chemistry, Faculty of Science, Palacky University Tr. 17. Listopadu 12 Olomouc 77900 Czech Republic

## Abstract

Numerous antibody-drug conjugate (ADC) linker technologies exist for the synthesis of ADCs with drug-to-antibody ratios (DARs) being an even integer (typically 2, 4 or 8). However, ADCs with odd-integer DARs are significantly harder to synthesise. Here, we report the synthesis of ADCs loaded with a single warhead, using TetraDVP linkers which simultaneously re-bridge all four interchain disulfides of an IgG1 antibody.

## Abstract 2

A tetra-reactive disulfide re-bridging reagent, TetraDVP, enables the efficient synthesis of antibody-drug conjugates (ADCs) bearing a single payload.

## Graphical abstract

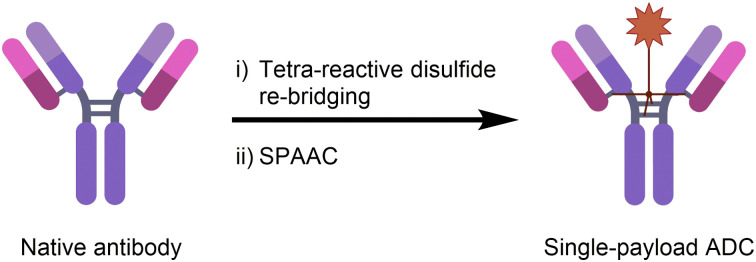

The synthesis of ADCs is a rapidly advancing field. There are currently 12 ADCs approved for medical use,^1,2^ and many more are progressing through clinical trials. Drug loading plays a pivotal role in determining the pharmacology of an ADC.^3^ However, due to the inherent 2-fold symmetry of antibodies, current site-selective conjugation methods are mostly limited to the synthesis of ADCs with even-integer DAR.^4^ While there have been rare reports of the generation of homogeneous ADCs with odd DARs,^5–11^ the applied methods tend to rely on engineered antibodies or bespoke payloads, or have poor efficiency, which greatly limits their widespread applicability. Methods which expand the repertoire of available drug loading, such as for the generic synthesis of ADCs with odd-integer DAR, would enable the synthesis of more finely tuned therapeutics.

Disulfide re-bridging, *via* reduction of the interchain disulfides of an IgG1 antibody followed by reaction with a bis-reactive reagent, has proven successful for the synthesis of ADCs bearing two and four payloads.^4,12^ However, there are very few methods for the accurate synthesis of ADCs bearing a single payload.^5–11^

Our group recently reported the first generation of all-in-one disulfide bridging crosslinking reagents, termed TetraDVPs, which use four divinylpyrimidine (DVP) moieties to simultaneously re-bridge all four interchain disulfides of an IgG1 antibody (Fig. 1).^13^ This resulted in antibody-linker conjugates (ALCs) in which all four peptide chains of the antibody were connected through a single chemical linker. ALCs synthesised using this technology were converted into antibody-fluorophore conjugates in near quantitative conversion *via* copper-catalysed azide-alkyne cycloaddition (CuAAC). However, when using the hydrophobic payload monomethyl auristatin E (MMAE), conversion to the corresponding ADC was less efficient, affording a DAR of 0.5, rather than the desired DAR of 1.^12^

**Fig. 1:**
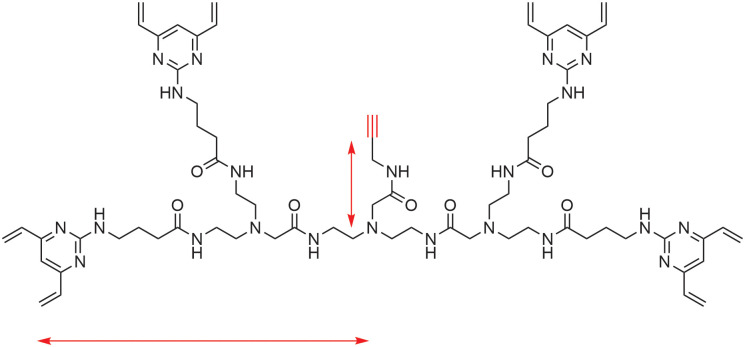
TetraDVP linkers reported by Dannheim *et al.* enabled the simultaneous re-bridging of all four interchain disulfides of an IgG1 antibody. However, subsequent CuAAC reaction failed to produce the desired ADCs in high conversion, postulated to be due to steric hindrance of the pendant alkyne.

Here, we report significant improvements in the conversion of new TetraDVP-based ALCs to ADCs.

It was postulated that steric hindrance around the central alkyne functionality was a significant factor contributing to the limited conversion observed with the initial TetraDVPs. To alleviate this, a second generation of TetraDVPs was devised in which the length of the linker to the click reactive functionality was increased to varying degrees. It was hoped that this would make the central functional handle more accessible to the payload reagent after bioconjugation. Additionally, exchanging the alkyne click handle for an azide on the TetraDVP scaffold was hoped to enable the use of strain-promoted azide-alkyne cycloaddition (SPAAC) reactions, such as with dibenzylcyclcooctyne (DBCO), and thus enable further optimisation of reaction time and remove the requirement for a copper catalyst.

To investigate the effect of changing either the linker backbone or the side chain, a second generation of TetraDVP scaffolds was devised (Fig. 2). The study began with the synthesis of eight alternative TetraDVPs (1a–d and 2a–d), containing varying numbers of PEG spacer units in the linker backbone (highlighted in blue, Fig. 2), and either short (1) or long (2) azide-containing side chains (highlighted in orange, Fig. 2; see ESI† for schemes and procedures). The design of these two series of TetraDVPs has been envisaged to minimise the steric hindrance during the re-bridging step whilst maximising the potential of a successful attachment of the warhead *via* SPAAC.

**Fig. 2:**
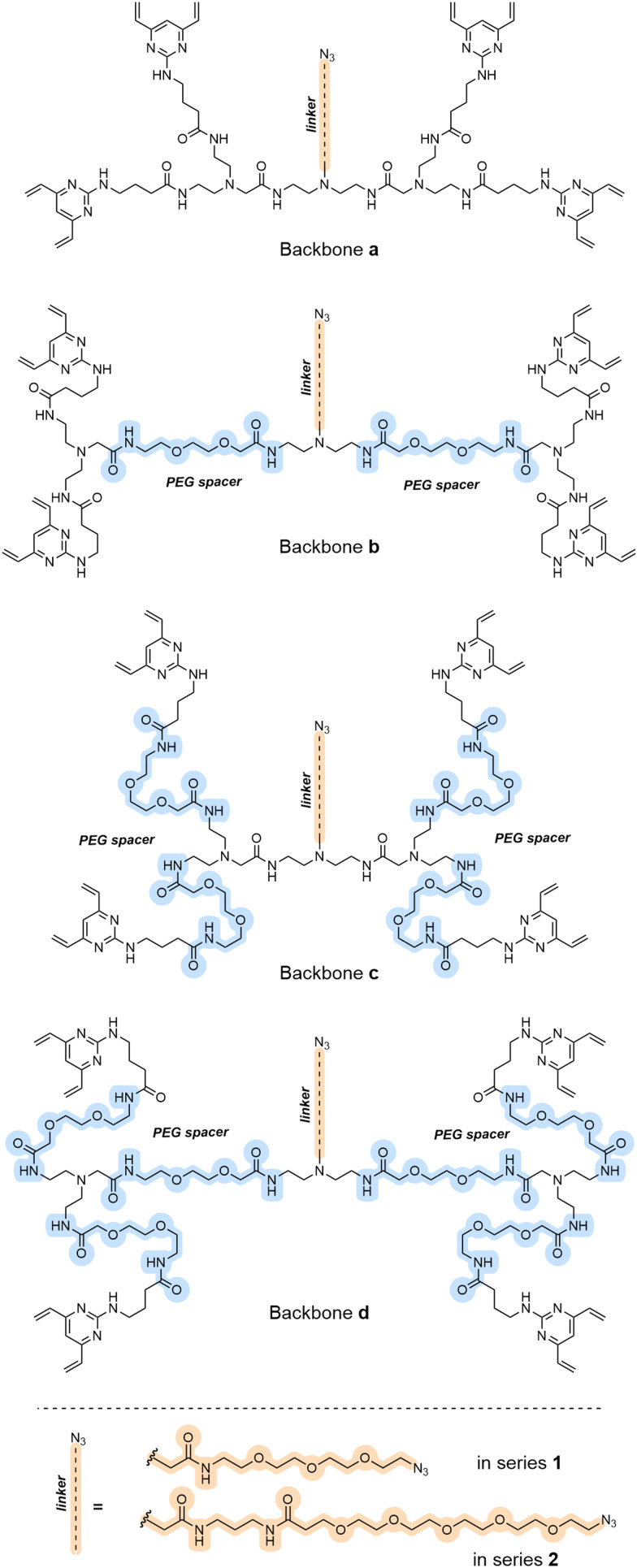
TetraDVP linkers synthesised in this work. PEG spacers were included in the linker backbones (a–d) and azide-containing side chains of different lengths were employed (series 1 and 2, respectively).

To assess the re-bridging efficiency of the eight linkers, bioconjugation was attempted with the anti-HER2 antibody trastuzumab (Scheme 1). The interchain disulfide bonds were reduced by incubation at 37 °C with ten equivalents of tris(2-carboxyethyl)phosphine hydrochloride (TCEP·HCl) for one hour. Initial optimisation using linker 1a identified efficient bioconjugation using ten equivalents of linker (see ESI†). The efficiency of bioconjugation of linkers 1a–d and 2a–d to trastuzumab was then compared. Pleasingly, incubating reduced trastuzumab at 37 °C with ten equivalents of linker for four hours provided near quantitative conversion to the desired ALCs in all cases, as shown by sodium dodecyl sulfate-polyacrylamide gel electrophoresis (SDS–PAGE). No species were observed in the gel which would correspond to free heavy or light chain, indicating successful re-bridging (Scheme 1). This indicates a remarkable steric flexibility of the TetraDVP backbone linkers, even without the presence of a PEG spacer unit. Double addition of the linker was not observed during analysis of any of the bioconjugation reactions. Moreover, the purity of the linkers did not significantly influence the overall outcome of the re-bridging.

**Scheme 1:**
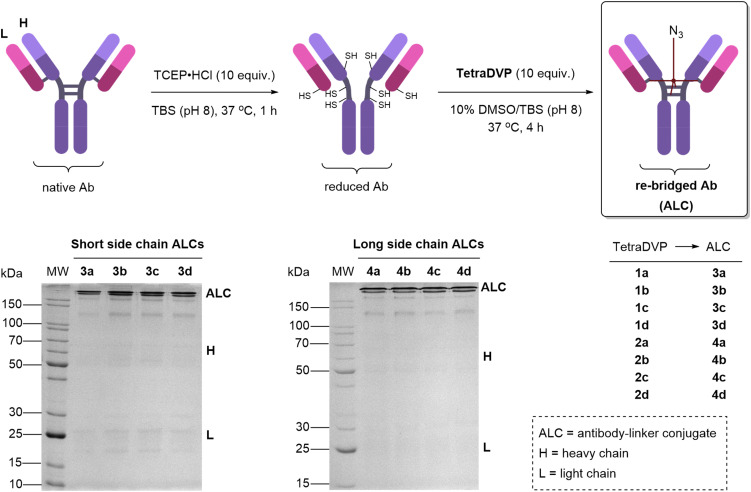
SDS-PAGE analysis, under reducing conditions, of trastuzumab (Ab) re-bridged with the two series of linkers. The major species is the fully re-bridged antibody for all linkers tested. Proteins were stained with Coomassie Brilliant Blue before imaging.

Given the reported disparity of click efficiency when using payloads of different hydrophilicity,^13^ it was decided to directly examine the click reactivity of the new ALCs 3a–d and 4a–d using a hydrophobic payload. Payload 5 (containing a protease-cleavable valine-citrulline motif, the tubulin inhibitor monomethyl auristatin E (MMAE), and a DBCO unit) was therefore synthesised (see ESI†).

The eight ALCs 3a–d and 4a–d were subjected to SPAAC reactions with the DBCO-containing payload 5 (Table 1). Initial incubation of the two components at 37 °C for four hours generated the desired ADCs 6a–d and 7a–d in improved conversion compared to the alkyne-containing linker previously reported.^13^ Pleasingly, extending the reaction time to 24 hours enabled improved conversion to the desired ADCs, enabling the synthesis of ADCs with DARs of 0.8–0.9, as determined by hydrophobic interaction chromatography (HIC). As was apparent from the obtained results, the length difference between the shorter (1) and a longer (2) azide-containing linker played a role in the outcome of the SPAAC reaction; in almost all cases the longer azide-containing TetraDVPs led to higher DARs (Table 1). Although using longer reaction times or greater equivalents of 5 did not lead to increased conversion (see ESI†), these results still represent a great improvement from the previously reported first generation of TetraDVP linkers and would serve as a valuable starting point for the further optimisation of the concept.

**Table 1:** Optimisation of the SPAAC reaction between ALCs and DBCO-containing payload 5

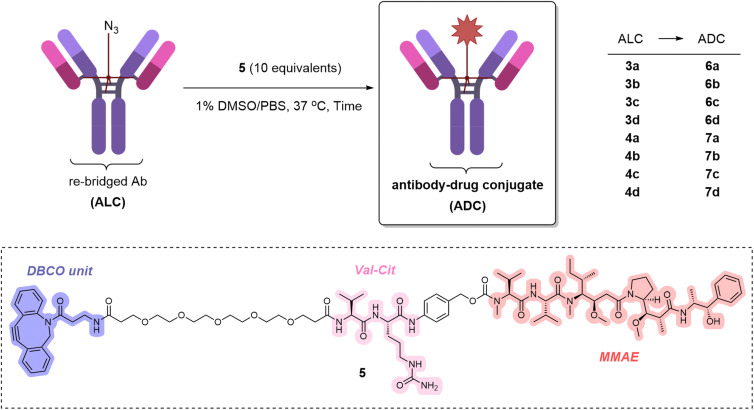									
Entry	Time/h	DAR							
		6a	6b	6c	6d	7a	7b	7c	7d
1	4	0.74	0.69	0.81	0.83	0.75	0.74	0.71	0.75
2	24	0.82	0.76	0.84	0.89	0.87	0.90	0.84	0.87

To further demonstrate the utility of the TetraDVP methodology, ALCs 4a–d were reacted with a DBCO reagent containing a pyrrolobenzodiazepine-dimer payload (8, Fig. 3). After optimisation, the reaction provided ADCs 9a–d with DARs of up to 0.94, as determined by HIC (see ESI†).

**Fig. 3:**
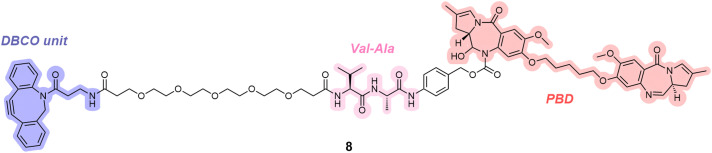
DBCO reagent 8 used for the addition of a pyrrolobenzodiazepine (PBD) dimer payload to ALCs 4a–d.

In summary, eight new TetraDVPs have been synthesised and assessed on their ability to re-bridge the native antibody trastuzumab. All linkers enabled nearly complete conversion to the corresponding ALCs using a small number of equivalents compared to the antibody. The new linkers also enabled significantly improved conversion of the ALCs to ADCs containing a single payload (MMAE and PBD-dimer). These results indicate a significant development in the TetraDVP technology, which enables the efficient synthesis of ADCs bearing a single payload, without the need for antibody engineering.

T. A. K. was involved in conceptualisation, investigation, visualisation, and writing. S. J. W. was involved in conceptualisation and investigation. M. K. and T. W. were involved in investigation and writing. S. K. was involved in writing. M. G. was involved in investigation and supervision. D. R. S. was involved in conceptualisation and supervision.

T. A. K. thanks BBSRC Doctoral Training Partnership (BB/S50757X/1) for funding. M. K. and T. W. thank EPSRC Centre for Doctoral Training – SynTech (EP/S024220/01) for studentships. S. K. is grateful to the Experientia Foundation (www.experientia.cz), Czech Science Foundation (GA CR 22-07138O) and Cambridge Isaac Newton Trust (Grant Ref. No: 22.39(l)) for their financial support. The Spring group acknowledges support from UKRI grants. For the purpose of Open Access, the authors have applied a CC BY public copyright licence to any Author Accepted Manuscript (AAM) version arising.

## Conflicts of interest

S. J. W. and D. R. S. are inventors on a patent application covering the compounds reported in this communication.

## Supplementary Material

CC-059-D3CC02980H-s001
