# Supplementary material for: Disulfide re-bridging reagents for single-payload antibody-drug conjugates
Source: Chem Commun (Camb). 2023 Jul 18;59(65):9868–71. doi: 10.1039/d3cc02980h (PMC10414422; doi:10.1039/d3cc02980h)
Supplement: CC-059-D3CC02980H-s001 [file CC-059-D3CC02980H-s001.pdf]

Electronic Supplementary Material (ESI) for ChemComm.
This journal is © The Royal Society of Chemistry 2023

Supporting information

Disulfide re-bridging reagents for single-payload antibody-drug conjugates

Thomas A. King,[a] Stephen J. Walsh,[a] Mia Kapun,[a] Thomas Wharton,[a] Sona Krajcovicova,[a,c] Melanie S. Glossop,[b] and David R. Spring[*a]

[a] Yusuf Hamied Department of Chemistry, University of Cambridge, Cambridge, CB2 1EW, UK
[b] Apollo Therapeutics Ltd, 50–60 Station Road, Cambridge, CB1 2JH
[c] Department of Chemistry, Faculty of Science, Palacky University, Tr. 17. Listopadu 12, Olomouc, 77900, Czech Republic

E-mail: spring@ch.cam.ac.uk

Contents

1 General **3**

2 Synthesis of divinylpyrimidine (DVP) reagent S1 **6**

3 Synthesis of TetraDVP linkers **9**

4 Synthesis of DBCO reagent 5 **33**

5 Trastuzumab bioconjugation **38**

5.1 General procedure for trastuzumab bioconjugation 38

5.2 Optimisation of bioconjugation using TetraDVP **1a** 39

5.3 Additional SDS-PAGE analysis including controls 41

6 ADC synthesis **42**

6.1 General procedure for click chemistry 42

7 Synthesis of PEG_4-azide reagents **45**

8 NMR spectra **49**

9 HPLC data **78**

10 Protein mass spectra **88**

11 Size-exclusion chromatography (SEC) and hydrophobic interaction chromatography (HIC) **108**

1 General

Small molecule chemistry

Proton nuclear magnetic resonance (^1H NMR) spectra were recorded at ambient temperature on a Bruker DPX-400 (400 MHz), Bruker Advance 400 QNP (400 MHz), and Bruker Avance 500 Cryo Ultrashield (500 MHz). ^1H NMR chemical shifts (δ_H) are reported in parts per million (ppm), to the nearest 0.01 ppm and are referenced to the residual non-deuterated solvent peak ($CDCl_3$: 7.26, CD_3OD: 3.31, DMSO-d_6: 2.50). Coupling constants (J) are reported in Hertz (Hz) to the nearest 0.1 Hz. Data are reported as follows: chemical shift, multiplicity (app = apparent; s = singlet; d = doublet; t = triplet; q = quartet; qn = quintet; sx = sextet; m = multiplet; or as a combination of these), coupling constant(s), integration and assignment.

Carbon NMR (^{13}C NMR) were recorded at ambient temperature on a Bruker DPX-400 (400 MHz), Bruker Advance 400 QNP (400 MHz), and Bruker Avance 500 Cryo Ultrashield (500 MHz). Chemical shifts (δ_C) are quoted in ppm, to the nearest 0.1 ppm, and are referenced to the residual non-deuterated solvent peak ($CDCl_3$: 77.16, CD_3OD: 49.00, DMSO-d_6: 39.52).

^1H NMR and ^{13}C NMR spectra assignments were supported by DEPT–135, COSY (2D, ^1H–^1H correlations), HSQC (2D, one bond ^1H–^{13}C correlations), HMBC (2D, multiple bond ^1H–^{13}C correlations) and NOESY (2D, ^1H–^1H correlations) where appropriate. The numbering of molecules used for ^{13}C and ^1H NMR assignments does not conform to IUPAC standards.

High resolution mass spectrometry (HRMS) measurements were recorded with a Micromass Q-TOF mass spectrometer or a Waters LCT Premier Time of Flight mass spectrometer using Electrospray ionisation (ESI) techniques. Mass values are reported within the 5 ppm error limit.

Thin layer chromatography (TLC) was performed using pre-coated Merck glass backed silica gel 60 F254 plates and visualised by quenching of UV fluorescence (λ_{max} = 254 nm) or by staining with potassium permanganate, ninhydrin, ammonium molybdate or bromocresol green. Retention factors (R_f) are quoted to 0.01. Flash column chromatography was carried out using Merck 9385 Kieselgel 60 SiO_2 (230–400 mesh) under a positive pressure of air unless otherwise stated. Automated flash column chromatography was carried out on a Combiflash Rf200 automated chromatography system with Redisep® reverse-phase C_{18}-silica flash columns (20–40 μm).

Liquid chromatography mass spectroscopy (LCMS) was carried out using a Waters ACQUITY HClass UPLC with an ESCi Multi-Mode Ionisation Waters SQ Detector 2 spectrometer using MassLynx 4.1 software; EI refers to the electrospray ionisation technique; LC system: solvent A: 2 mM NH_4OAc in H_2O/MeCN (95:5); solvent B: MeCN; solvent C: 2% formic acid$_{(aq)}$; column: ACQUITY UPLC CSH C18 (2.1 mm × 50 mm, 1.7 μm, 130 Å) at 40 °C; gradient: 5–95 % B with constant 5% C, over 1 minute at flow rate of 0.6 mL/minute; detector: PDA eλ Detector 220 - 800 nm, interval 1.2 nm.

Analytical high-performance LC (HPLC) was carried out using an Agilent 1260 Infinity system with a

reversed-phase Supelcosil™ ABZ+PLUS column (150 mm × 4.6 mm, 3 μm) eluting with a linear gradient system (solvent A: 0.05% (v/v) TFA in H_2O, solvent B: 0.05% (v/v) TFA in MeCN) over 15 minutes, unless otherwise stated, at a flow rate of 1 mL/min. HPLC was monitored by UV absorbance at 220 and 254 nm. Preparative HPLC was carried out using an Agilent 1260 Infinity with a reversed-phase Supelcosil™ ABZ+PLUS column (250 mm × 21.2 mm, 5 μm) eluting with a linear gradient system (solvent A: 0.1% (v/v) TFA in H_2O, solvent B: 0.05% (v/v) TFA in MeCN) over 20 minutes at a flow rate of 20 mL/min. HPLC was monitored by UV absorbance at 220 and 254 nm.

Ethyl acetate, methanol, dichloromethane, acetonitrile and toluene were distilled from calcium hydride. Diethyl ether was distilled from a mixture of lithium aluminium hydride and calcium hydride. Tetrahydrofuran was dried using sodium wire and distilled from a mixture of lithium aluminium hydride and calcium hydride with triphenylmethane as an indicator. Petroleum ether (henceforth referred to as 'pet. ether') was distilled before use and refers to the fraction between 40–60 °C. All other solvents and reagents were obtained from commercial suppliers and used without further purification. Reactions were carried out under a stream of nitrogen using oven-dried glassware unless otherwise stated. Room temperature (rt) refers to ambient temperature. Temperatures of -10 °C were maintained using an acetone-ice bath. All temperatures below -10 °C were maintained using an acetone-cardice bath. Temperatures of 0 °C were maintained using an ice-water bath.

Protein chemistry

Non-reducing Tris-Glycine SDS-PAGE with 8% or 12% acrylamide with 4% stacking gel was performed as standard. Broad range molecular weight marker (10–200 kDa, New England BioLabs) was run in all gels. Samples were prepared with reducing or non-reducing loading dye (10 μL, reducing dye contained β-mercaptoethanol) and heated to 90 °C for 5 min before loading. Gels were run at constant voltage (200 V) for 45–60 min in Laemmli running buffer (LRB). All gels were stained with Coomassie dye and imaged on a Syngene gel imaging system. Gels containing fluorescently labelled samples were imaged for in-gel fluorescence prior to Coomassie staining.

The concentration of antibody species in solution was determined by UV-vis spectroscopy using a NanoDrop One spectrophotometer. Sample buffer was used as the blank for baseline correction. The following equations were used to determine the concentration of ALC and ADC, accounting for the presence of DVP moieties:

$$[Tras]\ /M = \frac{A_{280} - 0.61A_{298}}{202242}$$

$$[Tras]\ /mgmL^{-1} = \frac{A_{280} - 0.61A_{298}}{1.39}$$

Protein LC-MS was performed on a Xevo G2-S TOF mass spectrometer coupled to an Acquity UPLC system using an Acquity UPLC BEH300 C4 column (1.7 μm, 2.1 × 50 mm). 0.1% Formic acid$_{(aq)}$ (solvent A) and 95% MeCN and 5% 0.1% formic acid$_{(aq)}$ (solvent B) were used as the mobile phase at a flow rate

of 0.2 mL/min. The gradient was programmed as follows: 95% A for 0.93 min, then a gradient to 100% B over 4.28 min, then 100% B for 1.04 minutes, then a gradient to 95% A over 1.04 min. The electrospray source was operated with a capillary voltage of 2.0 kV and a cone voltage of 190 V. Nitrogen was used as the desolvation gas at a total flow rate of 850 L/h. Total mass spectra were reconstructed from the ion series using the MaxEnt 1 algorithm preinstalled on MassLynx 4.2 software according to the manufacturer's instructions. Trastuzumab samples were deglycosylated with PNGase F (New England Biolabs) prior to LC-MS analysis. Only the region of each total ion chromatogram (TIC) containing protein signals was analysed. All calculated values for the masses of trastuzumab conjugates are based on the observed mass ion of native trastuzumab under the same preparation and ionisation conditions (145,171 Da).

Analytical size exclusion chromatography (SEC) was carried out using an Agilent 1260 Infinity system with a Tosoh TSKgel G3000SWXL column (30 cm × 7.8 mm, 5 μm) eluting with PBS (50 mM NaPi, 100 mM NaCl, 0.2% (w/v) sodium azide, pH 7) over 30 minutes at a flow rate of 0.5 mL/min. HPLC was monitored by UV absorbance at 280 nm, and extent of aggregation was determined based on peak area.

Analytical hydrophobic interaction chromatography (HIC) was carried out using an Agilent 1260 Infinity system with a Tosoh TSKgel Butyl-NPR column (3.5 cm × 4.6 mm, 2.5 μm) eluting with a linear gradient system of 0–100% solvent B in solvent A (solvent A: 1.5 M ammonium sulfate, 25 mM NaPi, pH 7; solvent B: 25% (v/v) isopropyl alcohol in 25 mM NaPi, pH 7) over 20 minutes at a flow rate of 0.6 mL/min. HPLC was monitored by UV absorbance at 280 nm, and DAR was calculated based on peak area.

DBCO-PBD dimer reagent **8** was purchased from Levena (SET317).

Figures containing antibodies were generated using Biorender.

2 Synthesis of divinylpyrimidine (DVP) reagent S1

Disulfide rebridging reagent **S1** was synthesised as reported previously.[1] The synthesis is provided here for completeness.

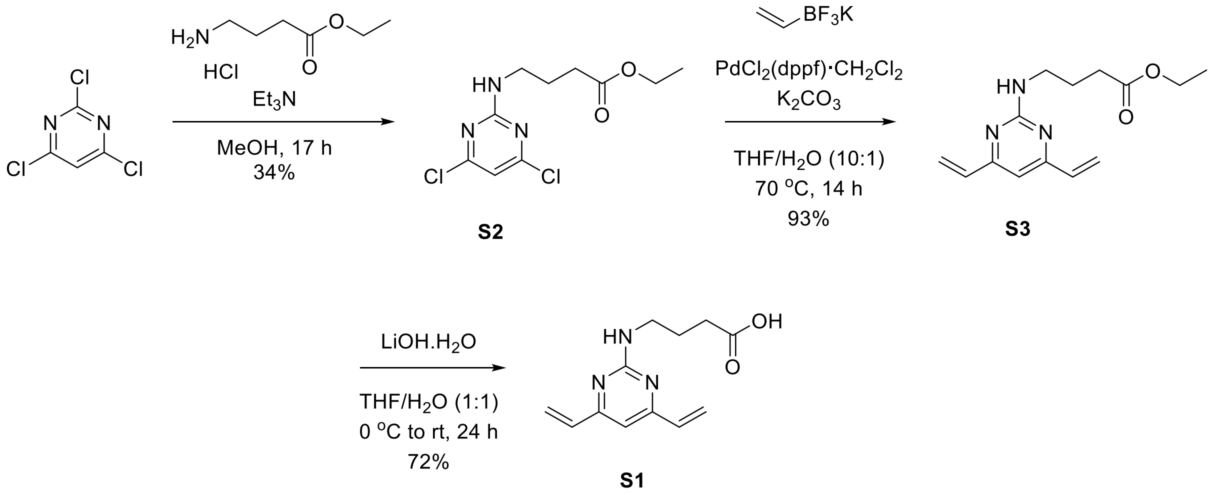

Figure S1: Synthetic route to the disulfide rebridging reagent **S1**.

Ethyl 4-((4,6-dichloropyrimidin-2-yl)amino)butanoate (S2)

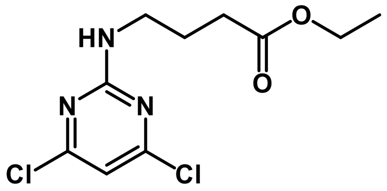

To a solution of ethyl 4-aminobutyrate hydrochloride (2.01 g, 12 mmol) and triethylamine (4.18 mL, 30 mmol) in methanol (100 mL) was added 2,4,6-trichloropyrimidine (1.15 mL, 10 mmol) dropwise. The reaction mixture was stirred for 17 hours, and the solvent removed *in vacuo*. The residue was submitted to flash column chromatography (0–20% EtOAc in pet. ether) to yield the product, ethyl 4-((4,6-dichloropyrimidin-2-yl)amino)butanoate (946 mg, 3.4 mmol, 34%), as a white, needle-like solid.

R$_f$: 0.31 (20% EtOAc in pet. ether); ^{1}**H NMR** (400 MHz, $CDCl_3$, 25 °C): δ (ppm) = 6.59 (s, 1H), 5.56 (app br s, 1H), 4.14 (q, J = 7.1 Hz, 2H), 3.48 (app q, J = 6.5 Hz, 2H), 2.39 (t, J = 7.2 Hz, 2H), 1.93 (app qn, J = 7.0 Hz, 2H), 1.25 (t, J = 7.1 Hz, 3H); ^{13}**C NMR** (100 MHz, $CDCl_3$): δ (ppm) = 173.2, 161.8, 161.5 (C_2), 161.2, 109.2, 60.8, 41.1, 31.7, 24.7, 14.4; **LRMS** (ESI) $C_{10}H_{13}Cl_2N_3O_2$ m/z: $[M+H]^+$ 278.1 (calc. 278.0).

Data agree with those reported in the literature.[1]

Ethyl 4-((4,6-divinylpyrimidin-2-yl)amino)butanoate (S3)

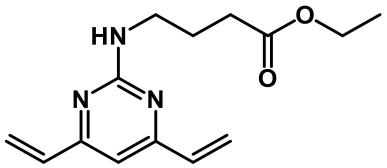

A mixture of ethyl 4-((4,6-dichloropyrimidin-2-yl)amino)butanoate (**S2**) (500 mg, 1.8 mmol), potassium vinyltrifluoroborate (722 mg, 5.4 mmol), $PdCl_2(dppf)\cdot CH_2Cl_2$ (220 mg, 0.27 mmol) and potassium carbonate (745 mg, 5.4 mmol) in THF (22 mL) and water (2.2 mL) was heated to 70 °C for 23 hours. The reaction mixture was cooled to ambient temperature, filtered through Celite and the solvent removed *in vacuo*. The residue was submitted to flash column chromatography (20–30% EtOAc in pet. ether) to yield the product, ethyl 4-((4,6-divinylpyrimidin-2-yl)amino)butanoate (437 mg, 1.67 mmol, 93%), as a pale yellow oil.

R*f*: 0.21 (20% EtOAc in pet. ether); ^{1}**H NMR** (400 MHz, $CDCl_3$, 25 °C): δ (ppm) = 6.58 (dd, *J* = 17.4, 10.6 Hz, 2H), 6.53 (s, 1H), 6.37 (d, *J* = 17.3 Hz, 2H), 5.57 (d, *J* = 10.6 Hz, 2H), 5.29 (app br s, 1H), 4.13 (app q, *J* = 7.1 Hz, 3H), 3.54 (app q, *J* = 6.5 Hz, 2H), 2.41 (t, *J* = 7.4 Hz, 2H), 1.96 (app qn, *J* = 7.1 Hz, 2H), 1.24 (t, *J* = 6.7 Hz, 3H); ^{13}**C NMR** (100 MHz, $CDCl_3$): δ (ppm) = 173.6, 163.8, 162.5, 135.8, 121.9, 105.8, 60.5, 40.9, 31.9, 25.2, 14.4; **LRMS** (ESI) $C_{14}H_{19}N_3O_2$ m/z: $[M+H]^+$ 262.2 (calc. 262.2).

Data agree with those reported in the literature.[1]

The NMR spectra of compound **S3** contain signals from ethyl acetate solvent. At high concentrations, this compound was found to show signs of degradation. After ester hydrolysis, the DVP product (**S1**) is stable, so the dilute, pure intermediate **S3** was carried through without complete evaporation of the purification solvent.

4-((4,6-Divinylpyrimidin-2-yl)amino)butanoic acid (S1)

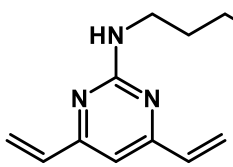

To a solution of ethyl 4-((4,6-divinylpyrimidin-2-yl)amino)butanoate (**S3**) (500 mg, 1.92 mmol) in THF (16 mL) and water (16 mL) was added lithium hydroxide monohydrate (80 mg, 1.92 mmol) and the mixture stirred for 23 hours. Further lithium hydroxide monohydrate (80 mg, 1.92 mmol) was added and the mixture stirred for 1 hour. Further lithium hydroxide monohydrate (80 mg, 1.92 mmol) was added and the mixture stirred for a further 1 hour. The reaction mixture was diluted with water (30 mL) and washed with diethylether (2 × 30 mL). The aqueous layer was adjusted to pH 6 using $HCl_{(aq)}$ (1 M) and extracted with CH_2Cl_2 (3 × 30 mL). The combined organic layers were dried ($MgSO_4$), filtered and concentrated *in vacuo*. The aqueous layers were further acidified to pH 3 using $HCl_{(aq)}$ (1 M) and extracted with CH_2Cl_2 (3 × 30 mL). The combined organic layers were dried ($MgSO_4$), filtered and concentrated *in vacuo* to yield the product, 4-((4,6-divinylpyrimidin-2-yl)amino)butanoic acid (279 mg, 1.2 mmol, 72%), as a white solid.

^{1}**H NMR** (400 MHz, $CDCl_3$, 25 °C): δ (ppm) = 6.84 (app br s, 1H), 6.58 (s, 1H), 6.55 (dd, J = 17.3, 10.7 Hz, 2H), 6.29 (br d, J = 17.0 Hz, 2H), 5.59 (d, J = 10.8 Hz, 2H), 3.59 (app q, J = 5.5 Hz, 2H), 2.46 (t, J = 6.9 Hz, 2H), 2.01 (app qn, J = 6.6 Hz, 2H); ^{13}**C NMR** (100 MHz, $CDCl_3$): δ (ppm) = 177.7, 163.9, 162.0, 135.2, 122.6, 104.0, 40.9, 32.3, 24.9; **LRMS** (ESI) $C_{12}H_{15}N_3O_2$ m/z: $[M+H]^+$ 234.0 (calc. 234.1).

Data agree with those reported in the literature.[1]

3 Synthesis of TetraDVP linkers

Example Tetra DVP: Synthesis of 1a

An example TetraDVP synthesis is given below for **1a**. The synthesis of **1b–d** and **2a–d** are similar, differing only in the length and type of the linkers used between the branch points.

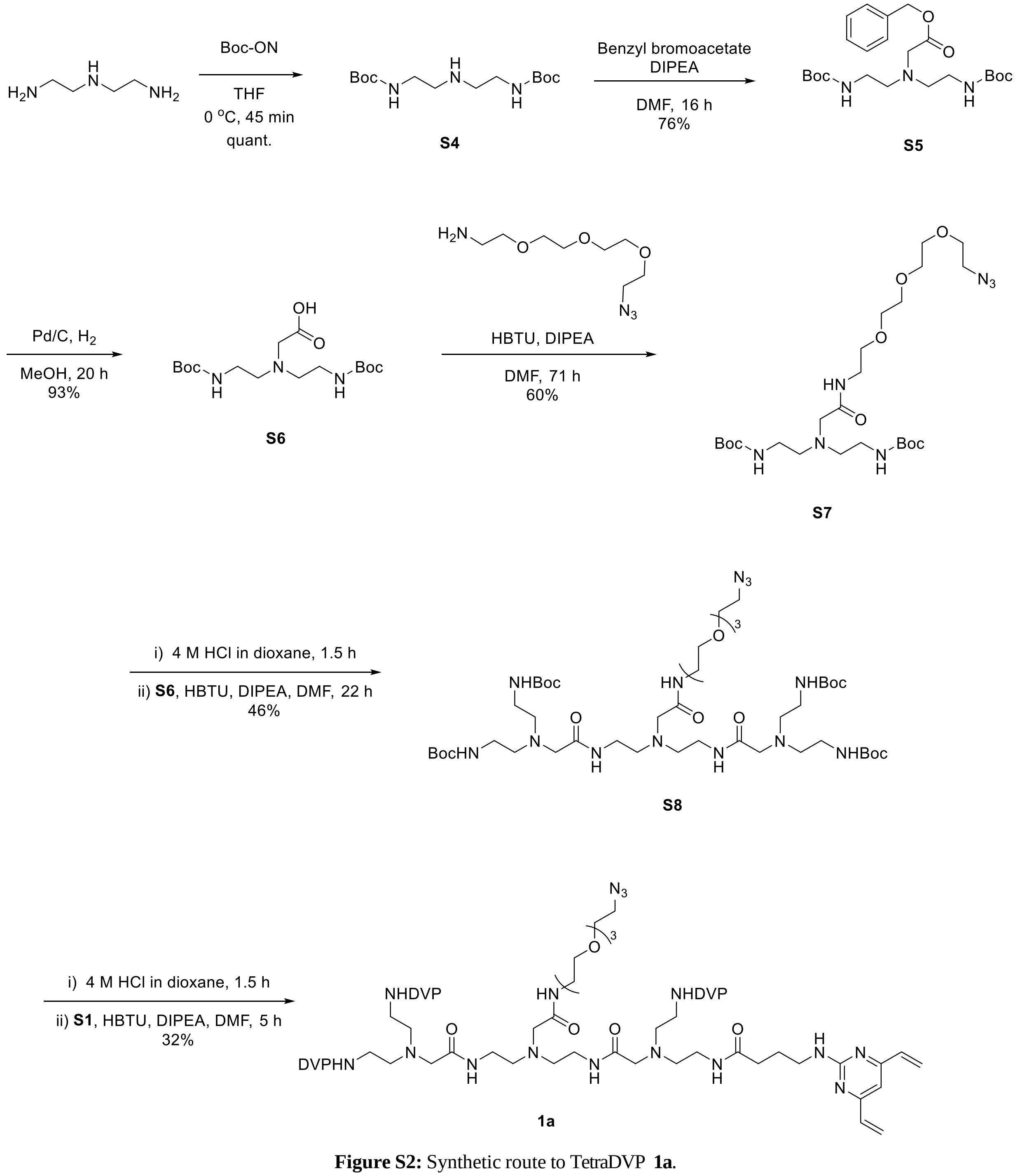

Figure S2: Synthetic route to TetraDVP **1a**.

Di-*tert*-butyl (azanediylbis(ethane-2,1-diyl))dicarbamate (S4)

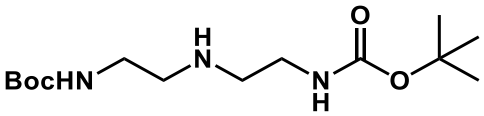

Diethylenetriamine (4.32 mL, 40.0 mmol) was cooled to 0 °C in THF (130 mL) and Boc-ON (19.7 g, 80.0 mmol) was added portion-wise over 15 minutes. The reaction mixture was stirred at 0 °C for 45 minutes. The mixture was concentrated *in vacuo* and submitted to flash column chromatography (0–10% MeOH in CH_2Cl_2) to yield the product, **S4** (12.1 g, 39.9 mmol, quantitative yield), as a viscous, pale-yellow oil.

R*f*: 0.28 (10% MeOH in CH_2Cl_2); ^{1}**H NMR** (400 MHz, $CDCl_3$, 25 °C): δ (ppm) = 4.97 (app br s, 2H), 3.27–3.17 (m, 4H), 2.74 (t, *J* = 5.7 Hz, 4H), 1.44 (s, 18H); ^{13}**C NMR** (100 MHz, $CDCl_3$): δ (ppm) = 156.4, 79.5, 49.0, 40.3, 28.6; **HRMS** (ESI) $C_{14}H_{29}N_3O_4$ m/z: $[M+Na]^+$ 326.2034 (calc. 326.2050).

Data agree with those reported in the literature.[1]

Benzyl bis(2-((*tert*-butoxycarbonyl)amino)ethyl)glycinate (S5)

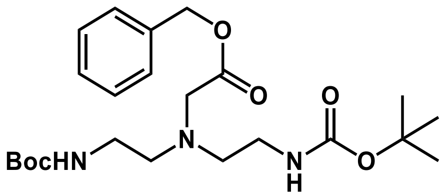

Amine **S4** (4.43 g, 14.6 mmol) was dissolved in DMF (49 mL) and benzyl bromoacetate (3.47 mL, 21.9 mmol) and DIPEA (3.05 mL, 17.5 mmol) were added dropwise. The reaction mixture was stirred for 16 hours. The mixture was diluted with water (850 mL) and extracted with EtOAc (3 × 300 mL). The combined organic extracts were washed with brine (5 × 350 mL) and $LiCl_{(aq)}$ (3 M, 2 × 350 mL), dried ($MgSO_4$), filtered and concentrated *in vacuo*. The crude mixture was submitted to flash column chromatography (0–50% EtOAc/pet. ether) to yield the product, **S5** (4.99 g, 11.1 mmol, 76%), as a colourless oil.

$\mathbf{R}_f$: 0.39 (50% EtOAc/pet. ether); ^{1}**H NMR** (400 MHz, $CDCl_3$, 25 °C): δ (ppm) = 7.39–7.30 (m, 5H), 5.14 (s, 2H), 5.12 (app br s, 2H), 3.42 (s, 2H), 3.14 (app q, J = 5.5 Hz, 4H), 2.73 (t, J = 5.8 Hz, 4H), 1.44 (s, 18H); ^{13}**C NMR** (101 MHz, $CDCl_3$): δ (ppm) = 171.6, 156.3, 135.7, 128.8, 128.6, 128.5, 79.3, 66.6, 55.3, 54.3, 38.8, 28.6; **HRMS** (ESI) $C_{22}H_{35}N_3O_6$ m/z: $[M+Na]^+$ 474.2576 (calc. 474.2574).

Data agree with those reported in the literature.[2]

Bis(2-((*tert*-butoxycarbonyl)amino)ethyl)glycine (S6)

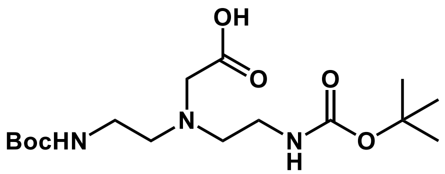

Benzyl ester **S5** (7.78 g, 17.2 mmol) was dissolved in MeOH (170 mL) and degassed with N_2 for 15 minutes. Palladium on carbon (10 wt% Pd, 340 mg) was added and the suspension flushed with H_2 for 10 minutes. The vent needle was removed, and a fresh H_2 balloon applied, and the suspension stirred for 20 hours. The mixture was filtered through Super-Cel, washed with MeOH (2 × 125 mL) and concentrated *in vacuo* to yield the product, **S6** (5.81 g, 16.1 mmol, 93%), as a white solid.

R$_f$: Baseline (50% EtOAc/pet. ether); ^{1}**H NMR** (500 MHz, DMSO–d_6, 25 °C): δ (ppm) = 6.64 (t, J = 5.0 Hz, 2H), 3.27 (s, 2H), 2.95 (app q, J = 6.0 Hz, 4H), 2.60 (t, J = 6.7 Hz, 4H), 1.37 (s, 18H); ^{13}**C NMR** (126 MHz, DMSO–d_6): δ (ppm) = 172.6, 155.6, 77.5, 54.9, 53.4, 38.4, 28.2; **HRMS** (ESI) $C_{16}H_{31}N_3O_6$ m/z: $[M+H]^+$ 362.2271 (calc. 362.2286).

Data agree with those reported in the literature.[2]

Bis-*N*-Boc-triamine with PEG_3 azide side chain (S7)

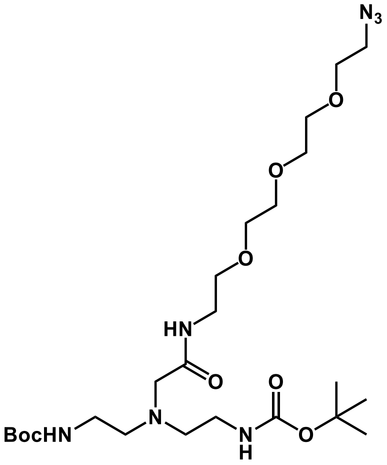

Acid **S6** (100 mg, 0.28 mmol) was dissolved in DMF (1 mL). 11-Azido-3,6,9-trioxaundecanamine (55 μL, 0.28 mmol), HBTU (105 mg, 0.28 mmol) and DIPEA (96 μL, 0.55 mmol) were added, and the reaction mixture stirred for 71 hours. The mixture was diluted with $HCl_{(aq)}$ (1 M, 25 mL) and extracted with EtOAc (3 × 10 mL). The combined organic layers were washed with brine (25 mL), dried (Na_2SO_4), filtered and concentrated *in vacuo*. The crude residue was submitted to flash column chromatography (0–6% MeOH/CH_2Cl_2) to yield the product, **S7** (94 mg, 0.17 mmol, 60%), as a viscous colorless oil.

R_f: 0.27 (6% MeOH/CH_2Cl_2); **^{1}H NMR** (400 MHz, $CDCl_3$, 25 °C): δ (ppm) = 7.47 (app br s, 1H), 5.68 (app br s, 2H), 3.68–3.57 (m, 12H), 3.46 (app q, J = 3.2 Hz, 2H), 3.37 (t, J = 4.9 Hz, 2H), 3.26–2.99 (m, 6H), 2.81–2.43 (m, 4H), 1.44 (s, 18H); **^{13}C NMR** (101 MHz, $CDCl_3$): δ (ppm) = 171.0, 156.7, 79.5, 70.8, 70.7, 70.6, 70.2, 70.1, 69.6, 55.3, 55.3, 50.8, 39.2, 38.3, 28.6; **HRMS** (ESI) $C_{24}H_{47}N_7O_8$ m/z: $[M+Na]^+$ 584.3401 (calc. 584.3378).

Tetra-*N*-Boc no-PEG backbone with PEG_3 azide side chain (S8)

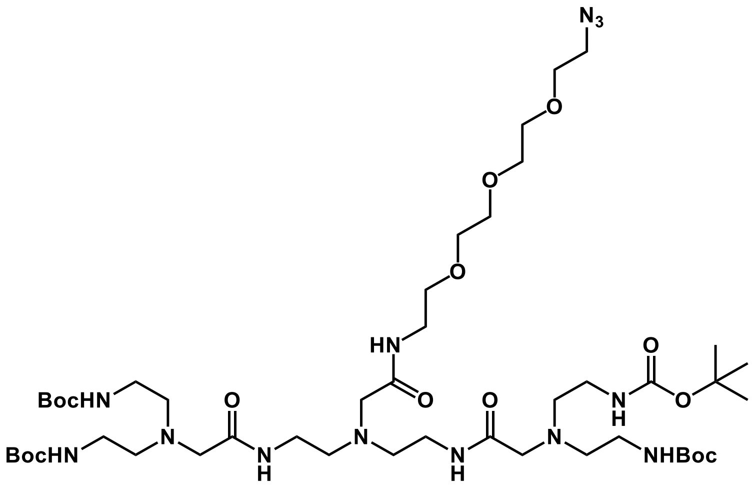

Bis-*N*-Boc amine **S7** (64 mg, 0.11 mmol) was suspended in a solution of HCl (4 M in dioxane, 1 mL) and stirred for 1.5 hours. The solvent was removed under a stream of nitrogen to leave a white solid, which was used without further purification. The intermediate was suspended in DMF (0.5 mL) and DIPEA (119 μL, 0.68 mmol), acid **S6** (103 mg, 0.28 mmol) and HBTU (108 mg, 0.28 mmol) were added. The reaction mixture was stirred for 22 hours. The mixture was diluted with water (20 mL) and extracted with EtOAc (2 × 20 mL). The combined organic extracts were washed with brine (4 × 50 mL), dried (Na_2SO_4), filtered and concentrated. The crude product was submitted to flash column chromatography (0–8% MeOH/CH_2Cl_2) to yield the product, **S8** (53.5 mg, 0.051 mmol, 46%), as a viscous colourless oil.

R$_f$: 0.28 (10% MeOH/CH_2Cl_2); ^{1}**H NMR** (400 MHz, $CDCl_3$, 25 °C): δ (ppm) = 7.71 (app br s, 2H), 5.82 (app br s, 4H), 3.70–3.60 (m, 10H), 3.57 (app br s, 2H), 3.47 (app br s, 3H), 3.38 (t, J = 4.5 Hz, 2H), 3.33 (app br s, 4H), 3.24 (app br s, 2H), 3.17 (app br s, 8H), 3.12 (app br s, 4H), 2.73 (app br s, 4H), 2.58 (app br s, 8H), 1.42 (s, 36H); ^{13}**C NMR** (100 MHz, $CDCl_3$): δ (ppm) = 171.8, 156.7, 79.3, 70.8, 70.7, 70.6, 70.3, 70.1, 69.6, 59.4, 59.0, 55.8, 55.0, 50.8, 39.2, 38.8, 37.7, 28.6; **HRMS** (ESI) $C_{46}H_{89}N_{13}O_{14}$ m/z: $[M+H]^+$ 1048.6742 (calc. 1048.6725).

TetraDVP no-PEG backbone with PEG_3 azide side chain (1a)

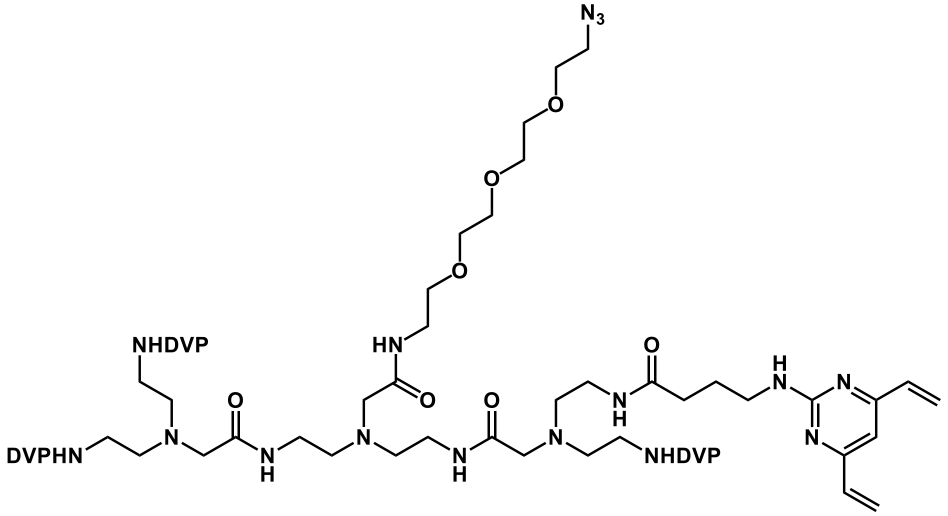

Tetra-*N*-Boc amine **S8** (20 mg, 0.019 mmol) was dissolved in CH_2Cl_2 (1.5 mL) and a solution of HCl (4 M in dioxane, 0.5 mL) was added. The reaction mixture was stirred for 1.5 hours and then the solvent was removed *in vacuo*. The intermediate was redissolved in DMF (1 mL) and acid **S1** (22.3 mg, 0.095 mmol), HBTU (36.2 mg, 0.095 mmol) and DIPEA (33 μL, 0.191 mmol) were added. The reaction mixture was stirred for 3.5 hours. LCMS indicated incomplete conversion, so further acid **S1** (8.9 mg, 0.038 mmol), HBTU (14.5 mg, 0.038 mmol) and DIPEA (13 μL, 0.076 mmol) were added. The mixture was stirred for a further 1.5 hours and then submitted to automated reversed-phase (C_{18}) flash column chromatography (10–70% MeCN in 0.1 M $NH_4OH_{(aq)}$) to yield the product, **1a** (9.05 mg, 6.0 μmol, 32%), as a pale yellow solid.

R$_t$: 8.116 min (5–95% solvent B in solvent A); ^{1}**H NMR** (400 MHz, $CDCl_3$, 25 °C): δ (ppm) = 7.72 (app br s, 3H), 6.55 (dd, *J* = 16.9, 10.1 Hz, 8H), 6.52 (s, 6H), 6.39 (d, *J* = 17.3 Hz, 8H), 5.62 (d, *J* = 10.5 Hz, 8H), 3.65–3.55 (m, 14H), 3.54–3.47 (m, 11H), 3.42 (q, *J* = 5.5 Hz, 4H), 3.39–3.29 (m, 20H), 2.85–2.62 (m, 11H), 2.38 (t, *J* = 7.1 Hz, 8H), 1.94 (app qn, *J* = 6.6 Hz, 8H); ^{13}**C NMR** (125 MHz, $CDCl_3$): δ (ppm) = 174.3, 163.4, 161.6, 136.4, 131.3, 121.3, 116.4, 107.9, 105.2, 70.7, 70.7, 70.6, 70.6, 70.6, 70.2, 70.1, 70.1, 69.4, 59.0, 55.9, 53.8, 50.8, 48.2, 46.9, 40.9, 39.3, 37.3, 33.9, 26.3, 25.7, 24.8, 24.4; **HRMS** (ESI) $C_{74}H_{109}N_{25}O_{10}$ m/z: $[M+H]^+$ 1508.8787 (calc. 1508.8862).

Bis-(N-Fmoc-PEG$_2$)-triamine with PEG$_3$ azide side chain (S9)

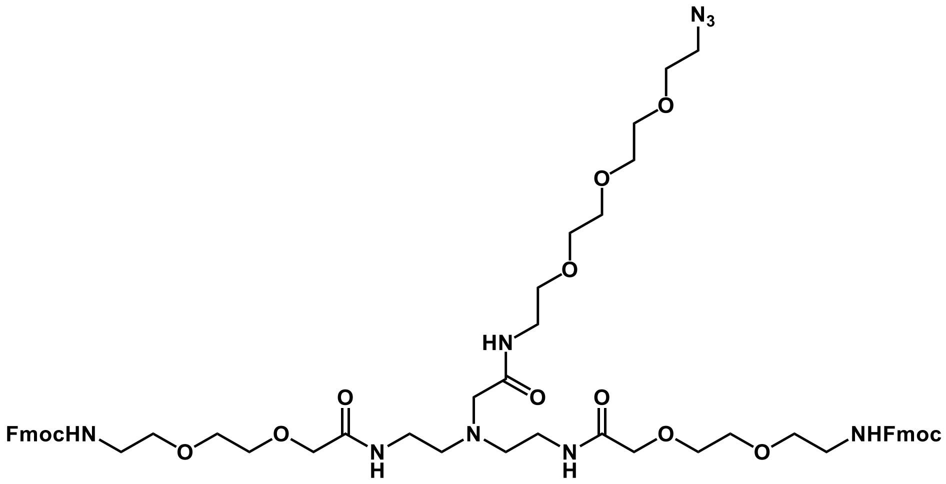

Bis-*N*-Boc amine **S7** (385 mg, 0.69 mmol) was dissolved in a solution of HCl (4 M in dioxane, 4 mL) and CH_2Cl_2 (2 mL) and stirred for 3 hours. The solvent was removed under a stream of nitrogen, and the resulting residue was redissolved in DMF (2.5 mL). Fmoc-8-Amino-3,6-dioxaoctanoic acid (532 mg, 1.38 mmol), HBTU (523 mg, 1.38 mmol) and DIPEA (721 μL, 4.14 mmol) were added, and the reaction mixture stirred for 17 hours. Some acid starting material remained, so further HBTU (261 mg, 0.69 mmol) and DIPEA (120 μL, 0.69 mmol) were added and the mixture stirred for a further 1 hour. The mixture was submitted directly to reverse-phase column chromatography (10–100% MeCN in H_2O) to yield the product, **S9** (378 mg, 0.34 mmol, 50%), as a viscous orange oil.

$\mathbf{R}_f$: 0.18 (7% MeOH in CH_2Cl_2); ^{1}**H NMR** (500 MHz, $CDCl_3$, 25 °C): δ (ppm) = 7.75 (d, J = 7.5 Hz, 4H), 7.60 (d, J = 7.2 Hz, 4H), 7.39 (t, J = 7.5 Hz, 4H), 7.30 (t, J = 7.4 Hz, 4H), 7.18 (br s, 2H), 5.71 (app br s, 2H), 4.40 (d, J = 6.4 Hz, 4H), 4.20 (t, J = 6.7 Hz, 2H), 3.99 (s, 4H), 3.68–3.51 (m, 24H), 3.43 (q, J = 5.6 Hz, 2H), 3.40–3.30 (m, 10H), 3.15 (s, 2H), 2.64 (t, J = 6.0 Hz, 4H); ^{13}**C NMR** (125 MHz, $CDCl_3$): δ (ppm) = 171.4, 170.5, 156.8, 144.1, 141.5, 127.8, 127.2, 125.2, 120.1, 71.1, 70.8, 70.7, 70.6, 70.6, 70.4, 70.2, 70.1, 70.1, 69.7, 66.7, 59.2, 54.8, 50.8, 47.4, 41.0, 39.0, 37.2; **HRMS** (ESI) $C_{56}H_{73}O_{14}N_9$ m/z: $[M+H]^+$ 1096.5346 (calc. 1096.5350).

Tetra-*N*-Boc backbone with PEG$_2$ units inside the branch point and PEG$_3$ azide side chain (S10)

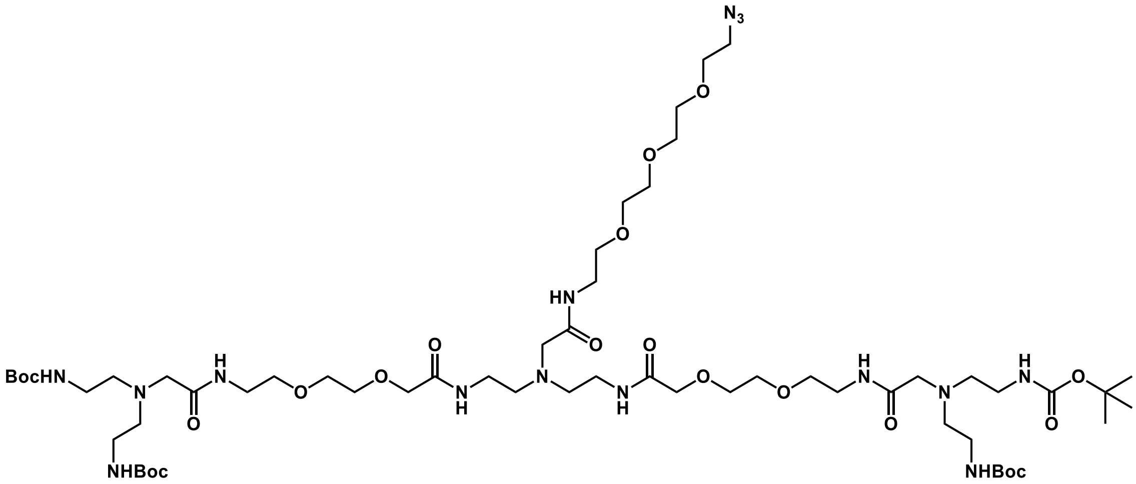

Bis-*N*-Fmoc amine **S9** (161 mg, 0.15 mmol) was dissolved in DMF (2 mL) and piperidine (116 μL, 1.18 mmol) was added. The mixture was stirred for 1 hour. The solvent was removed under a stream of nitrogen to leave a white solid. The residue was suspended in DMF (1.5 mL) and acid **S6** (133 mg, 0.37 mmol), HBTU (139 mg, 0.37 mmol) and DIPEA (128 μL, 0.73 mmol) were added. The reaction mixture was stirred for 1.5 hours. The crude mixture was submitted to reverse-phase column chromatography (10–100% MeCN/H_2O) to yield the product, **S10** (118 mg, 0.08 mmol, 59%), as a pale-yellow solid.

^{1}**H NMR** (500 MHz, $CDCl_3$, 25 °C): δ (ppm) = 7.49 (br s, 2H), 7.22 (app br s, 1H), 5.60 (s, 2H), 4.09–3.93 (m, 4H), 3.70–3.59 (m, 25H), 3.57 (t, *J* = 4.8 Hz, 4H), 3.50–3.41 (m, 7H), 3.38 (t, *J* = 5.0 Hz, 6H), 3.27–3.03 (m, 10H), 2.82–2.48 (m, 8H), 2.16 (app br s, 2H), 1.44 (s, 36H); ^{13}**C NMR** (125 MHz, $CDCl_3$): δ (ppm) = 171.0, 163.2, 156.6, 152.0, 79.7, 71.0, 70.8, 70.7, 70.6, 70.3, 70.1, 70.1, 69.6, 69.5, 55.9, 55.6, 55.1, 54.8, 53.7, 50.8, 47.2, 41.0, 39.2, 28.6; **HRMS** (ESI) $C_{58}H_{111}N_{15}O_{20}$ m/z: $[M+2H]^{2+}$ 669.9153 (calc. 669.9138).

TetraDVP backbone with PEG_2 units inside the branch point and PEG_3 azide side chain (1b)

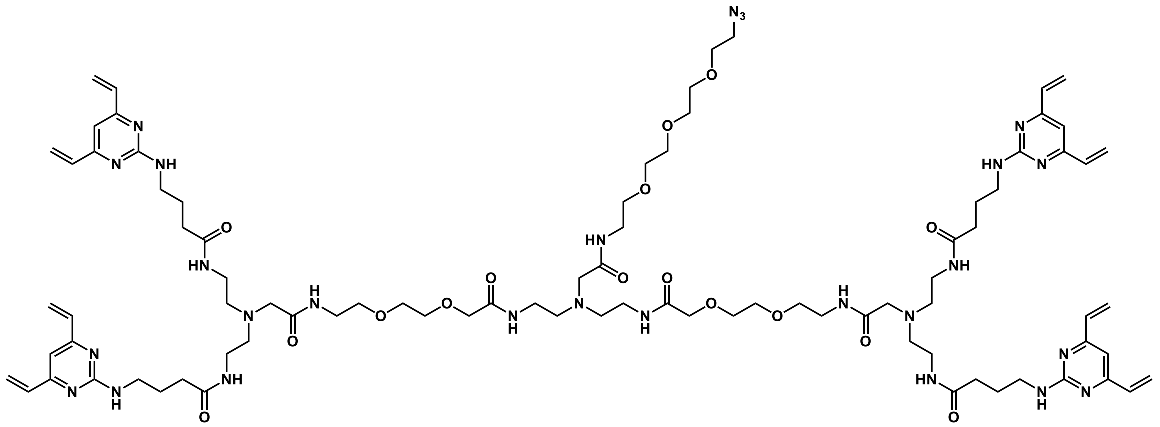

Tetra-*N*-Boc amine **S10** (25 mg, 0.019 mmol) was dissolved in a solution of HCl (4 M in dioxane, 1 mL) and stirred for 1 hour. The solvent was removed under a stream of nitrogen, acid **S1** (26.1 mg, 0.112 mmol), HBTU (42.5 mg, 0.112 mmol) were added and the combined solids suspended in DMF (0.75 mL). DIPEA (59 μL, 0.336 mmol) was added and the reaction mixture stirred for 1 hour. The mixture was submitted to reverse-phase column chromatography (10–100% MeCN in 0.1 M $NH_4OH_{(aq)}$) to yield the product, **1b** (9.71 mg, 5.4 μmol, 28%), as a brown solid.

R$_f$: 7.278 min (5–95% solvent B in solvent A); ^{1}**H NMR** (500 MHz, DMSO–d$_6$, 25 °C): δ (ppm) = 7.80–7.72 (m, 7H), 7.68 (t, *J* = 5.7 Hz, 2H), 7.04 (t, *J* = 5.6 Hz, 4H), 6.76 (s, 4H), 6.58 (dd, *J* = 17.3, 10.6 Hz, 8H), 6.35 (d, *J* = 16.6 Hz, 8H), 5.58 (dd, *J* = 10.3, 1.4 Hz, 8H), 3.85 (s, 4H), 3.61–3.57 (m, 2H), 3.56–3.45 (m, 16H), 3.44–3.36 (m, 9H), 3.29 (q, *J* = 6.6 Hz, 9H), 3.24 (q, *J* = 5.9 Hz, 6H), 3.17 (q, *J* = 6.3 Hz, 4H), 3.11 (q, *J* = 6.2 Hz, 8H), 3.08–3.03 (m, 6H), 2.56 (t, *J* = 6.8 Hz, 4H), 2.53–2.49 (m, 8H, overlap with solvent), 2.14 (t, *J* = 7.7 Hz, 8H), 1.76 (app qu, *J* = 7.2 Hz, 8H), 0.95 (d, *J* = 6.5 Hz, 3H); ^{13}**C NMR** (125 MHz, DMSO–d$_6$): δ (ppm) = 172.1, 170.6, 170.5, 169.2, 163.1, 162.4, 136.1, 121.4, 104.5, 70.1, 70.0, 69.8, 69.7, 69.7, 69.5, 69.3, 69.2, 69.0, 58.3, 58.0, 54.2, 54.1, 50.0, 40.3, 38.1, 36.8, 36.4, 33.1, 25.3; **HRMS** (ESI) $C_{86}H_{132}O_{16}N_{27}$ m/z: $[M+H]^+$ 1799.0355 (calc. 1799.0340).

Tetra-*N*-Fmoc backbone with PEG_2 units outside the branch point and PEG_3 azide side chain (S11)

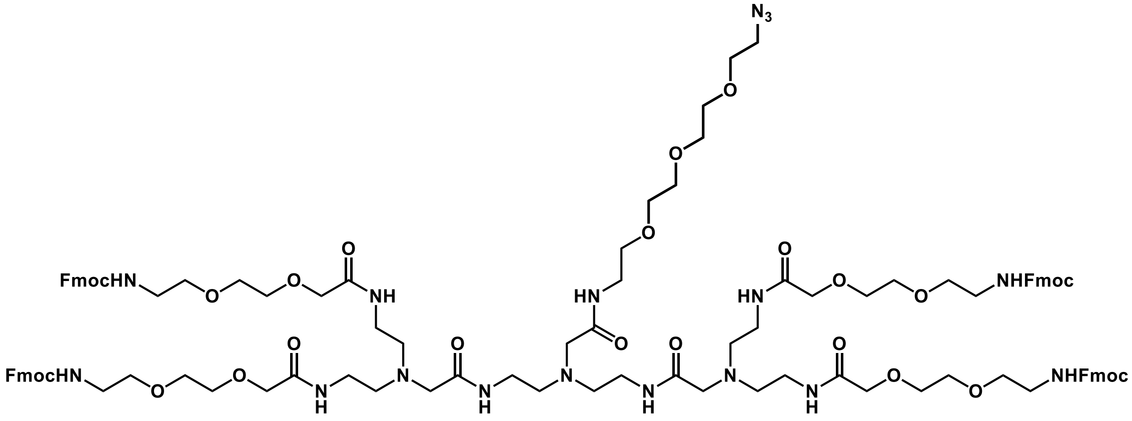

Tetra *N*-Boc amine **S8** (35 mg, 0.033 mmol) was suspended in a solution of HCl (4 M in dioxane, 1 mL) and stirred for 1.5 hours. The solvent was removed under a stream of nitrogen to leave a white solid, which was used without further purification. The intermediate was suspended in DMF (0.3 mL) and Fmoc-8-amino-3,6-dioxaoctanoic acid (51 mg, 0.13 mmol), HBTU (50 mg, 0.13 mmol) and DIPEA (70 μL, 0.40 mmol) were added. The reaction mixture was stirred for 18 hours. The mixture was diluted with water (20 mL) and extracted with EtOAc (2 × 20 mL). The combined organic extracts were washed with $LiCl_{(aq)}$ (5 wt%, 30 mL), dried (Na_2SO_4), filtered and concentrated. The crude product was submitted to flash column chromatography (4–10% MeOH/CH_2Cl_2) to yield the product, **S11** (13 mg, 5.9 μmol, 18%), as a white solid.

R*f*: 0.10 (10% MeOH/CH_2Cl_2); ^{1}**H NMR** (400 MHz, MeOD, 25 °C): δ (ppm) = 7.76 (d, *J* = 7.5 Hz, 8H), 7.61 (d, *J* = 7.4 Hz, 8H), 7.36 (t, *J* = 7.4 Hz, 8H), 7.27 (t, *J* = 7.4 Hz, 8H), 4.58 (s, 2H), 4.46 (app br s, 1H), 4.32 (d, *J* = 6.8 Hz, 7H), 4.16 (t, *J* = 6.7 Hz, 4H), 3.94 (s, 8H), 3.64–3.57 (m, 17H), 3.56–3.53 (m, 8H), 3.53–3.43 (m, 10H), 3.36 (t, *J* = 5.4 Hz, 2H), 3.27 (t, *J* = 5.9 Hz, 18H), 3.20 (s, 2H), 3.18 (s, 4H), 3.05 (app br s, 1H), 2.66 (t, *J* = 6.5 Hz, 4H), 2.61 (t, *J* = 6.2 Hz, 8H); ^{13}**C NMR** (101 MHz, MeOH–d_4): δ (ppm) = 174.2, 172.7, 158.8, 145.3, 142.6, 128.8, 128.2, 126.2, 121.0, 72.0, 71.6, 71.5, 71.4, 71.2, 71.1, 71.0, 67.7, 59.7, 55.9, 55.6, 48.5, 41.7, 40.0, 38.3; **HRMS** (ESI) $C_{110}H_{141}N_{17}O_{26}$ m/z: $[M+H]^+$ 2117.0291 (calc. 2117.0310).

TetraDVP backbone with PEG_2 units outside the branch point and PEG_3 azide side chain (1c)

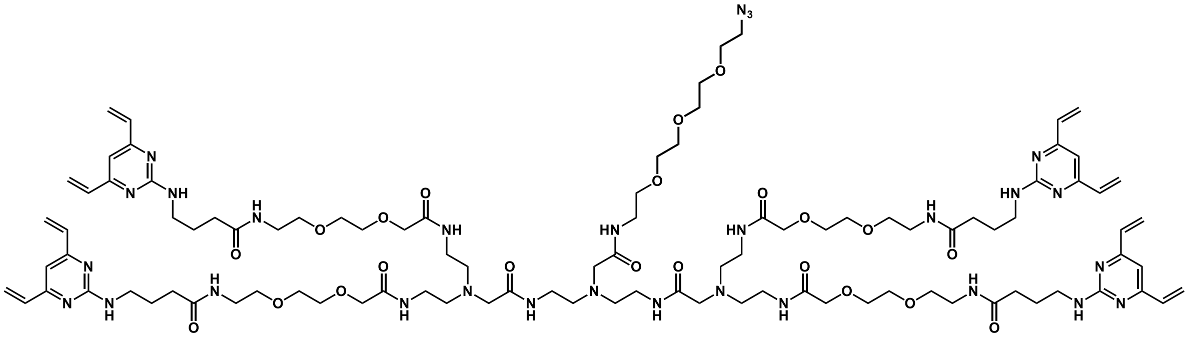

Tetra-*N*-Fmoc amine **S11** (10 mg, 4.7 μmol) was dissolved in DMF (0.3 mL) and piperidine (4.6 μL, 0.047 mmol) was added. The mixture was stirred for 2 hours. The solvent was removed under a stream of nitrogen to leave a white solid, which was used without further purification. The intermediate was suspended in DMF (0.3 mL) and acid **S1** (5.0 mg, 0.021 mmol), HBTU (8 mg, 0.021 mmol) and DIPEA (7.4 μL, 0.043 mmol) were added. The reaction mixture was stirred for 2 hours and then submitted directly to automated reversed-phase (C_{18}) flash column chromatography (10–80% MeCN in water). Fractions containing product were combined and lyophilised to yield the product, **1c** (1.9 mg, 0.89 μmol, 19%), as an off-white solid.

R*t*: 7.675 min (5–95% solvent B in solvent A); ^{1}**H NMR** (400 MHz, DMSO–d_6, 25 °C): δ (ppm) = 7.85 (t, *J* = 5.5 Hz, 4H), 7.81–7.74 (m, 3H), 7.69 (t, *J* = 5.6 Hz, 4H), 7.04 (t, *J* = 5.5 Hz, 4H; NH), 6.76 (s, 4H), 6.57 (dd, *J* = 17.3, 10.7 Hz, 8H), 6.34 (d, *J* = 16.1 Hz, 8H), 5.57 (dd, *J* = 10.5, 1.6 Hz, 8H), 3.85 (s, 8H), 3.59–3.46 (m, 29H), 3.40 (t, *J* = 5.7 Hz, 8H), 3.23–3.11 (m, 23H), 3.07 (app br s, 6H), 2.67 (t, *J* = 1.8 Hz, 3H), 2.59–2.53 (m, 7H), 2.34–2.31 (m, 5H), 2.13 (t, *J* = 7.6 Hz, 11H), 1.74 (app qn, *J* = 7.4 Hz, 11H); ^{13}**C NMR** (125 MHz, DMSO–d_6): δ (ppm) = 172.2, 170.7, 169.3, 143.7, 136.2, 124.3, 124.0, 121.5, 121.1, 104.5, 70.2, 70.0, 69.8, 69.8, 69.7, 69.6, 69.4, 69.3, 69.2, 58.1, 54.0, 50.0, 38.5, 36.5, 33.1, 25.4; **HRMS** (ESI) $C_{98}H_{153}O_{22}N_{29}$ m/z: $[M+H]^+$ 2089.1782 (calc. 2089.1818).

Tetra-*N*-Fmoc backbone with PEG_2 units inside and outside the branch point and PEG_3 azide side chain (S12)

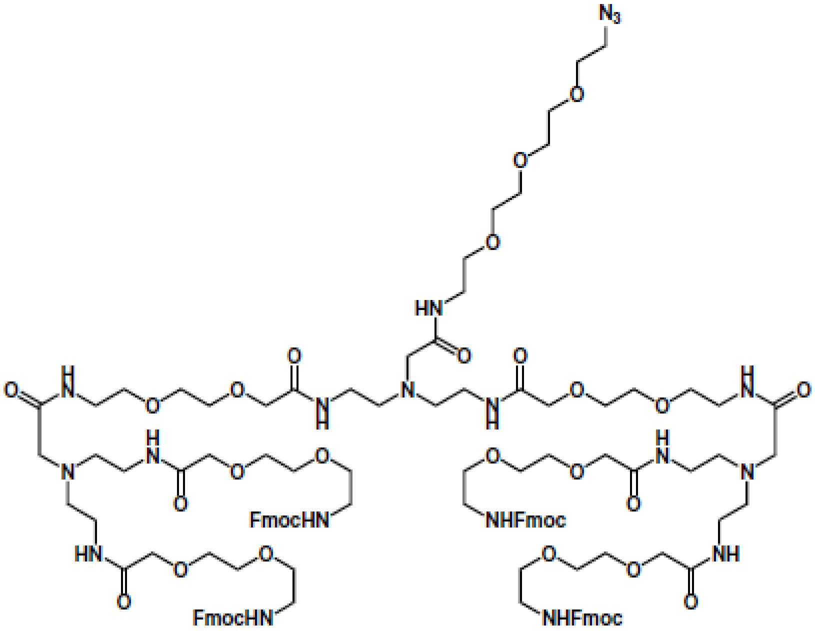

Tetra-*N*-Boc amine **S10** (102 mg, 0.076 mmol) was dissolved in a solution of HCl (4 M in dioxane, 2 mL) and stirred for 1 hour. The solvent was removed under a stream of nitrogen, and the resulting white solid re-dissolved in DMF (2 mL). Fmoc-8-Amino-3,6-dioxaoctonoic acid (176 mg, 0.46 mmol), HBTU (173 mg, 0.46 mmol) and DIPEA (239 μL, 1.37 mmol) were added and the reaction mixture stirred for 19.5 hours. The mixture was submitted to reverse-phase column chromatography (10–100% MeCN in H_2O) to yield the product, **S12** (50.6 mg, 0.021 mmol, 28%), as a white solid.

^{1}**H NMR** (500 MHz, DMSO – d_6, 25 °C): δ (ppm) = 7.87 (d, J = 7.5 Hz, 8H), 7.77–7.71 (m, 3H), 7.70–7.65 (m, 13H), 7.40 (t, J = 7.4 Hz, 8H), 7.34–7.29 (m, 11H), 4.28 (d, J = 6.9 Hz, 8H), 4.20 (t, J = 6.8 Hz, 4H), 3.86 (s, 7H), 3.85 (s, 4H), 3.58–3.47 (m, 32H), 3.44–3.39 (m, 13H), 3.36 (t, J = 4.9 Hz, 2H), 3.24 (q, J = 5.6 Hz, 6H), 3.19–3.11 (m, 19H), 3.07 (s, 6H), 2.56 (t, J = 6.7 Hz, 11H); ^{13}**C NMR** (100 MHz, DMSO–d_6): δ (ppm) = 170.6, 169.7, 169.3, 169.2, 142.6, 139.4, 137.4, 128.9, 127.3, 121.4, 120.0, 70.2, 70.2, 70.0, 69.8, 69.7, 69.7, 69.5, 69.3, 69.0, 58.0, 54.1, 50.0, 40.3, 40.1, 38.1, 36.4; **HRMS** (ESI) $C_{122}H_{164}O_{32}N_{19}$ m/z: $[M+H]^+$ 2407.1743 (calc. 2407.1784).

TetraDVP backbone with PEG_2 units inside and outside the branch point and PEG_3 azide side chain (1d)

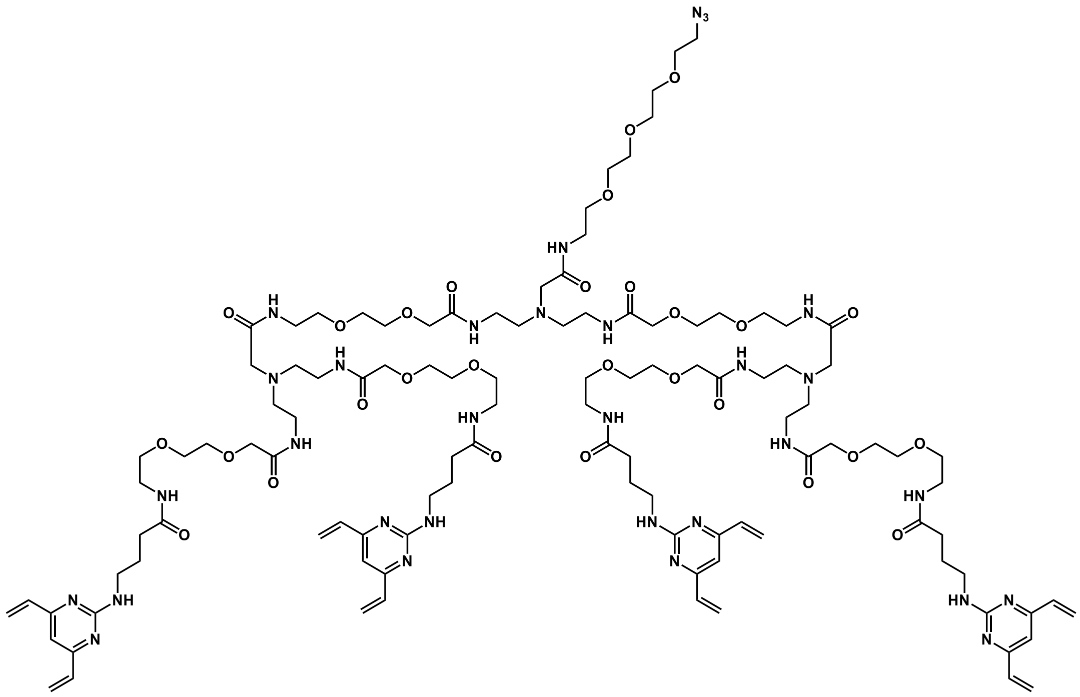

Tetra-*N*-Fmoc amine **S12** (24 mg, 0.01 mmol) was dissolved in DMF (1 mL) and piperidine (9.9 μL, 0.10 mmol) was added. The mixture was stirred for 30 minutes, and the solvent was removed under a stream of nitrogen. The crude residue was redissolved in DMF (0.75 mL). **S1** (14 mg, 0.06 mmol), HBTU (23 mg, 0.06 mmol) and DIPEA (31 μL, 0.18 mmol) were added and the reaction mixture was stirred for 2 hours. The crude mixture was submitted to reverse-phase flash column chromatography (0–100% MeCN in 0.1 M $NH_4OH_{(aq)}$) to yield the product, **1d** (8.6 mg, 3.6 μmol, 36%), as a pale brown solid.

R$_f$: 7.080 min (5–95% solvent B in solvent A); ^{1}**H NMR** (500 MHz, DMSO–d_6, 25 °C): δ (ppm) = 7.86 (t, J = 5.5 Hz, 4H; NH), 7.78–7.72 (m, 3H; NH), 7.70–7.63 (m, 6H; NH), 7.04 (t, J = 5.7 Hz, 4H), 6.76 (s, 4H), 6.57 (dd, J = 10.7, 17.2 Hz, 8H), 6.35 (d, J = 16.8 Hz, 8H), 5.57 (dd, J = 1.4, 10.7 Hz, 8H), 3.86 (s, 12H), 3.60–3.47 (m, 36H), 3.45–3.36 (m, 17H), 3.30–3.22 (m, 18H), 3.22–3.13 (m, 21H), 3.07 (s, 6H), 2.56 (t, J = 6.4 Hz, 10H), 2.13 (t, J = 7.5 Hz, 8H), 1.74 (app qn, J = 7.2 Hz, 8H); ^{13}**C NMR** (125 MHz, DMSO–d_6): δ (ppm) = 172.1, 170.6, 170.5, 169.2, 169.2, 163.2, 162.4, 136.1, 121.4, 104.5, 70.2, 70.0, 69.8, 69.7, 69.7, 69.5, 69.3, 69.3, 69.2, 69.1, 69.0, 58.0, 54.1, 50.0, 40.3, 38.4, 38.1, 36.4, 33.0, 25.3; **HRMS** (ESI) $C_{110}H_{175}O_{28}N_{31}$ m/z: $[M+H]^+$ 2379.3252 (calc. 2379.3296).

Late-stage intermediates in the synthesis of TetraDVPs **2a**, **2c** and **2d**, were supplied by Apollo Therapeutics. A representative complete synthesis of TetraDVP **2b** is provided for reference.

Bis(2-((((9*H*-fluoren-9-yl)methoxy)carbonyl)amino)ethyl)glycine (S13)

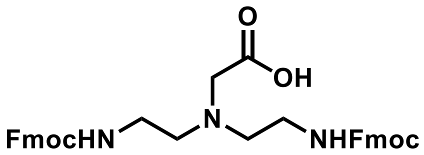

Diethylene triamine (157 μL, 1.45 mmol) was diluted in DMF (4 mL) and cooled to 0 °C. A solution of Fmoc-OSu (981 mg, 2.90 mmol) in DMF (2 mL) was added portion-wise, and the reaction mixture was stirred at 0 °C for 30 minutes. The mixture was diluted with CH_2Cl_2 (100 mL) and washed with $NaHCO_{3(aq)}$ (100 mL), $LiCl_{(aq)}$ (3 M, 100 mL), dried ($MgSO_4$), filtered and concentrated *in vacuo*. α-bromo acetic acid (242 mg, 1.74 mmol) was added, followed by DIPEA (530 μL, 3.05 mmol) and the reaction stirred for 2 hours. The reaction mixture was diluted with CH_2Cl_2 (100 mL) and $HCl_{(aq)}$ (1 M, 100 mL) and then extracted with CH_2Cl_2 containing a few drops of MeOH (3 × 50 mL). The combined organic extracts were washed with brine (200 mL), dried ($MgSO_4$), filtered and concentrated *in vacuo*. The mixture was submitted to reverse-phase column chromatography (20–70% MeCN in 0.5% formic $acid_{(aq)}$) to yield the product, **S13** (197 mg, 0.32 mmol, 22%), as a white solid.

R_f: 0.09 (10% MeOH in CH_2Cl_2); **^{1}H NMR** (400 MHz, MeOH–d4, 25 °C): δ (ppm) = 7.74 (d, J = 7.3 Hz, 4H), 7.58 (d, J = 7.3 Hz, 4H), 7.34 (t, J = 7.4 Hz, 4H), 7.24 (t, J = 7.3 Hz, 4H), 4.32 (d, J = 6.8 Hz, 4H), 4.13 (t, J = 6.7 Hz, 2H), 3.63 (s, 2H), 3.42–3.36 (m, 4H), 3.21–3.14 (m, 4H); **^{13}C NMR** (100 MHz, DMSO–d_6): δ (ppm) = 166.9, 156.3, 143.8, 140.8, 127.7, 127.1, 125.1, 120.2, 65.7, 54.9, 53.0, 46.7, 37.9; **LRMS** (ESI) $C_{36}H_{35}N_3O_6$ m/z: $[M+H]^+$ 606.7 (calc. 606.3).

Data agree with those reported in the literature.[3]

Bis((9*H*-fluoren-9-yl)methyl) (8,16-dioxo-3,6,18,21-tetraoxa-9,12,15-triazatricosane-1,23-diyl)dicarbamate (S14)

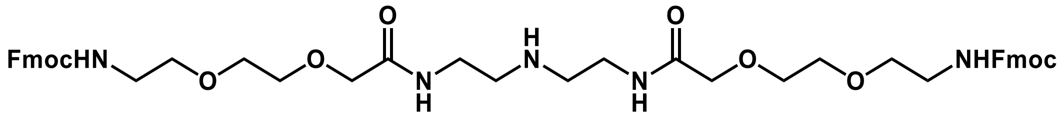

Diethylene triamine (0.5 mL, 4.6 mmol) was diluted in DMF (15 mL). Fmoc-8-Amino-3,6-dioxaoctanoic acid (3.75 g, 9.7 mmol) was added, followed by HBTU (3.86 g, 10.2 mmol) and DIPEA (2.42 mL, 13.9 mmol) and the reaction mixture was stirred for 2 hours. The reaction was diluted with water (100 mL), and extracted with EtOAc (3 × 75 mL). The combined organic extracts were washed with $LiCl_{(aq)}$ (3 M, 100 mL) and then dried ($MgSO_4$), filtered and concentrated onto Celite *in vacuo* and submitted to flash column chromatography (0–10% MeOH/CH_2Cl_2) to yield the product, **S14** (2.92 g, 3.5 mmol, 76%), as a foamy white solid.

R*f*: 0.23 (8% MeOH/CH_2Cl_2); ^{1}**H NMR** (400 MHz, $CDCl_3$, 25 °C): δ (ppm) = 8.14 (app br s, 2H), 7.73 (d, *J* = 7.5 Hz, 4H), 7.62 (br s, 2H), 7.57 (d, *J* = 7.4 Hz, 4H), 7.37 (t, *J* = 7.4 Hz, 4H), 7.28 (t, *J* = 7.5 Hz, 4H), 5.42 (app br s, 1H), 4.36 (d, *J* = 6.4 Hz, 4H), 4.18 (t, *J* = 6.5 Hz, 2H), 3.97 (s, 4H), 3.66–3.40 (m, 16H), 3.37–3.14 (m, 8H); ^{13}**C NMR** (100 MHz, $CDCl_3$): δ (ppm) = 173.9, 155.9, 144.0, 141.4, 127.9, 127.3, 125.2, 120.1, 70.9, 70.0, 69.8, 69.6, 67.0, 48.3, 47.2, 40.8, 36.4; **HRMS** (ESI) $C_{46}H_{55}N_5O_{10}$ m/z: $[M+H]^+$ 838.4004 (calc. 838.4022).

***tert*-Butyl (3-(2-bromoacetamido)propyl)carbamate (S15)**

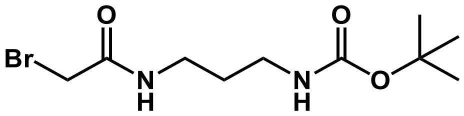

N-Boc-1,3-propanediamine (4.00 g, 22.9 mmol) was dissolved in CH_2Cl_2 (40 mL) and cooled to -10 °C. Bromoacetyl bromide (1.0 mL, 11.5 mmol) was added dropwise and the reaction mixture allowed to warm to 10 °C over 40 minutes with stirring. The mixture was filtered, diluted with CH_2Cl_2 (50 mL) and washed with $HCl_{(aq)}$ (1 M, 100 mL) and brine (100 mL). The organic layer was dried ($MgSO_4$), filtered and concentrated *in vacuo*. The crude residue was submitted to flash column chromatography (0–2% MeOH in CH_2Cl_2) to yield the product, **S15** (1.93 g, 6.54 mmol, 57%) as a white solid.

R*f*: 0.19 (2% MeOH/CH_2Cl_2); ^{1}**H NMR** (400 MHz, $CDCl_3$, 25 °C): δ (ppm) = 7.07 (app br s, 1H), 4.83 (app br s, 1H), 3.87 (s, 2H), 3.34 (app q, J = 5.8 Hz, 2H), 3.22–3.14 (m, 2H), 1.66 (app qn, J = 6.3 Hz, 2H), 1.44 (s, 9H); ^{13}**C NMR** (100 MHz, $CDCl_3$): δ (ppm) = 166.2, 156.8, 79.7, 37.2, 36.9, 30.1, 29.3, 28.5; **LRMS** (ESI) $C_{10}H_{19}\,^{81}BrN_2O_3$ m/z: $[M+H]^+$ 297.1 (calc. 297.1).

Data agree with those reported in the literature.[4]

Bis((9*H*-fluoren-9-yl)methyl) (12-(2-((3-((*tert*-butoxycarbonyl)amino) propyl)amino)-2-oxoethyl)-8,16-dioxo-3,6,18,21-tetraoxa-9,12,15-triazatricosane-1,23-diyl)dicarbamate (S16)

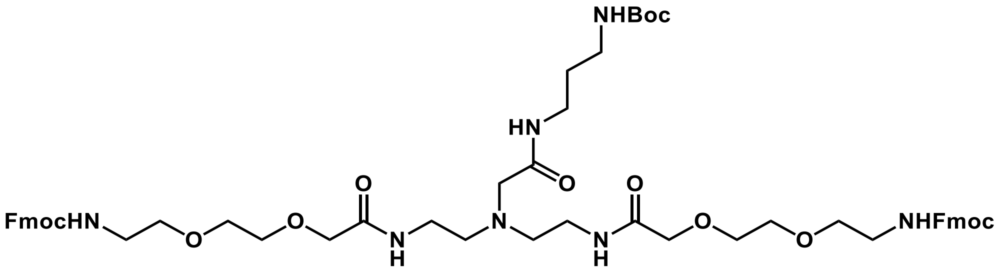

To a solution of amine **S14** (240 mg, 0.29 mmol) in DMF (2 mL) was added α-bromo amide **S15** (110 mg, 0.37 mmol) and DIPEA (65 μL, 0.37 mmol) and the reaction mixture was stirred for 28 hours. The reaction mixture was diluted with CH_2Cl_2 (40 mL) and washed with $LiCl_{(aq)}$ (3 M, 2 × 40 mL). The organic layer was dried ($MgSO_4$), filtered and concentrated. The crude product was submitted to flash column chromatography (0–10% MeOH in CH_2Cl_2) to yield the product, **S16** (139 mg, 0.13 mmol, 45%), as a white solid.

R$_f$: 0.18 (7% MeOH/CH_2Cl_2); ^{1}**H NMR** (400 MHz, DMSO-d_6, 25 °C): δ (ppm) = 7.87 (d, J = 7.5 Hz, 4H), 7.74 (t, J = 6.0 Hz, 1H), 7.70 (t, J = 5.6 Hz, 2H), 7.67 (d, J = 7.6 Hz, 4H), 7.40 (t, J = 7.4 Hz, 4H), 7.31 (t, J = 7.3 Hz, 6H), 6.75 (t, J = 5.9 Hz, 1H), 4.28 (d, J = 6.9 Hz, 4H), 4.19 (t, J = 6.6 Hz, 2H), 3.86 (s, 4H), 3.58–3.51 (m, 8H), 3.44–3.38 (m, 4H, overlaps with solvent), 3.17 (app q, J = 6.5 Hz, 4H), 3.14 (app q, J = 6.1 Hz, 4H), 3.06 (app q, J = 6.5 Hz, 2H), 3.04 (s, 2H), 2.91 (app q, J = 6.4 Hz, 2H), 2.54 (t, J = 6.6 Hz, 4H), 1.49 (app qn, J = 6.7 Hz, 2H), 1.35 (s, 9H); ^{13}**C NMR** (101 MHz, DMSO-d_6): δ (ppm) = 170.6, 169.4, 156.3, 155.7, 144.0, 140.8, 127.7, 127.1, 125.2, 120.2, 77.6, 70.3, 70.1, 69.4, 69.2, 65.4, 58.2, 54.1, 46.8, 40.2, 37.5, 36.5, 36.0, 29.8, 28.3; **HRMS** (ESI) $C_{56}H_{73}N_7O_{13}$ m/z: $[M+H]^+$ 1052.5354 (calc. 1052.5339).

Tetra-*N*-Fmoc backbone with PEG_2 units inside the branch point and *N*-Boc-amine side chain (S17)

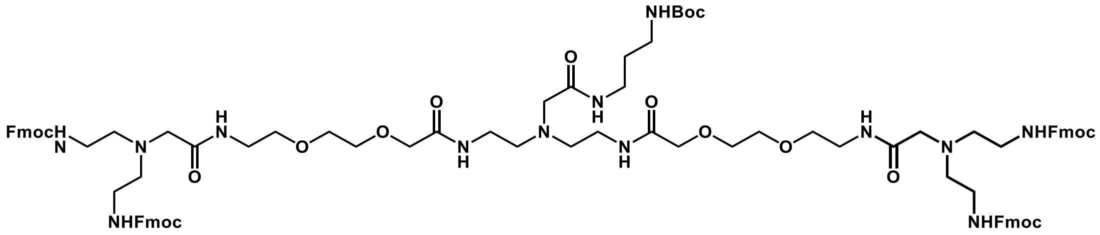

Bis-*N*-Fmoc-amine **S16** (158 mg, 0.15 mmol) was dissolved in DMF (1.5 mL). Piperidine (37 μL, 0.38 mmol) was added and the mixture stirred for 1.5 hours. The solvent was removed under a stream of nitrogen and then the crude residue was redissolved in DMF (1.5 mL). Acid **S13** (227 mg, 0.38 mmol), HBTU (142 mg, 0.38 mmol) and DIPEA (65 μL, 0.38 mmol) were added and the mixture stirred for 17 hours. The crude mixture was submitted directly to reverse-phase purification (10–100% MeCN in H_2O) to yield the product, **S17** (72 mg, 0.04 mmol, 27%), as a white solid.

R$_t$: 10.961 min (5–95% solvent B in solvent A); ^{1}**H NMR** (500 MHz, $CDCl_3$, 25 °C): δ (ppm) = 7.77–7.67 (m, 8H), 7.64–7.48 (m, 9H), 7.43–7.32 (m, 10H), 7.30–7.22 (m, 10H, overlaps with solvent), 4.47–4.41 (m, 10H), 4.21–4.14 (m, 6H), 4.07–3.95 (m, 5H), 3.83–3.48 (m, 31H), 3.45–3.31 (m, 6H), 3.28–3.05 (m, 7H), 1.68–1.60 (m, 2H), 1.42–1.35 (m, 9H); ^{13}**C NMR** (126 MHz, $CDCl_3$): δ (ppm) = 171.9, 164.3, 157.1, 144.0, 143.9, 141.5, 141.4, 127.9, 127.9, 127.2, 125.4, 125.2, 125.0, 120.2, 120.1, 79.6, 70.8, 70.6, 70.5, 70.4, 70.1, 69.1, 67.4, 67.3, 53.3, 52.6, 47.3, 39.6, 38.0, 36.8, 36.6, 34.6, 29.8, 29.2, 28.6, 24.3; **HRMS** (ESI) $C_{98}H_{119}N_{13}O_{19}$ m/z: $[M+H]^+$ 1782.9805 (calc. 1782.9820).

Tetra-*N*-Fmoc backbone with PEG$_2$ units inside the branch point and PEG$_4$ azide side chain (S18)

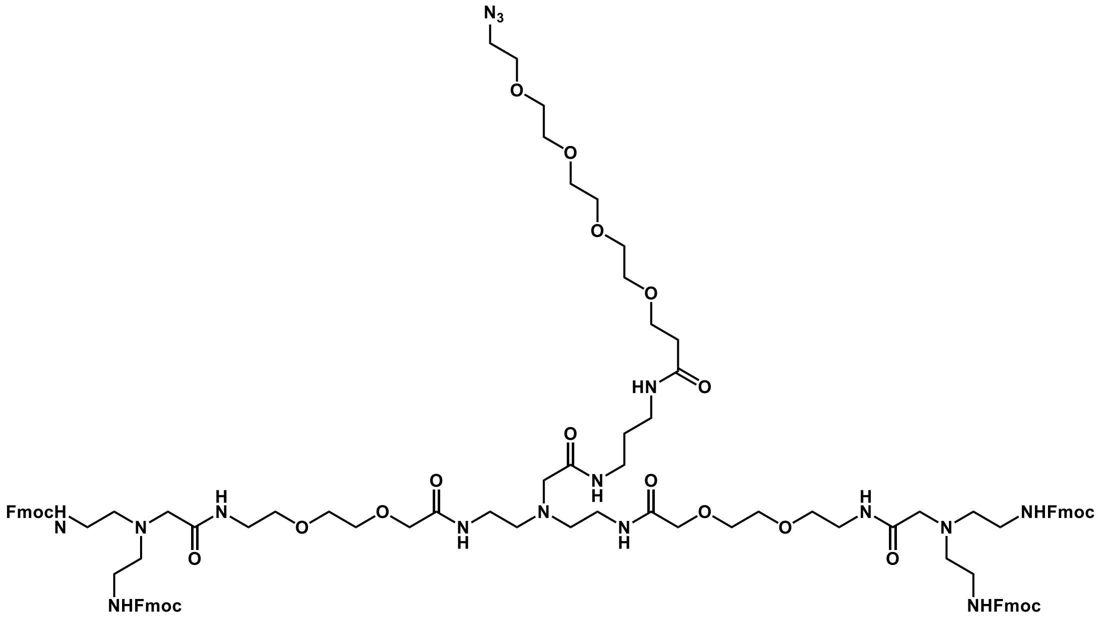

tert-Butyl ester **S24** (49 mg, 0.14 mmol) was dissolved in CH_2Cl_2 (0.25 mL) and a solution of HCl (4 M in dioxane, 0.25 mL) was added. The solution was stirred for 24 hours. *N*-Boc amine **S17** (65 mg, 0.036 mmol) was dissolved in CH_2Cl_2 (0.25 mL) and a solution of HCl (4 M in dioxane, 0.25 mL) was added. The mixture was stirred for 1 hour. Both solutions were evaporated to dryness under nitrogen. The crude acid was redissolved in DMF (0.5 mL) and the solution added to the solid amine HCl salt. HBTU (64 mg, 0.17 mmol) and DIPEA (29 μL, 0.17 mmol) were added and the mixture stirred for 3 hours. The crude mixture was submitted directly to reversed-phase purification (10–100% MeCN in H_2O) to yield the product, **S18** (54 mg, 0.028 mmol, 77%), as an off-white solid.

R$_t$: 10.957 min (5–95% solvent B in solvent A); ^{1}**H NMR** (400 MHz, $CDCl_3$, 25 °C): δ (ppm) = 7.78–7.69 (m, 8H), 7.65–7.49 (m, 8H), 7.43–7.32 (m, 9H), 7.31–7.21 (m, 10H, overlaps with solvent), 4.39–4.29 (m, 9H), 4.25–4.12 (m, 6H), 3.99 (br s, 4H), 3.80–3.47 (m, 44H), 3.40–3.32 (m, 6H), 3.24 (app q, J = 6.3 Hz, 6H), 2.42–2.36 (m, 2H); **HRMS** (ESI) $C_{104}H_{130}N_{16}O_{22}$ m/z: $[M+3H]^{3+}$ 652.6594 (calc. 652.6588).

TetraDVP backbone with PEG_2 units inside the branch point and PEG_4 azide side chain (2b)

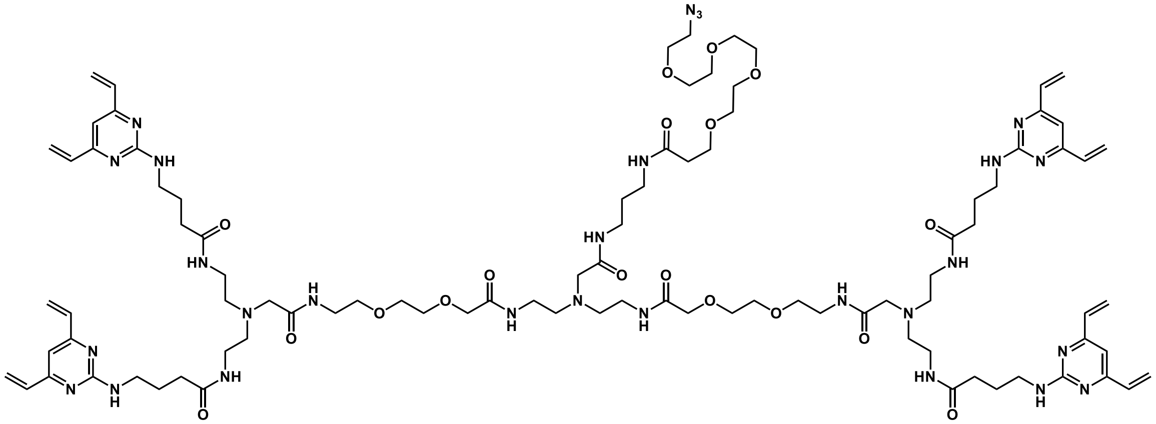

Tetra-*N*-Fmoc amine **S18** (25 mg, 0.013 mmol) was dissolved in DMF (0.5 mL). Piperidine (6.3 μL, 0.064 mmol) was added and the mixture stirred for 1 hour. The solvent was removed under a stream of nitrogen. DVP acid (**S1**) (18 mg, 0.077 mmol) and HBTU (29 mg, 0.077 mmol) were added and the mixture was dissolved in DMF (0.5 mL). DIPEA (13 μL, 0.077 mmol) was added and the mixture stirred for 3.5 hours. The crude mixture was submitted directly to reversed-phase chromatography (10–100% MeCN in H_2O) to yield the product, **2b** (5.28 mg, 2.74 μmol, 21%), as a pale yellow solid.

R$_t$: 7.647 min (5–95% solvent B in solvent A); **HRMS** (ESI) $C_{92}H_{140}N_{28}O_{18}$ m/z: $[M+H]^+$ 1926.0890 (calc. 1926.0973).

TetraDVP no-PEG backbone with PEG_4 azide side chain (2a)

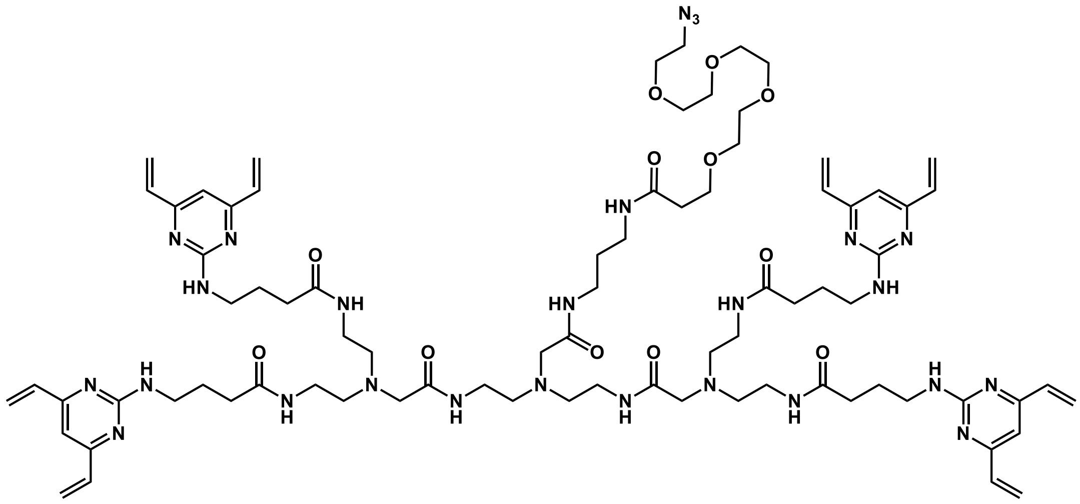

Tetra-*N*-Boc-amine precursor (18 mg, 0.015 mmol) was dissolved with a solution of HCl (4 M in dioxane, 1 mL) and the mixture was stirred for 1 hour. The solvent was removed under a stream of nitrogen, and then the off-white solid residue was redissolved in DMF (1 mL). DVP acid (**S1**) (18 mg, 0.076 mmol), HATU (23 mg, 0.061 mmol), and DIPEA (53 μL, 0.31 mmol) were added and the mixture was stirred for 2 hours. The crude mixture was submitted directly to reverse-phase purification (10–70% MeCN in 0.1 M $NH_4OH_{(aq)}$) to yield the product, **2a** (1.59 mg, 0.97 μmol, 6%), as a yellow solid.

R$_t$: 8.171 min (5–95% solvent B in solvent A); **HRMS** (ESI) $C_{80}H_{118}N_{26}O_{12}$ m/z: $[M+H]^+$ 1635.9434 (calc. 1635.9496).

TetraDVP backbone with PEG_2 units outside the branch point and PEG_4 azide side chain (2c)

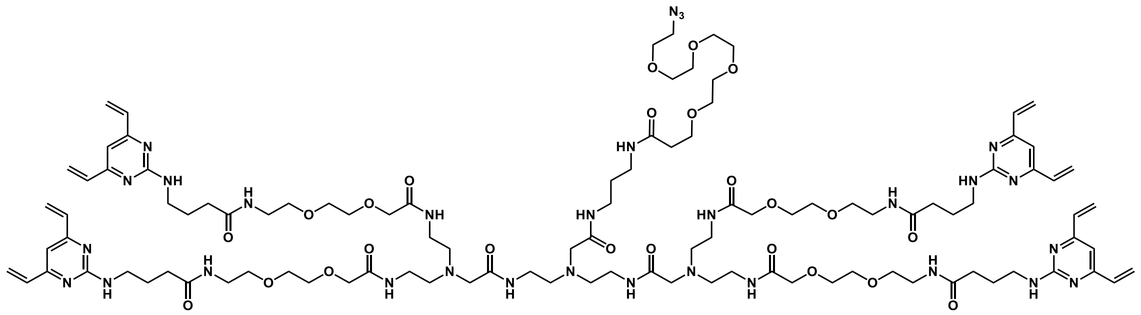

Tetra-*N*-Boc-amine precursor (20 mg, 0.011 mmol) was dissolved with a solution of HCl (4 M in dioxane, 1 mL) and the mixture was stirred for 1.5 hours. The solvent was removed under a stream of nitrogen, and then the crude residue was redissolved in DMF (1.5 mL). DVP acid (**S1**) (13 mg, 0.057 mmol), HATU (17 mg, 0.046 mmol), and DIPEA (30 μL, 0.17 mmol) were added and the mixture was stirred for 1 hour. The crude mixture was submitted directly to reverse-phase purification (10–70% MeCN in 0.1 M $NH_4OH_{(aq)}$) to yield the product, **2c** (2.06 mg, 0.93 μmol, 8%), as a yellow solid.

R$_t$: 7.393 (5–95% solvent B in solvent A); **HRMS** (ESI) $C_{104}H_{162}N_{30}O_{24}$ m/z: $[M+Na]^+$ 2238.2192 (calc. 2238.2270).

TetraDVP backbone with PEG_2 units inside and outside the branch point and PEG_4 azide side chain (2d)

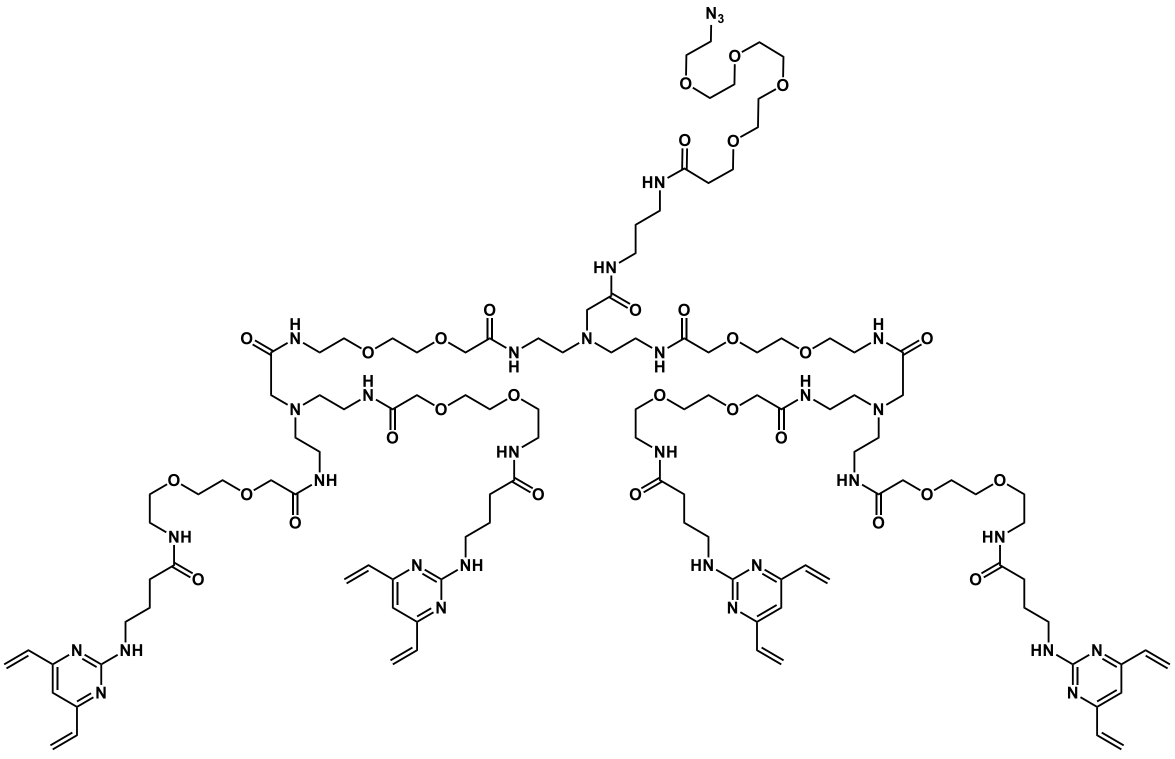

Tetra-*N*-Boc-amine precursor (20 mg, 9.8 μmol) was dissolved with a solution of HCl (4 M in dioxane, 1.5 mL) and the mixture was stirred for 1 hour. The solvent was removed under a stream of nitrogen, and then the crude residue was redissolved in DMF (1 mL). DVP acid (**S1**) (11 mg, 0.049 mmol), HATU (15 mg, 0.039 mmol), and DIPEA (26 μL, 0.15 mmol) were added and the mixture was stirred for 1 hour. The crude mixture was submitted directly to reverse-phase purification (20–60% MeCN in 0.1 M $NH_4OH_{(aq)}$) to yield the product, **2d** (1.89 mg, 0.75 μmol, 8%), as a pale brown solid.

R$_t$: 7.204 min (5–95% solvent B in solvent A); **HRMS** (ESI) $C_{116}H_{184}N_{32}O_{30}$ m/z: $[M+2]^{2+}$ 1253.7000 (calc. 1253.7001).

4 Synthesis of DBCO reagent 5

Synthesis towards Fmoc-Val-Cit-PAB-MMAE has been reported in the literature.[5] Full synthesis towards DBCO reagent **5** is provided below. It is important to note that MMAE is a potent cytotoxin and must be handled with care. We recommend performing any reactions involving unmodified MMAE on small scale and notifying others in the vicinity that MMAE is in use.

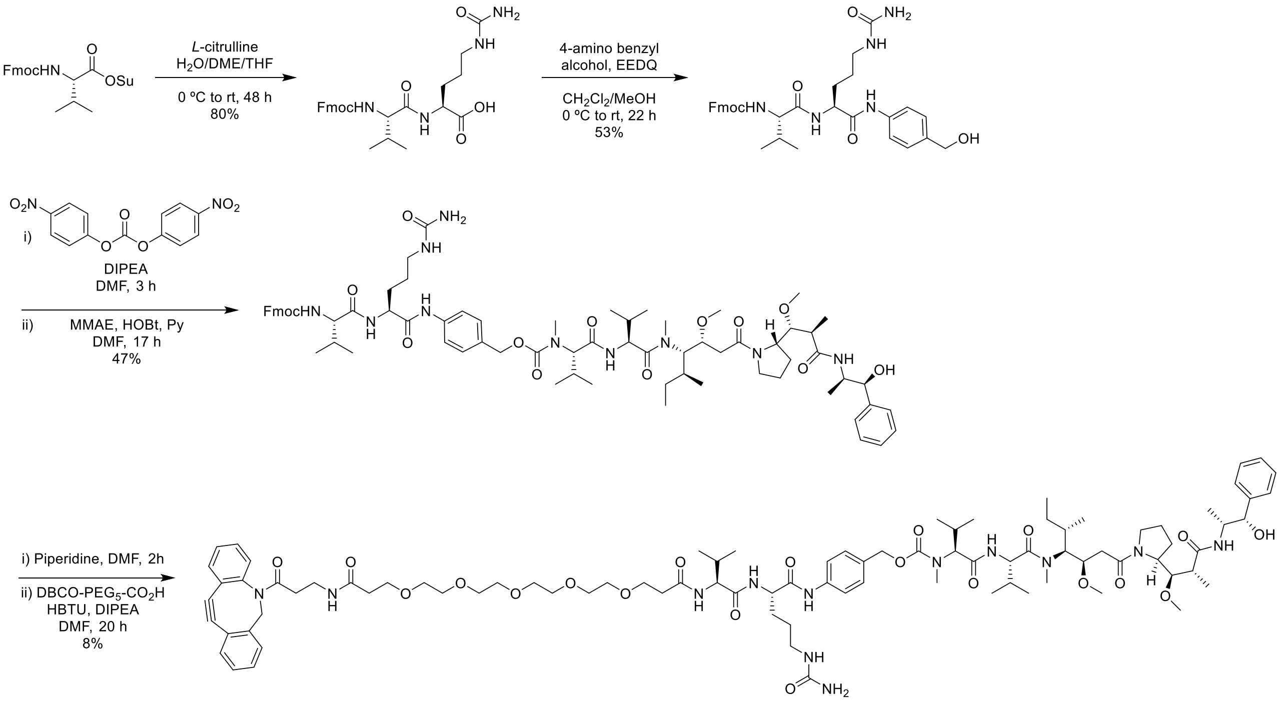

5

Figure S3: Synthetic route to DBCO reagent **5**.

Fmoc-Val-Cit-OH (S19)

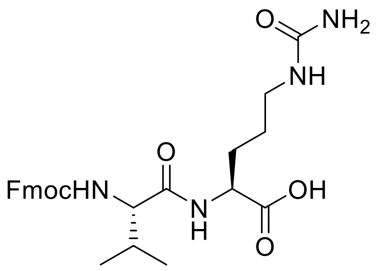

L-citrulline (85 mg, 0.486 mmol) in DME (1.5 mL) was added to a solution of Fmoc-Val-OSu (202 mg, 0.463 mmol) and $NaHCO_{3\,(aq)}$ (42.8 mg, 0.509 mmol) in H_2O (3 mL) and THF (4 mL) at 0 °C. The reaction warmed to rt and stirred for 48 hours. Upon completion, the reaction was adjusted to pH 10 with sat. aq. K_2CO_3 and washed with EtOAc (2 × 20 mL). The aqueous layer was acidified to pH 4 with 10% aq. citric acid and the formed gelatinous mixture was filtered and dried *in vacuo* to yield Fmoc-Val-Cit-OH, **S19** (193 mg, 0.389 mmol, 80%), as an off-white solid.

^{1}H NMR (700 MHz, DMSO–d$_6$, 25 °C): δ (ppm) = 7.89 (d, J = 7.6 Hz, 2H), 7.75 (dd, J = 11.7, 7.8 Hz, 2H), 7.41 (t, J = 7.4 Hz, 2H), 7.34–7.30 (m, 2H), 6.00 (s, 1H), 4.31–4.26 (m, 1H), 4.27–4.19 (m, 2H), 4.17–4.13 (m, 1H), 3.94–3.87 (m, 1H), 2.99–2.92 (m, 2H), 1.98 (app sx, J = 6.8 Hz, 1H), 1.74–1.66 (m, 1H), 1.61–1.53 (m, 1H), 1.46–1.35 (m, 2H), 0.89 (d, J = 6.8 Hz, 3H), 0.86 (d, J = 6.8 Hz, 3H); **^{13}C NMR** (176 MHz, DMSO–d$_6$): δ (ppm) = 173.4, 171.3, 158.8, 156.1, 143.9, 143.9, 140.7, 127.7, 127.1, 125.4, 120.1, 65.7, 59.8, 51.9, 46.7, 38.8, 30.6, 28.4, 26.6, 19.2, 18.2; **HRMS** (ESI) $C_{26}H_{32}N_4O_6$ m/z: $[M+H]^+$ 497.2394 (calc. 497.2395).

Data agree with those reported in the literature.[5]

Fmoc-Val-Cit-PABA (S20)

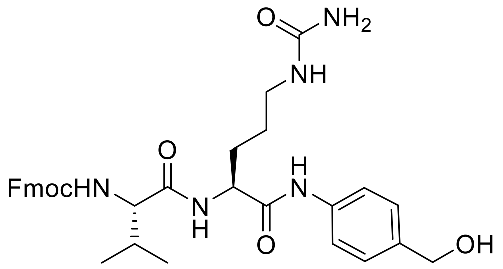

A solution of Fmoc-Val-Cit-OH (**S19**) (104 mg, 0.210 mmol), 4-aminobenzyl alcohol (52.0 mg, 0.419 mmol) and 2-ethoxy-1-ethoxycarbonyl-1,2-dihydroquinoline (104 mg, 0.419 mmol) in DCM (2.5 mL) and MeOH (1.5 mL) was stirred for 22 hours. Upon completion, the mixture was diluted with Et_2O (10 mL), filtered and washed with Et_2O (2 x 4 mL) to yield Fmoc-Val-Cit-PABA, **S20** (66.4 mg, 0.110 mmol, 53%), as a white solid.

^{1}H NMR (700 MHz, DMSO–d_6, 25 °C): δ (ppm) = 9.98 (s, 1H), 8.12 (d, *J* = 7.5 Hz, 1H), 7.89 (d, *J* = 7.6 Hz, 2H), 7.74 (t, *J* = 8.1 Hz, 2H), 7.54 (d, *J* = 8.4 Hz, 2H), 7.46–7.39 (m, 3H), 7.34–7.30 (m, 2H), 7.23 (d, *J* = 8.4 Hz, 2H), 6.00 (t, *J* = 5.8 Hz, 1H), 5.41 (s, 2H), 5.09 (t, *J* = 5.5 Hz, 1H), 4.43–4.39 (m, 3H), 4.33–4.20 (m, 3H), 3.95–3.91 (m, 1H), 3.06–2.89 (m, 2H), 2.04–1.95 (m, 1H), 1.74–1.55 (m, 2H), 1.50–1.32 (m, 2H), 0.89–0.84 (m, 6H); **^{13}C NMR** (176 MHz, DMSO–d_6): δ (ppm) = 171.3, 170.4, 158.9, 156.1, 143.9, 140.7, 137.5, 137.4, 127.7, 127.1, 126.9, 125.4, 120.1, 118.9 , 65.7, 62.6, 60.1, 53.1, 46.7, 38.6, 30.5, 29.5, 26.8, 19.2, 18.3; **HRMS** (ESI) $C_{33}H_{39}N_5O_6$ m/z: $[M+H]^+$ 602.2968 (calc. 602.2973).

Data agree with those reported in the literature.[5]

Fmoc-Val-Cit-PABC-MMAE (S21)

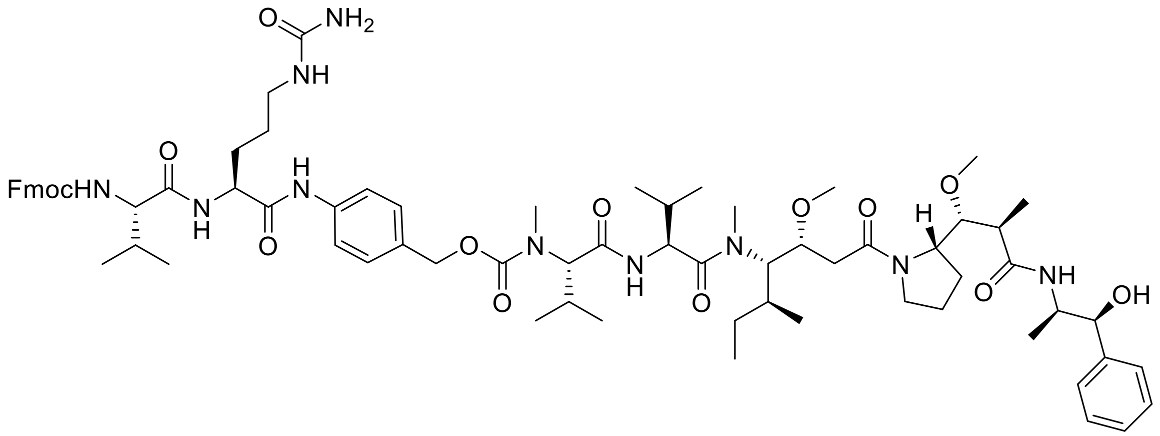

A solution of Fmoc-Val-Cit-PABA (**S20**) (200 mg, 0.332 mmol), bis(4-nitrophenyl) carbonate (202 mg, 0.665 mmol) and DIPEA (86.8 µL, 0.498 mmol) was stirred at rt for 3 hours. Upon completion, the mixture was concentrated under a stream of N_2. The crude residue was precipitated with EtOAc (3 mL) and Et_2O (30 mL), allowed to stand for 30 minutes and then filtered to yield Fmoc-Val-Cit-PAB-PNP as a light brown solid, which was carried through without further purification.

A solution of MMAE (25.0 mg, 34.8 µmol), Fmoc-Val-Cit-PAB-PNP (53.4 mg, 69.6 µmol), HOBt (9.40 mg, 69.6 µmol) and pyridine (28.2 µL, 348 µmol) in DMF (1.5 mL) was stirred at rt for 17 hours. Upon completion, the reaction mixture was concentrated under a stream of N_2. The crude residue was dissolved in CH_2Cl_2/MeOH (30 mL, 3:2), filtered and the filtrate purified by flash column chromatography (0–10% MeOH in CH_2Cl_2) to yield Fmoc-Val-Cit-PAB-MMAE, **S21** (22.0 mg, 16.4 µmol, 47%), as a white solid.

$\mathbf{R}_t$: 13.281 min (5–95% solvent B in solvent A); **HRMS** (ESI) $C_{73}H_{104}N_{10}O_{14}$ m/z: $[M+H]^+$ 1345.7795 (calc. 1345.7806).

Data agree with those reported in the literature.[5]

WARNING! MMAE is a potent cytotoxin and must be handled with care. We recommend performing any reactions involving unmodified MMAE on small scale and notifying others in the vicinity that MMAE is in use.

DBCO-PEG$_5$-Val-Cit-PAB-MMAE (5)

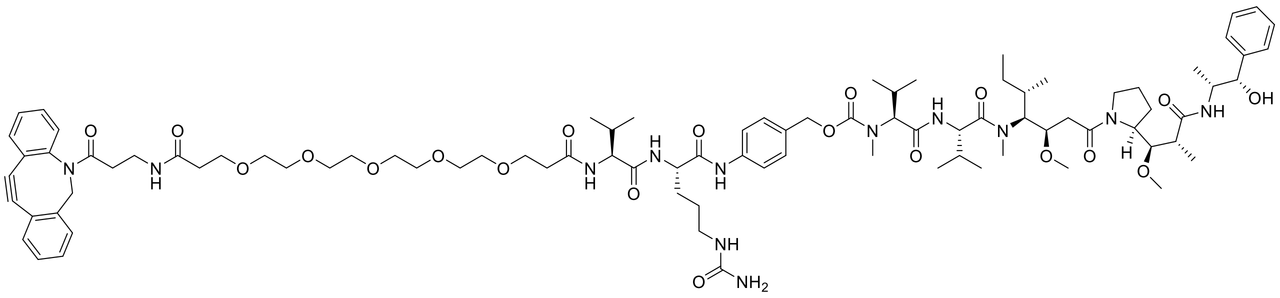

A solution of Fmoc-Val-Cit-PAB-MMAE **S21** (8 mg, 5.94 µmol) and piperidine (2.9 µL, 29.7 µmol) in DMF (0.5 mL) was stirred at rt for 2 hours. Upon completion, the mixture was concentrated under a stream of N_2 to give crude H-Val-Cit-PAB-MMAE which was carried through without further purification.

A solution of H-Val-Cit-PAB-MMAE, DBCO-PEG$_5$-COOH (4.3 mg, 7.13 µmol), HBTU (4.5 mg, 11.9 µmol) and DIPEA (3.1 µL, 17.8 µmol) in DMF (0.5 mL) was stirred at rt for 20 hours. Upon completion, the reaction was concentrated under a stream of N_2 and the crude residue purified by reverse-phase column chromatography (10–60% MeCN in 0.5% HCOOH in H_2O) to yield DBCO-PEG$_5$-Val-Cit-PAB-MMAE, **5** (0.8 mg, 0.47 µmol, 8%), as a white solid.

R*t*: 12.038 min (5–95% solvent B in solvent A); **HRMS** (ESI) $C_{90}H_{132}N_{12}O_{20}$ m/z: $[M+2H]^+$ 851.4919 (calc. 851.4993).

5 Trastuzumab bioconjugation

5.1 General procedure for trastuzumab bioconjugation

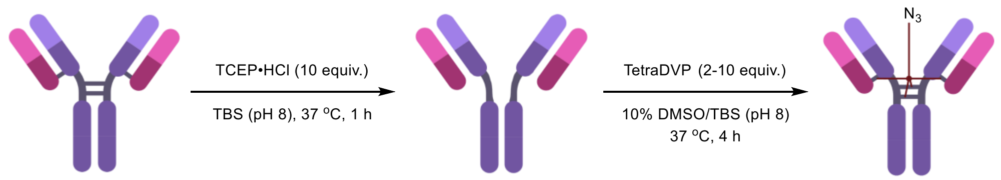

General procedure A:

To a solution of trastuzumab[a] in TBS buffer (1×, pH 8, [Tras] = 2.5 $mgml^{-1}$, 200 μL, 3.4 nmol) was added TCEP·HCl (5 mM stock in TBS, 6.8 μL, 34 nmol, 10 equiv.) and the mixture was incubated at 37 °C for 1 hour on a thermal shaker at 400 rpm. DMSO was added to ensure a final organic solvent concentration of 10–15%. TetraDVP (1–10 mM stock in DMSO, 2–10 equiv.) was added and the mixture incubated at 37 °C for 4 hours on a thermal shaker at 400 rpm. The conjugate was purified by Zeba™ Spin Desalting Column (MW cut-off 40,000 Da, Thermo Fisher Scientific) which had been equilibrated into PBS (3 × 300 μL). Organic solvent was reduced to <0.01% by PBS diafiltration using an Amicon-Ultra centrifugal filter (MW cut-off 10,000 Da, Merck Millipore).

[a]Lyophilised Herceptin was used, therefore additives such as L-Histidine were also present in the mixture.

5.2 Optimisation of bioconjugation using TetraDVP 1a

Initial screening of TetraDVP equivalents for the disulfide rebridging of reduced trastuzumab was conducted with TetraDVP **1a**.

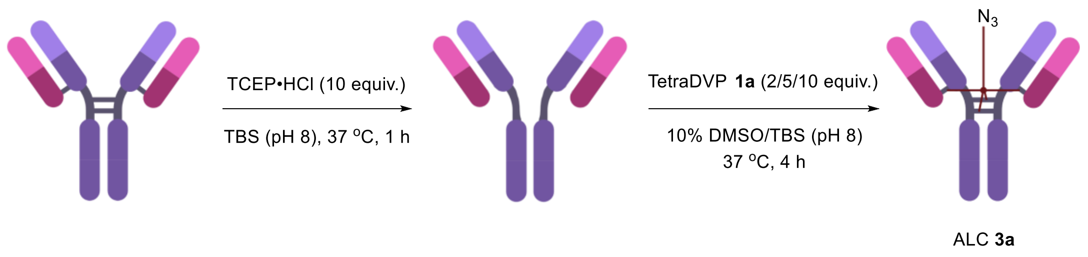

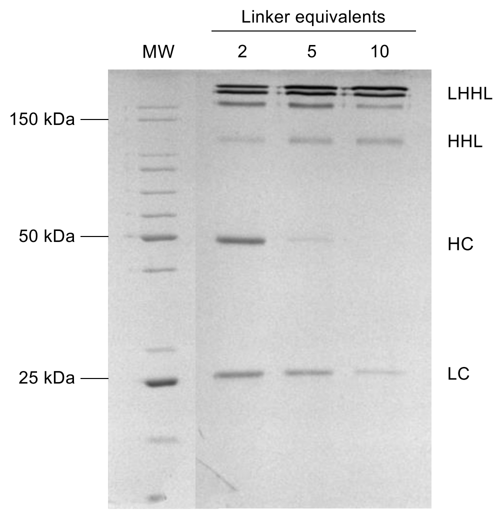

Figure S4: Initial screening of TetraDVP equivalents for the simultaneous rebridging of the disulfides in the IgG1 antibody trastuzumab. TetraDVP **1a** was used. Proteins were stained with Coomassie Brilliant Blue before imaging. 'HC' = un-rebridged Heavy Chain, 'LC' = un-rebridged Light Chain; 'LHHL' = Light-Heavy-Heavy-Light (full antibody), and indicates the fully rebridged species, containing TetraDVP. 'HHL' = Heavy-Heavy-Light, and indicates a partially rebridged species, containing TetraDVP.

ALC 3a

Bioconjugation was carried out using 10 equivalents of TetraDVP **1a** according to General Procedure A. SDS-PAGE (12% polyacrylamide gel, run at 200V over 50 minutes, visualised with Coomassie Brilliant Blue stain) indicated that the fully re-bridged antibody was the major product.

HRMS (ESI) $[M+H]^+$ 146,687 Da (calc. 146,679 Da).

ALC 3b

Bioconjugation was carried out using 10 equivalents of TetraDVP **1b** according to General Procedure A. SDS-PAGE (12% polyacrylamide gel, run at 200V over 50 minutes, visualised with Coomassie Brilliant Blue stain) indicated that the fully re-bridged antibody was the major product.

HRMS (ESI) $[M+H]^+$ 146,979 Da (calc. 146,969 Da).

ALC 3c

Bioconjugation was carried out using 10 equivalents of TetraDVP **1c** according to General Procedure A. SDS-PAGE (12% polyacrylamide gel, run at 200V over 50 minutes, visualised with Coomassie Brilliant Blue stain) indicated that the fully re-bridged antibody was the major product.

HRMS (ESI) $[M+H]^+$ 147,269 Da (calc. 147,259 Da).

ALC 3d

Bioconjugation was carried out using 10 equivalents of TetraDVP **1d** according to General Procedure A. SDS-PAGE (12% polyacrylamide gel, run at 200V over 50 minutes, visualised with Coomassie Brilliant Blue stain) indicated that the fully re-bridged antibody was the major product.

HRMS (ESI) $[M+H]^+$ 147,559 Da (calc. 147,549 Da).

ALC 4a

Bioconjugation was carried out using 10 equivalents of TetraDVP **2a** according to General Procedure A. SDS-PAGE (12% polyacrylamide gel, run at 200V over 50 minutes, visualised with Coomassie Brilliant Blue stain) indicated that the fully re-bridged antibody was the major product.

HRMS (ESI) $[M+H]^+$ 146,817 Da (calc. 146,808 Da).

ALC 4b

Bioconjugation was carried out using 10 equivalents of TetraDVP **2b** according to General Procedure A. SDS-PAGE (12% polyacrylamide gel, run at 200V over 50 minutes, visualised with Coomassie Brilliant Blue stain) indicated that the fully re-bridged antibody was the major product.

HRMS (ESI) $[M+H]^+$ 147,109 Da (calc. 147,098 Da).

ALC 4c

Bioconjugation was carried out using 10 equivalents of TetraDVP **2c** according to General Procedure A. SDS-PAGE (12% polyacrylamide gel, run at 200V over 50 minutes, visualised with Coomassie Brilliant Blue stain) indicated that the fully re-bridged antibody was the major product.

HRMS (ESI) $[M+H]^+$ 147,397 Da (calc. 147,388 Da).

ALC 4d

Bioconjugation was carried out using 10 equivalents of TetraDVP **2d** according to General Procedure A. SDS-PAGE (12% polyacrylamide gel, run at 200V over 50 minutes, visualised with Coomassie Brilliant Blue stain) indicated that the fully re-bridged antibody was the major product.

HRMS (ESI) $[M+H]^+$ 147,687 Da (calc. 147,678 Da).

5.3 Additional SDS-PAGE analysis including controls

SDS-PAGE analysis of the reaction between trastuzumab and TetraDVP **2c** under General Procedure A and related controls.

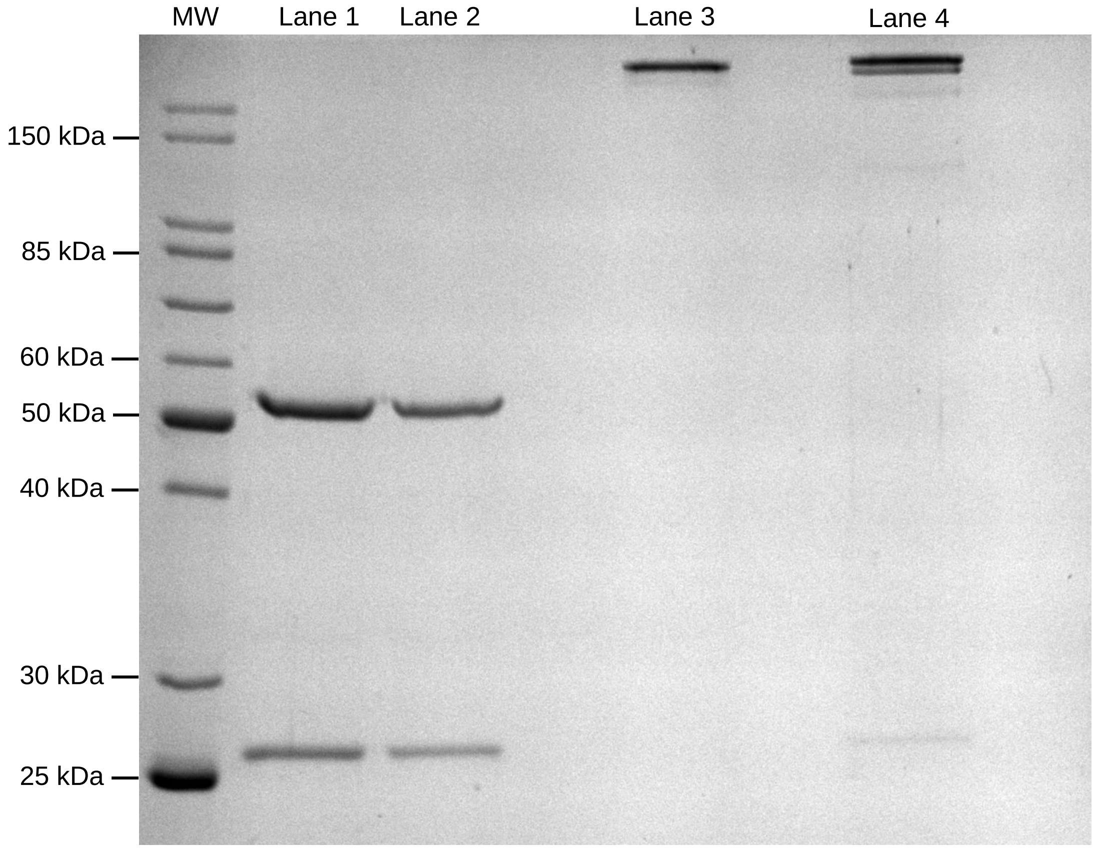

Figure S5: SDS-PAGE analysis of the reaction between trastuzumab and TetraDVP **2c**. Lane 1: Reaction without initial incubation with TCEP (no re-bridging observed); Lane 2: Trastuzumab; Lane 3: Trastuzumab (non-reducing dye, ‘intact’); Lane 4: ALC **4c**. MW = molecular weight ladder.

6 ADC synthesis

6.1 General procedure for click chemistry

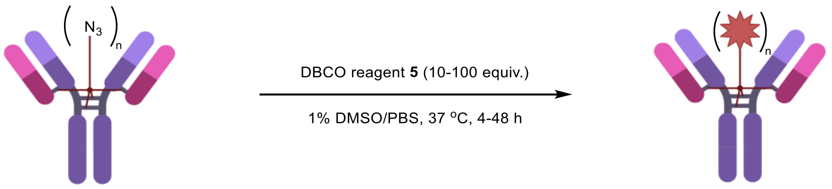

General Procedure B:

To a solution of trastuzumab-linker conjugate in PBS ([Tras] = 1.0 mgml^{-1}, 60–100 μL, 0.40–0.68 nmol) was added a solution of DBCO click reagent **5** in DMSO (10 mM stock concentration, 0.40–0.68 μL, 4.0–6.8 nmol, 10 equiv.) and the mixture was incubated at 37 °C for 4–48 hours on a thermal shaker at 400 rpm. The conjugate was purified by Zeba™ Spin Desalting Column (MW cut-off 40,000 Da, Thermo Fisher Scientific) which had been equilibrated into PBS (3 × 300 μL). Organic solvent was reduced to <0.01% by PBS diafiltration using an Amicon-Ultra centrifugal filter (MW cut-off 10,000 Da, Merck Millipore).

Optimisation reactions for the synthesis of ADCs

Table S1: Optimisation of the SPAAC reaction between ALCs **3a–d** and **4a–d** DBCO reagent **5**. Conditions in entries 3, 4 and 5 were not applied to ALCs **4a–d** due to insignificant improvements observed when applied to ALCs **3a–d**.

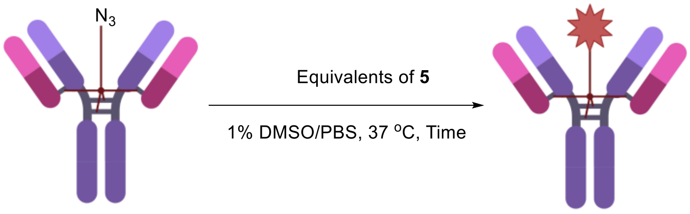

ALC	→ ADC
**3a**	**6a**
**3b**	**6b**
**3c**	**6c**
**3d**	**6d**
**4a**	**7a**
**4b**	**7b**
**4c**	**7c**
**4d**	**7d**

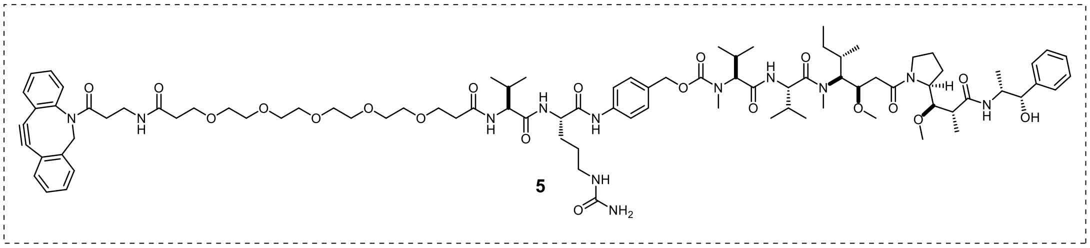

Entry	Equivalents of DBCO reagent **5**	Time /h	DAR **6a**	**6b**	**6c**	**6d**	**7a**	**7b**	**7c**	**7d**
1	10	4	0.74	0.69	0.81	0.83	0.75	0.74	0.71	0.75
2	10	24	0.82	0.76	0.84	0.89	0.87	0.90	0.84	0.87
3	100	24	0.65	0.61	0.69	0.74	-	-	-	-
4	10	48	0.79	0.74	0.82	0.88	-	-	-	-
5	2 × 10	2 × 24	0.80	-	0.82	-	-	-	-	-

ADC 6a

Click reaction was carried out via General Procedure B using ALC **3a** and the mixture was incubated for 24 hours to produce the desired ADC.

HRMS (ESI) $[M+H]^+$ 148,390 Da (calc. 148,380 Da).

ADC 6b

Click reaction was carried out via General Procedure B using ALC **3b** and the mixture was incubated for 24 hours to produce the desired ADC.

HRMS (ESI) $[M+H]^+$ 148,681 Da (calc. 148,670 Da).

ADC 6c

Click reaction was carried out via General Procedure B using ALC **3c** and the mixture was incubated for 24 hours to produce the desired ADC.

HRMS (ESI) $[M+H]^+$ 148,971 Da (calc. 148,960 Da).

ADC 6d

Click reaction was carried out via General Procedure B using ALC **3d** and the mixture was incubated for 24 hours to produce the desired ADC.

HRMS (ESI) $[M+H]^+$ 149,262 Da (calc. 149,250 Da).

ADC 7a

Click reaction was carried out via General Procedure B using ALC **4a** and the mixture was incubated for 24 hours to produce the desired ADC.

HRMS (ESI) $[M+H]^+$ 148,518 Da (calc. 148,509 Da).

ADC 7b

Click reaction was carried out via General Procedure B using ALC **4b** and the mixture was incubated for 24 hours to produce the desired ADC.

HRMS (ESI) $[M+H]^+$ 148,808 Da (calc. 148,799 Da).

ADC 7c

Click reaction was carried out via General Procedure B using ALC **4c** and the mixture was incubated for 24 hours to produce the desired ADC.

HRMS (ESI) $[M+H]^+$ 149,098 Da (calc. 149,089 Da).

ADC 7d

Click reaction was carried out via General Procedure B using ALC **4d** and the mixture was incubated for 24 hours to produce the desired ADC.

HRMS (ESI) $[M+H]^+$ 149,389 Da (calc. 149,379 Da).

General Procedure C:

To a solution of trastuzumab-linker conjugate in PBS ([Tras] = 1.8 $mgml^{-1}$) was added a solution of DBCO click reagent **9** in DMA (10 equiv.) and further DMA added to make up the organic solvent concentration to 10% v/v. The mixture was incubated at room temperature for 24 hours to produce the desired ADC.

ADC 9a

Click reaction was carried out via General Procedure C using ALC **4a**, to provide the desired ADC with a DAR of 0.84 as determined by HIC.

HRMS (ESI) $[M+H]^+$ 151,506 Da.

ADC 9b

Click reaction was carried out via General Procedure C using ALC **4b**, to provide the desired ADC with a DAR of 0.90 as determined by HIC.

HRMS (ESI) $[M+H]^+$ 151,796 Da.

ADC 9c

Click reaction was carried out via General Procedure C using ALC **4c**, to provide the desired ADC with a DAR of 0.94 as determined by HIC.

HRMS (ESI) $[M+H]^+$ 152,087 Da.

ADC 9d

Click reaction was carried out via General Procedure C using ALC **4d**, to provide the desired ADC with a DAR of 0.89 as determined by HIC.

HRMS (ESI) $[M+H]^+$ 152,377 Da.

.

7 Synthesis of PEG_4-azide reagents

During this work, a PEG_4-azide reagent was required to install the pendant azide on the TetraDVP backbones of series **2a–d**. Whilst the desired reagents were commercially available, their cost prohibited large scale synthesis. An alternative route to the required reagents is provided below.

HO – PEG_4 – $CH_2CH_2CO_2$tBu (S22)

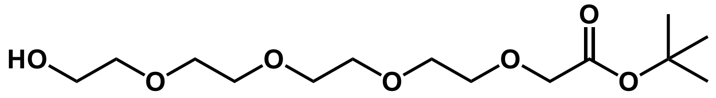

In each of four separate reaction vessels, tetraethylene glycol (10.4 mL, 60 mmol), *tert*-butyl acrylate (8.73 mL, 60 mmol) and sodium hydroxide (240 mg, 6 mmol) were combined. Each vessel was submitted to microwave irradiation, with stirring, at 70 °C for 15 minutes. The crude reaction mixtures were combined, diluted with CH_2Cl_2 and concentrated *in vacuo*. The crude mixture was submitted to flash column chromatography (70–100% EtOAc in pet. ether) to yield the product, **S22** (35.8 g, 111 mmol, 46%), as a colourless oil.

R*f*: 0.11 (EtOAc); ^{1}**H NMR** (400 MHz, $CDCl_3$, 25 °C): δ (ppm) = 3.75–3.57 (m, 18H), 2.67 (br s, 1H), 2.49 (t, J = 6.2 Hz, 2H), 1.44 (s, 9H); ^{13}**C NMR** (100 MHz, $CDCl_3$): δ (ppm) = 171.0, 80.6, 72.6, 70.8, 70.7, 70.7, 70.6, 70.5, 70.5, 67.0, 61.9, 36.4, 28.2; **LRMS** (ESI) $C_{15}H_{30}O_7$ m/z: $[M+Na]^+$ 345.2 (calc. 345.2).

Data agree with those reported in the literature.[6]

TsO – PEG_4 – $CH_2CH_2CO_2$tBu (S23)

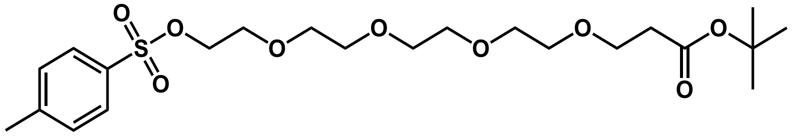

Alcohol **S22** (500 mg, 1.55 mmol) was dissolved in THF (5 mL) and sodium hydroxide (68 mg, 1.71 mmol) in H_2O (1 mL) was added. The solution was cooled to 0 °C and a solution of tosyl chloride (355 mg, 1.86 mmol) in THF (3 mL) was added dropwise. The mixture was allowed to warm to room temperature and stirred for 4 hours. The mixture was concentrated *in vacuo*, diluted with H_2O (10 mL) and extracted with CH_2Cl_2 (3 × 20 mL). The combined organic extracts were washed with brine (30 mL), dried ($MgSO_4$), filtered and concentrated *in vacuo*. The crude product was subjected to flash column chromatography (40–70% EtOAc in pet. ether) to yield the product, **S23** (316 mg, 0.66 mmol, 43%), as a colourless oil.

R*f*: 0.28 (70% EtOAc in pet. ether); ^{1}**H NMR** (400 MHz, $CDCl_3$, 25 °C): δ (ppm) = 7.79 (d, J = 7.6 Hz, 2H), 7.33 (d, J = 7.7 Hz, 2H), 4.15 (app s, 2H), 3.71–3.67 (m, 4H), 3.61 (app s, 8H), 3.57 (app s, 4H), 2.49 (t, J = 6.4 Hz, 2H), 2.44 (s, 3H), 1.44 (s, 9H); ^{13}**C NMR** (100 MHz, $CDCl_3$): δ (ppm) = 171.0, 144.9, 133.2, 129.9, 128.1, 80.6, 70.9, 70.8, 70.7, 70.7, 70.6, 70.5, 69.4, 68.8, 67.0, 36.4, 28.2, 21.8; **LRMS** (ESI) $C_{22}H_{36}O_9S$ m/z: $[M+Na]^+$ 499.3 (calc. 499.2).

Data agree with those reported in the literature.[7]

N_3 – PEG$_4$ – $CH_2CH_2CO_2$tBu (S24)

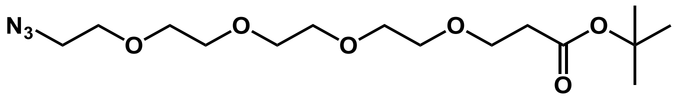

Tosylate **S23** (268 mg, 0.56 mmol) was dissolved in DMF (1 mL) and sodium azide (55 mg, 0.84 mmol) was added. The reaction mixture was heated to 50 °C for 21 hours. The mixture was diluted with H_2O (10 mL) and extracted with CH_2Cl_2 (5 × 10 mL). The combined organic extracts were washed with brine (40 mL), dried ($MgSO_4$), filtered and concentrated *in vacuo*. The crude mixture was submitted to flash column chromatography (50–90% EtOAc in pet. ether) to yield the product, **S24** (153.8 mg, 0.44 mmol, 79%) as a colourless oil.

R*f*: 0.53 (EtOAc); ^{1}**H NMR** (400 MHz, $CDCl_3$, 25 °C): δ (ppm) = 3.70 (t, *J* = 6.9 Hz, 2H), 3.65 (app s, 10H), 3.61 (app s, 4H), 3.38 (app s, 2H), 2.49 (t, *J* = 6.3 Hz, 2H), 1.44 (s, 9H); ^{13}**C NMR** (100 MHz, $CDCl_3$): δ (ppm) = 171.0, 80.6, 70.8, 70.8, 70.8, 70.7, 70.6, 70.5, 70.2, 67.0, 50.8, 36.4, 28.2; **HRMS** (ESI) $C_{15}H_{29}N_3O_6$ m/z: $[M+Na]^+$ 370.1945 (calc. 370.1948).

Data agree with those reported in the literature.[6]

N_3 – PEG$_4$ – $CH_2CH_2CO_2H$ (S25)

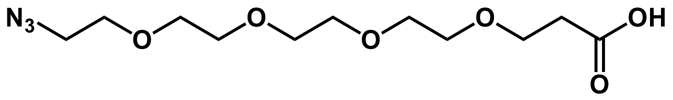

Ester **S24** (200 mg, 0.58 mmol) was dissolved in CH_2Cl_2 (3 mL). A solution of HCl (4 M in dioxane, 2 mL, 8.6 mmol) was added, and the reaction mixture stirred for 20 hours. The mixture was concentrated to yield the product, **S25** (162.3 mg, 0.56 mmol, 96%) as an orange oil.

R*f*: Baseline (EtOAc); ^{1}**H NMR** (400 MHz, $CDCl_3$, 25 °C): δ (ppm) = 6.20 (br s, 1H), 3.77 (t, *J* = 5.9 Hz, 2H), 3.70–3.64 (m, 14H), 3.39 (app d, *J* = 8.6 Hz, 2H), 2.63 (t, *J* = 6.0 Hz, 2H); ^{13}**C NMR** (100 MHz, $CDCl_3$): δ (ppm) = 175.3, 70.8, 70.8, 70.7, 70.6, 70.6, 70.4, 70.1, 66.5, 50.8, 35.0; **HRMS** (ESI) $C_{11}H_{21}N_3O_6$ m/z: $[M+Na]^+$ 314.1310 (calc. 314.1322).

Data agree with those reported in the literature.[6]

N_3 – PEG_4 – $CH_2CH_2CO_2Su$ (S26)

In order to access the corresponding NHS-ester of acid **S25**, an EDC-mediated amide coupling could be used. Whilst this reagent was not used for the synthesis of TetraDVP linkers reported here, it is included for those who may want to make use of the reagent.

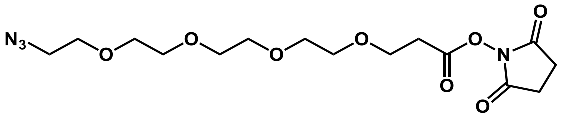

Acid **S25** (506 mg, 1.74 mmol) and *N*-hydroxysuccinimide (296 mg, 2.57 mmol) were dissolved in CH_2Cl_2 (6 mL). EDC·HCl (658 mg, 3.43 mmol) was added, and the reaction mixture stirred for 45 hours. The mixture was washed with sat. aq. $NaHSO_4$ (15 mL) and brine (15 mL), dried ($MgSO_4$), filtered and concentrated *in vacuo* to yield the product, **S26** (525.1 mg, 1.35 mmol, 79%) as a pale yellow oil.

$\mathbf{R}_f$: 0.45 (EtOAc); ^{1}**H NMR** (400 MHz, $CDCl_3$, 25 °C): δ (ppm) = 3.83 (t, J = 6.4 Hz, 2H), 3.68–3.64 (m, 14H), 3.37 (app s, 2H), 2.89 (t, J = 6.4 Hz, 2H), 2.82 (app s, 4H); ^{13}**C NMR** (100 MHz, $CDCl_3$): δ (ppm) = 169.1, 166.8, 70.8, 70.8, 70.8, 70.8, 70.7, 70.6, 70.1, 65.8, 50.8, 32.3, 25.7; **HRMS** (ESI) $C_{15}H_{24}N_4O_8$ m/z: $[M+Na]^+$ 411.1489 (calc. 411.1486).

References

[1] Dannheim, F. M.; Walsh, S. J.; Orozco, C. T.; Hansen, A. H.; Bargh, J. D.; Jackson, S. E.; Bond, N. J.; Parker, J. S.; Carroll, J. S.; Spring, D. R. *Chemical Science* **2022**, *13*, 8781–8790.

[2] Ndinguri, M. W.; Fronczek, F. R.; Marzilli, P. A.; Crowe, W. E.; Hammer, R. P.; Marzilli, L. G. *Inorganica Chimica Acta* **2010**, *363*, 1796–1804.

[3] Tanada, M.; Tsujita, S.; Sasaki, S. *Journal of Organic Chemistry* **2005**, *71*, 125–134.

[4] Kalesse, M.; Loos, A. *Liebigs Annales* **1996**, 935–939.

[5] United States Patent Application Publication, US20100233190A1, 2010.

[6] Béquignat, J. B.; Ty, N.; Rondon, A.; Taiariol, L.; Degoul, F.; Canitrot, D.; Quintana, M.; Navarro-Teulon, I.; Miot-Noirault, E.; Boucheix, C.; Chezal, J. M.; Moreau, E. *European Journal of Medicinal Chemistry* **2020**, *203*, 112574.

[7] Miller, M. L.; Roller, E. E.; Zhao, R. Y.; Leece, B. A.; Ab, O.; Baloglu, E.; Goldmacher, V. S.; Chari, R. V. *Journal of Medicinal Chemistry* **2004**, *47*, 4802–4805.

8 NMR spectra

Ethyl 4-((4,6-dichloropyrimidin-2-yl)amino)butanoate (**S2**)

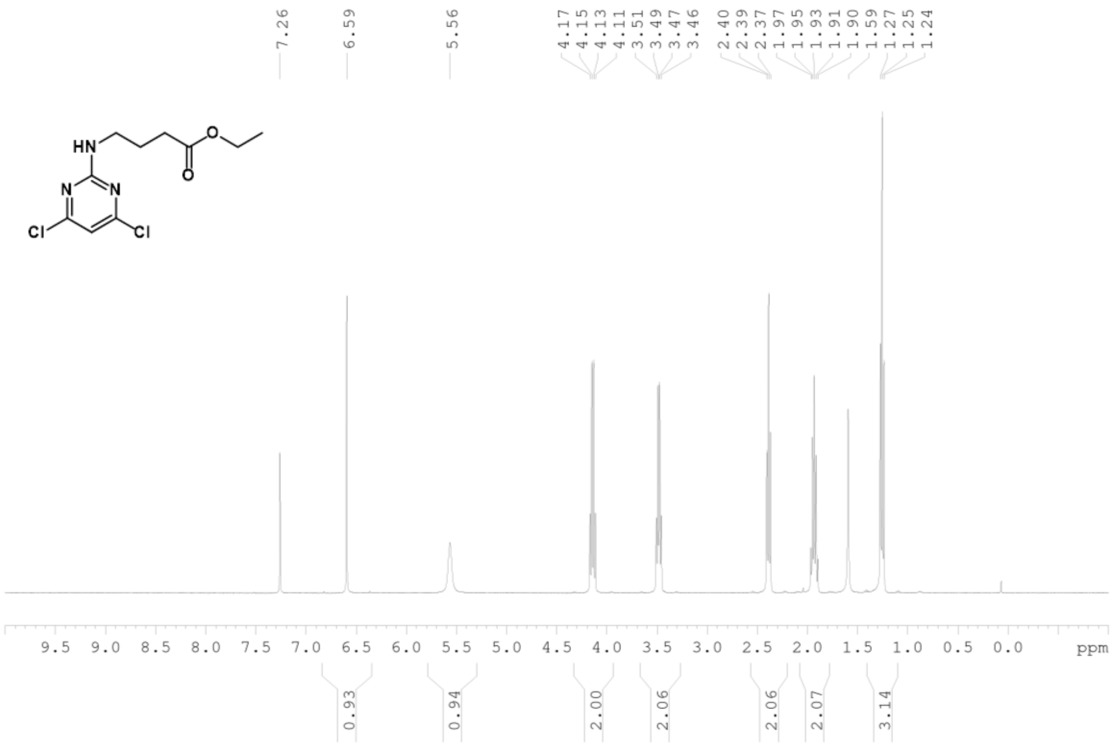

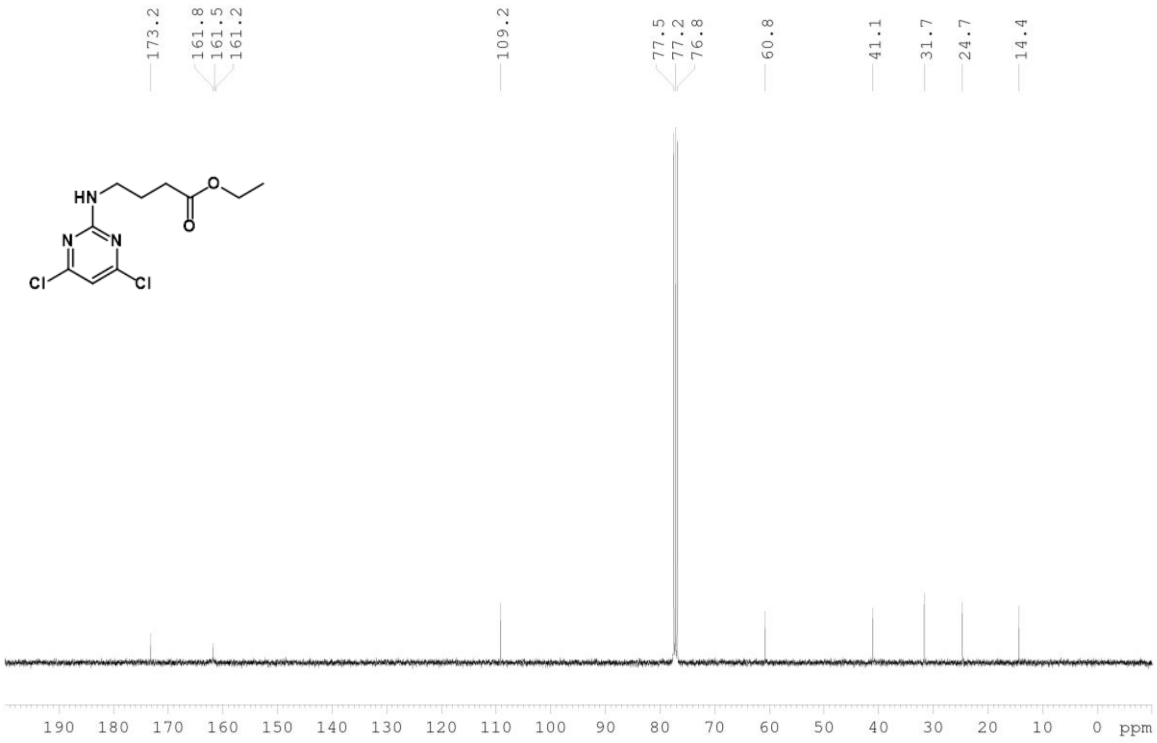

Ethyl 4-((4,6-divinylpyrimidin-2-yl)amino)butanoate (**S3**)

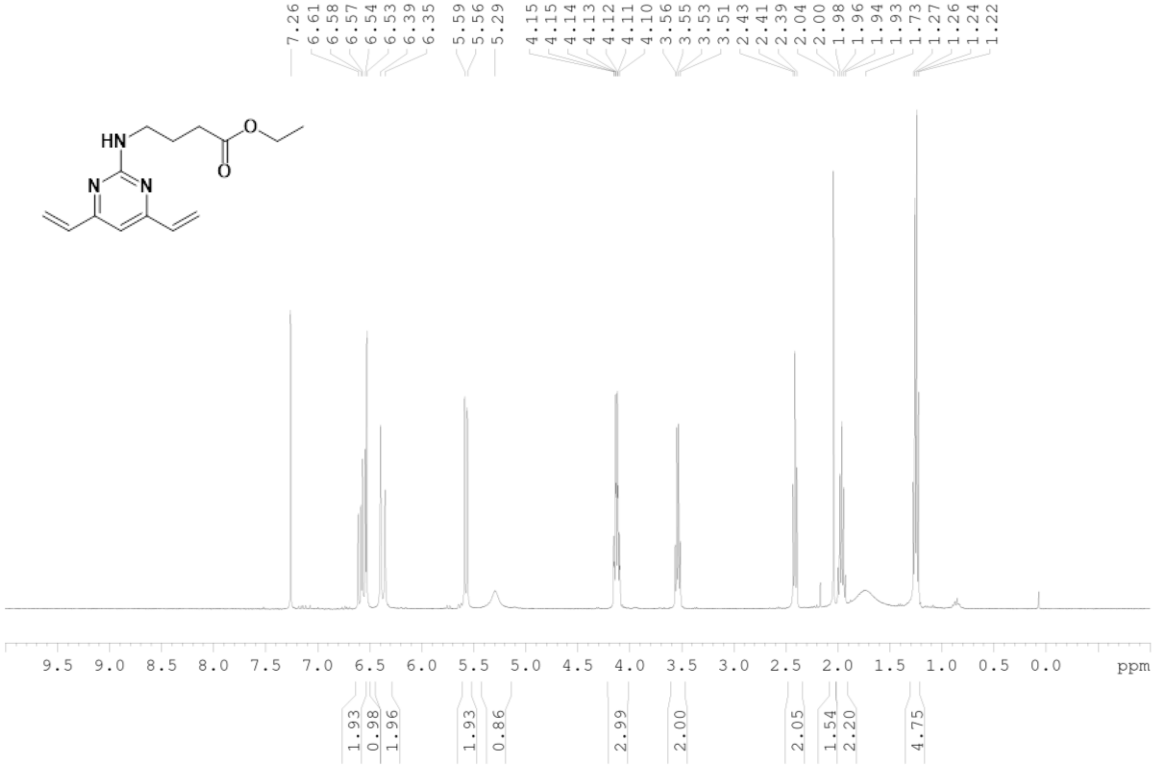

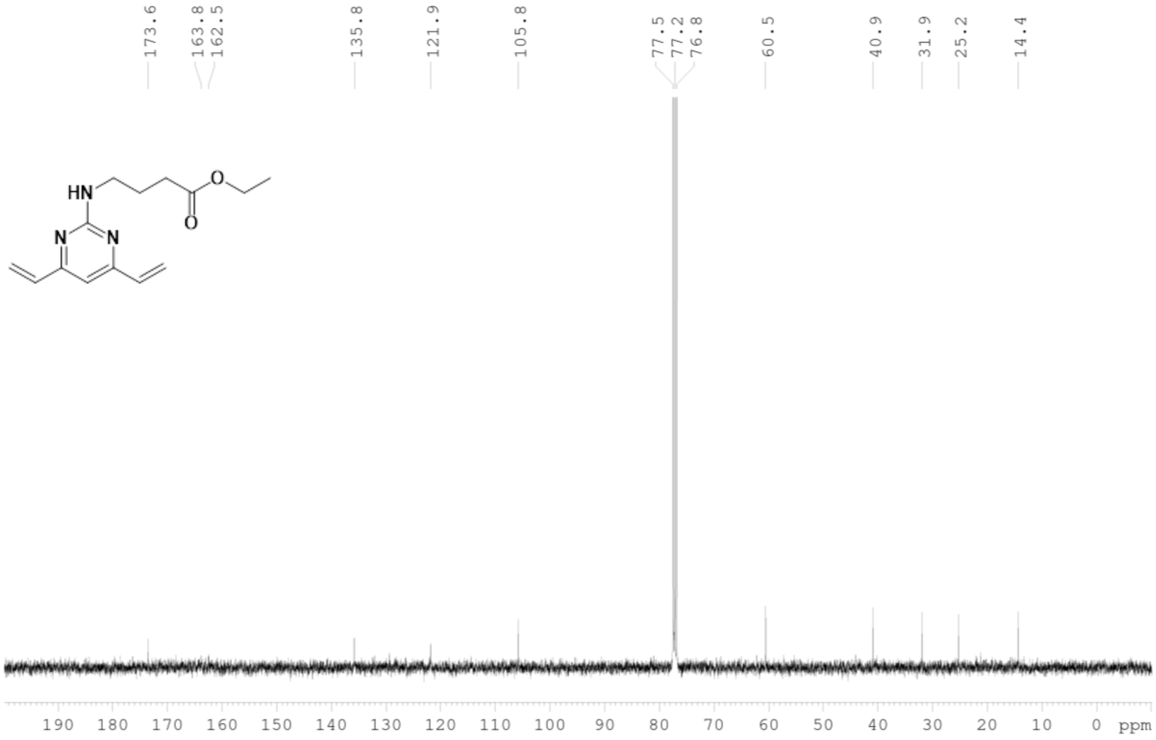

4-((4,6-Divinylpyrimidin-2-yl)amino)butanoic acid (**S1**)

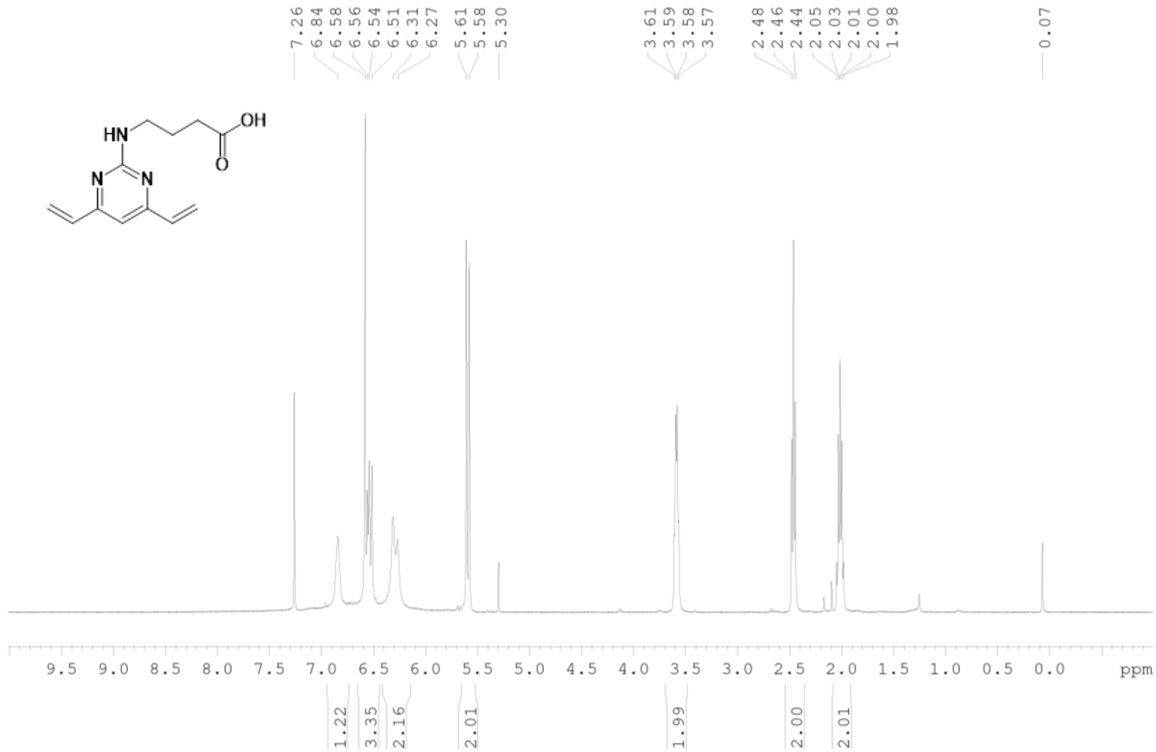

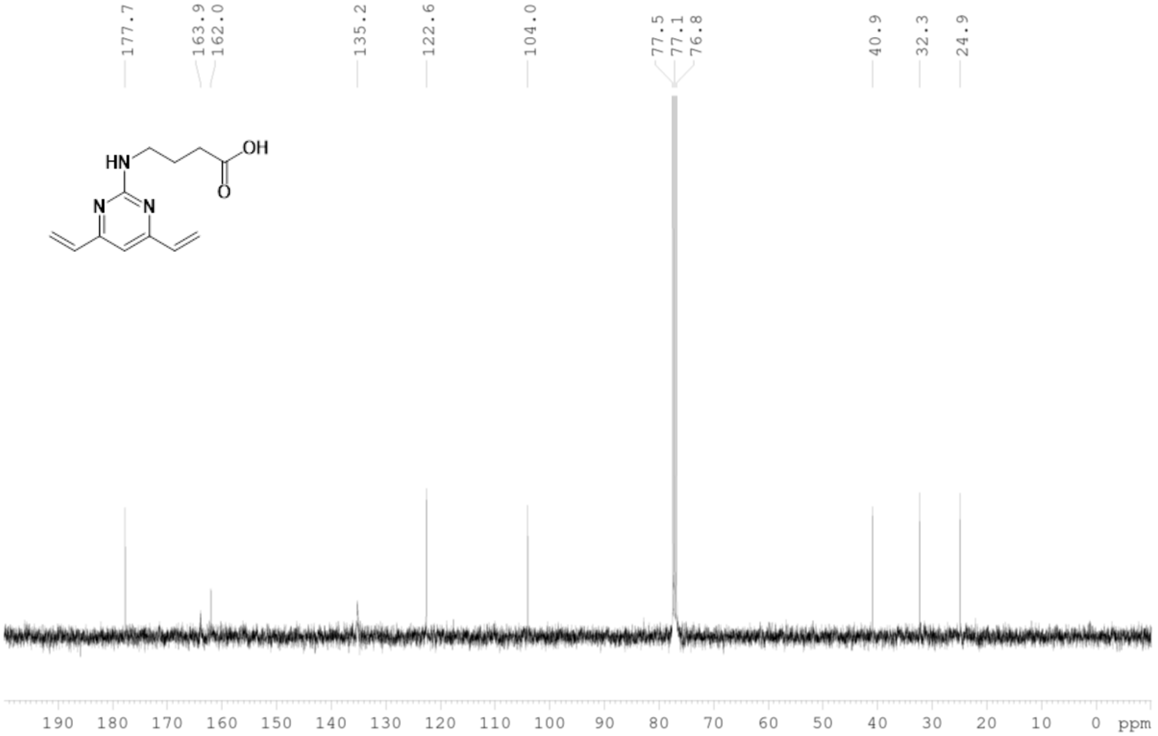

Di-*tert*-butyl (azanediylbis(ethane-2,1-diyl))dicarbamate (**S4**)

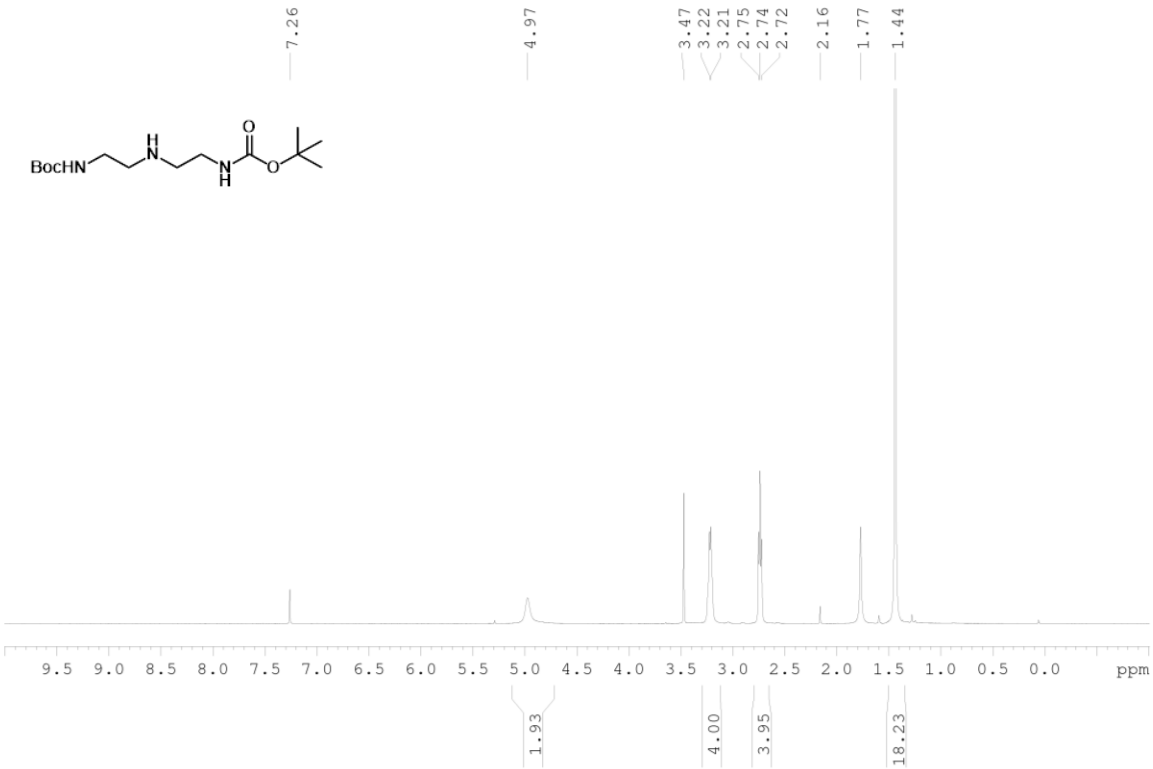

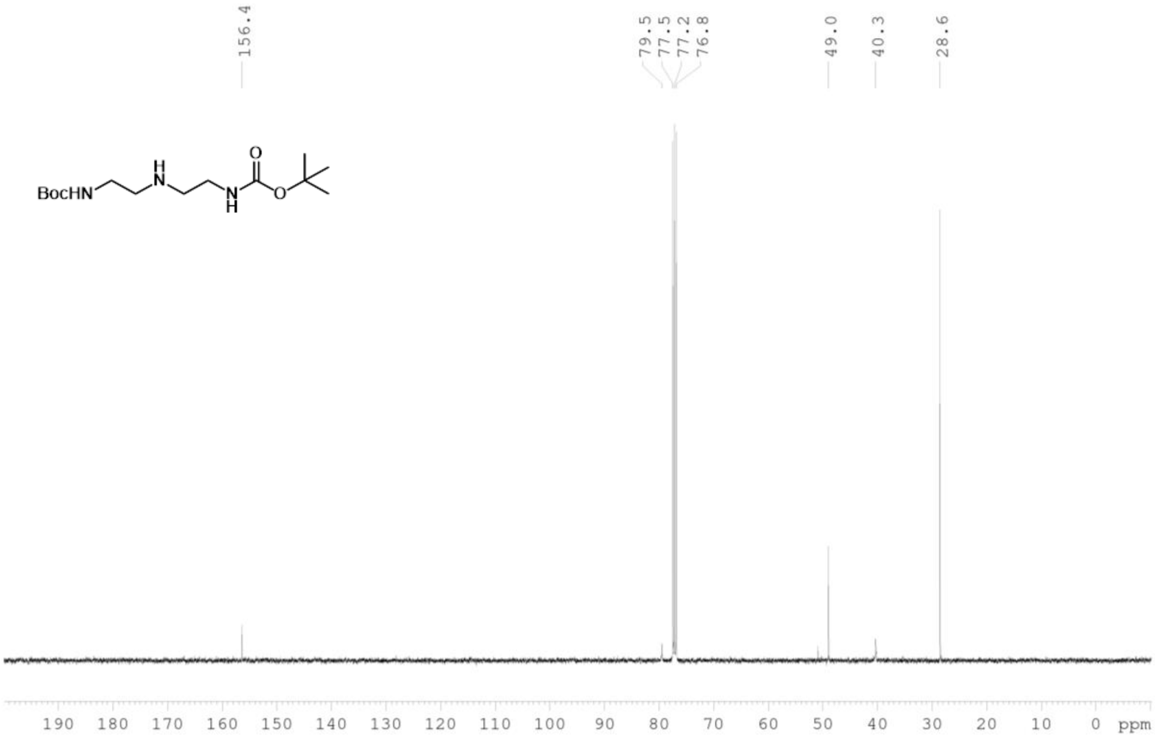

Benzyl bis(2-((*tert*-butoxycarbonyl)amino)ethyl)glycinate (**S5**)

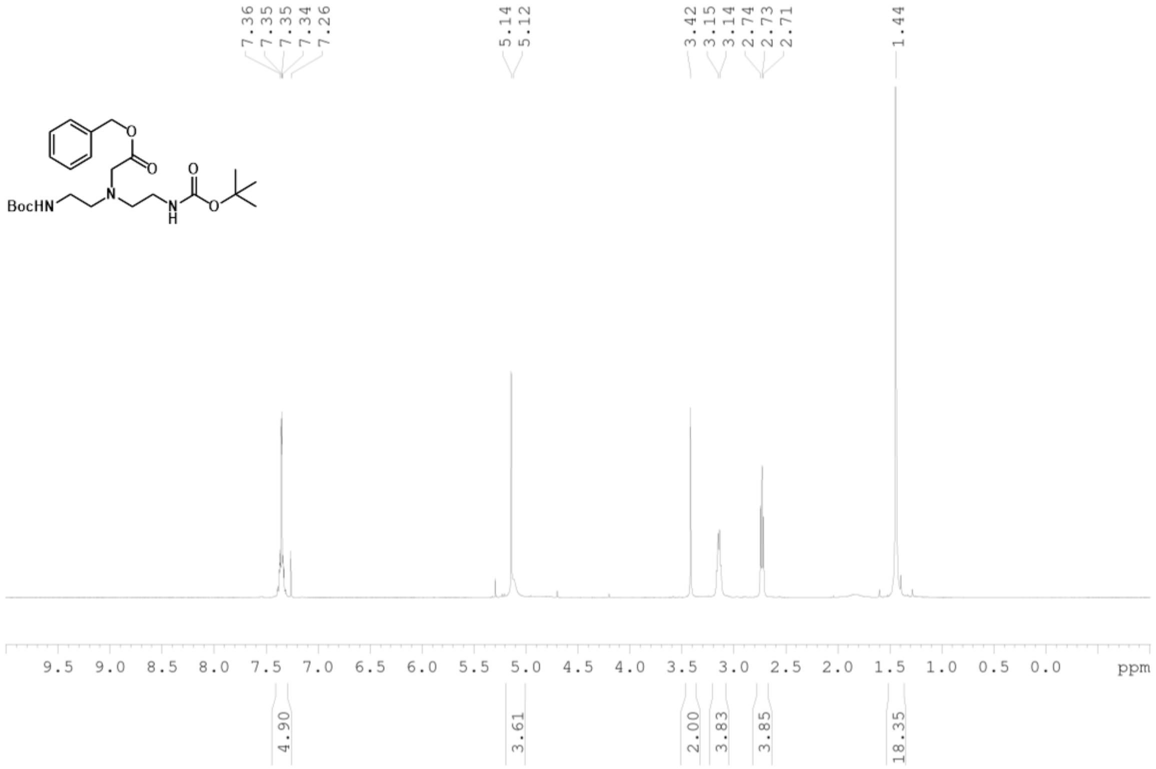

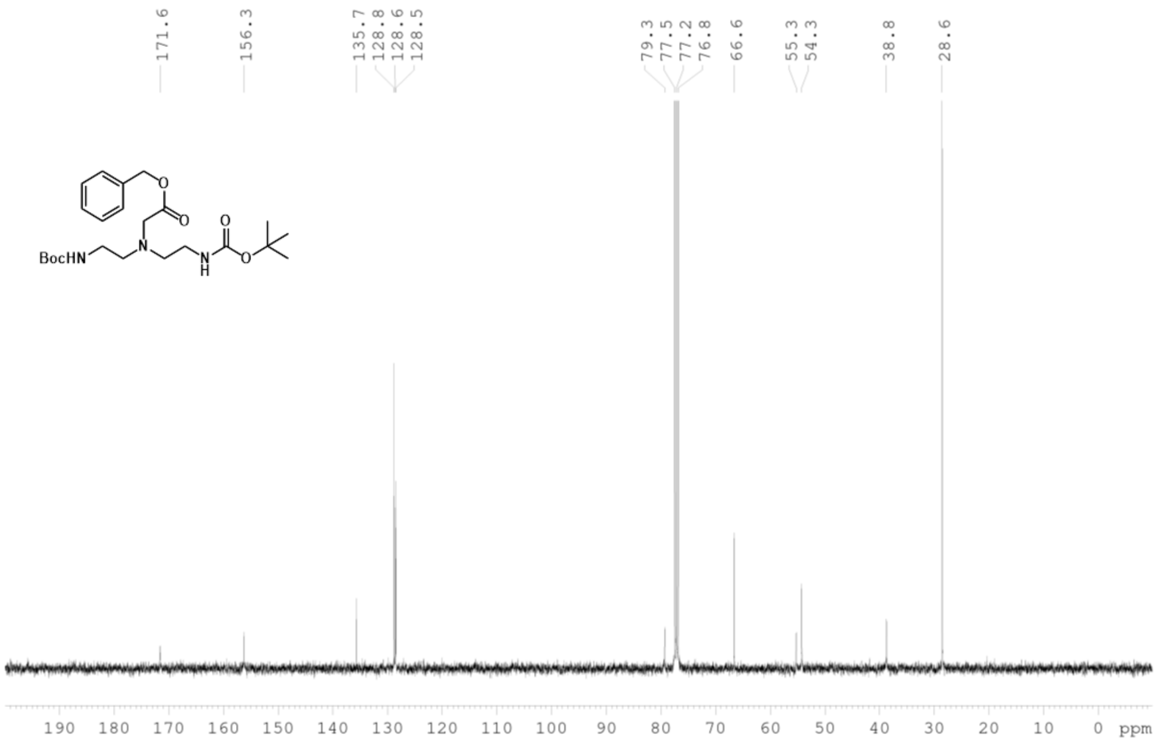

Bis(2-((*tert*-butoxycarbonyl)amino)ethyl)glycine (**S6**)

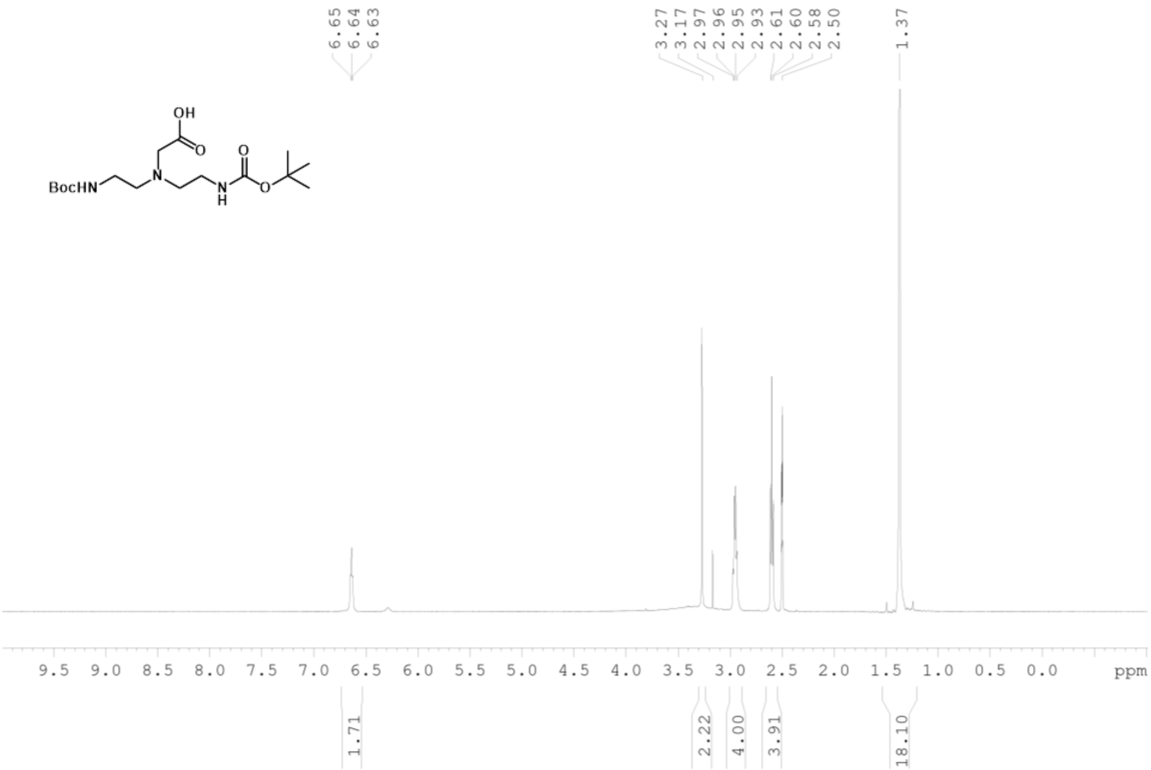

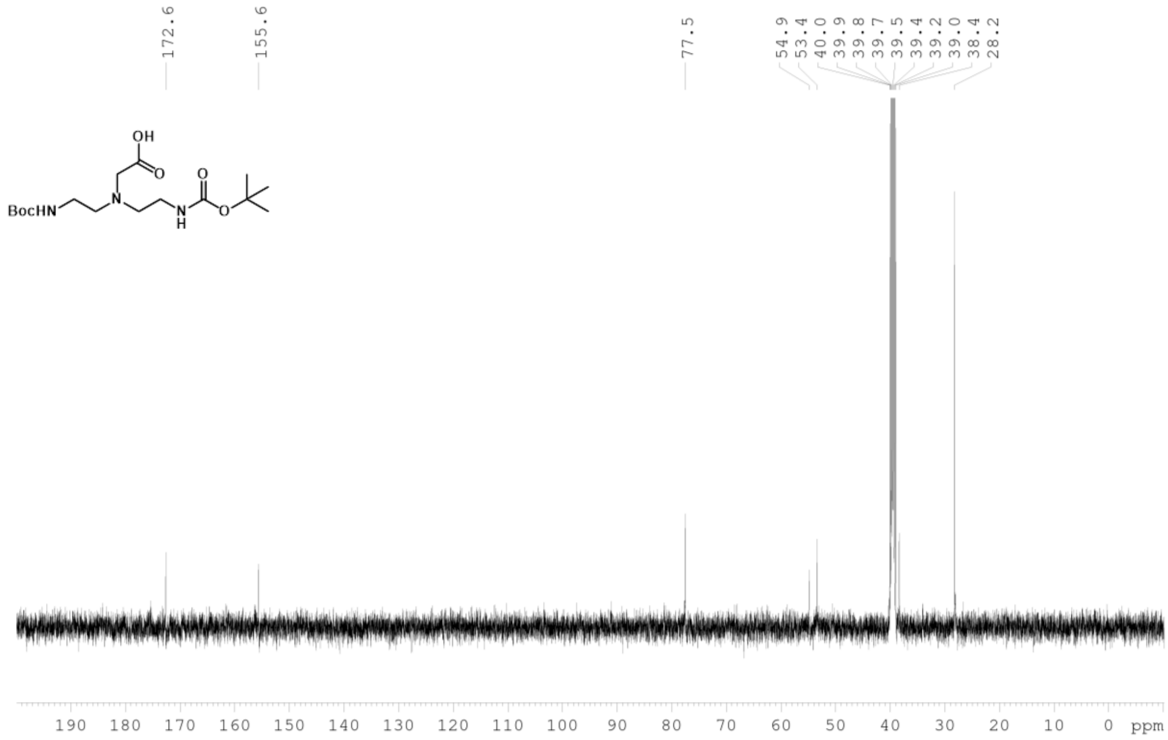

Bis-*N*-Boc-triamine with PEG_3 azide side chain (**S7**)

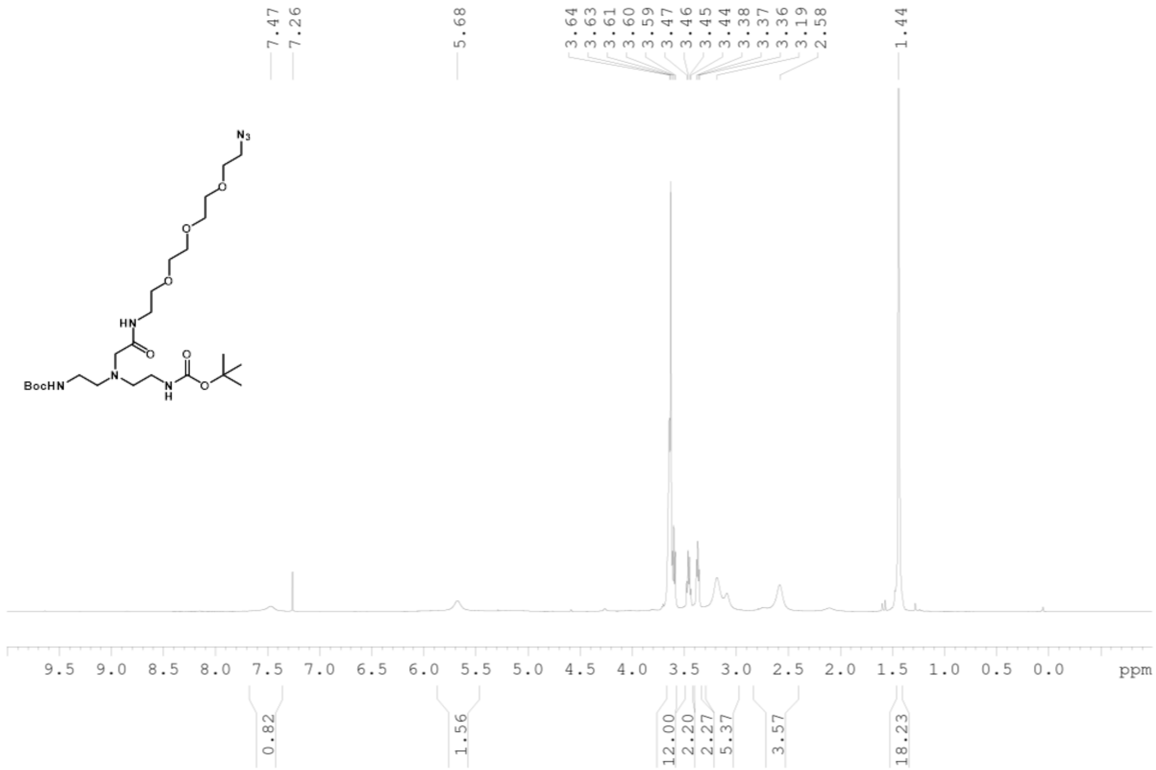

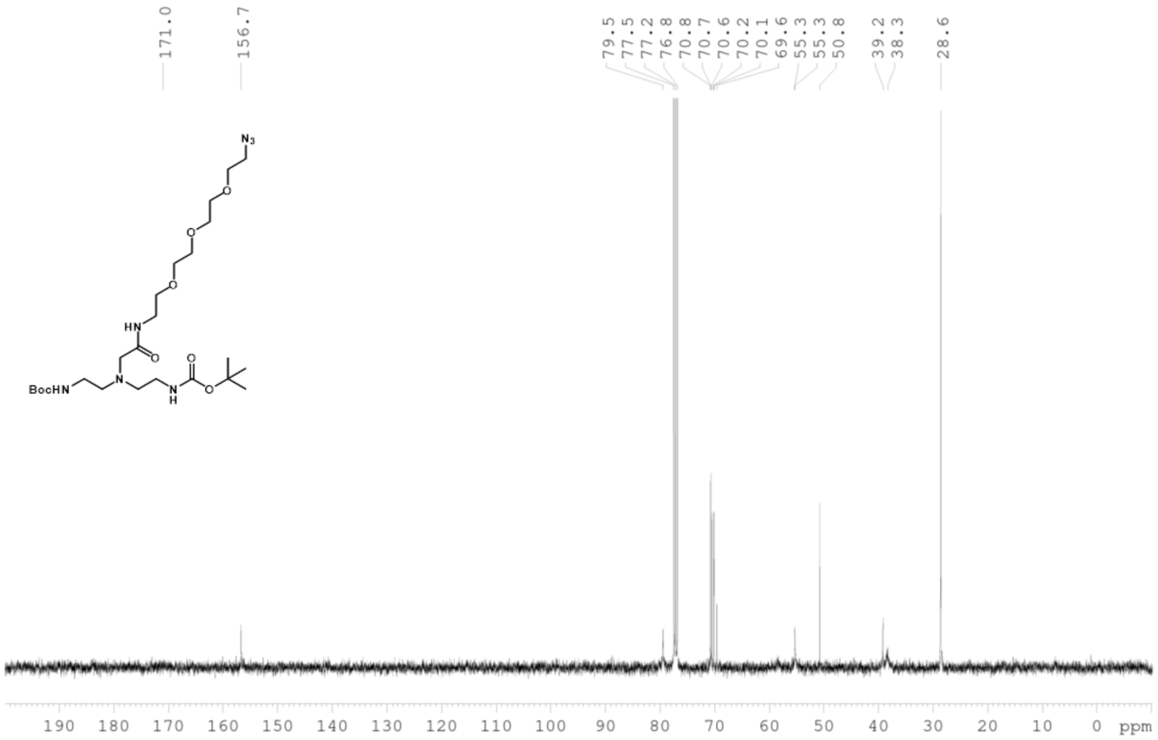

Tetra-*N*-Boc no-PEG backbone with PEG_3 azide side chain (**S8**)

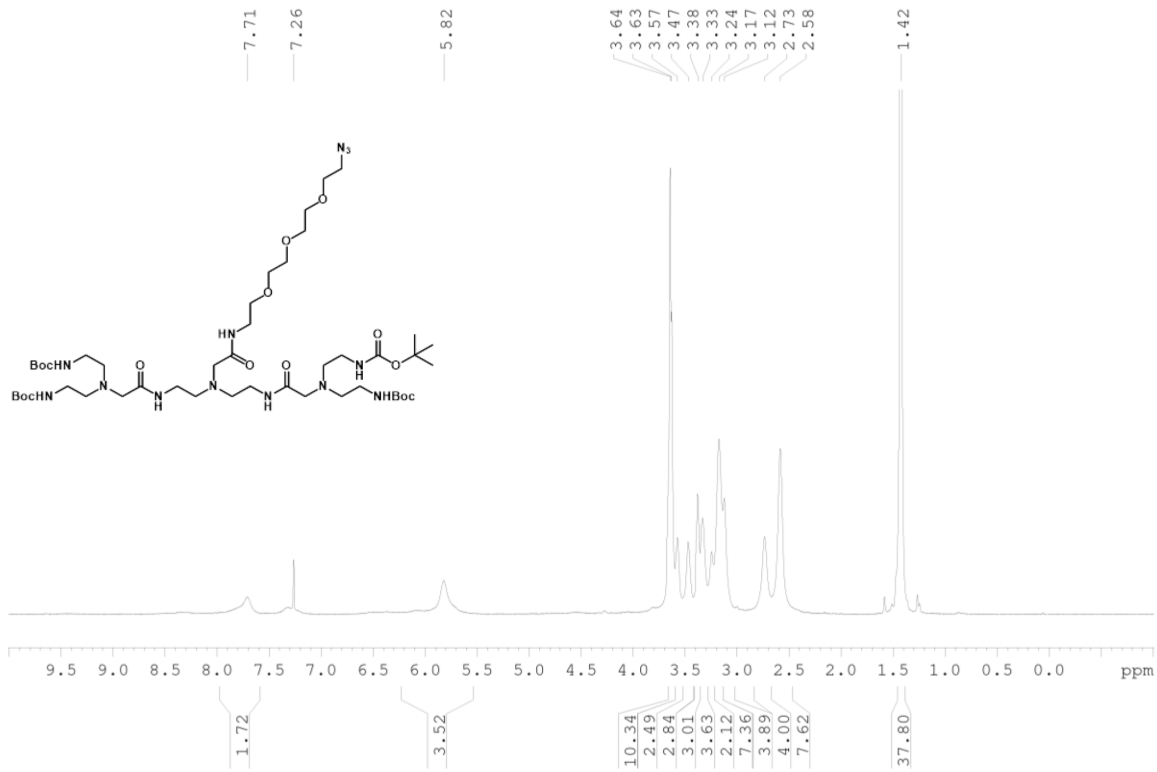

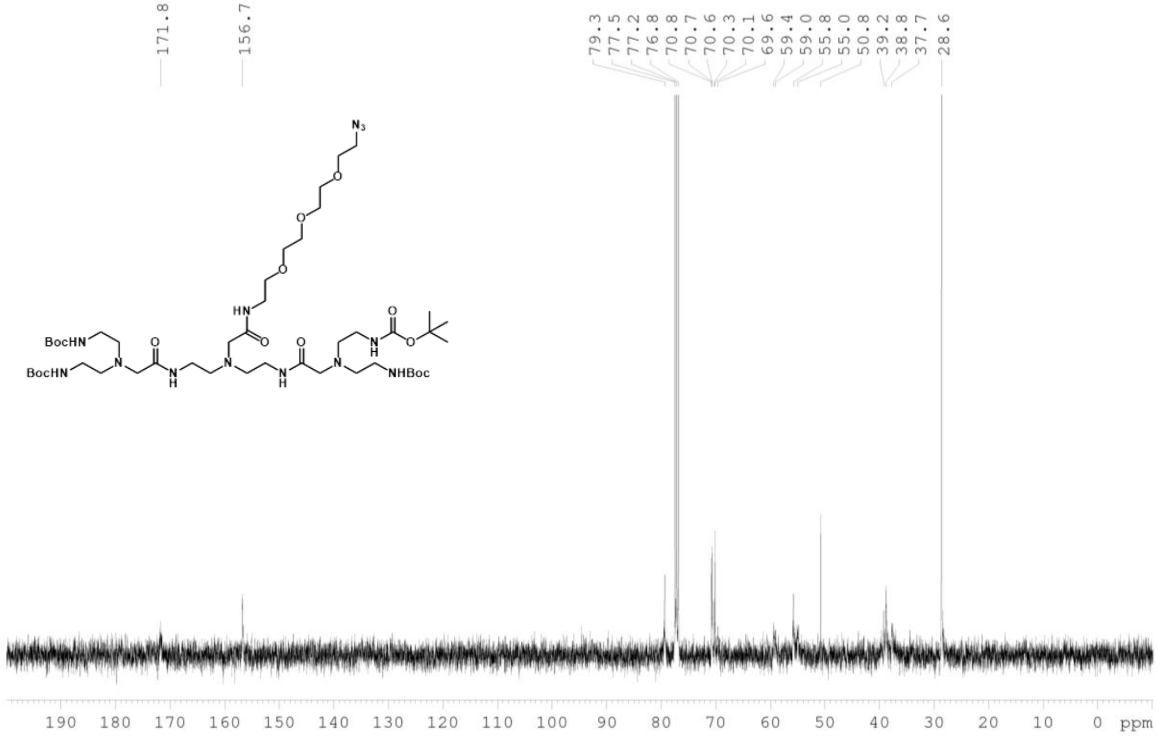

TetraDVP no-PEG backbone with PEG_3 azide side chain (**1a**)

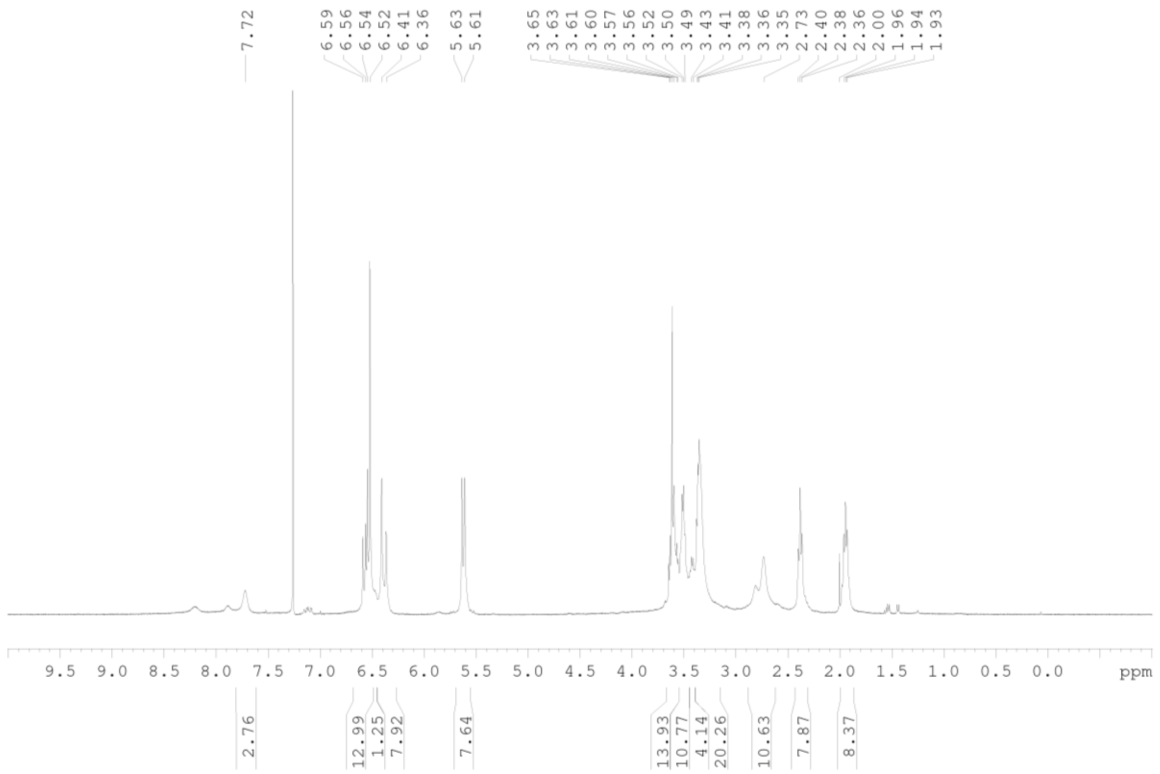

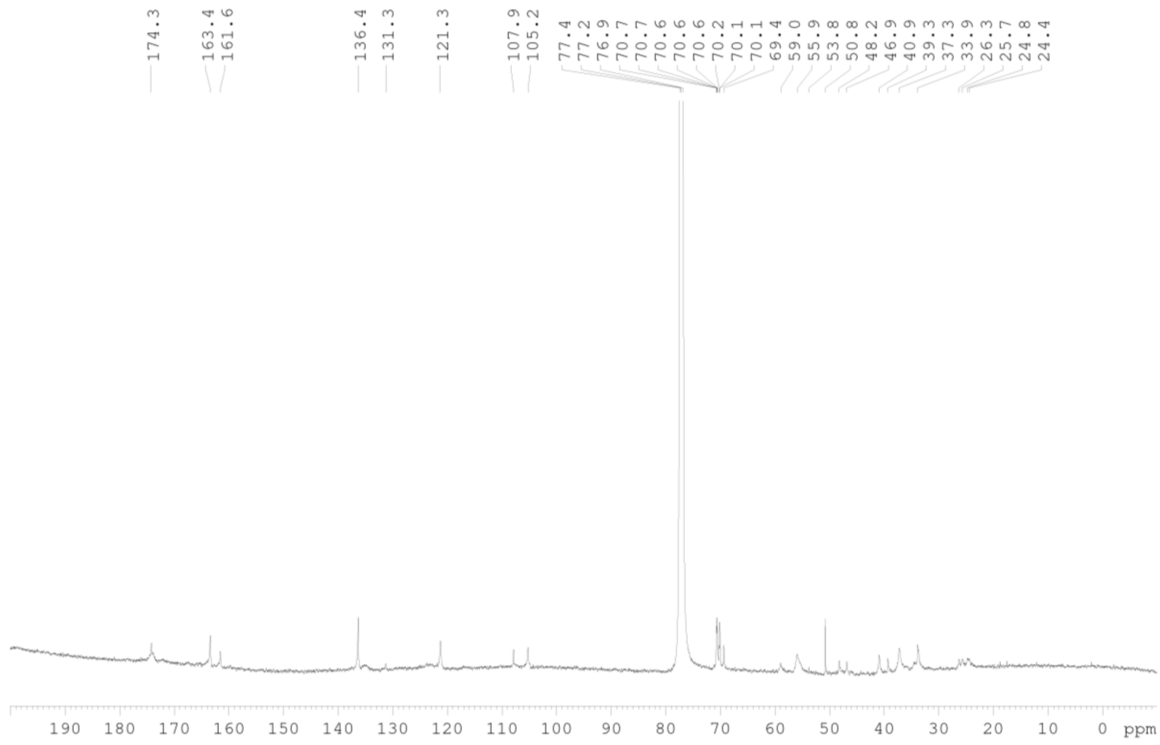

Bis-(*N*-Fmoc-PEG$_2$)-triamine with PEG$_3$ azide side chain (**S9**)

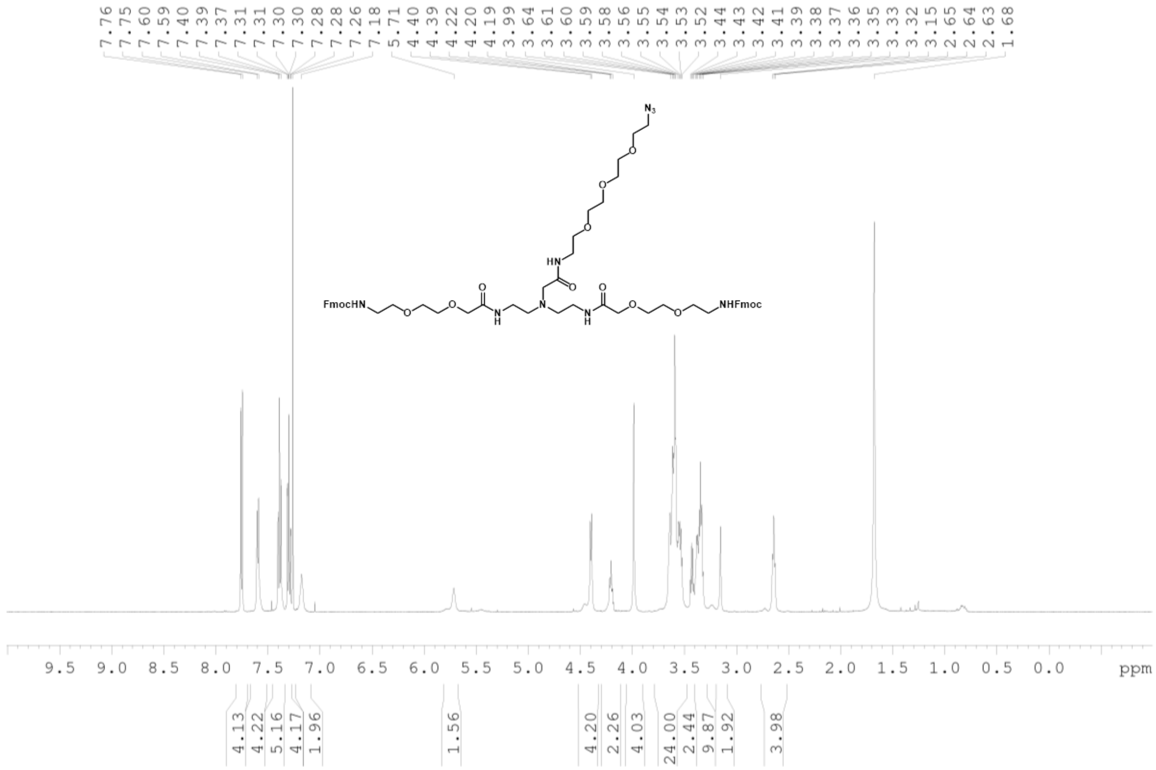

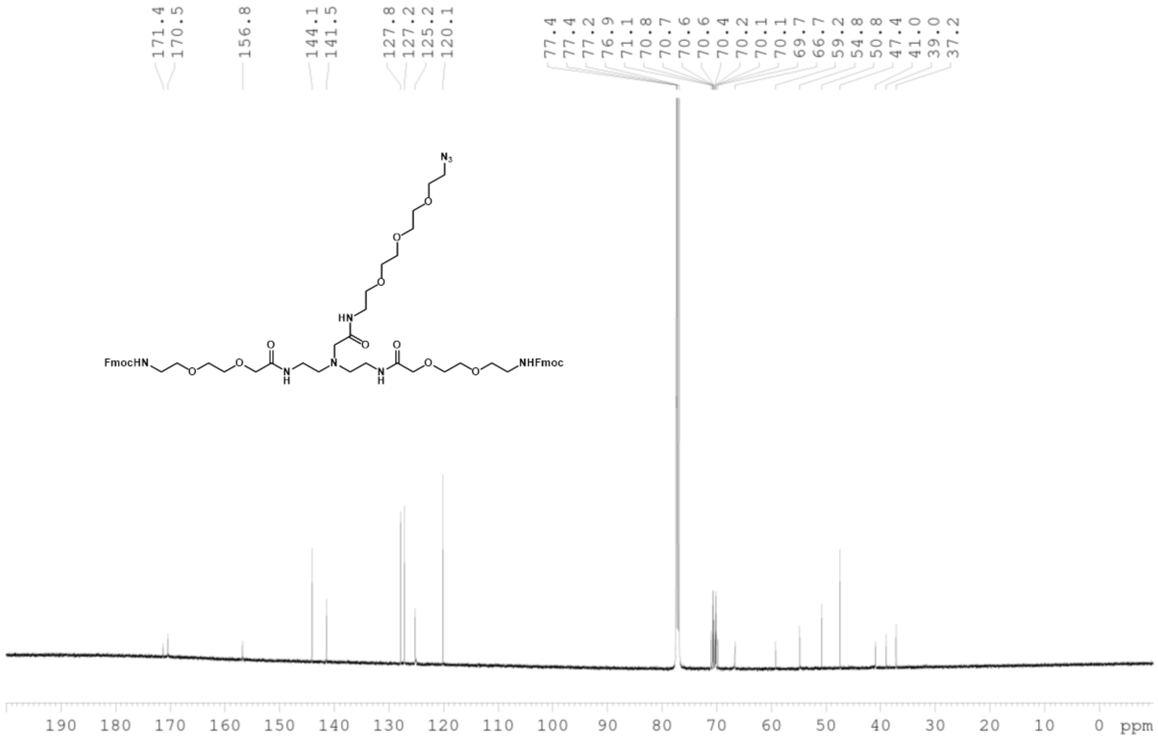

Tetra-*N*-Boc backbone with PEG_2 units inside the branch point and PEG_3 azide side chain (**S10**)

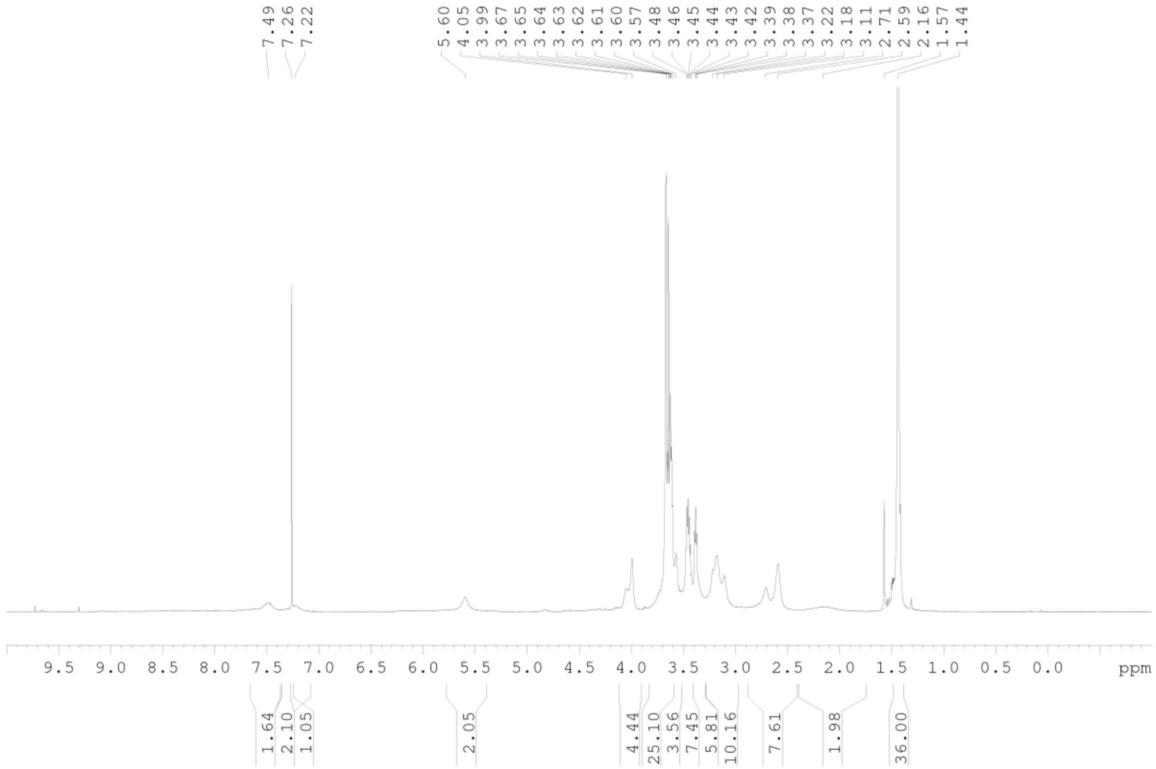

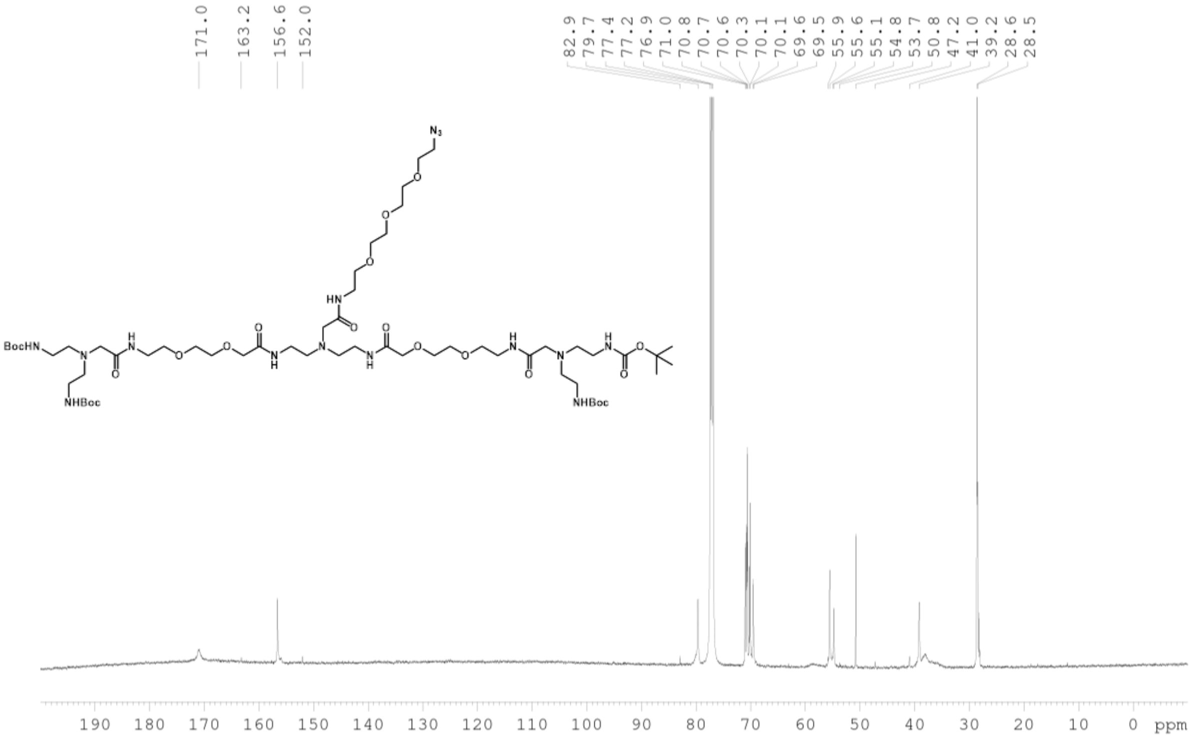

TetraDVP backbone with PEG_2 units inside the branch point and PEG_3 azide side chain (**1b**)

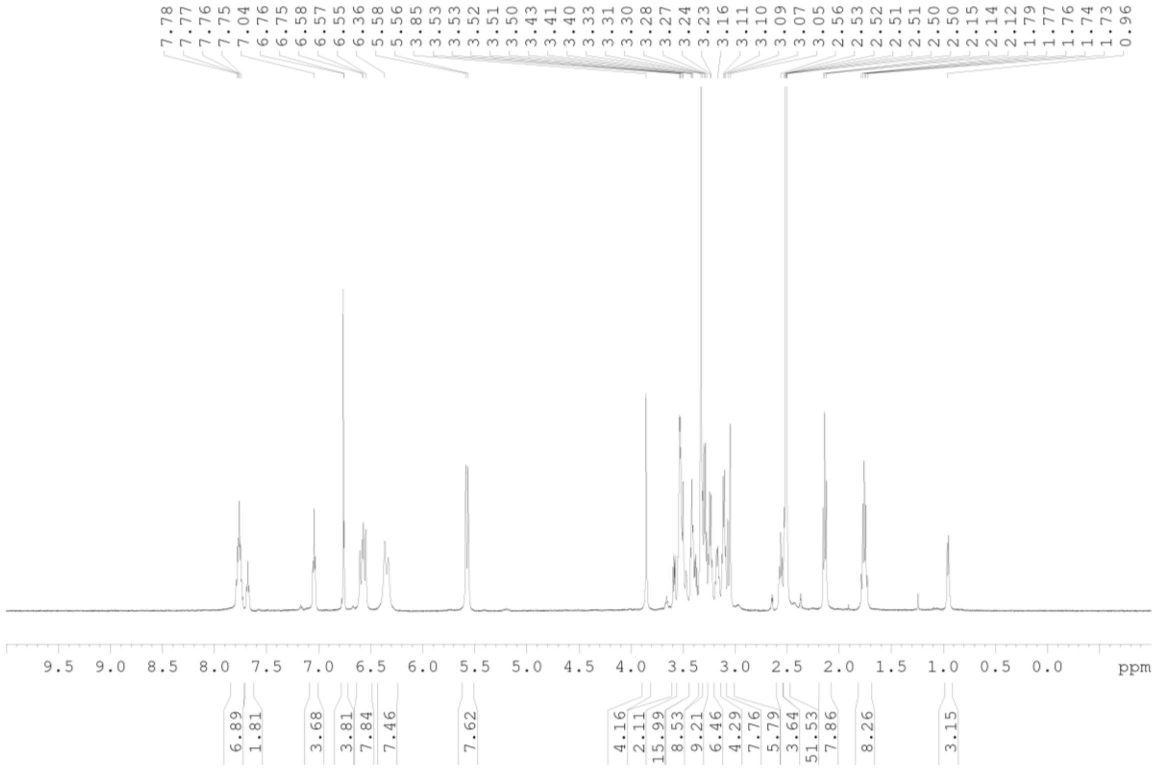

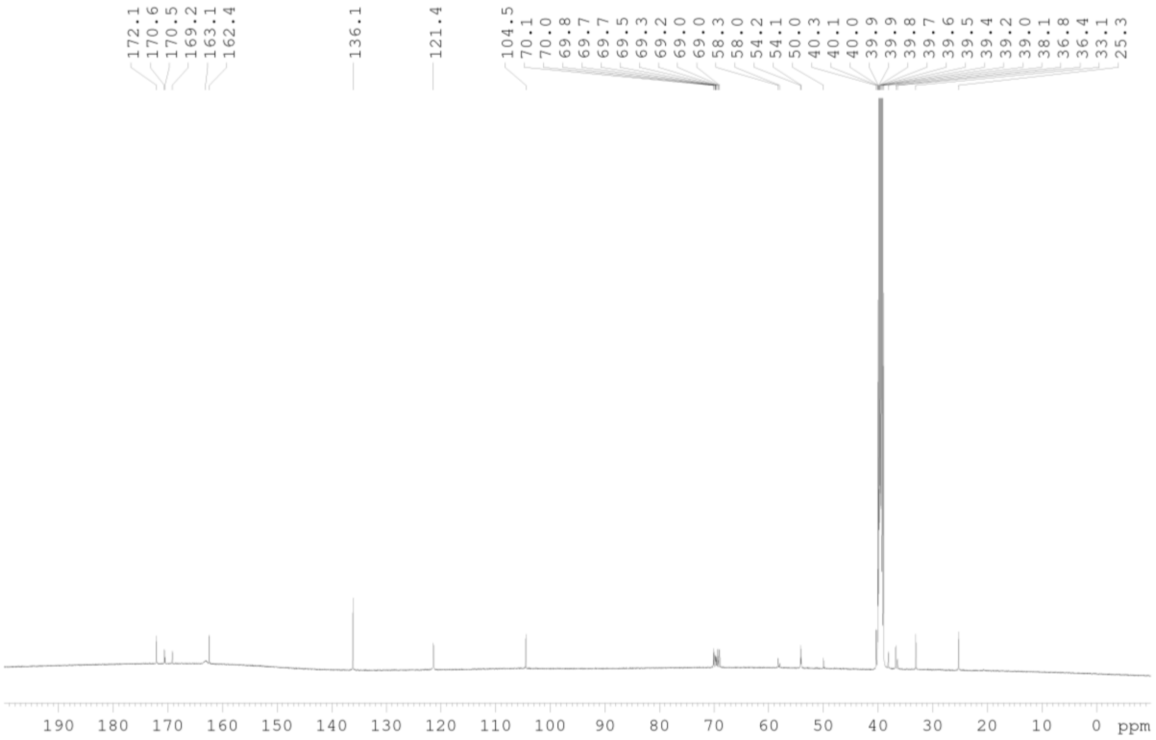

Tetra-*N*-Fmoc backbone with PEG_2 units outside the branch point and PEG_3 azide side chain (**S11**)

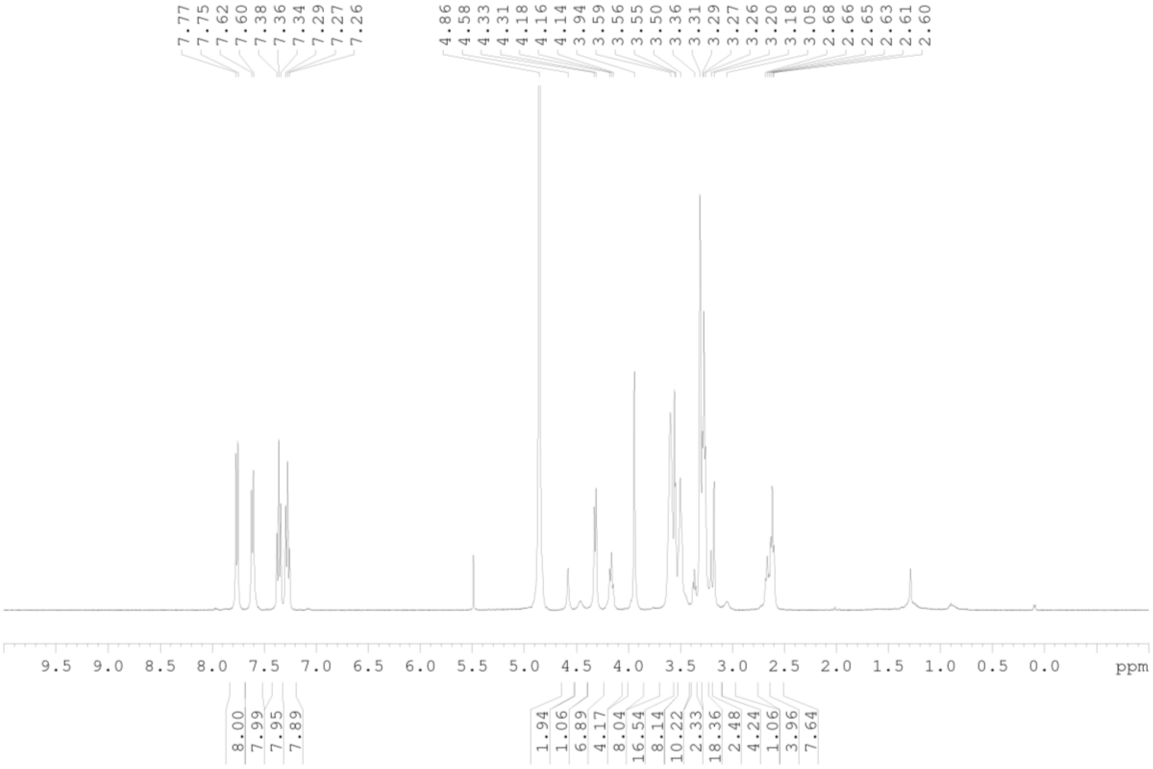

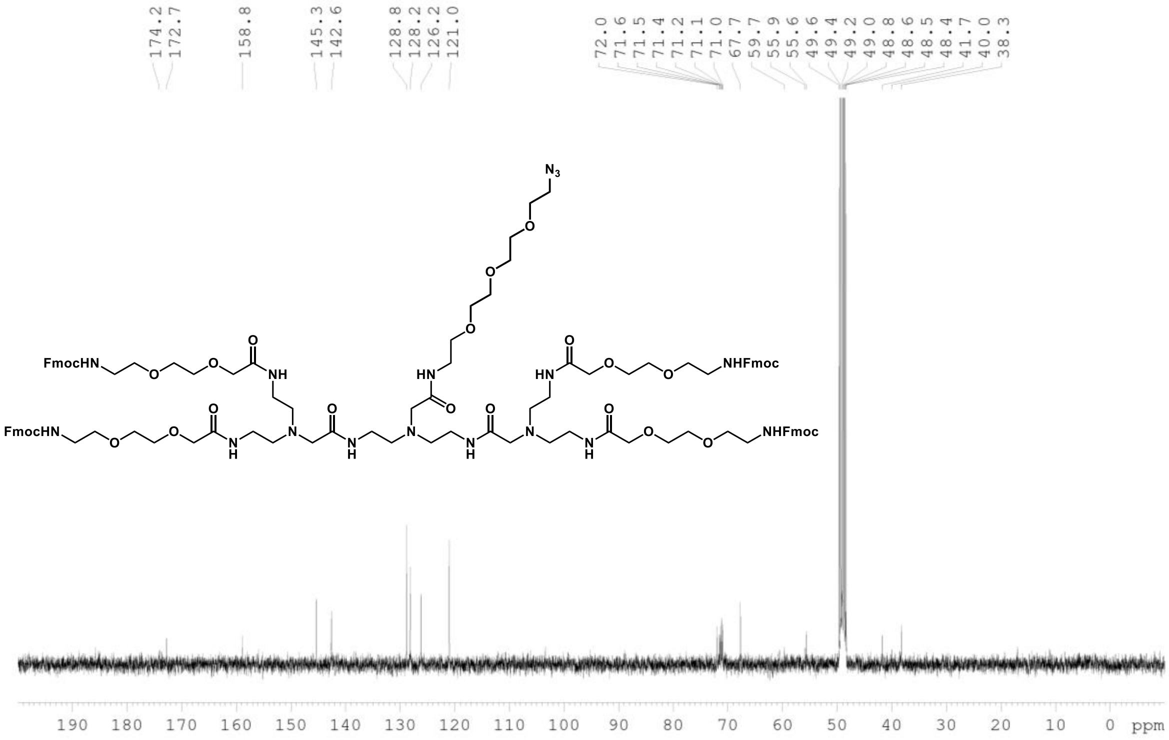

TetraDVP backbone with PEG_2 units outside the branch point and PEG_3 azide side chain (**1c**)

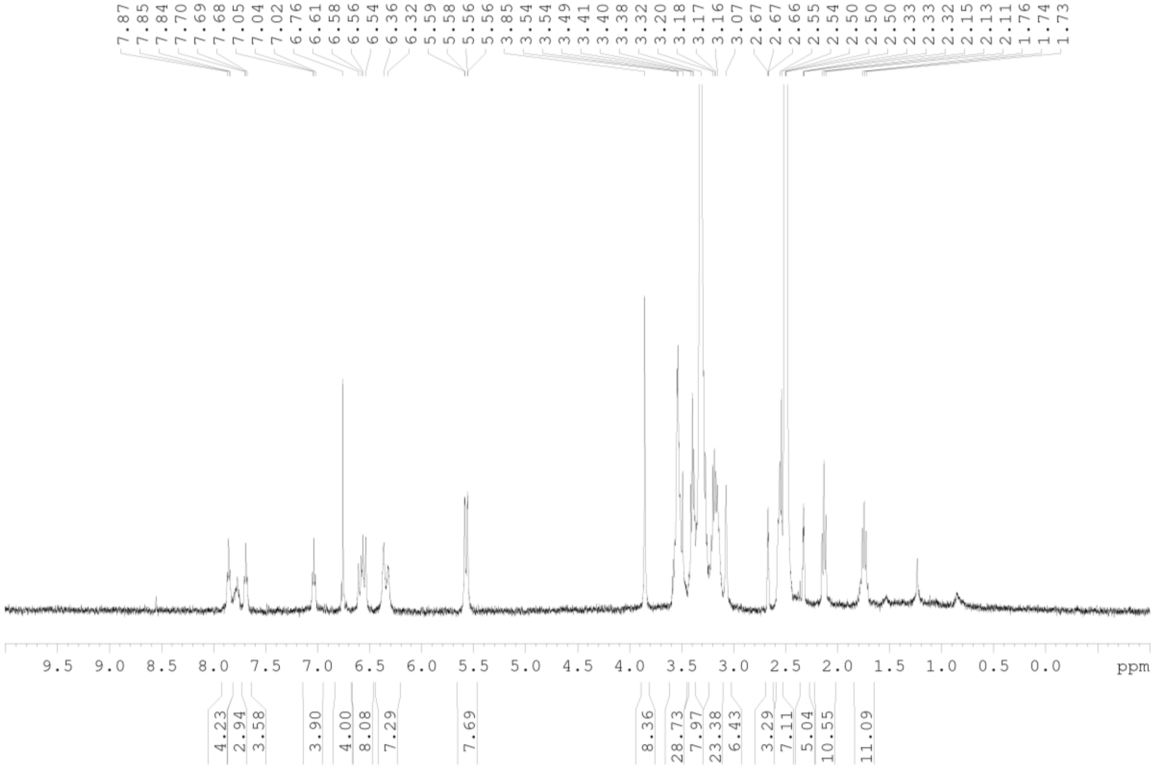

Tetra-*N*-Fmoc backbone with PEG_2 units inside and outside the branch point and PEG_3 azide side chain (**S12**)

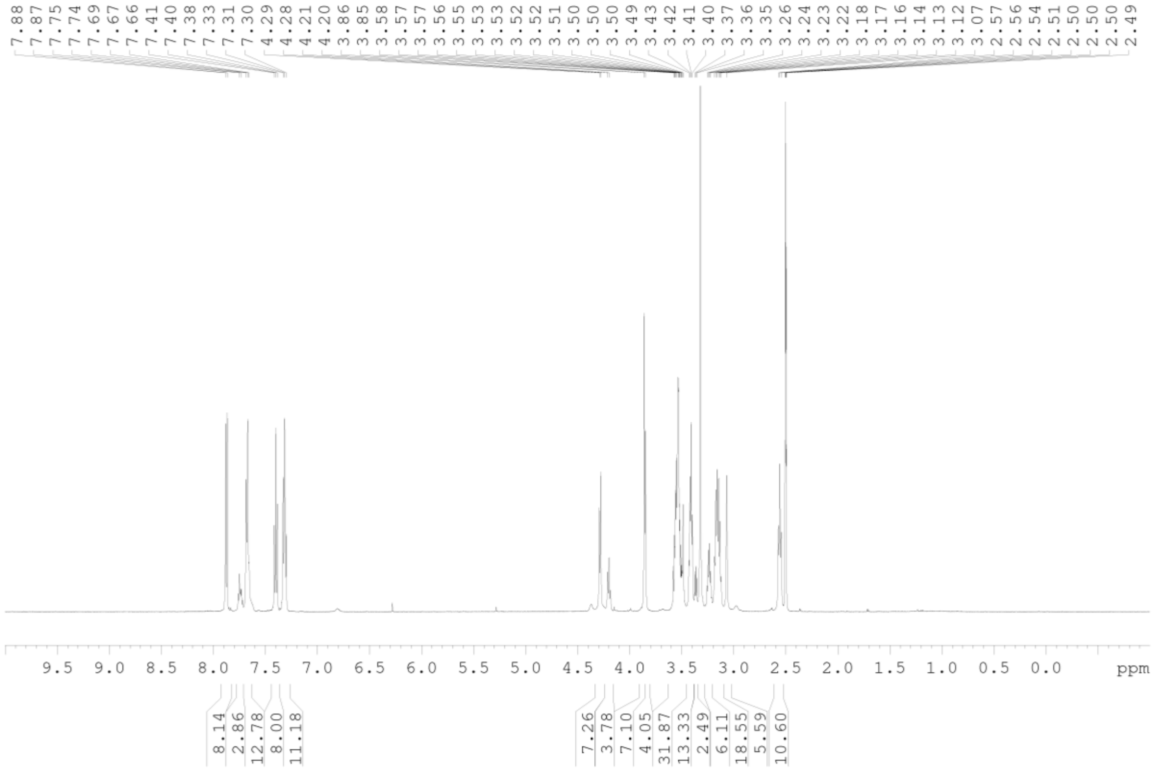

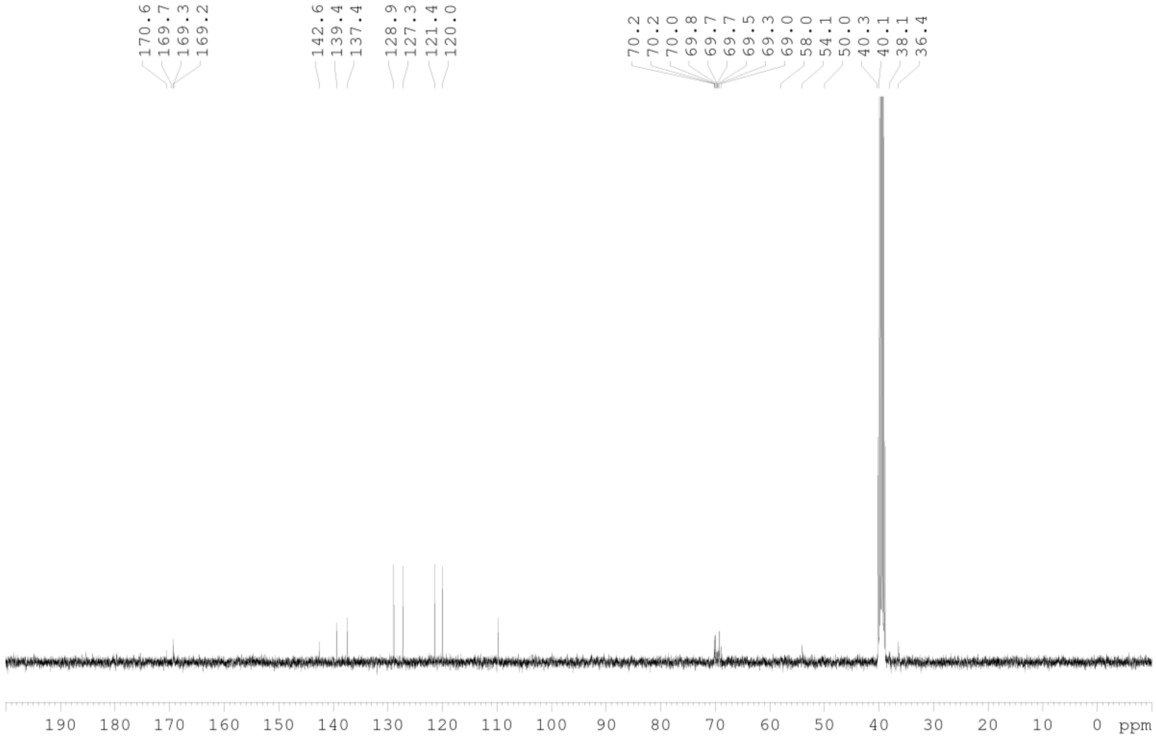

TetraDVP backbone with PEG_2 units inside and outside the branch point and PEG_3 azide side chain (**1d**)

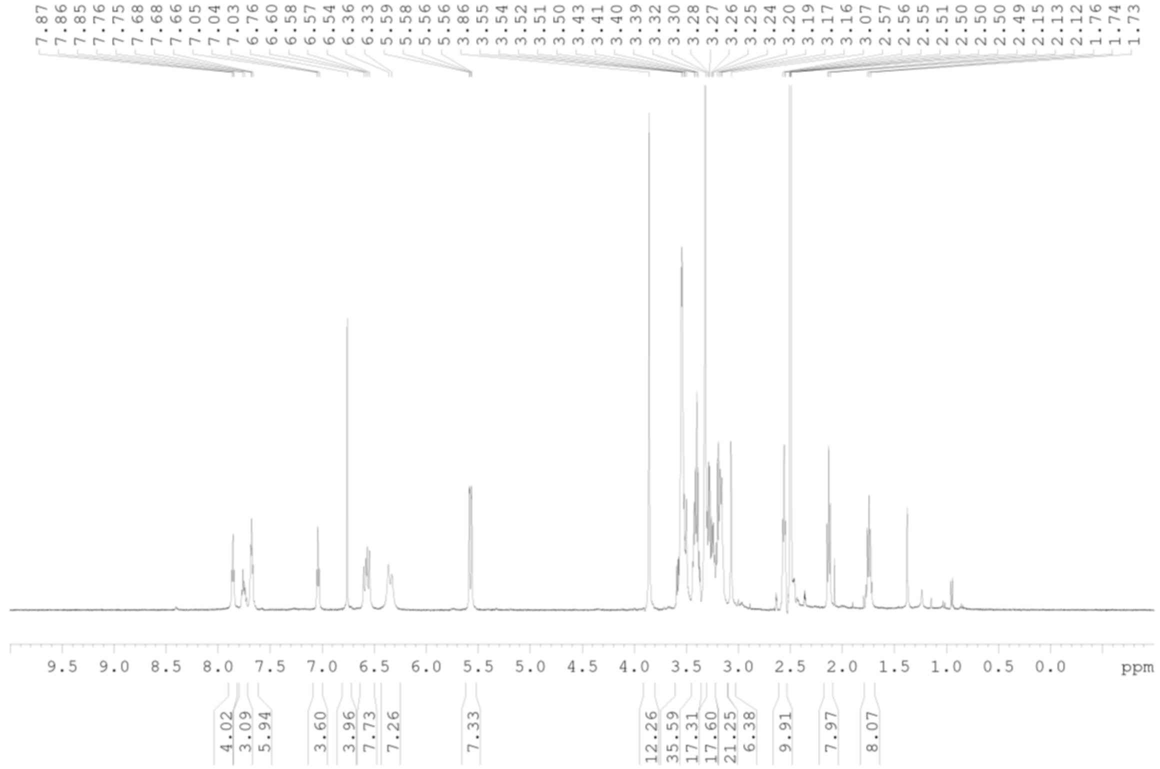

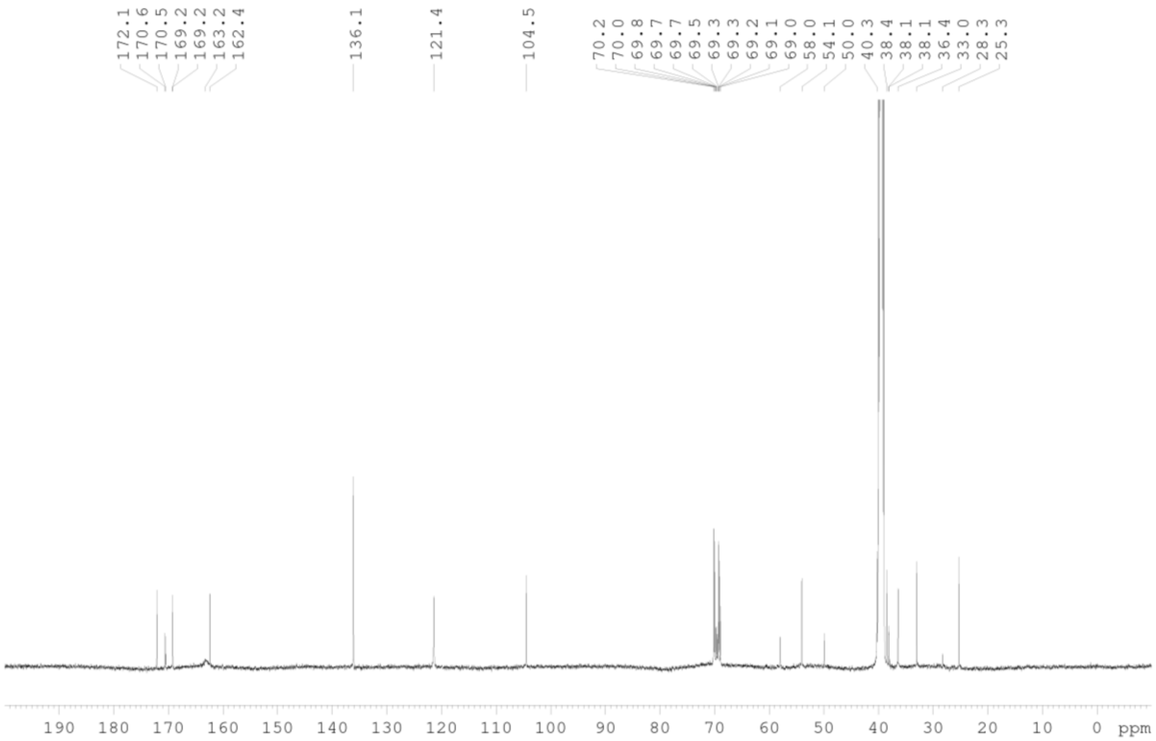

Bis(2-((((9*H*-fluoren-9-yl)methoxy)carbonyl)amino)ethyl)glycine (**S13**)

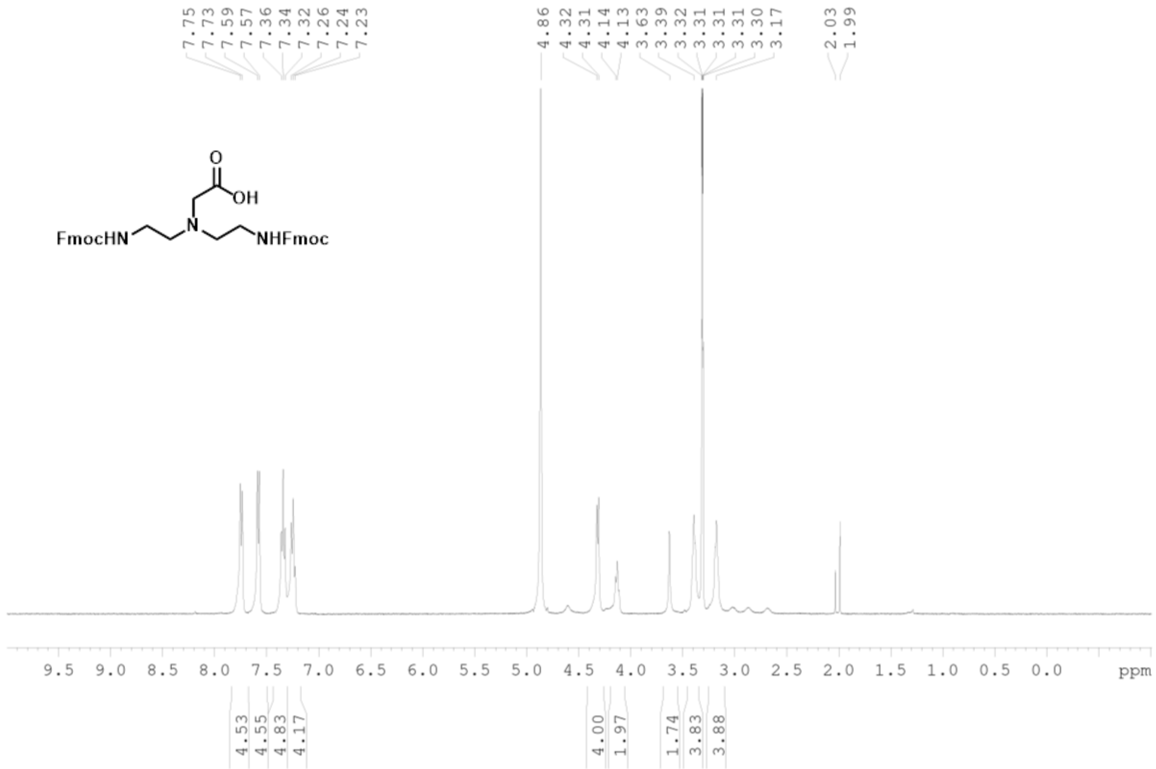

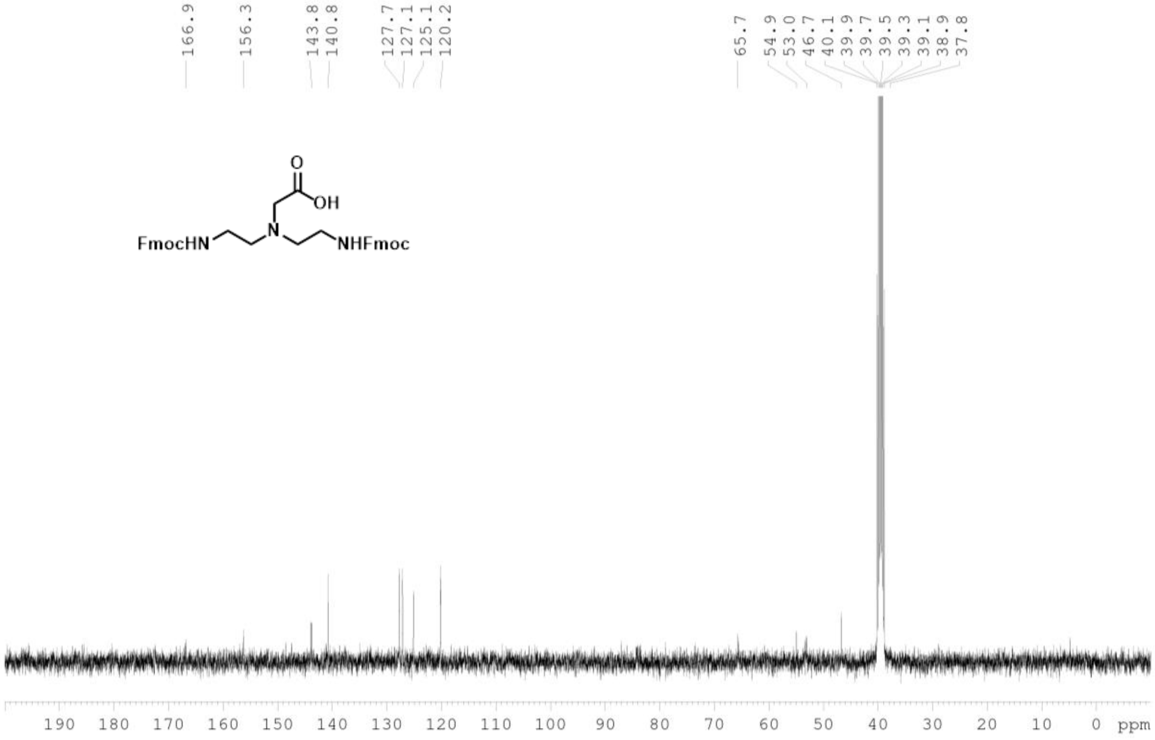

Bis((9*H*-fluoren-9-yl)methyl) (8,16-dioxo-3,6,18,21-tetraoxa-9,12,15-triazatricosane-1,23-diyl)dicarbamate (**S14**)

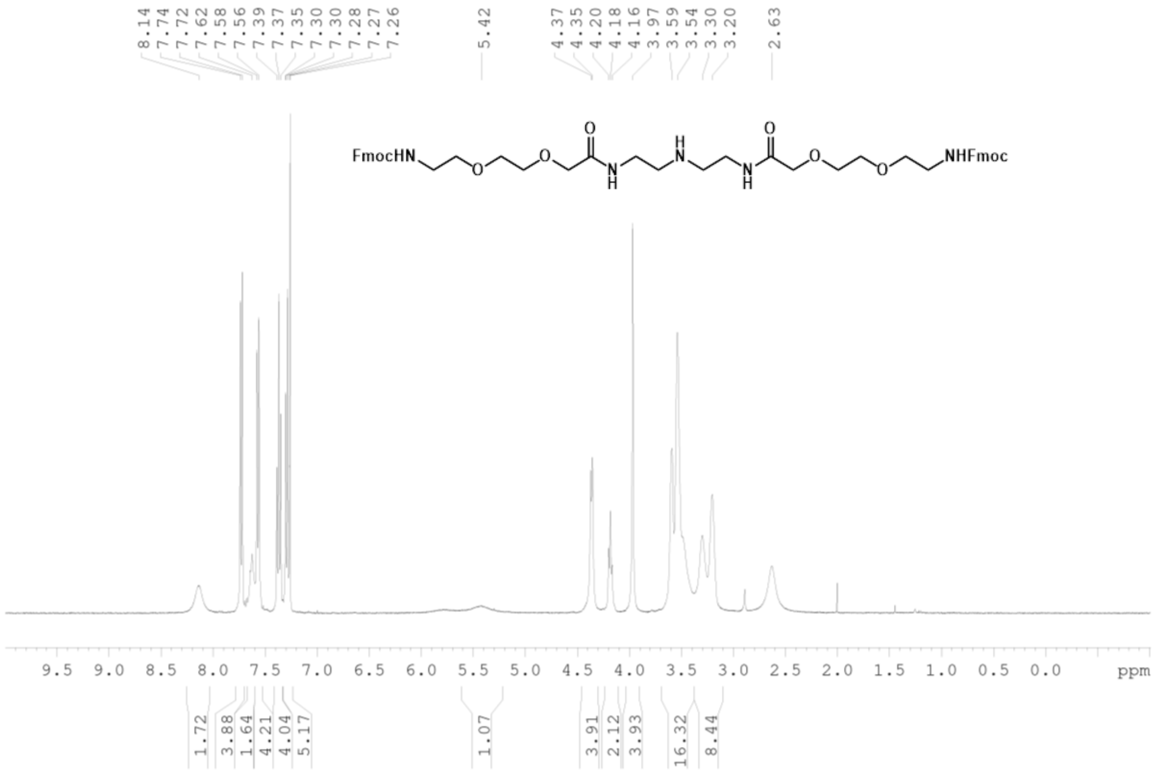

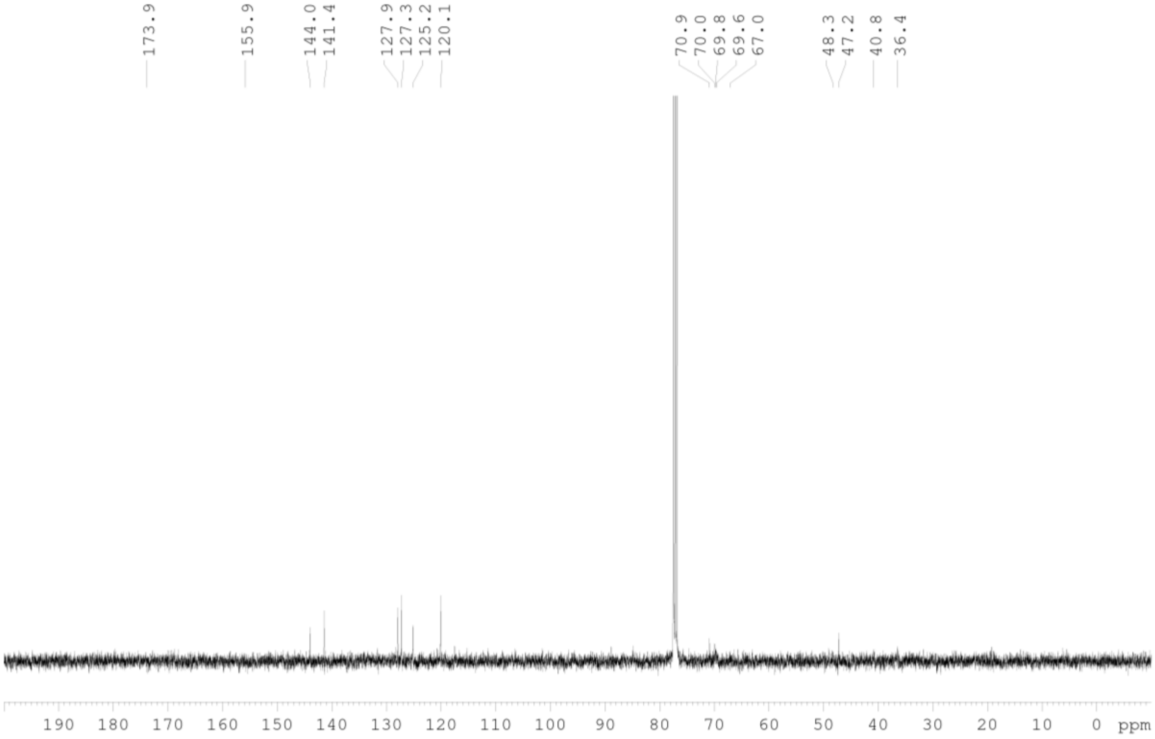

tert-Butyl (3-(2-bromoacetamido)propyl)carbamate (**S15**)

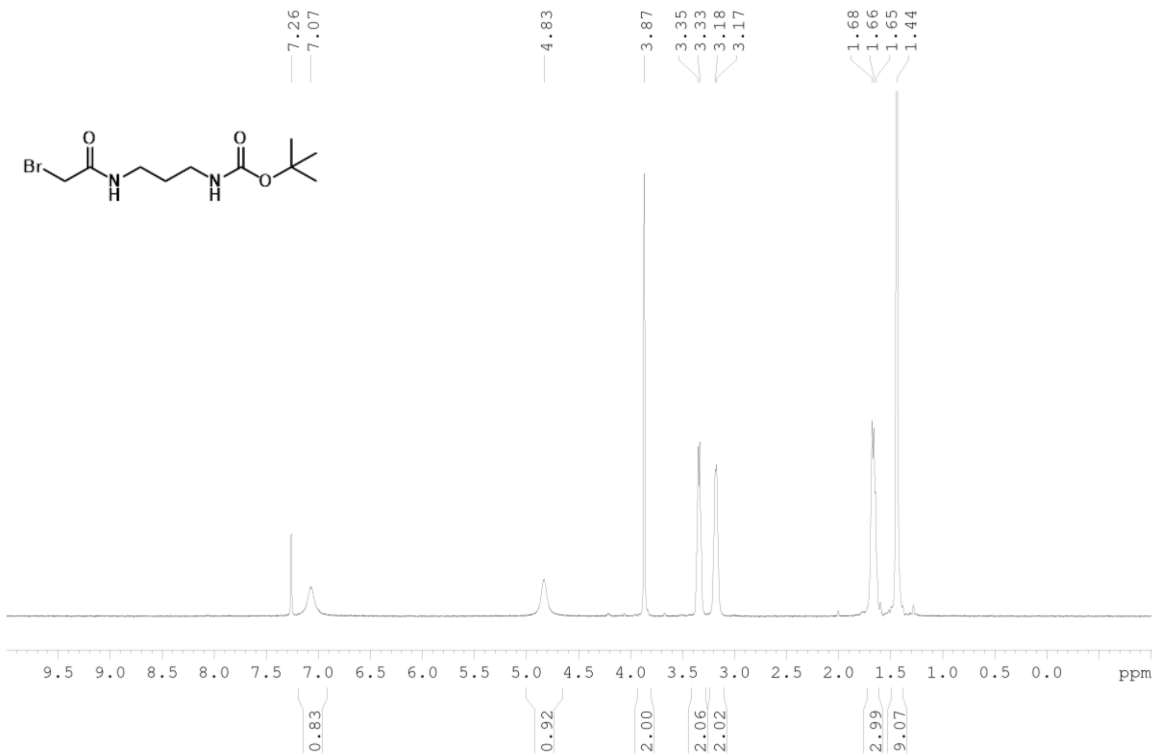

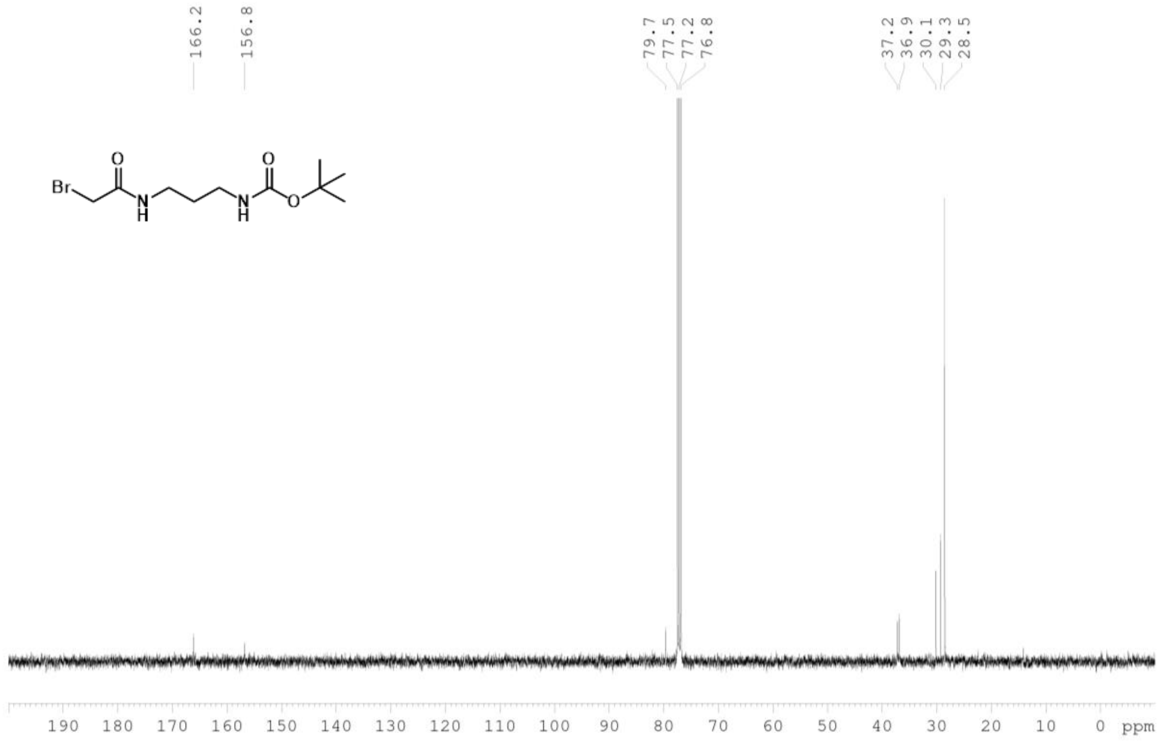

Bis((9*H*-fluoren-9-yl)methyl) (12-(2-((3-((*tert*-butoxycarbonyl)amino)propyl)amino)-2-oxoethyl)-8,16-dioxo-3,6,18,21-tetraoxa-9,12,15-triazatricosane-1,23-diyl)dicarbamate (**S16**)

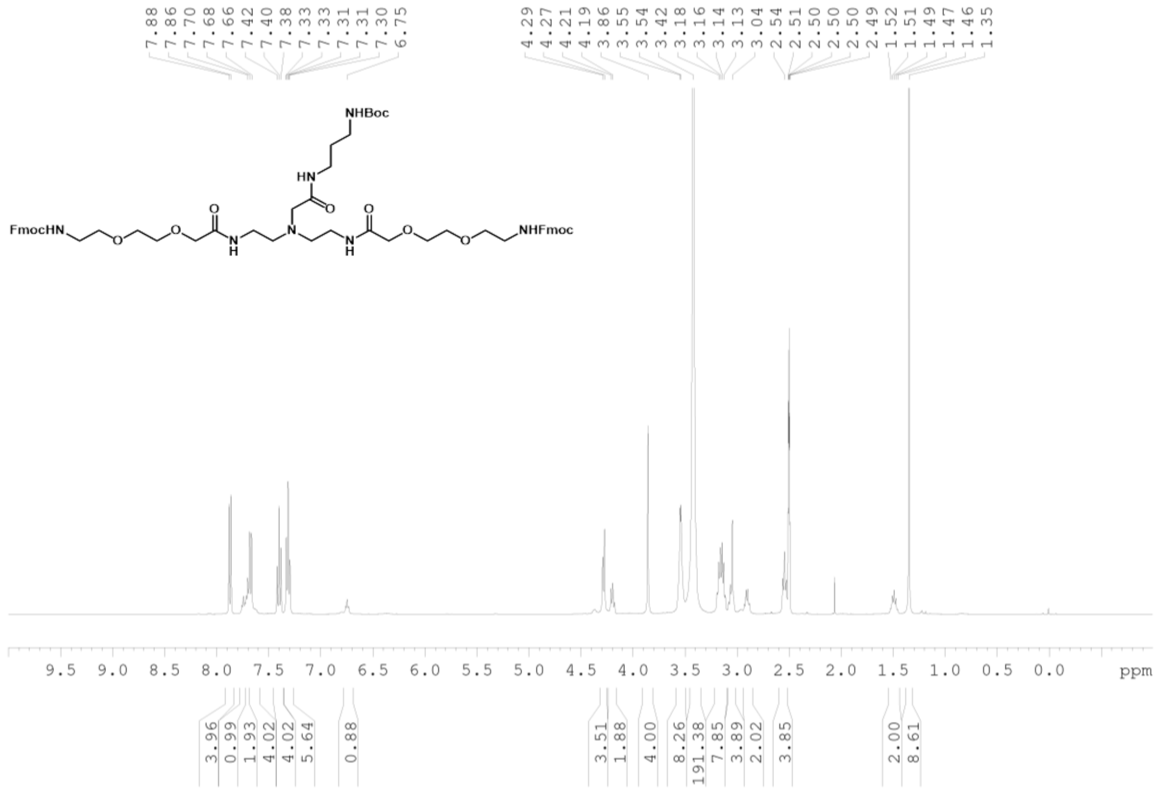

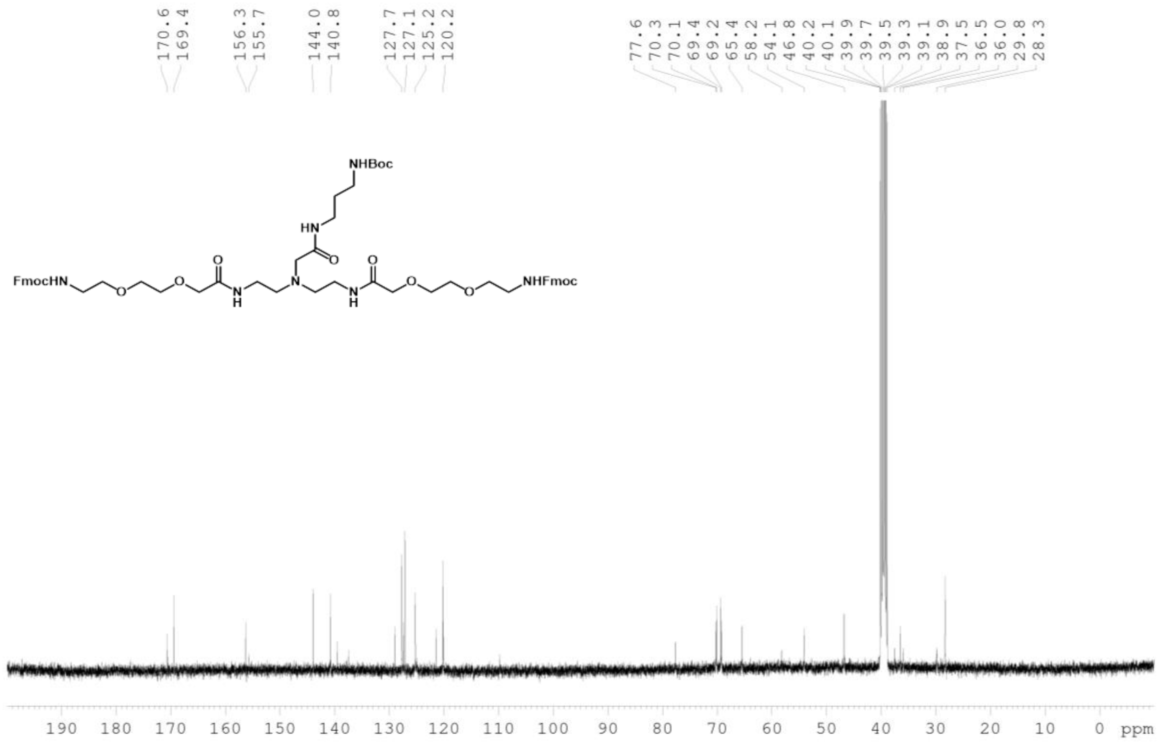

Tetra-*N*-Fmoc backbone with PEG_2 units inside the branch point and *N*-Boc-amine side chain (**S17**)

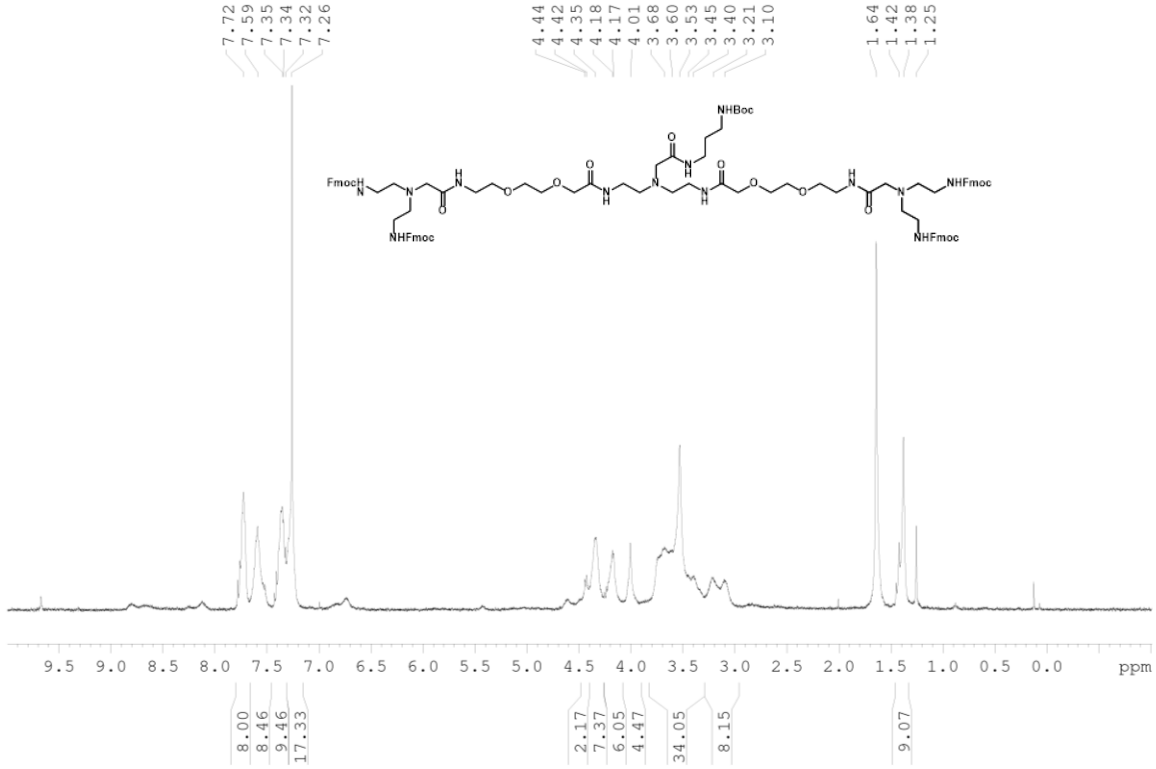

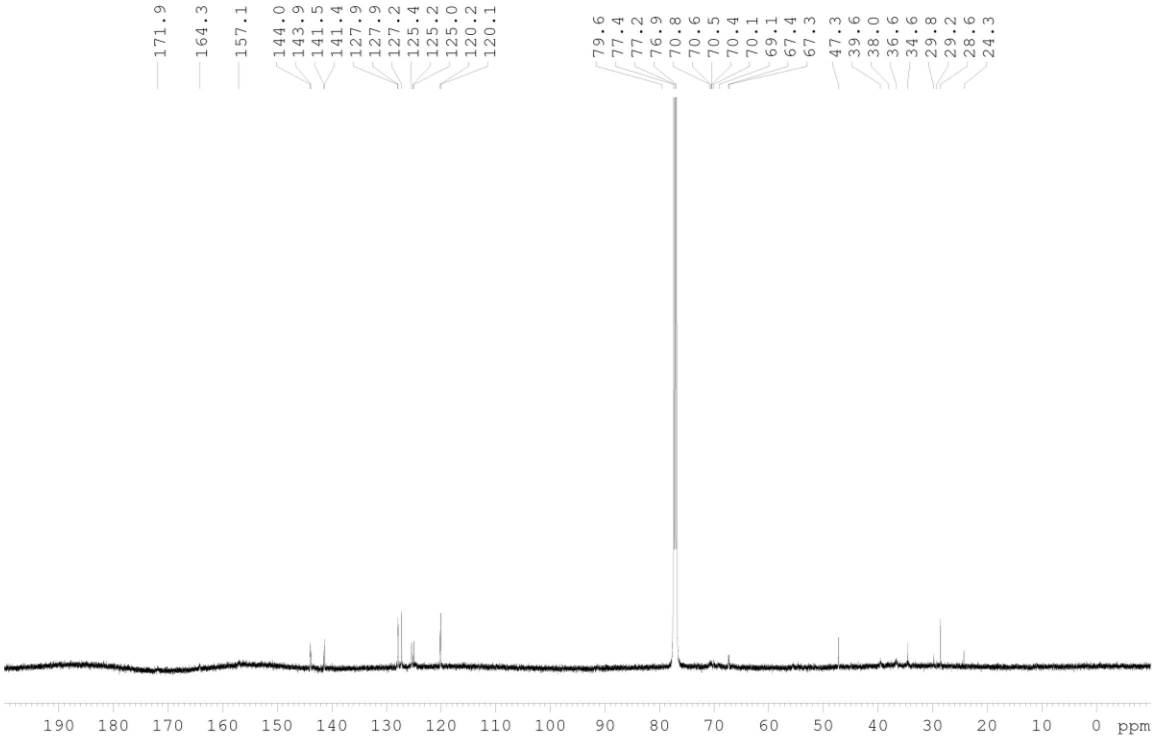

Tetra-*N*-Fmoc backbone with PEG_2 units inside the branch point and PEG_4 azide side chain (**S18**)

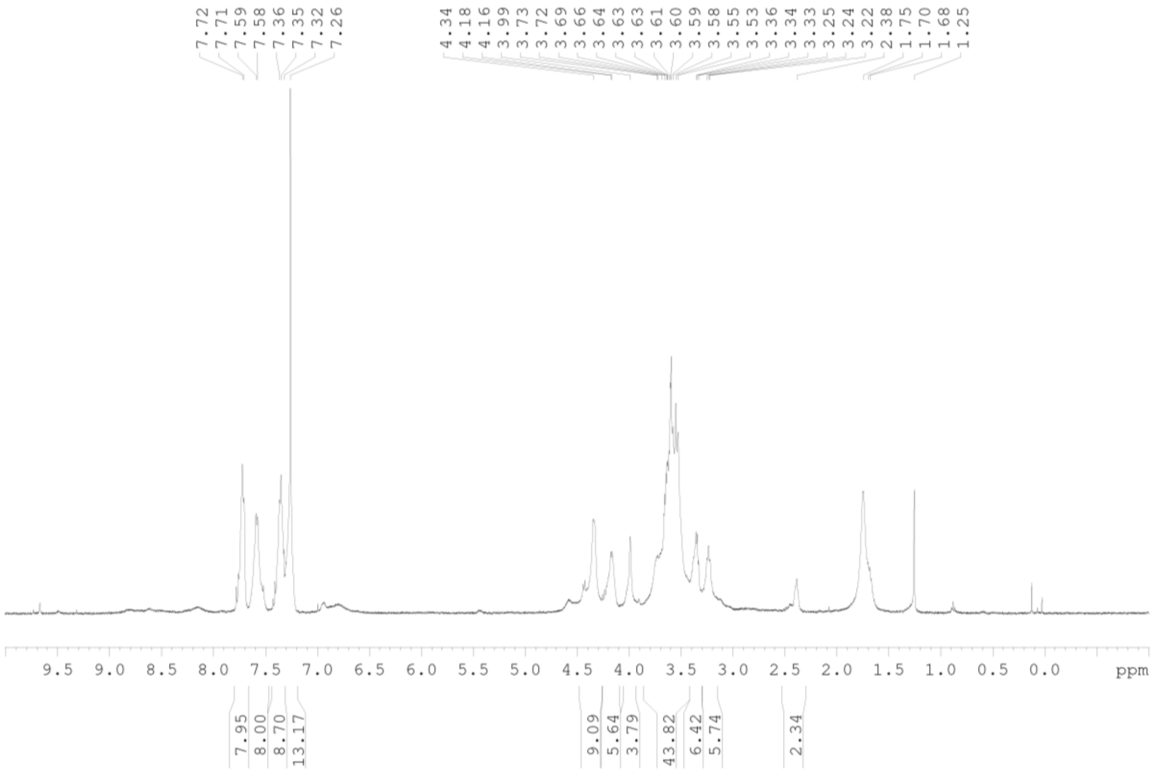

Fmoc-Val-Cit-OH (**S19**)

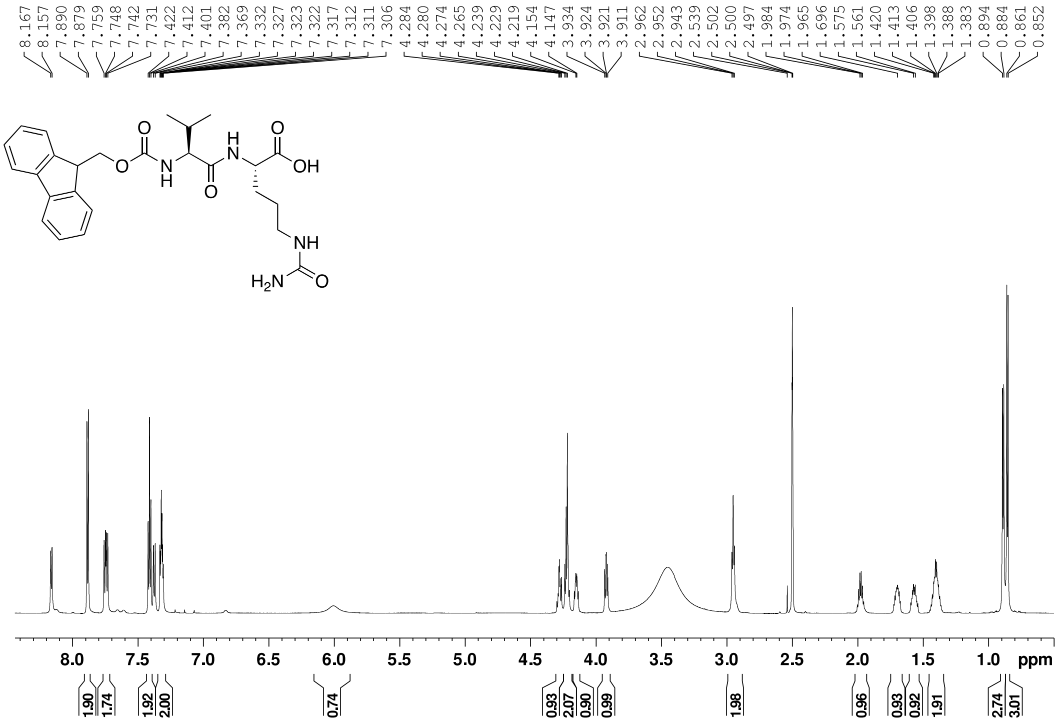

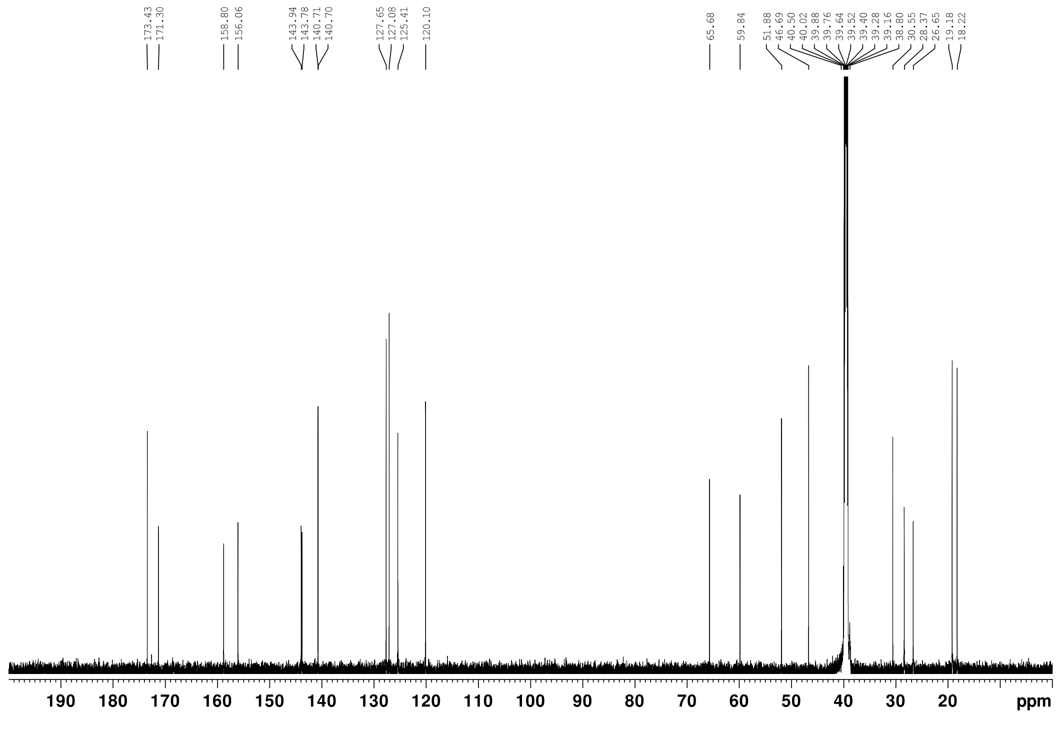

Fmoc-Val-Cit-PABA (**S20**)

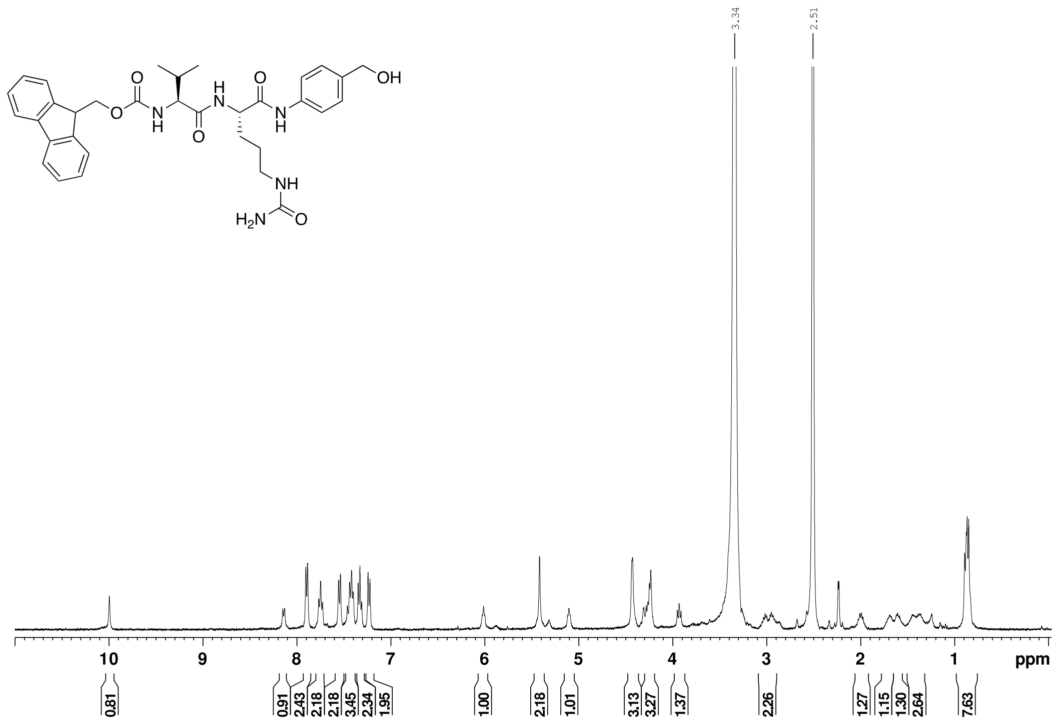

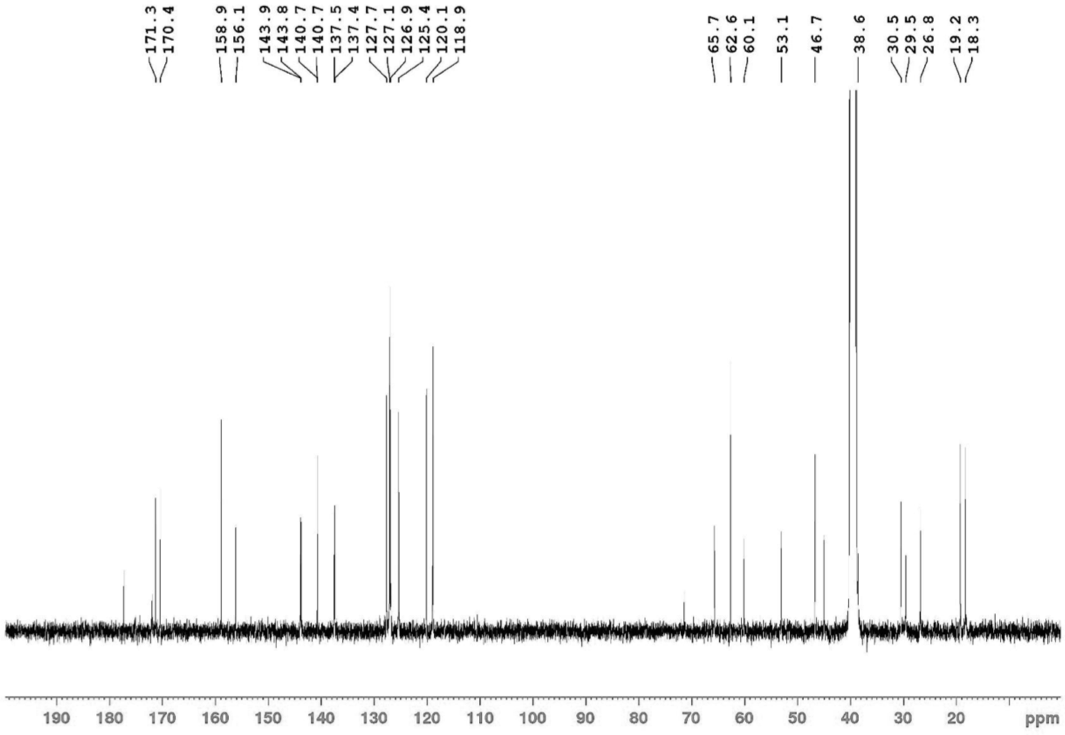

HO – PEG_4 – $CH_2CH_2CO_2tBu$ (**S22**)

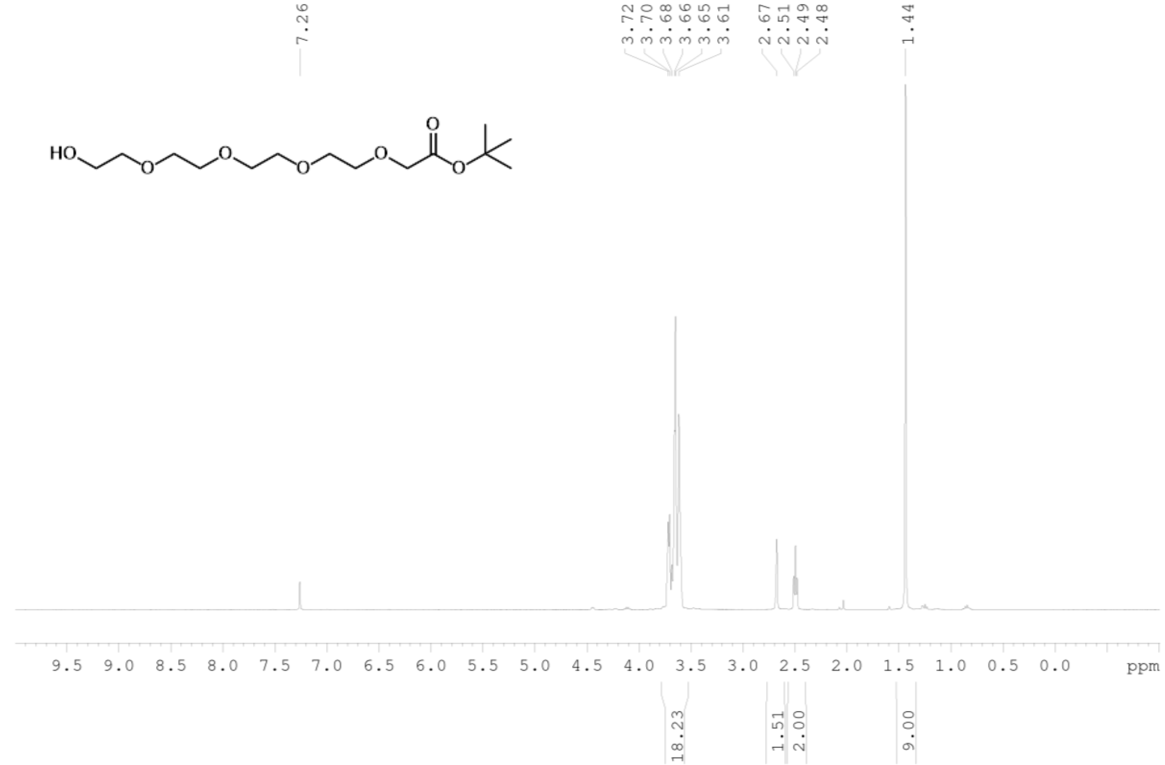

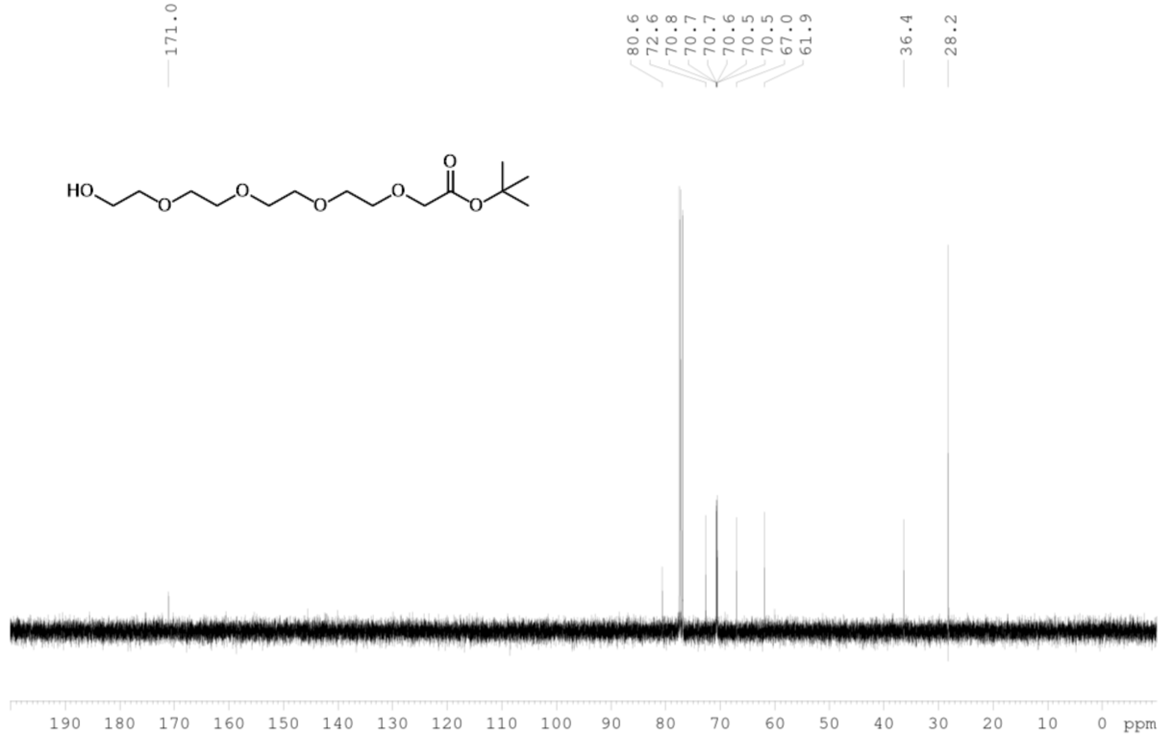

TsO – PEG_4 – $CH_2CH_2CO_2$tBu (**S23**)

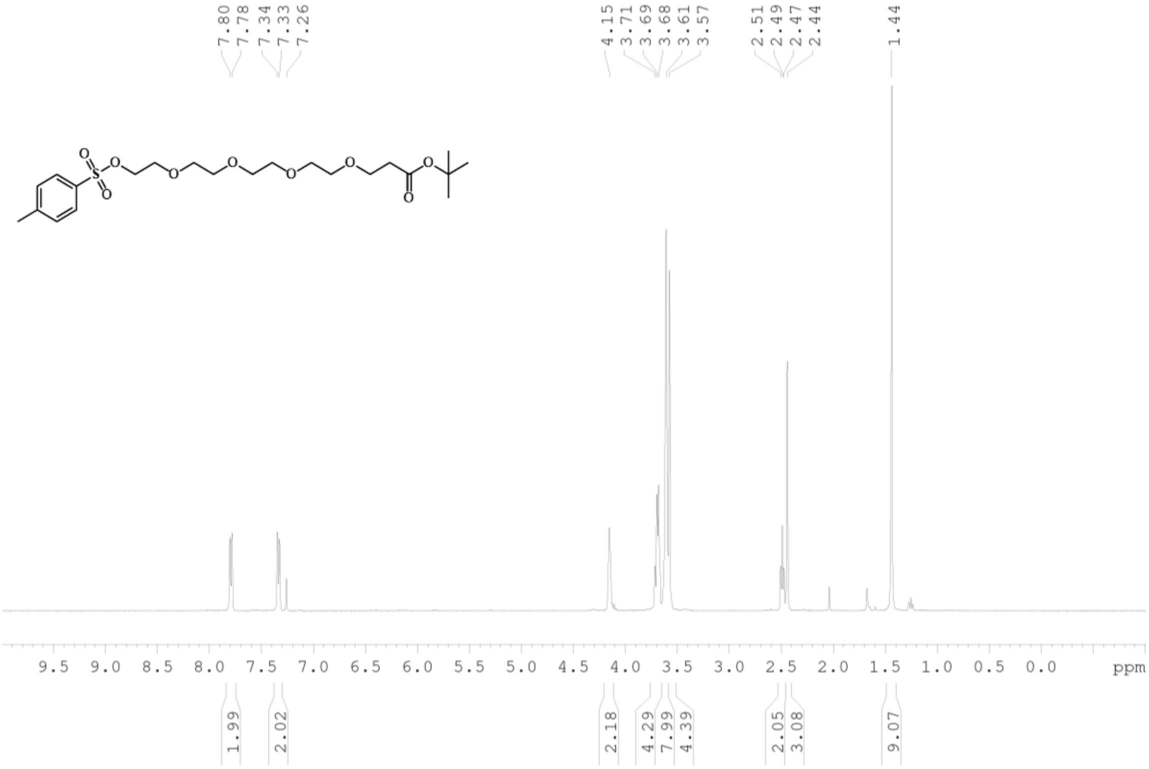

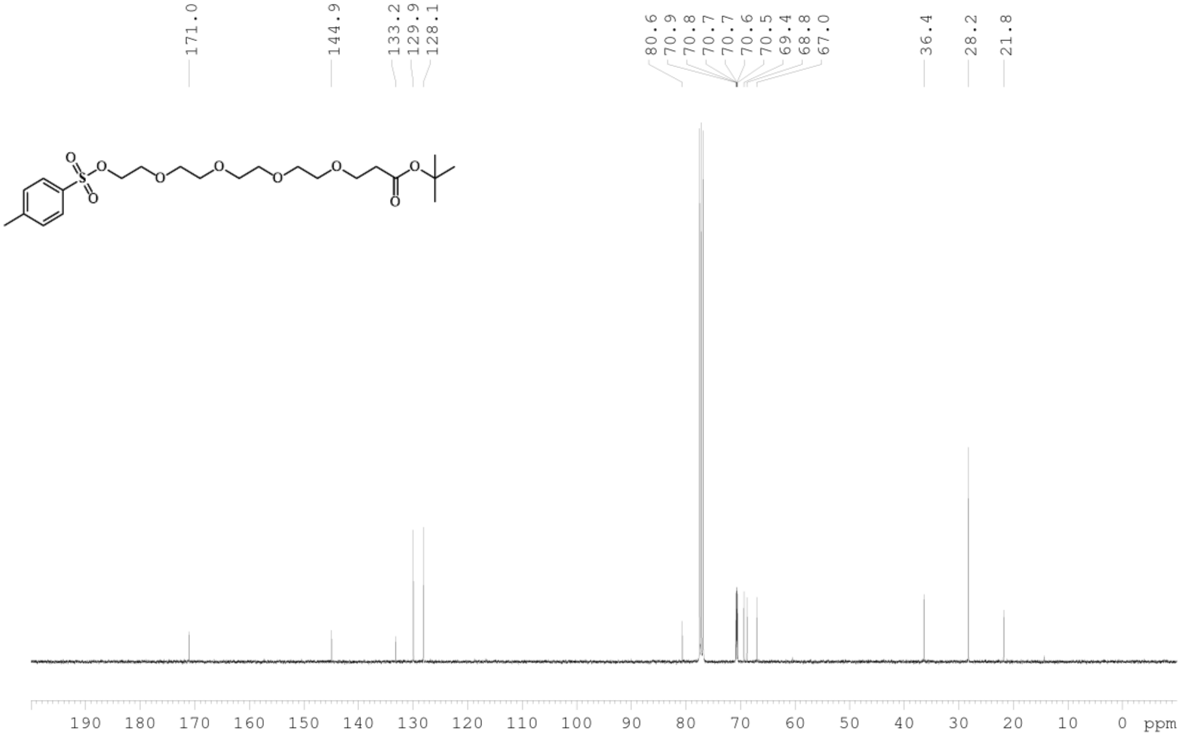

$N_3 - PEG_4 - CH_2CH_2CO_2tBu$ (**S24**)

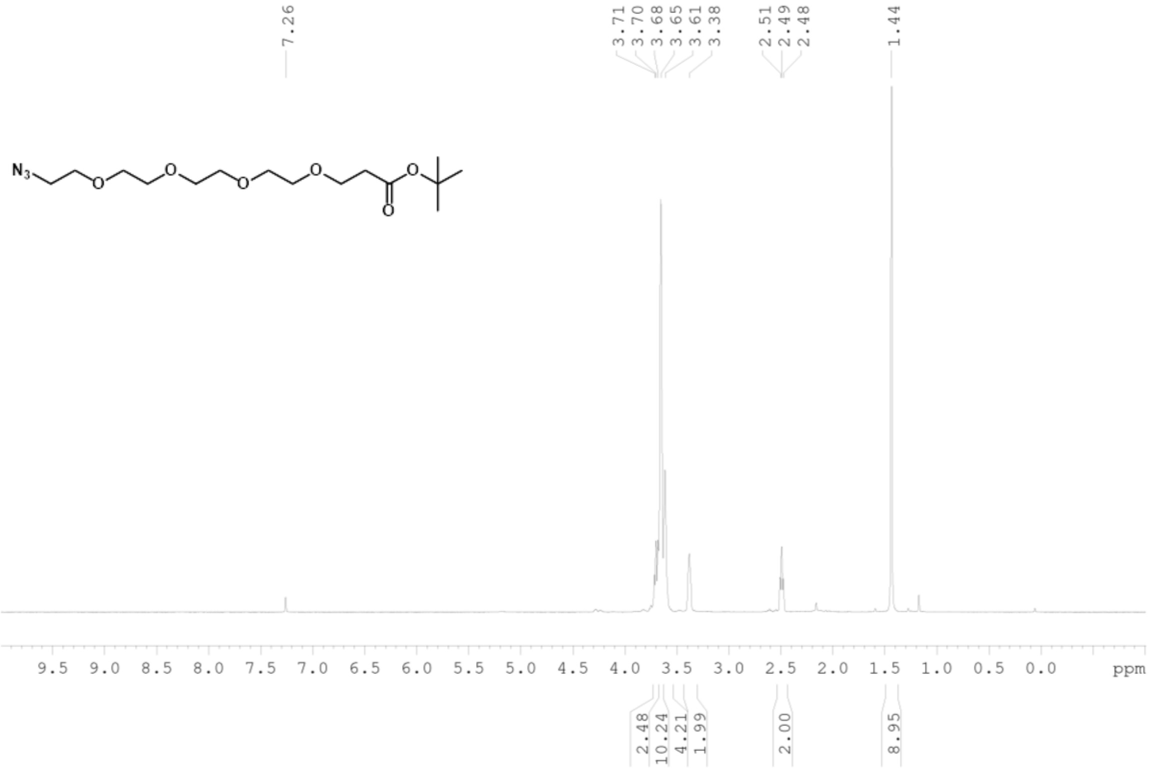

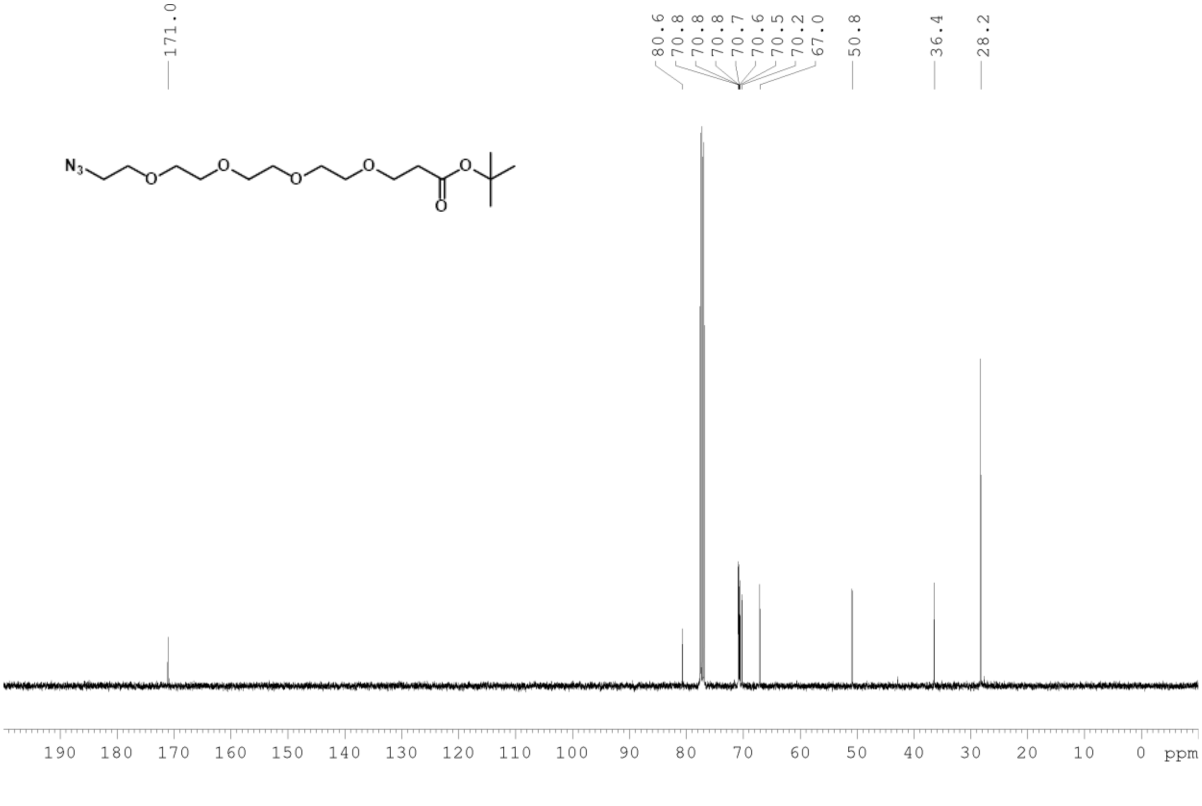

$N_3 – PEG_4 – CH_2CH_2CO_2H$ (**S25**)

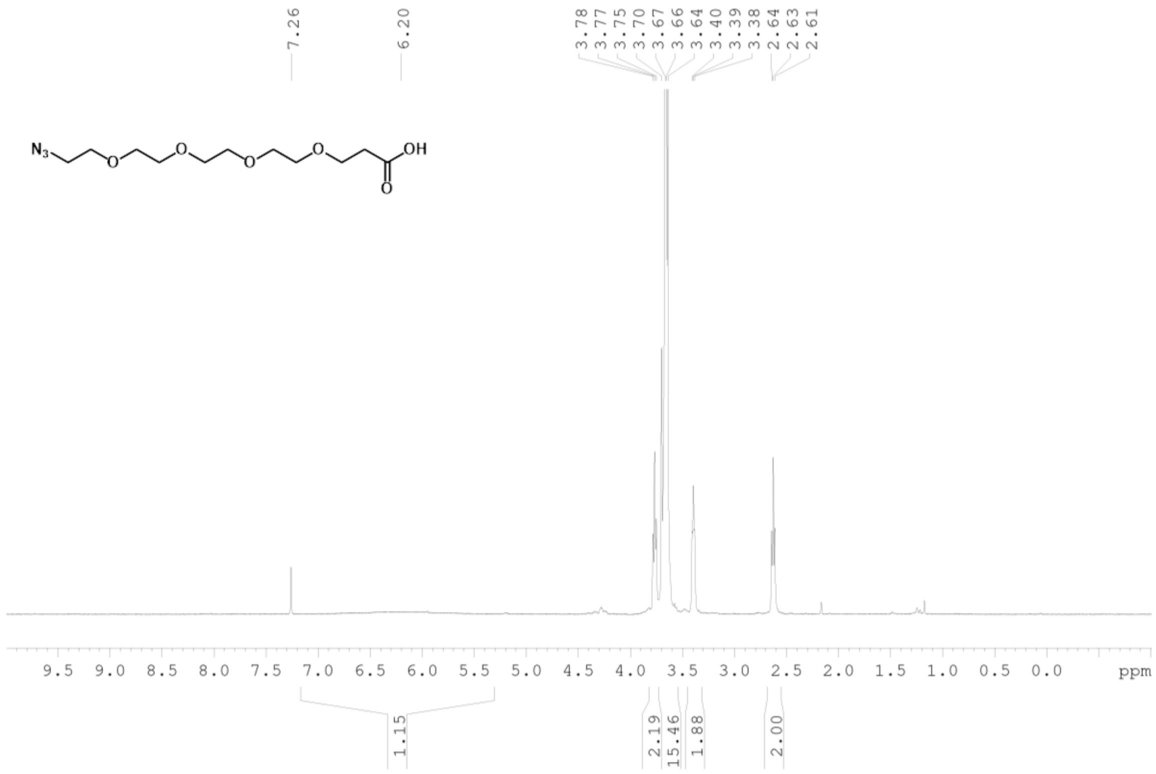

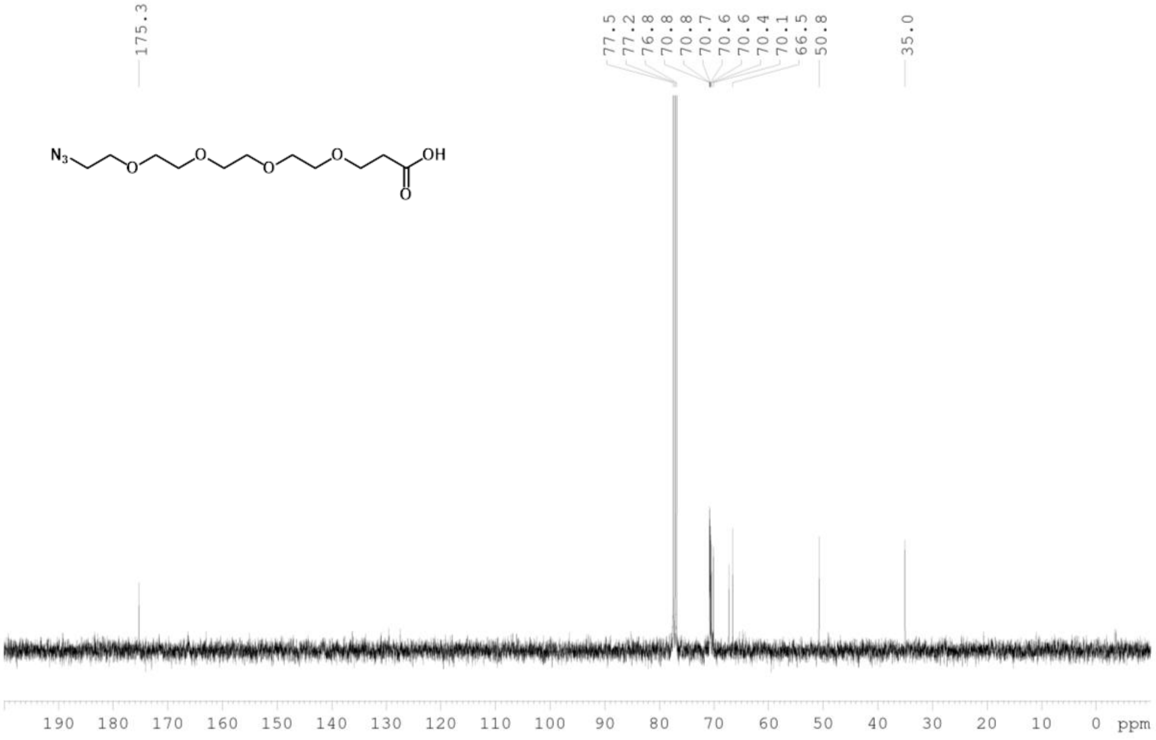

$N_3 – PEG_4 – CH_2CH_2CO_2Su$ (**S26**)

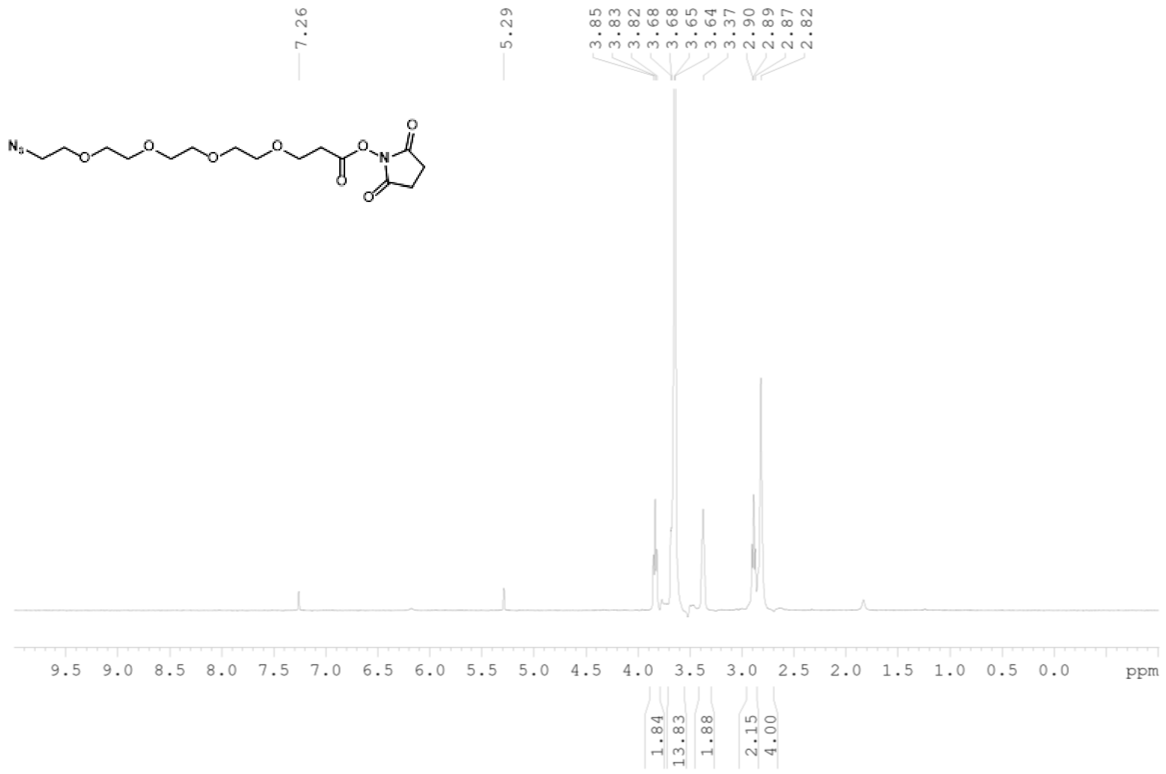

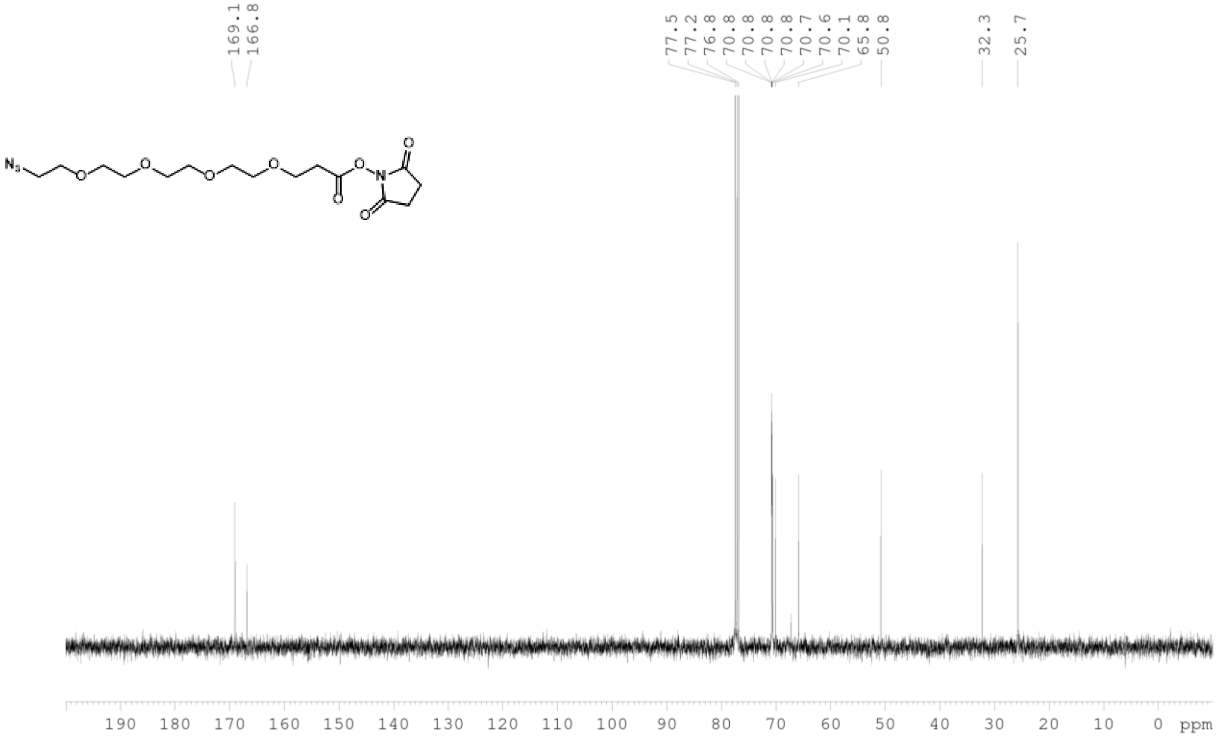

9 HPLC data

TetraDVP no-PEG backbone with PEG_3 azide sidechain (**1a**)

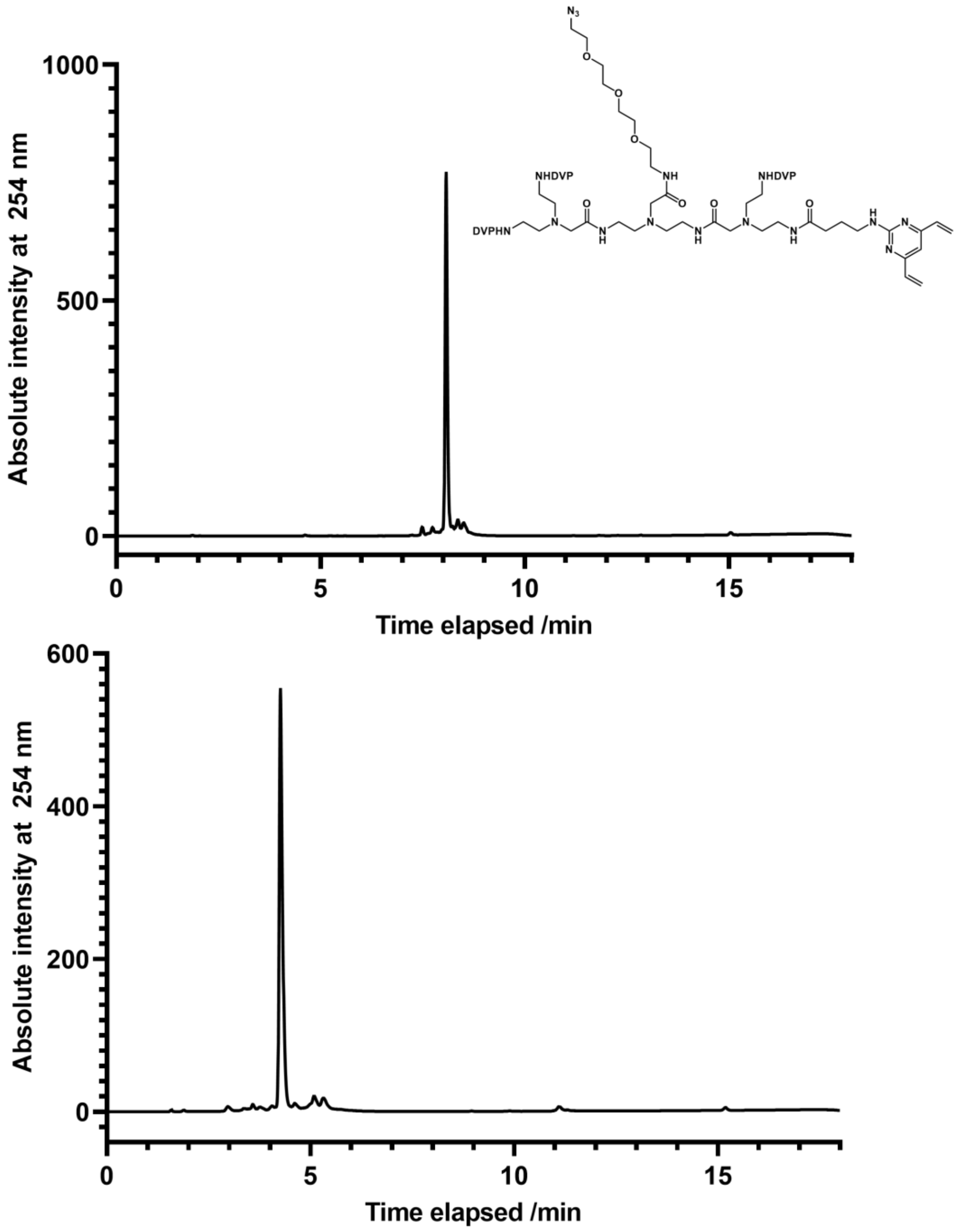

Figure S6: HPLC traces at 254 nm for compound **1a**. HPLC run with gradients of 5–95% solvent B in solvent A (top) and 30–80% solvent B in solvent A (bottom). Purity based on integration of >79%.

TetraDVP backbone with PEG_2 units inside the branch point and PEG_3 azide side chain (**1b**)

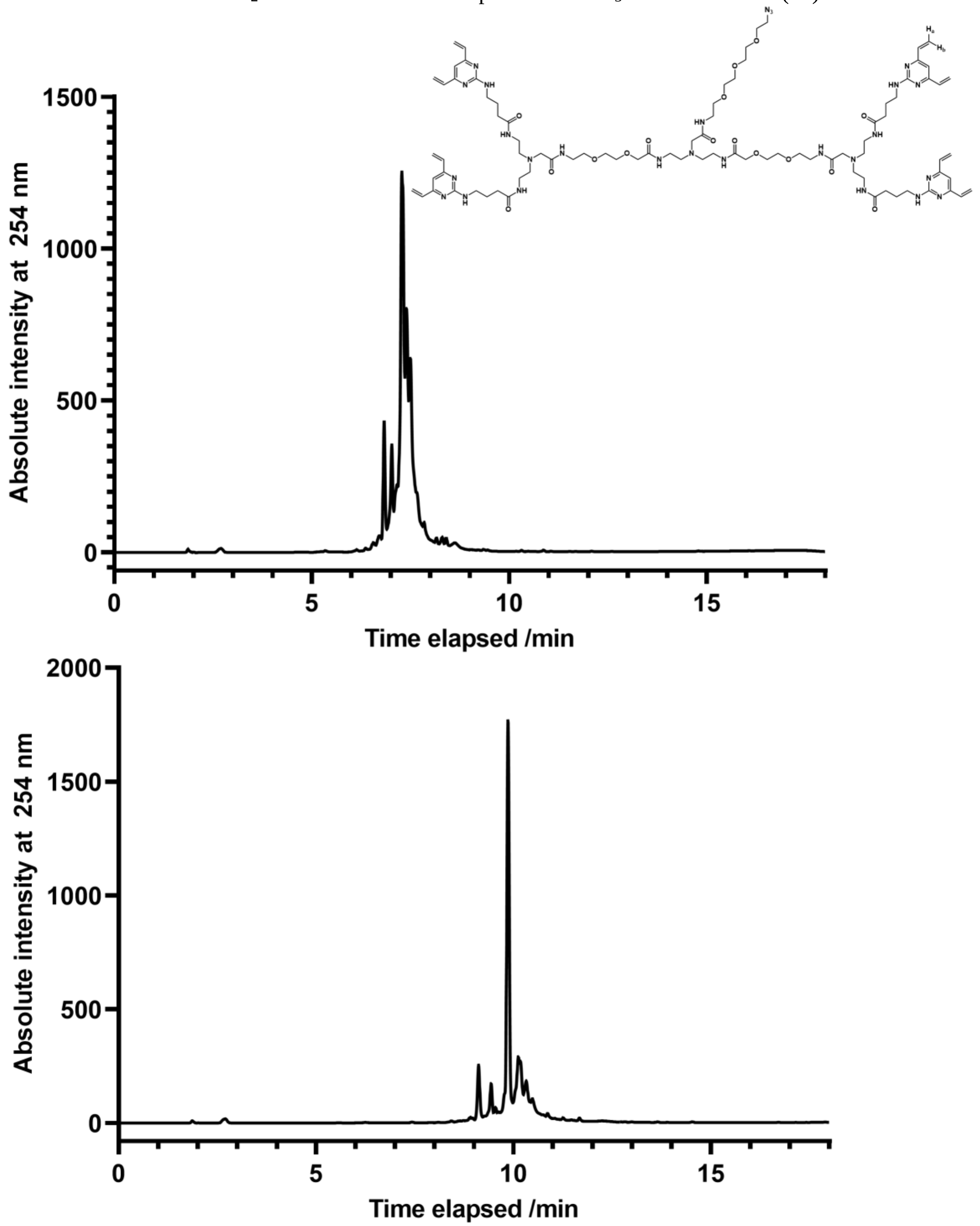

Figure S7: HPLC traces at 254 nm for compound **1b**. HPLC run with gradients of 5–95% solvent B in solvent A (top) and 20–60% solvent B in solvent A (bottom). Purity based on integration of >31%.

TetraDVP backbone with PEG_2 units outside the branch point and PEG_3 azide side chain (**1c**)

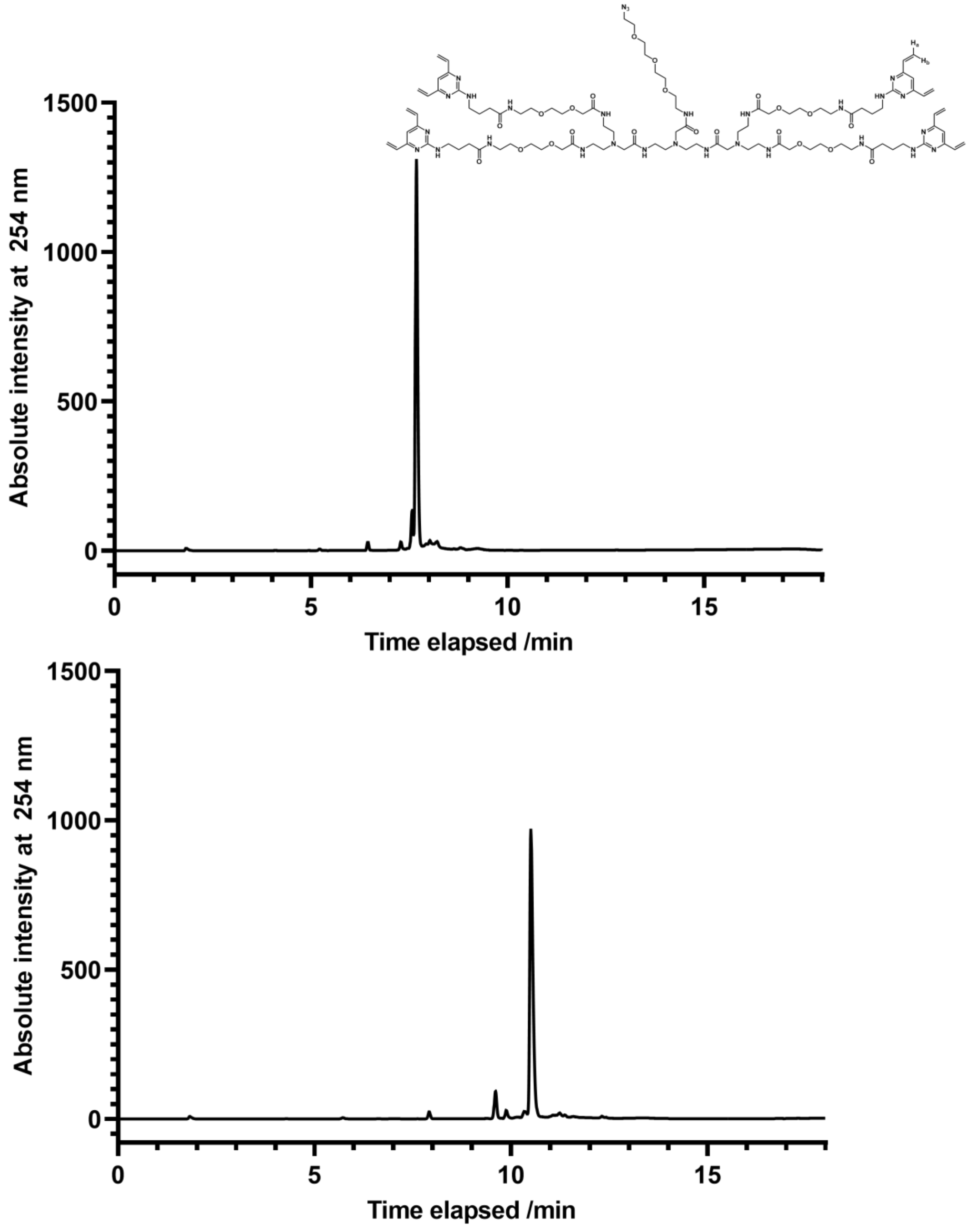

Figure S8: HPLC traces at 254 nm for compound **1c**. HPLC run with gradients of 5–95% solvent B in solvent A (top) and 20–60% solvent B in solvent A (bottom). Purity based on integration of >78%.

TetraDVP backbone with PEG_2 units inside and outside the branch point and PEG_3 azide side chain (**1d**)

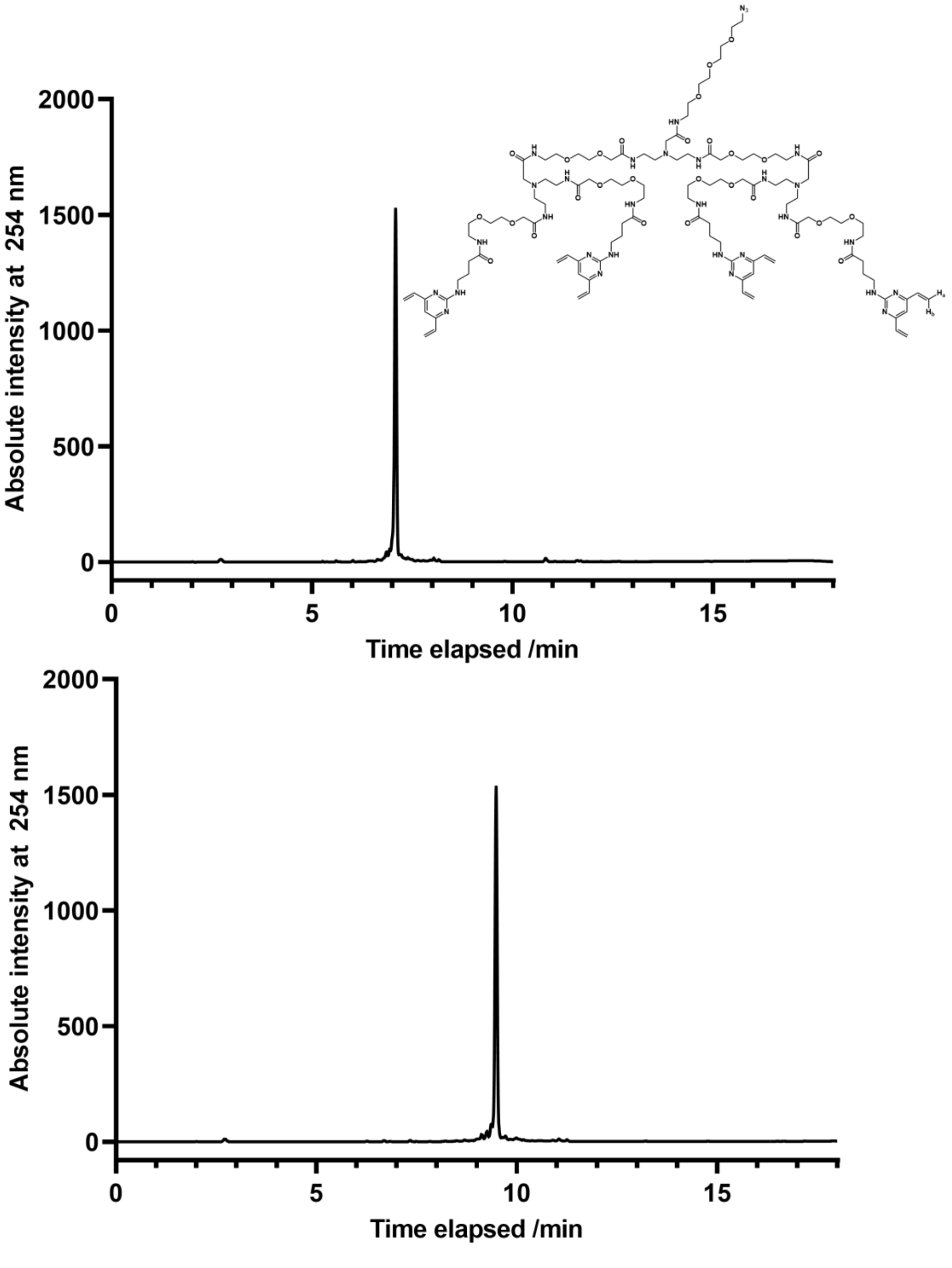

Figure S9: HPLC traces at 254 nm for compound **1d**. HPLC run with gradients of 5–95% solvent B in solvent A (top) and 20–60% solvent B in solvent A (bottom). Purity based on integration of >83%.

TetraDVP backbone with PEG_2 units inside the branch point and PEG_4 azide side chain (**2b**)

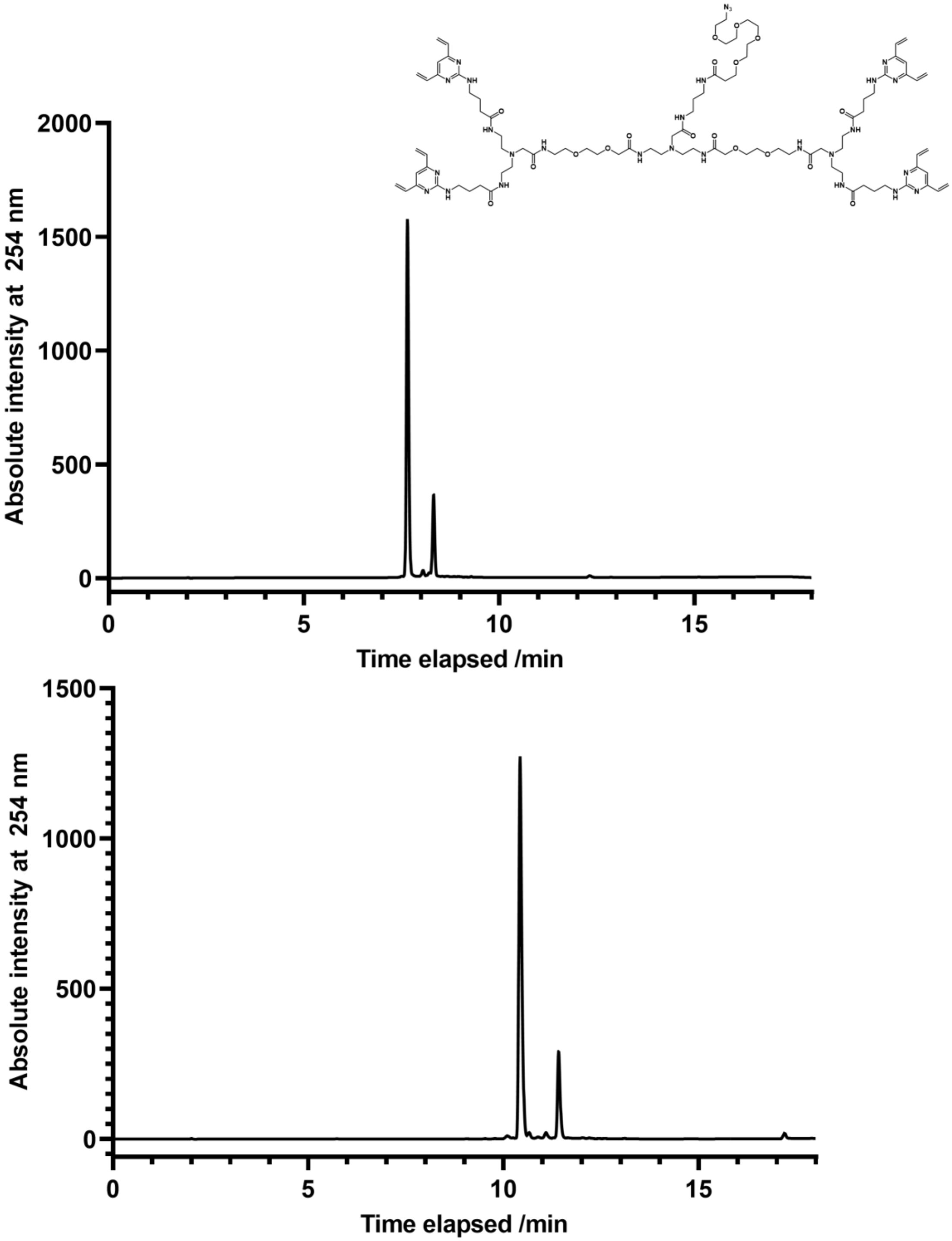

Figure S10: HPLC traces at 254 nm for compound **2b**. HPLC run with gradients of 5–95% solvent B in solvent A (top) and 20–60% solvent B in solvent A (bottom). Purity based on integration of >78%.

TetraDVP no-PEG backbone with PEG_4 azide side chain (**2a**)

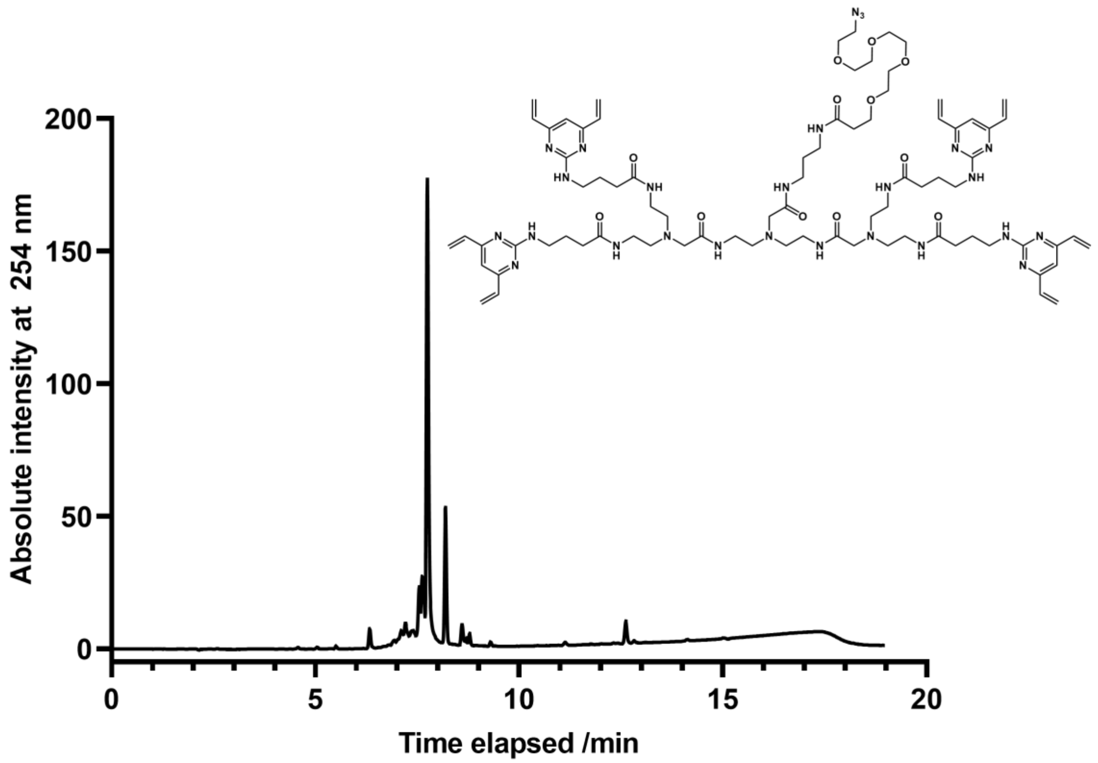

Figure S11: HPLC trace at 254 nm for compound **2a**. HPLC run with gradient of 5–95% solvent B in solvent. Purity based on integration of >51%.

TetraDVP backbone with PEG$_2$ units outside the branch point and PEG$_4$ azide side chain (**2c**)

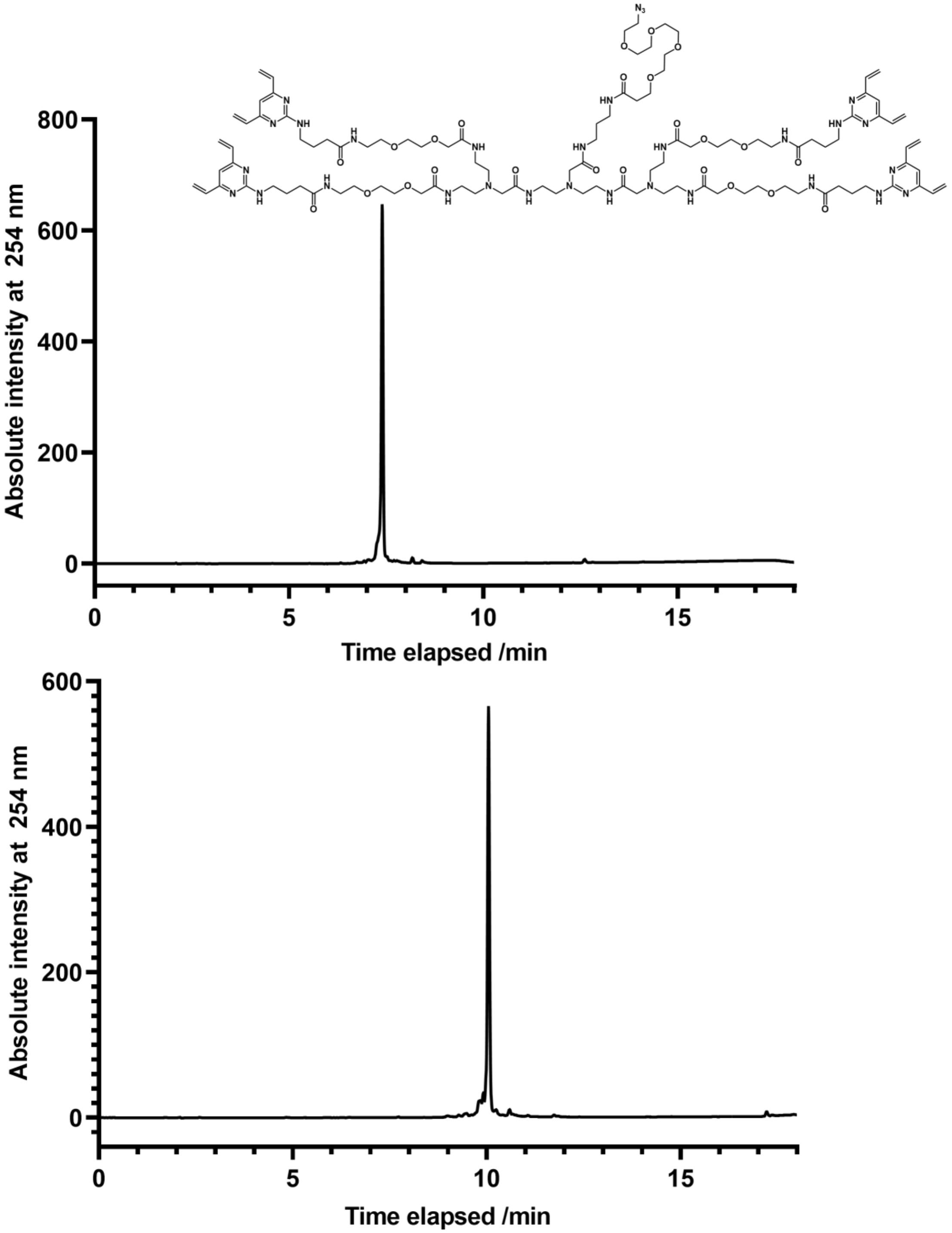

Figure S12: HPLC traces at 254 nm for compound **2c**. HPLC run with gradients of 5–95% solvent B in solvent A (top) and 20–60% solvent B in solvent A (bottom). Purity based on integration of >81%.

TetraDVP backbone with PEG$_2$ units inside and outside the branch point and PEG$_4$ azide side chain (**2d**)

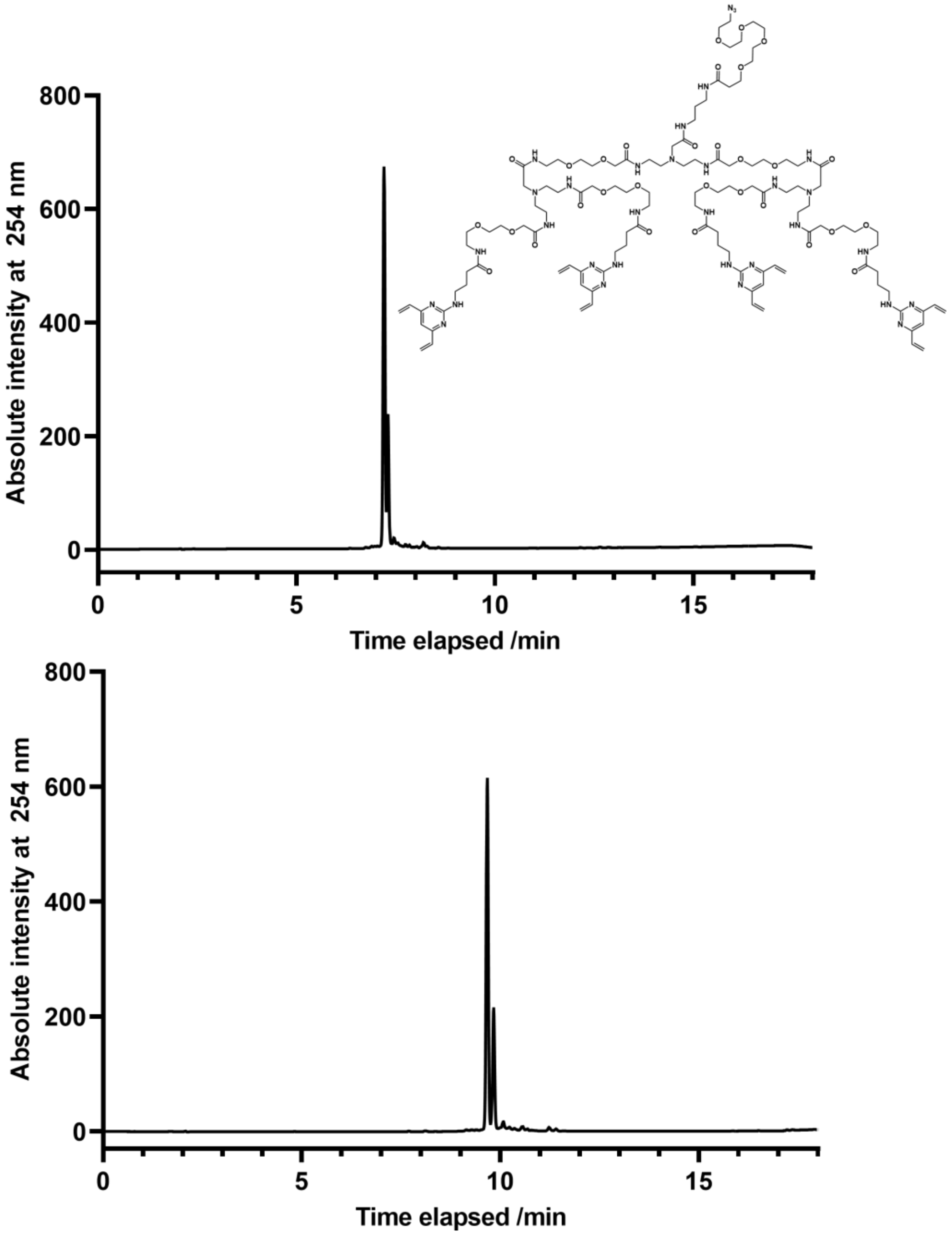

Figure S13: HPLC traces at 254 nm for compound **2d**. HPLC run with gradients of 5–95% solvent B in solvent A (top) and 20–60% solvent B in solvent A (bottom). Purity based on integration of >65%.

Fmoc-Val-Cit-PAB-MMAE (**S21**)

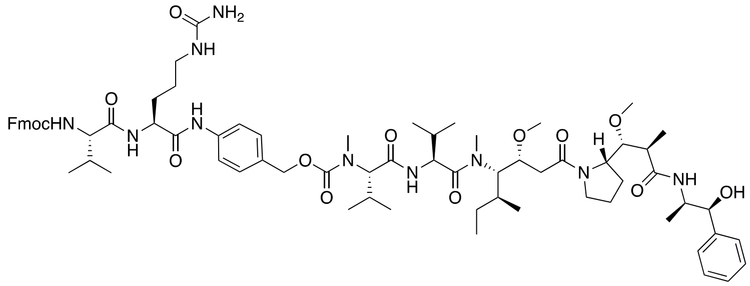

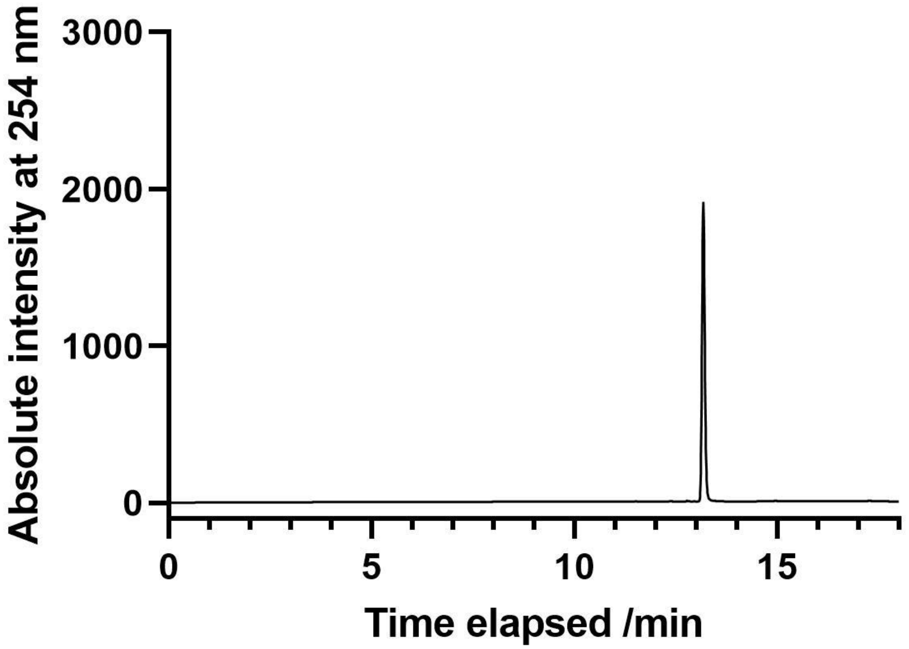

Figure S14: HPLC traces at 254 nm for compound **S21**. HPLC run with gradients of 5–95% solvent B in solvent A. Purity based on integration of >99%.

DBCO-PEG5-Val-Cit-PAB-MMAE (**5**)

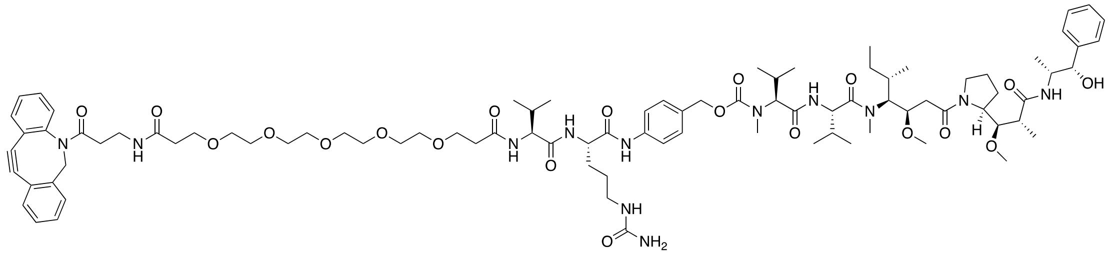

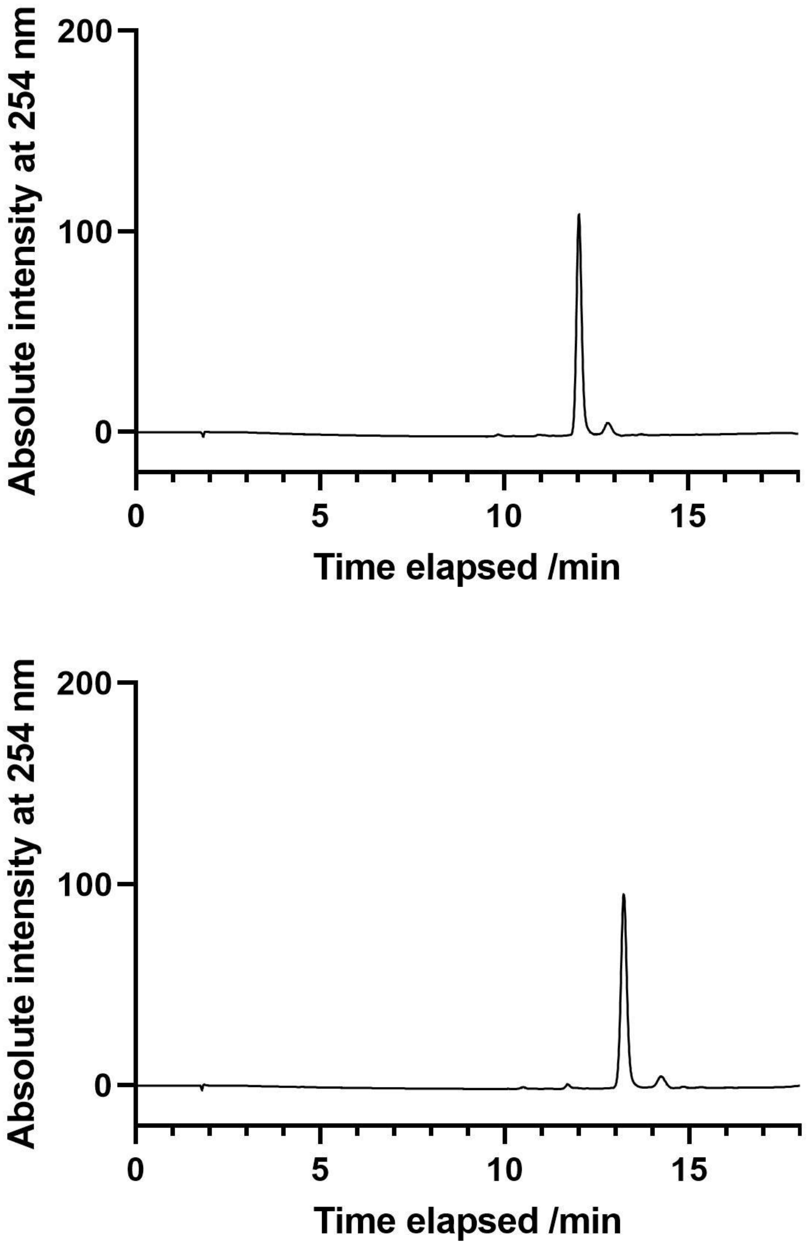

Figure S15: HPLC traces at 254 nm for compound **5**. HPLC run with gradients of 5–95% solvent B in solvent A (top) and 10–80% solvent B in solvent A (bottom). Purity based on integration of 92%.

10 Protein mass spectra

ALC **3a**

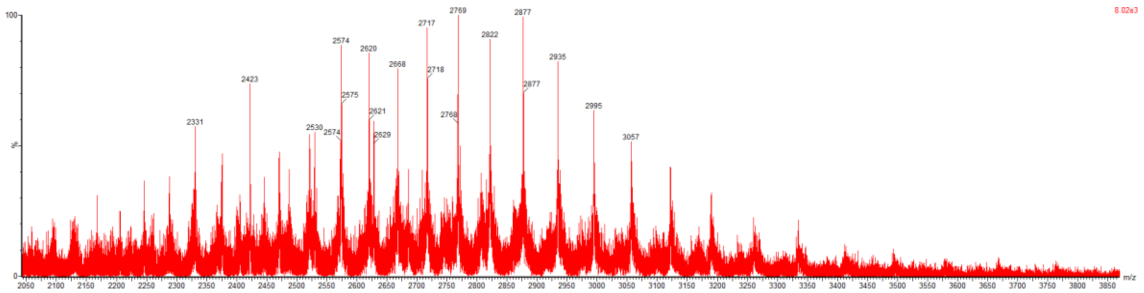

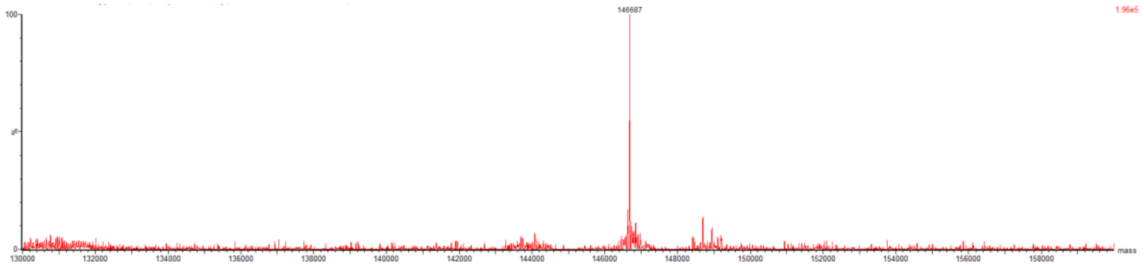

Figure S16: Protein mass spectrum (top) and deconvolution (bottom) of ALC **3a**

ALC **3b**

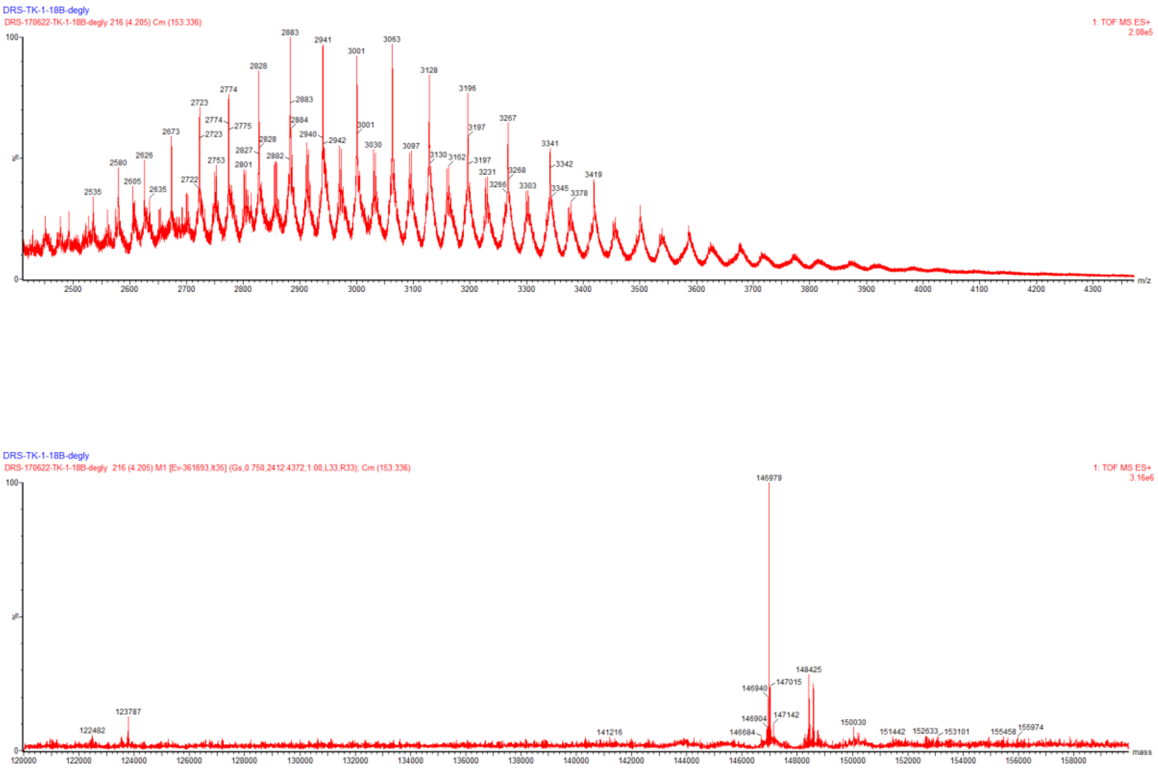

Figure S17: Protein mass spectrum (top) and deconvolution (bottom) of ALC **3b**

ALC **3c**

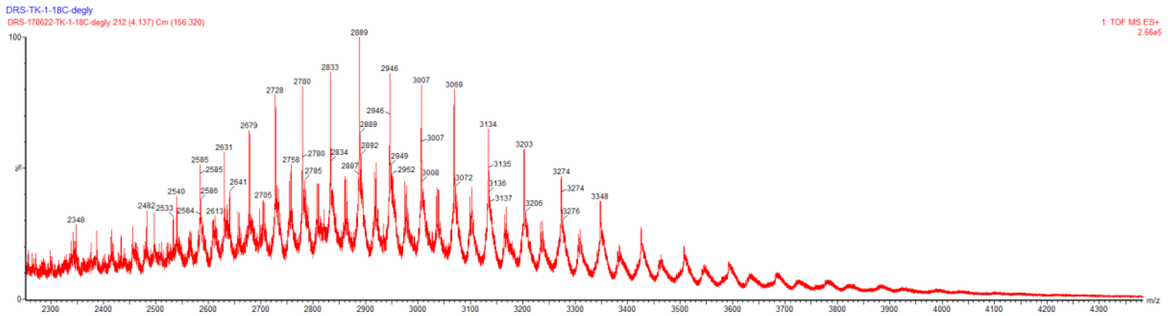

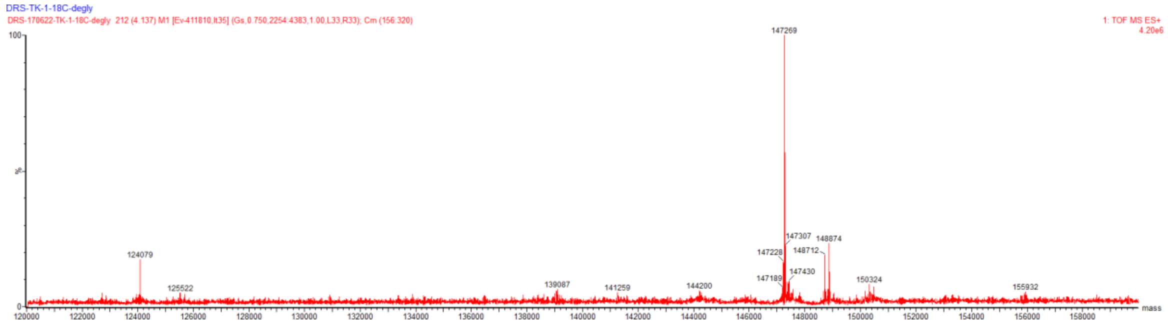

Figure S18: Protein mass spectrum (top) and deconvolution (bottom) of ALC **3c**

ALC **3d**

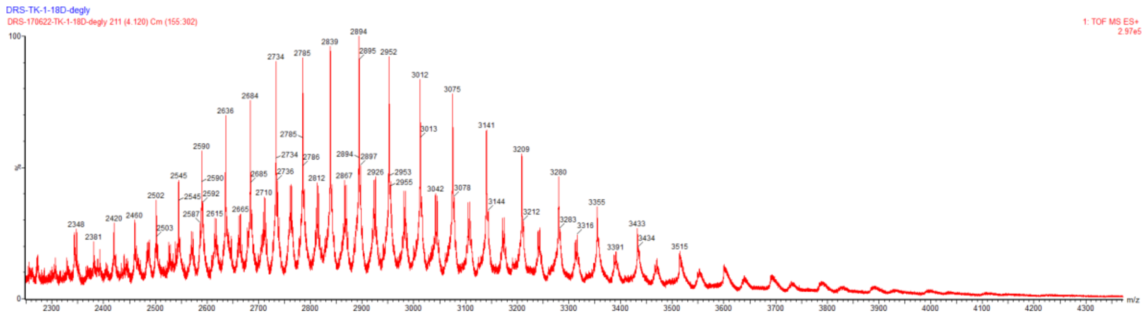

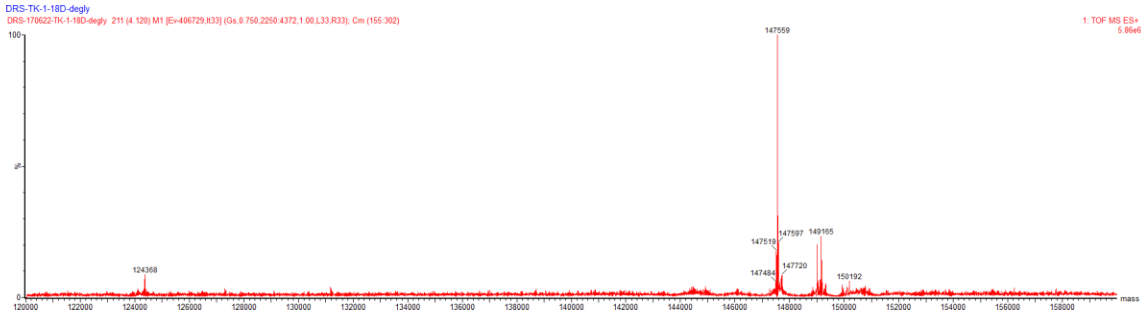

Figure S19: Protein mass spectrum (top) and deconvolution (bottom) of ALC **3d**

ALC **4a**

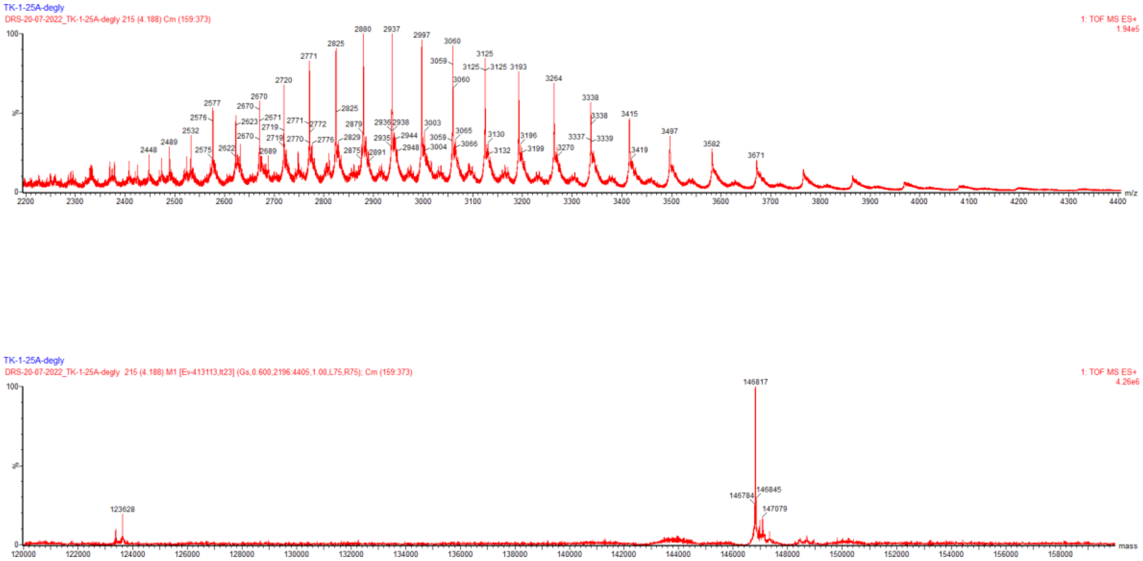

Figure S20: Protein mass spectrum (top) and deconvolution (bottom) of ALC **4a**

ALC **4b**

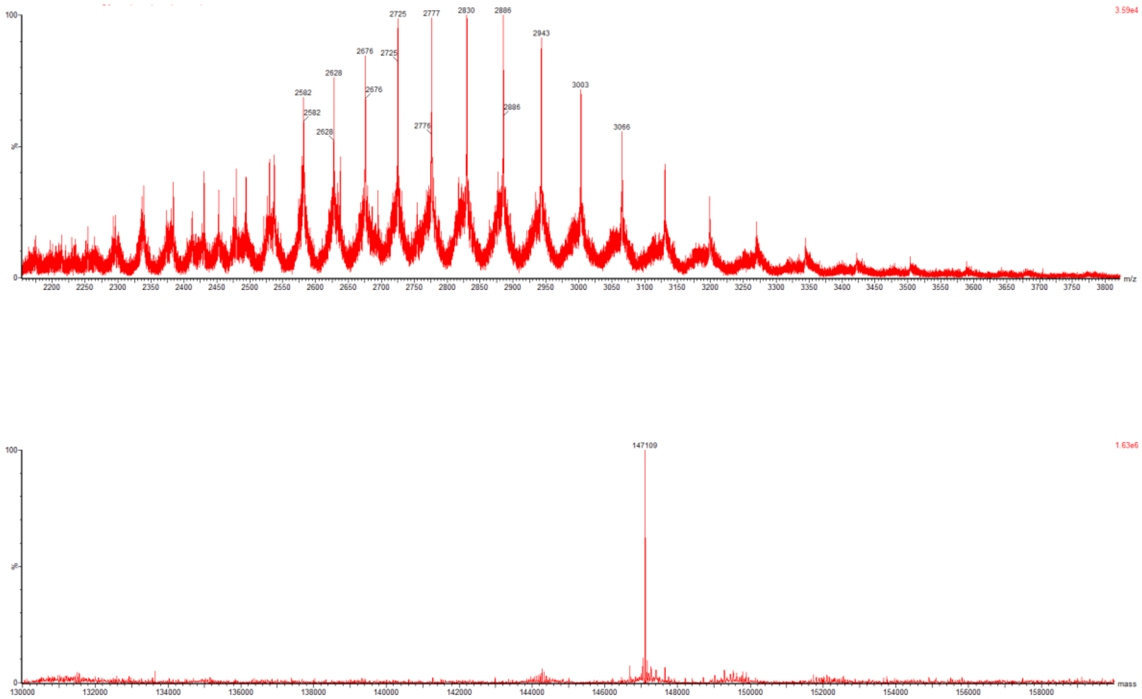

Figure S21: Protein mass spectrum (top) and deconvolution (bottom) of ALC **4b**

ALC **4c**

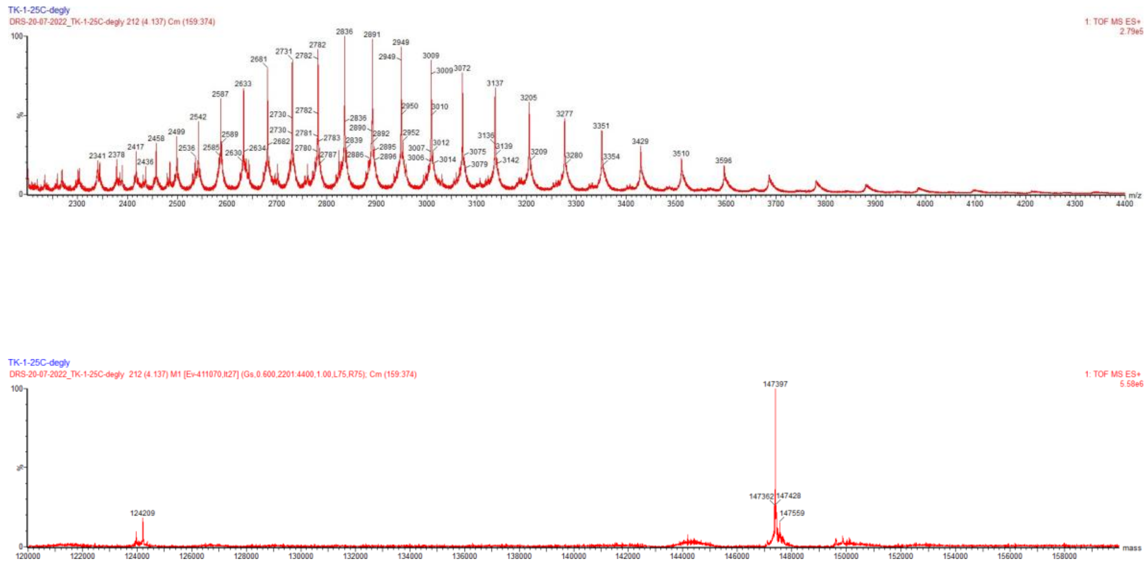

Figure S22: Protein mass spectrum (top) and deconvolution (bottom) of ALC **4c**

ALC **4d**

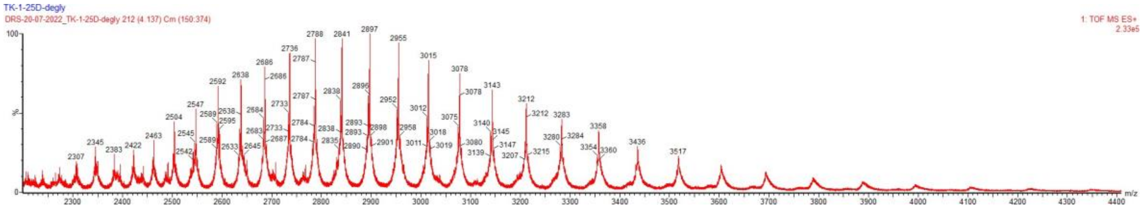

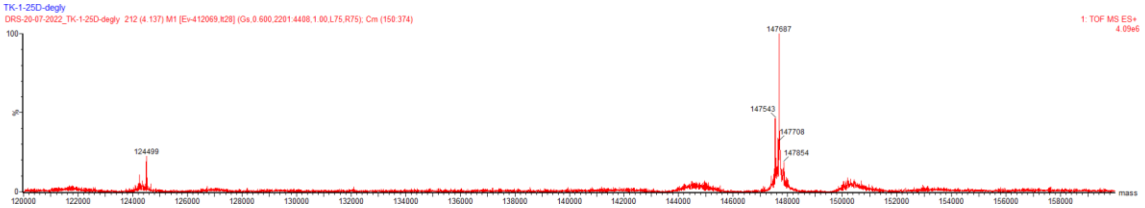

Figure S23: Protein mass spectrum (top) and deconvolution (bottom) of ALC **4d**

ADC **6a**

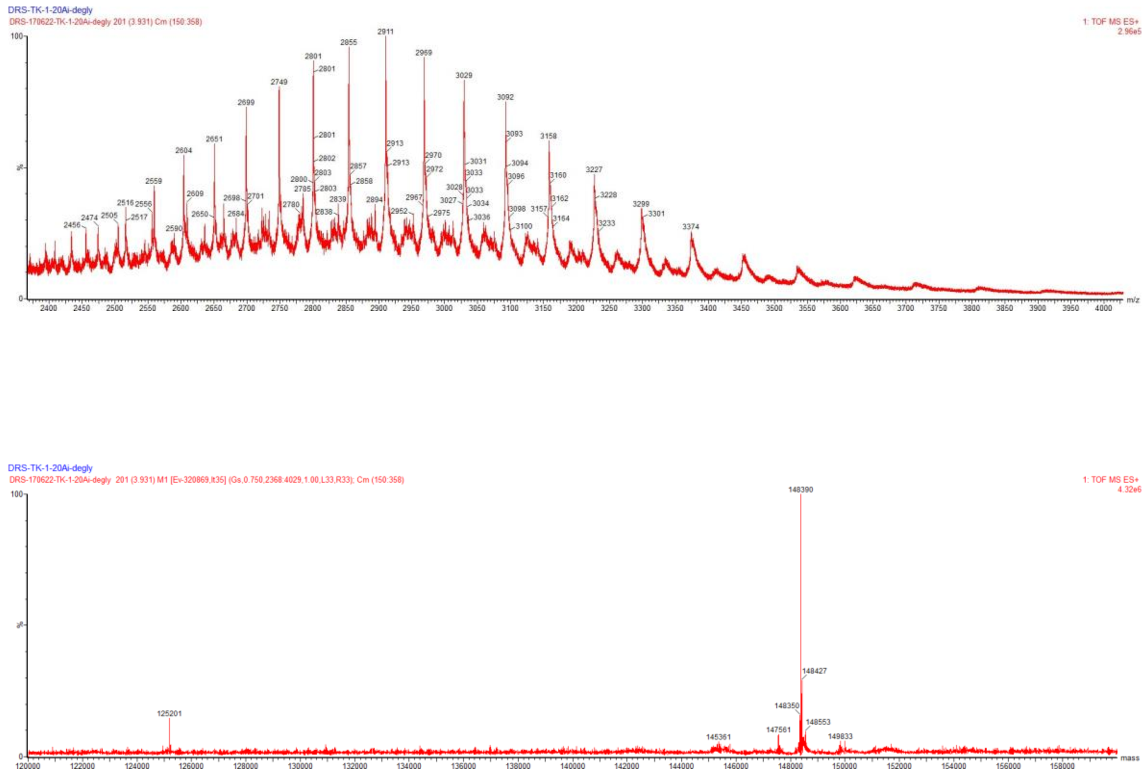

Figure S24: Protein mass spectrum (top) and deconvolution (bottom) of ADC **6a**

ADC **6b**

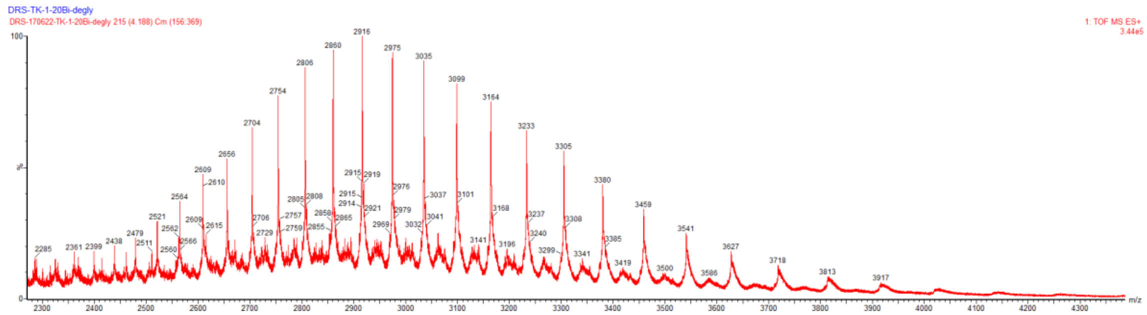

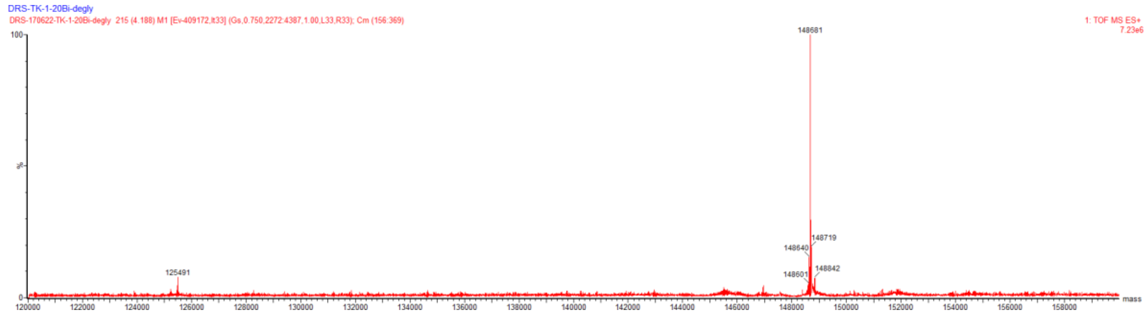

Figure S25: Protein mass spectrum (top) and deconvolution (bottom) of ADC **6b**

ADC **6c**

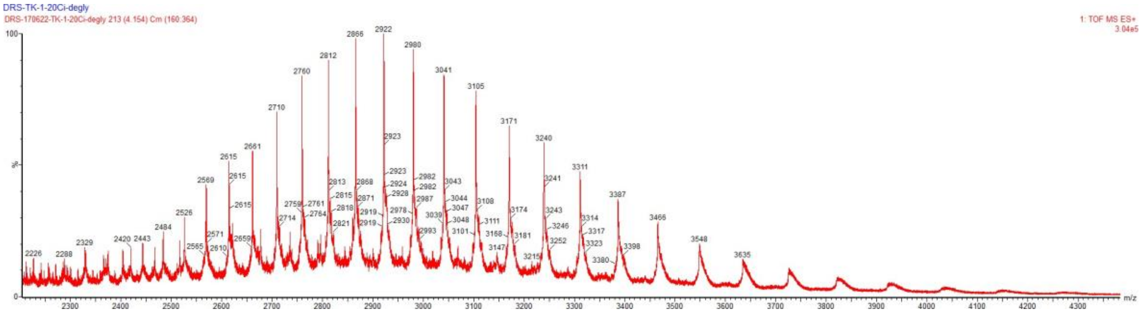

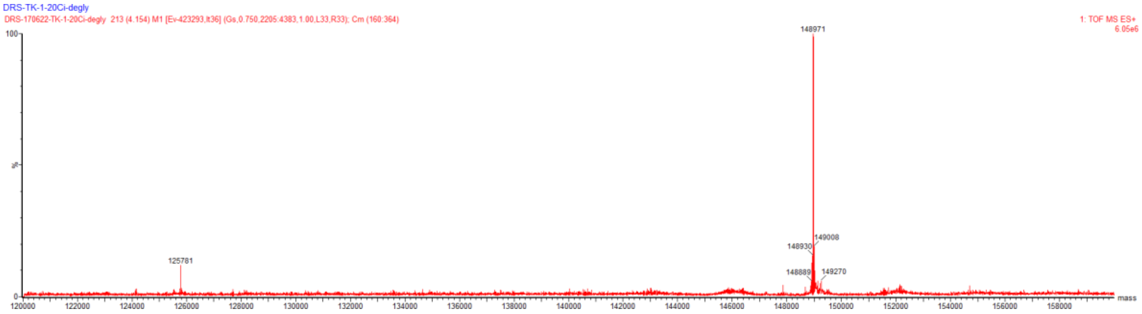

Figure S26: Protein mass spectrum (top) and deconvolution (bottom) of ADC **6c**

ADC **6d**

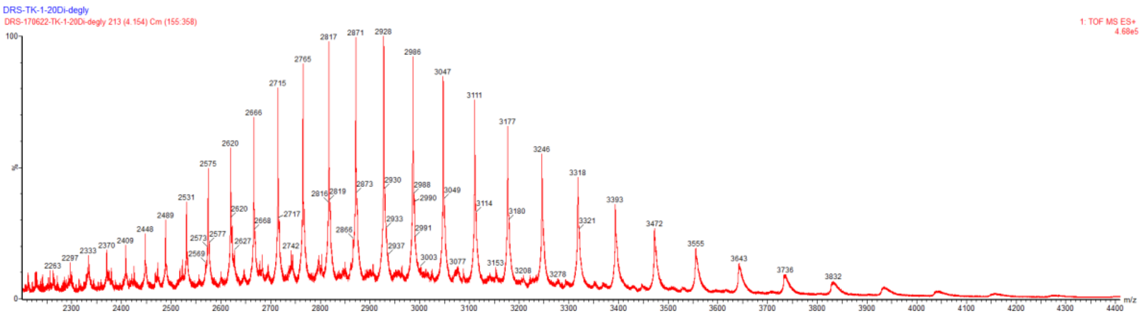

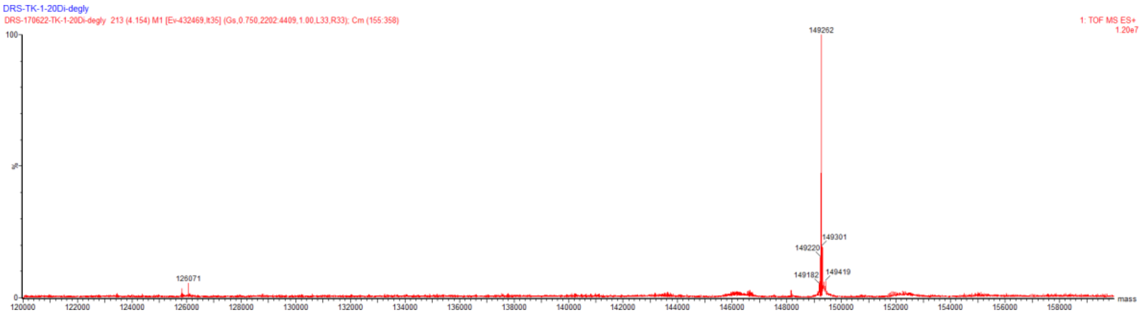

Figure S27: Protein mass spectrum (top) and deconvolution (bottom) of ADC **6d**

ADC **7a**

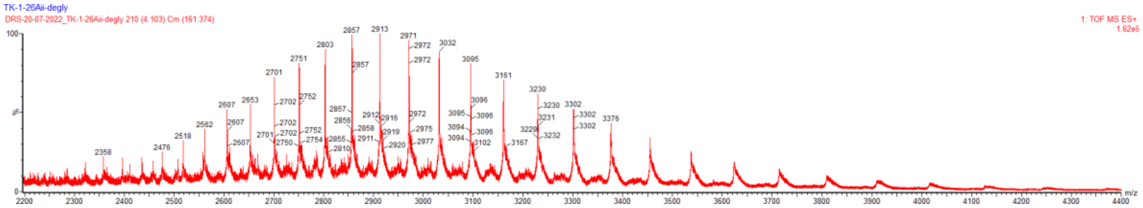

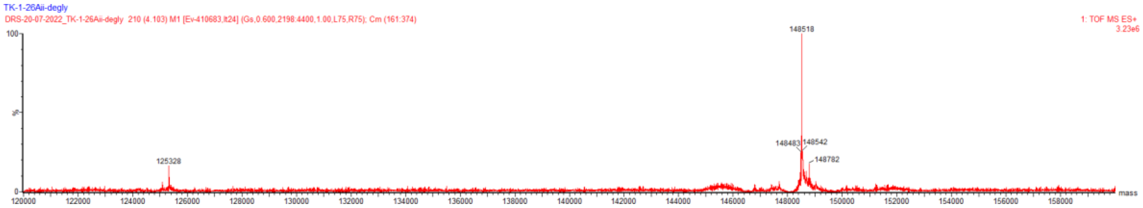

Figure S28: Protein mass spectrum (top) and deconvolution (bottom) of ADC **7a**

ADC **7b**

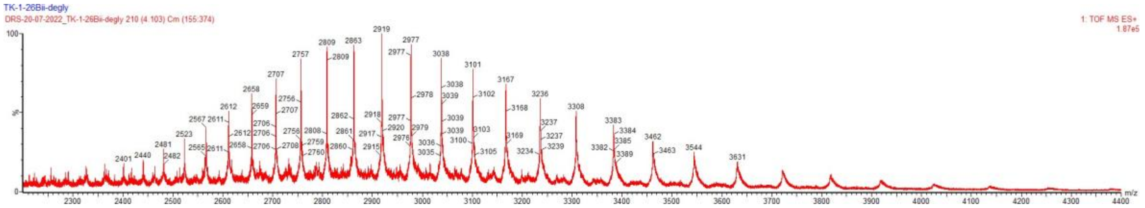

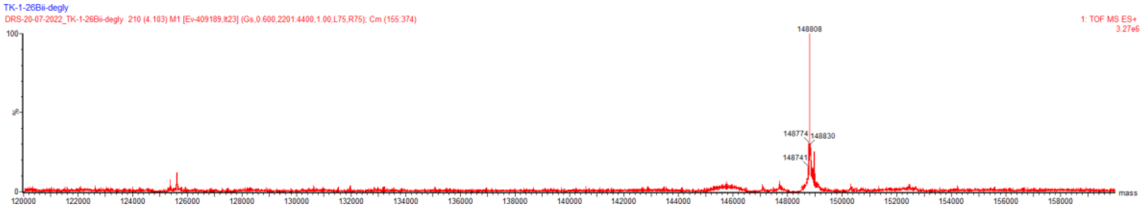

Figure S29: Protein mass spectrum (top) and deconvolution (bottom) of ADC **7b**

ADC **7c**

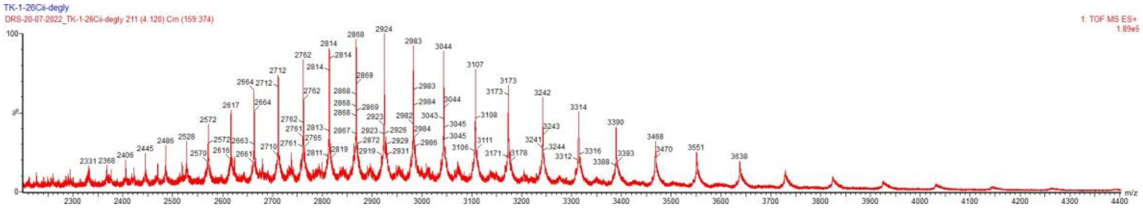

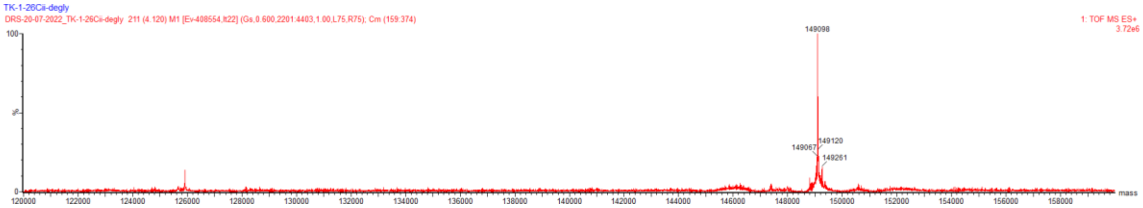

Figure S30: Protein mass spectrum (top) and deconvolution (bottom) of ADC **7c**

ADC **7d**

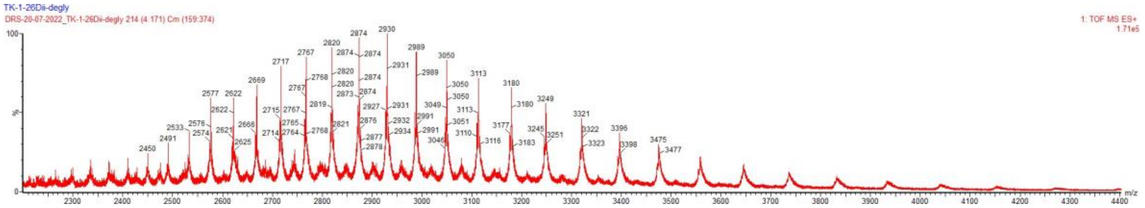

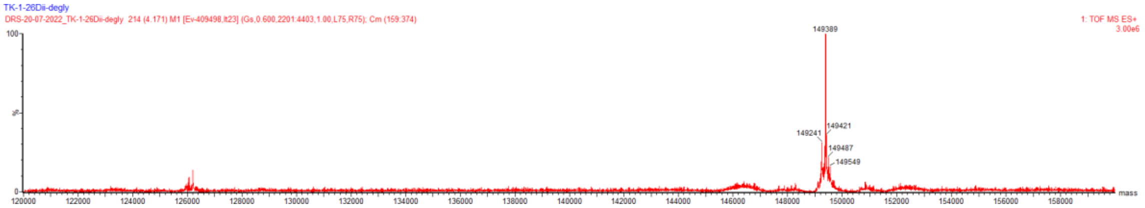

Figure S31: Protein mass spectrum (top) and deconvolution (bottom) of ADC **7d**

ADC **9a**

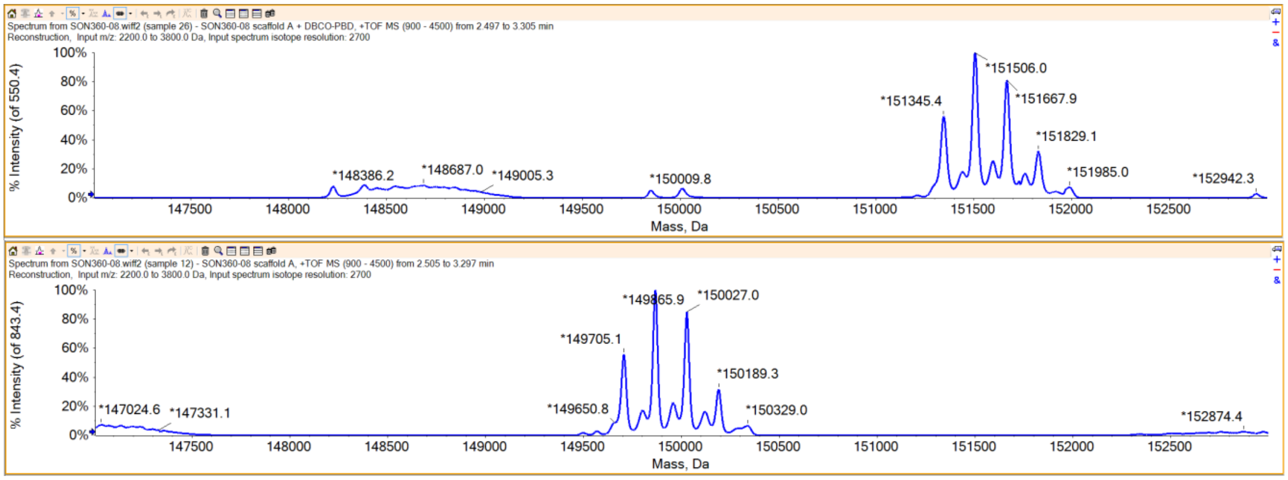

Figure S32: Intact mass spectrum of ADC **9a** (top) and ALC **4a** (bottom). $\Delta m/z_{(ADC\text{-}ALC)}$ = 1640 Da (calc. 1632 Da).

ADC **9b**

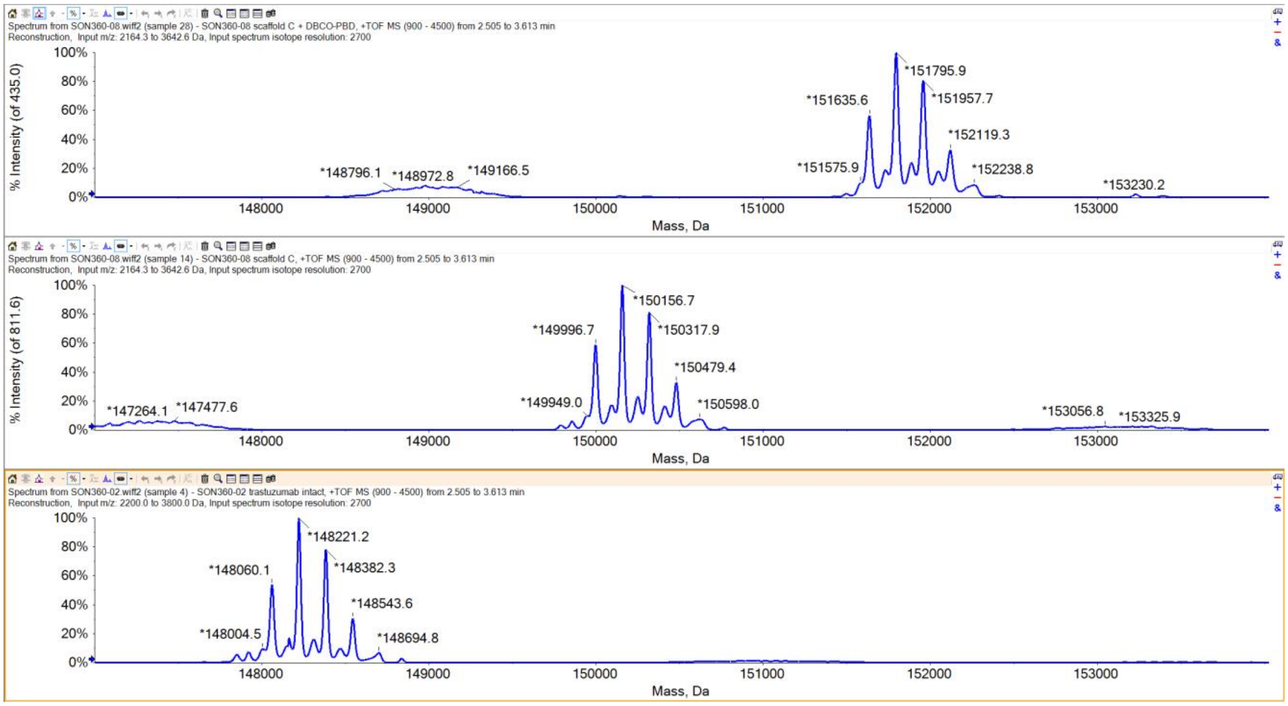

Figure S33: Intact mass spectrum of ADC **9b** (top), ALC **4b** (middle) and native trastuzumab (bottom). $\Delta m/z_{(ADC-ALC)}$ = 1639 Da (calc. 1632 Da).

ADC **9c**

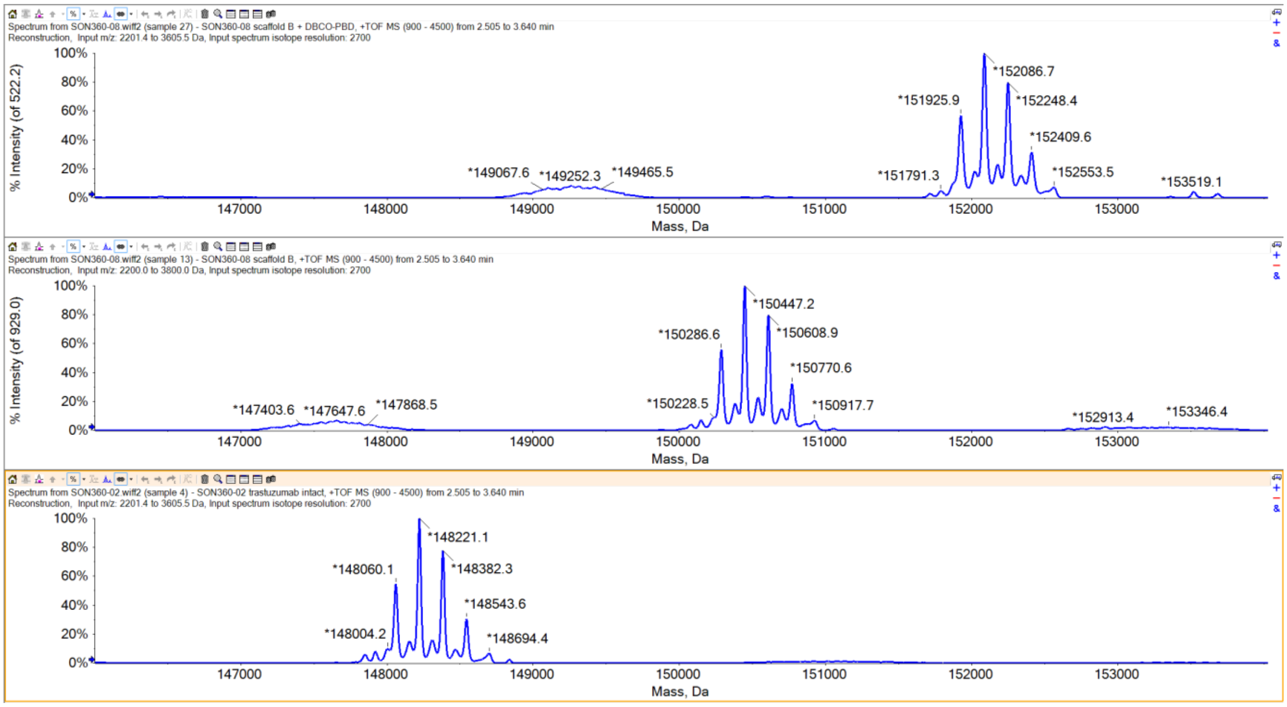

Figure S34: Intact mass spectrum of ADC **9c** (top), ALC **4c** (middle) and native trastuzumab (bottom). $\Delta m/z_{(ADC\text{-}ALC)}$ = 1640 Da (calc. 1632 Da).

ADC **9d**

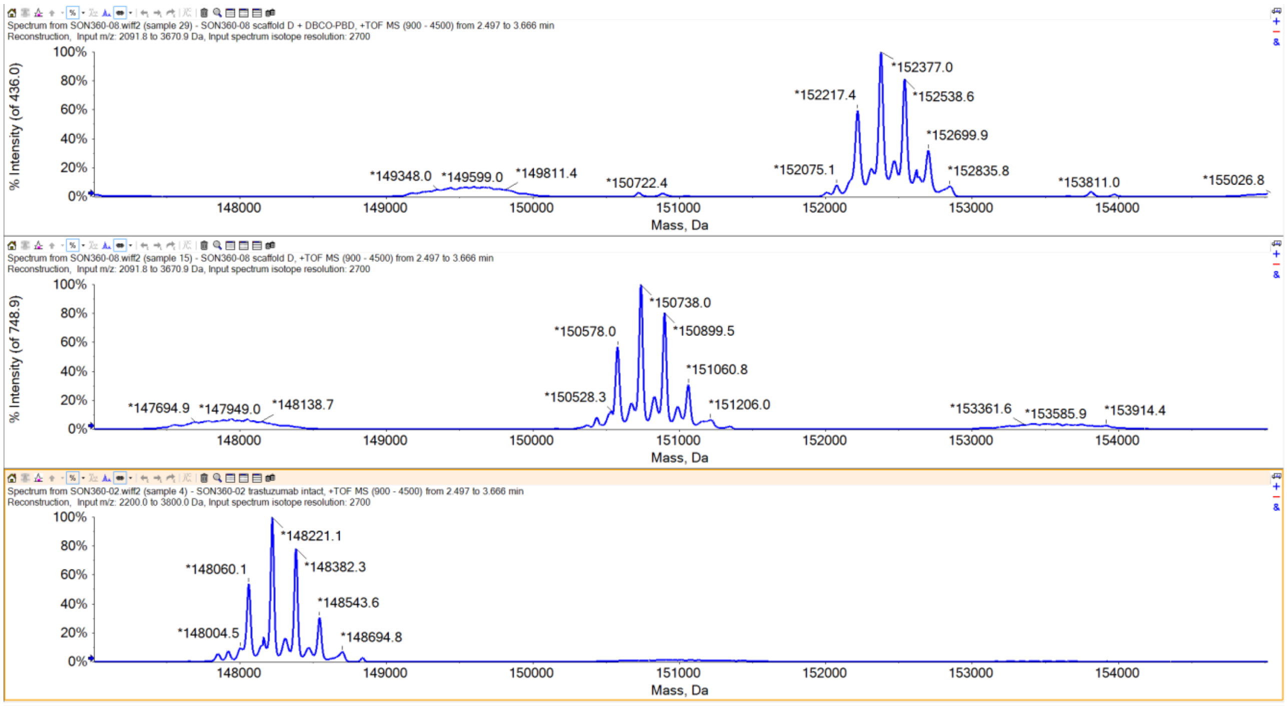

Figure S35: Intact mass spectrum of ADC **9d** (top), ALC **4d** (middle) and native trastuzumab (bottom). $\Delta m/z_{(ADC\text{-}ALC)}$ = 1639 Da (calc. 1632 Da).

11 Size-exclusion chromatography (SEC) and hydrophobic interaction chromatography (HIC)

ALC **3a**

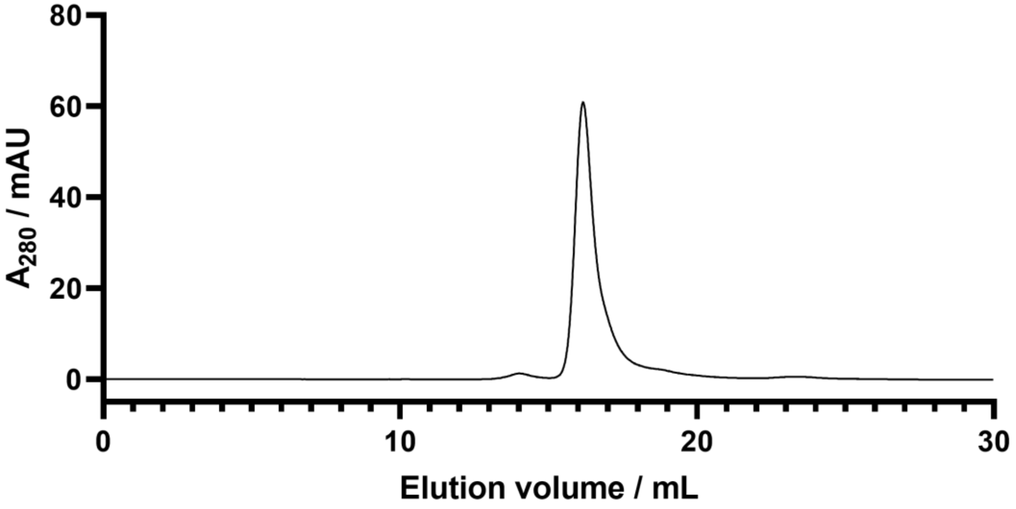

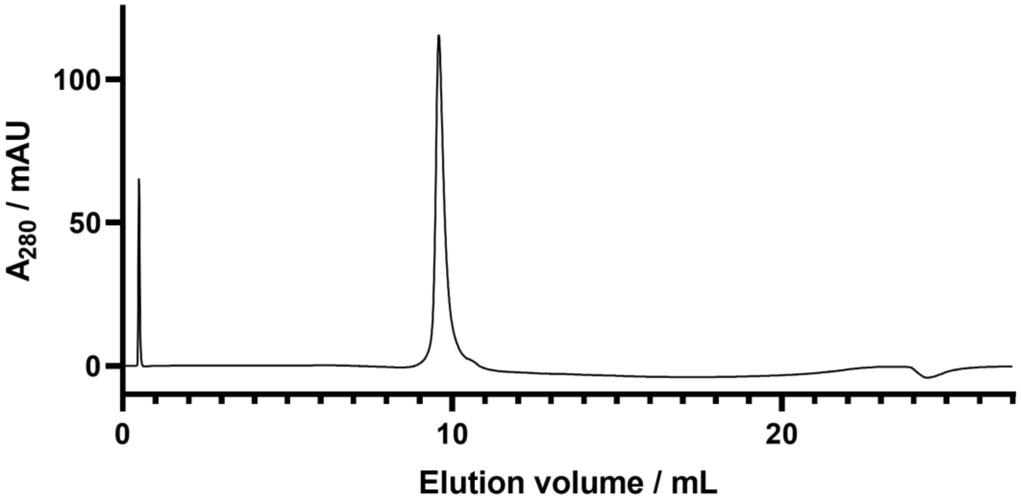

Figure S36: SEC (top) and HIC (bottom) traces for the reaction of **1a** with trastuzumab to give ALC **3a**

ALC **3b**

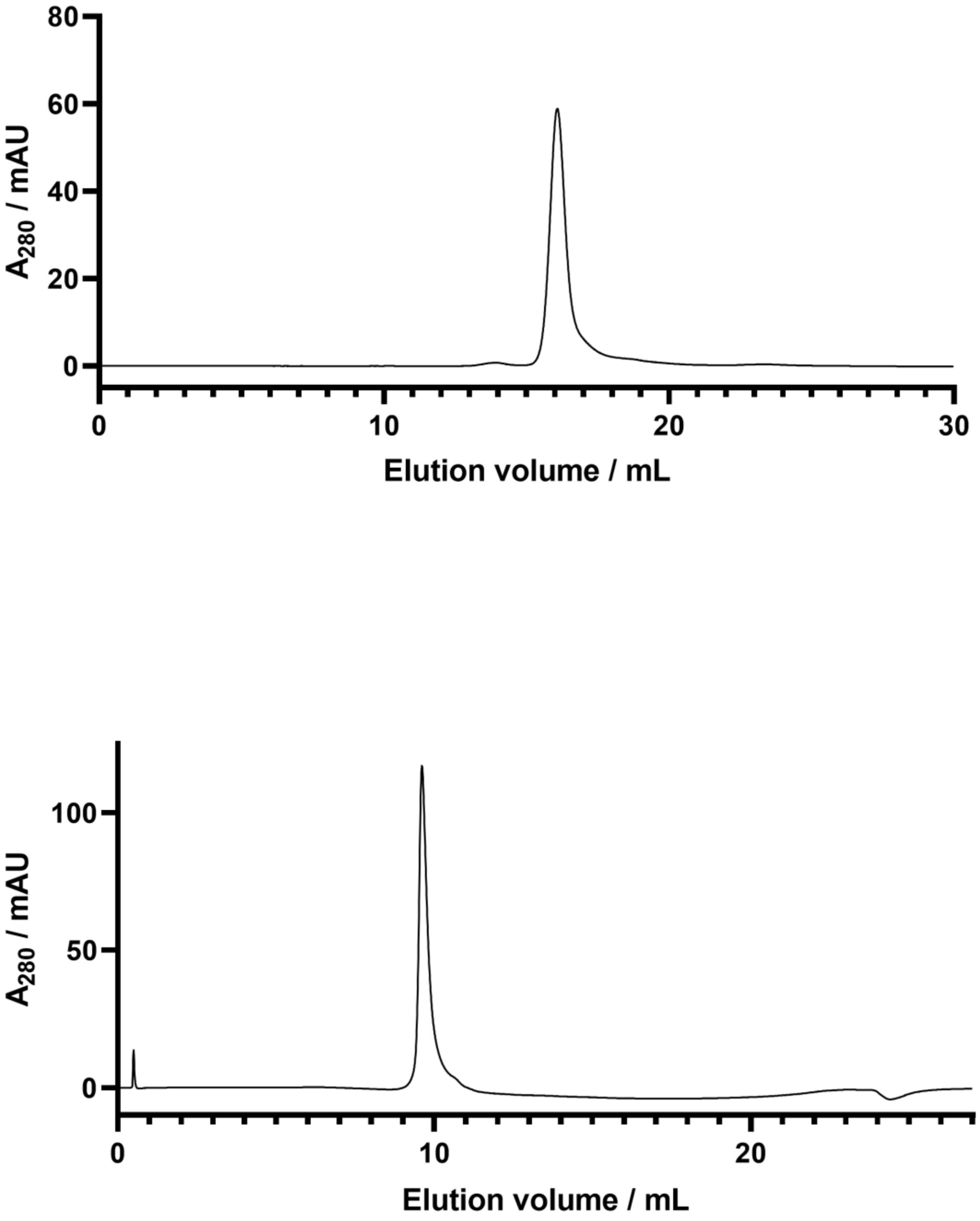

Figure S37: SEC (top) and HIC (bottom) traces for the reaction of **1b** with trastuzumab to give ALC **3b**

ALC **3c**

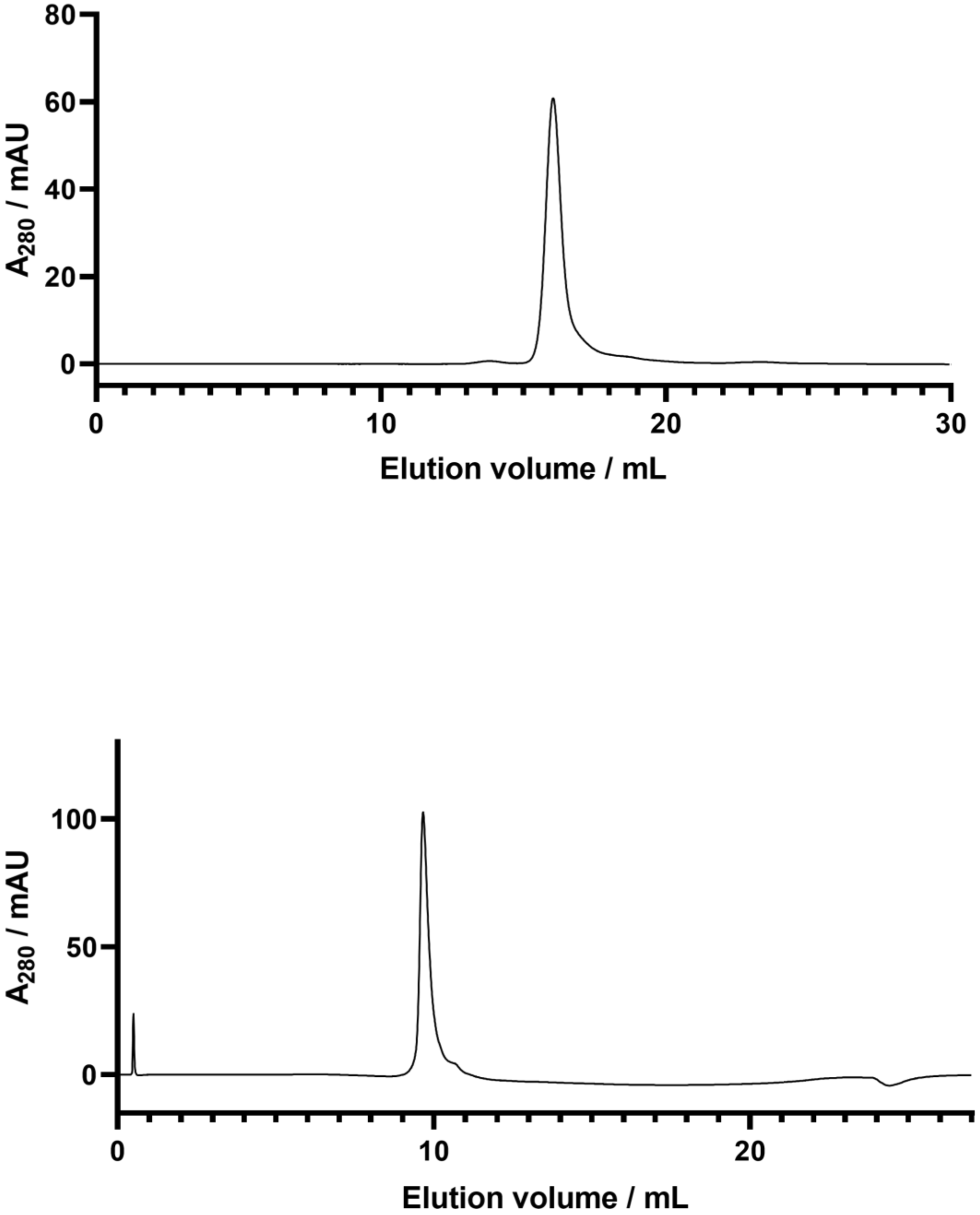

Figure S38: SEC (top) and HIC (bottom) traces for the reaction of **1c** with trastuzumab to give ALC **3c**

ALC **3d**

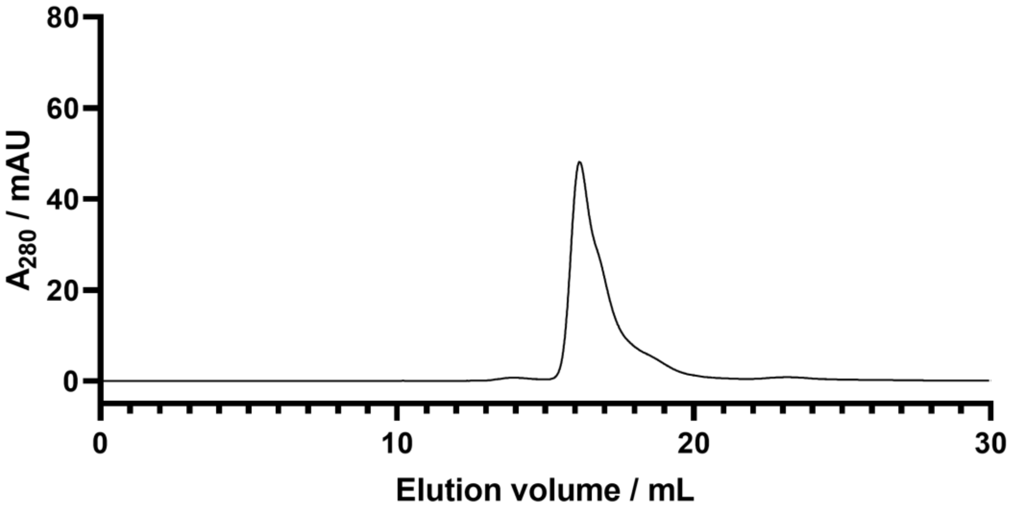

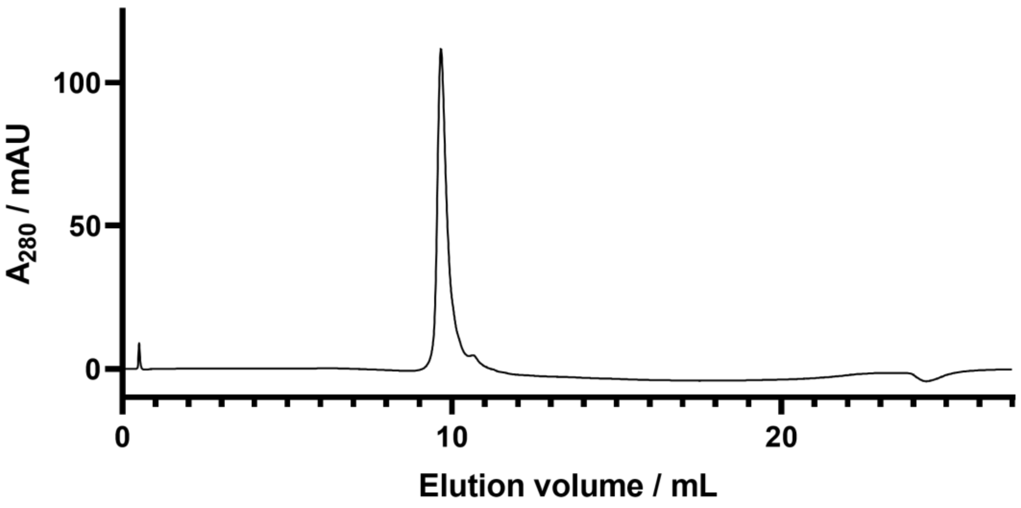

Figure S39: SEC (top) and HIC (bottom) traces for the reaction of **1d** with trastuzumab to give ALC **3d**

ALC **4a**

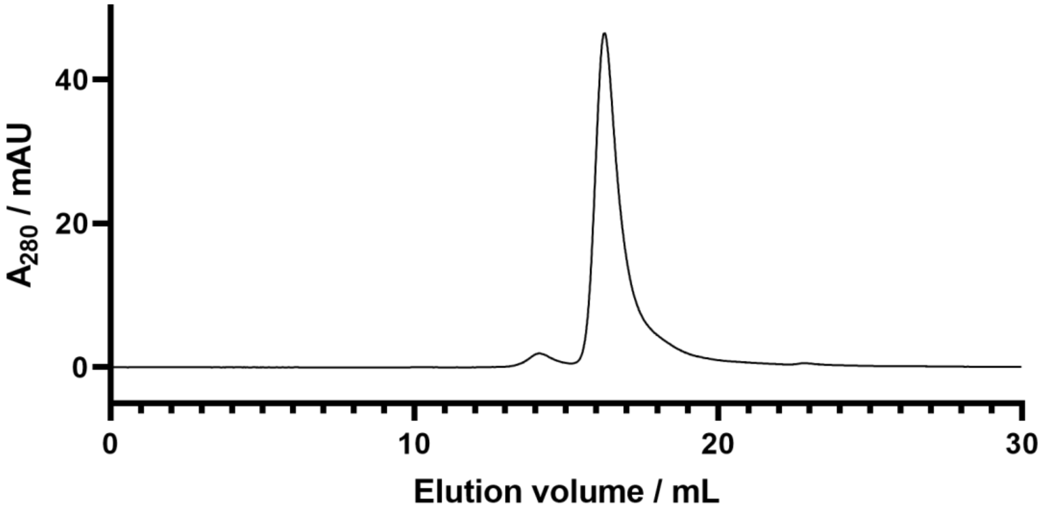

Figure S40: SEC trace for the reaction of **2a** with trastuzumab to give ALC **4a**

ALC **4b**

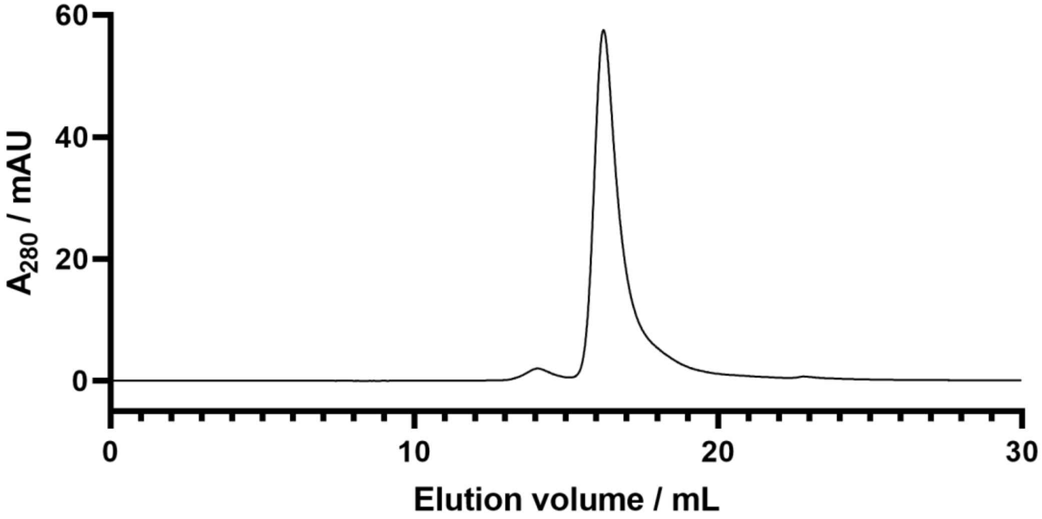

Figure S41: SEC trace for the reaction of **2b** with trastuzumab to give ALC **4b**

ALC **4c**

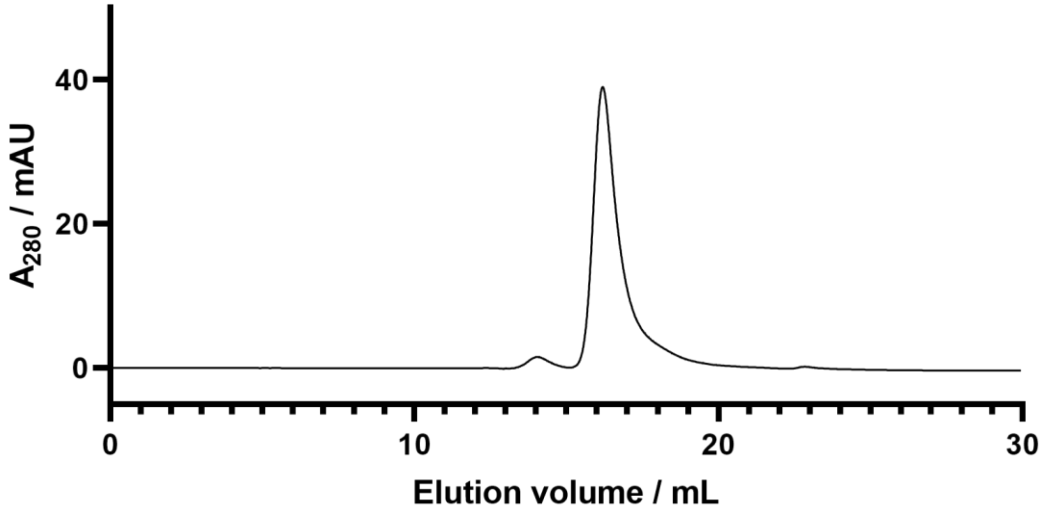

Figure S42: SEC trace for the reaction of **2c** with trastuzumab to give ALC **4c**

ALC **4d**

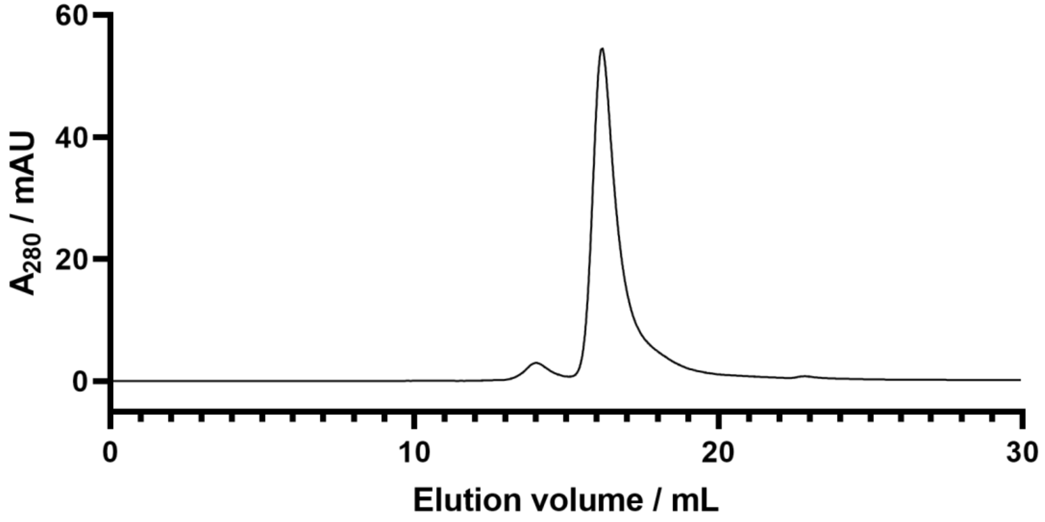

Figure S43: SEC trace for the reaction of **2d** with trastuzumab to give ALC **4d**

ADC **6a**

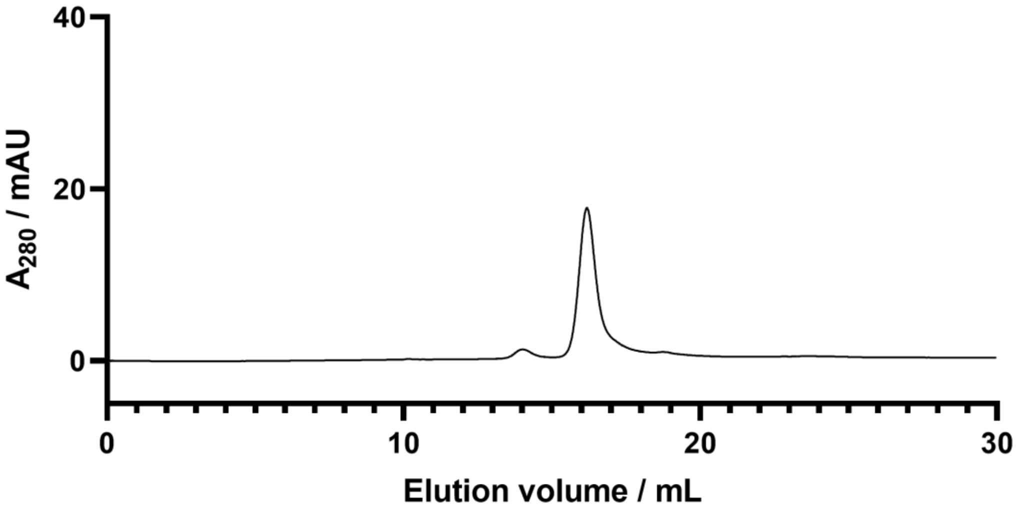

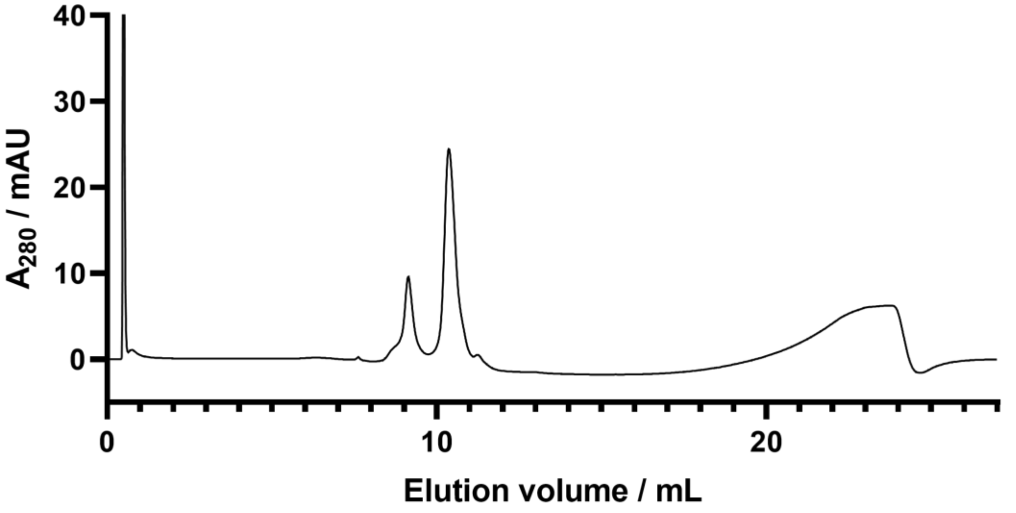

Figure S44: SEC (top) and HIC (bottom) traces of conjugate ADC **6a** from the reaction of ALC **3a** with DBCO-PEG$_5$-Val-Cit-MMAE (**5**). Method: DBCO reagent (10 equiv.), 4 h.

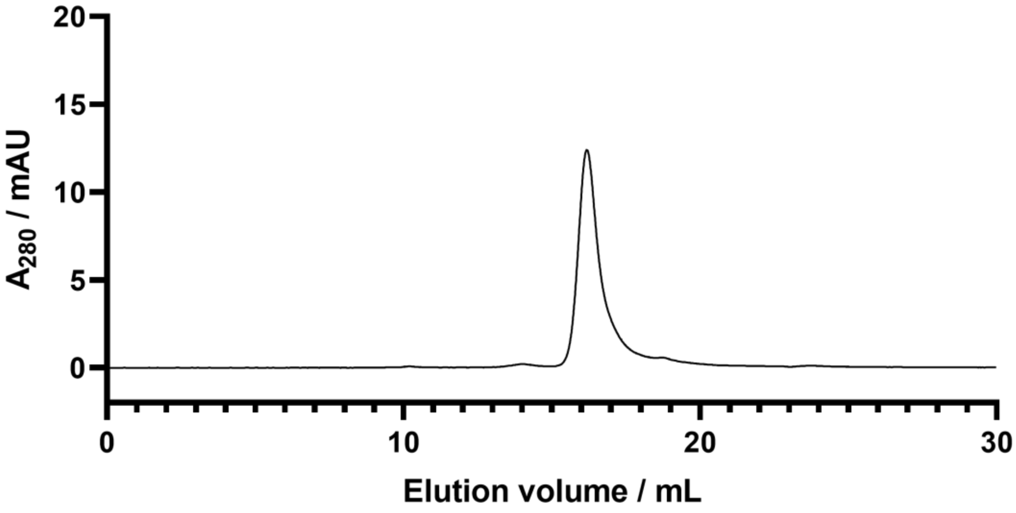

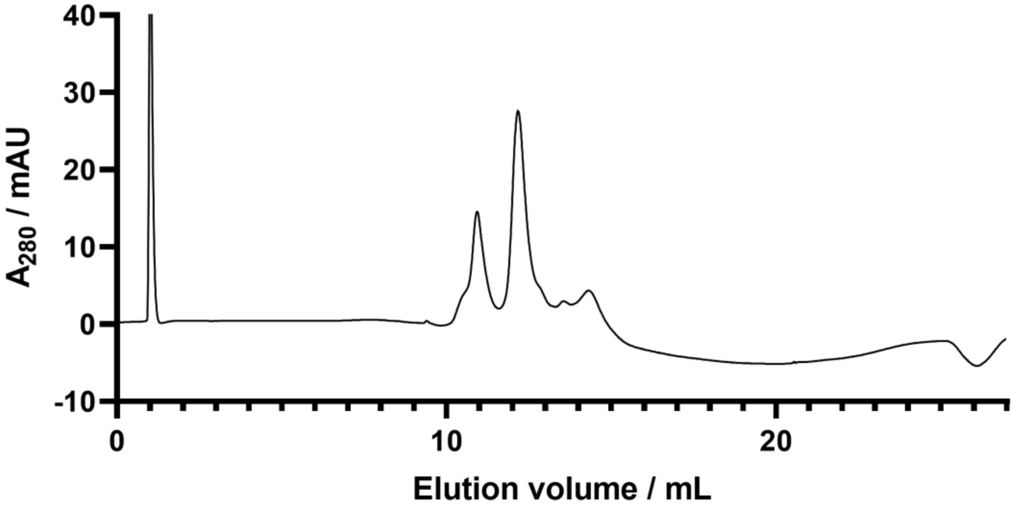

Figure S45: SEC (top) and HIC (bottom) traces of conjugate ADC **6a** from the reaction of ALC **3a** with DBCO-PEG$_5$-Val-Cit-MMAE (**5**). Method: DBCO reagent (100 equiv.), 24 h.

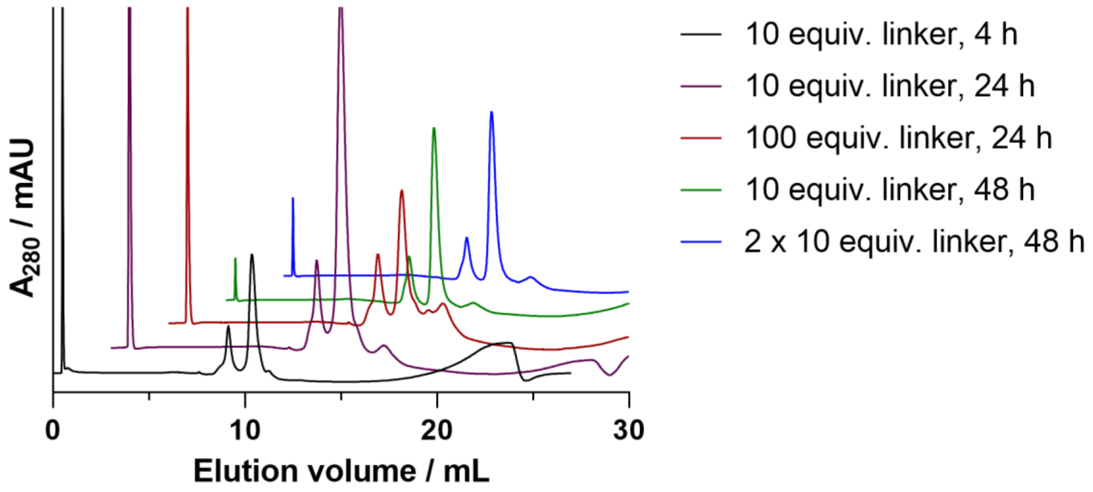

Figure S46: HIC traces of conjugate ADC **6a** from the reaction of ALC **3a** with DBCO-PEG$_5$-Val-Cit-MMAE (**5**). Method A: DBCO reagent (10 equiv.), 4 h; Method B: DBCO reagent (10 equiv.), 24 h; Method C: DBCO reagent (100 equiv.), 24 h; Method D: DBCO reagent (10 equiv.), 48 h; Method E: DBCO reagent (10 equiv.), 24 h then DBCO reagent (10 equiv.), 24 h.

ADC **6b**

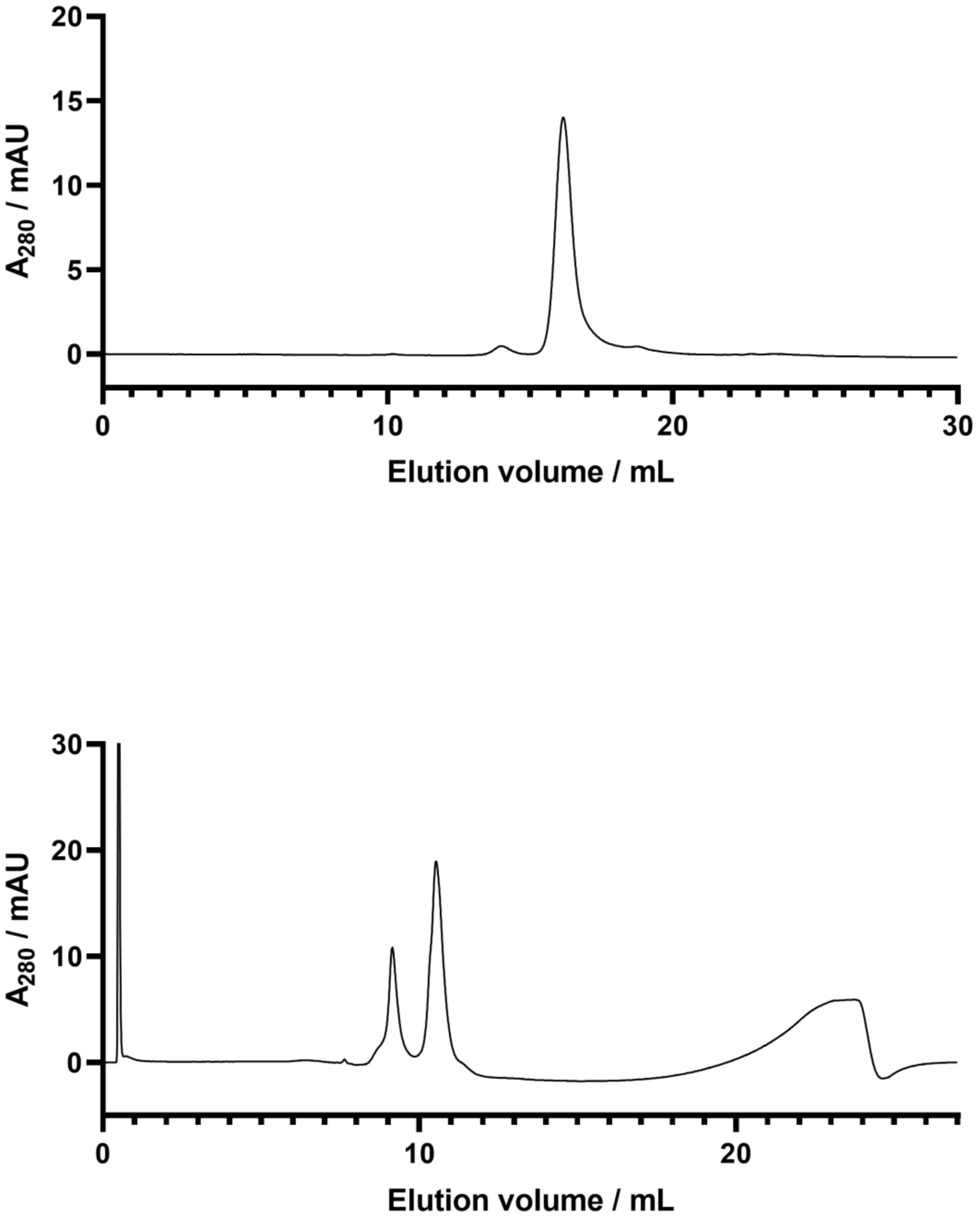

Figure S47: SEC (top) and HIC (bottom) traces of conjugate ADC **6b** from the reaction of ALC **3b** with DBCO-PEG$_5$-Val-Cit-MMAE (**5**). Method: DBCO reagent (10 equiv.), 4 h.

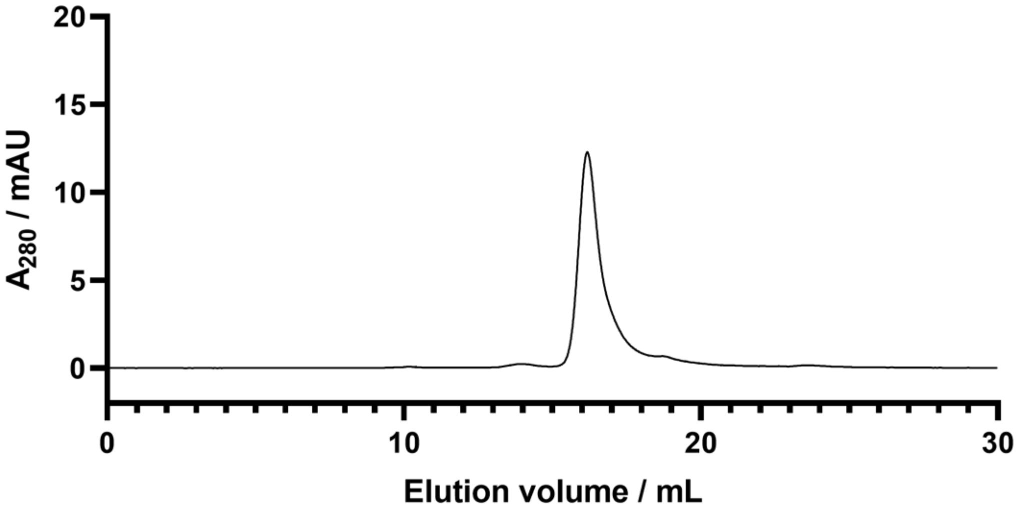

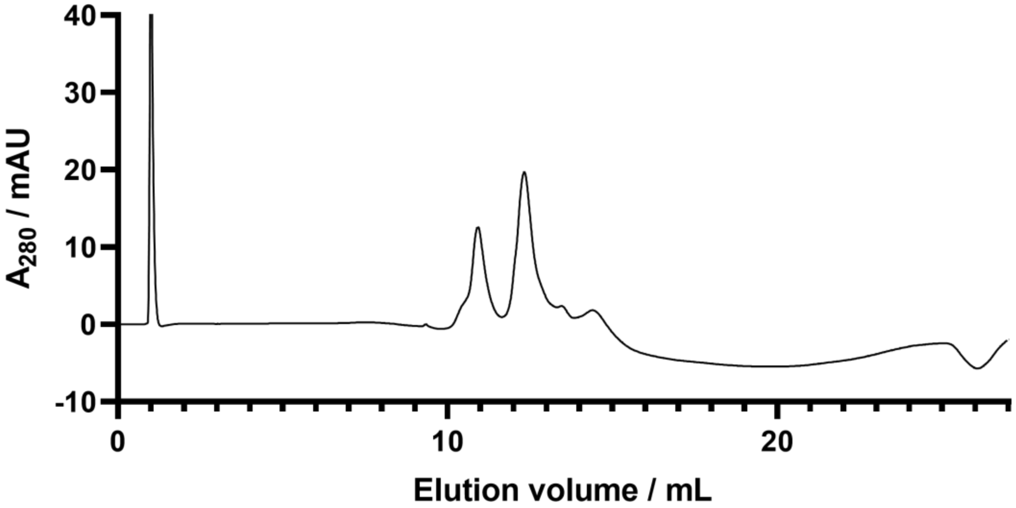

Figure S48: SEC (top) and HIC (bottom) traces of conjugate ADC **6b** from the reaction of ALC **3b** with DBCO-PEG$_5$-Val-Cit-MMAE (**5**). Method: DBCO reagent (100 equiv.), 24 h.

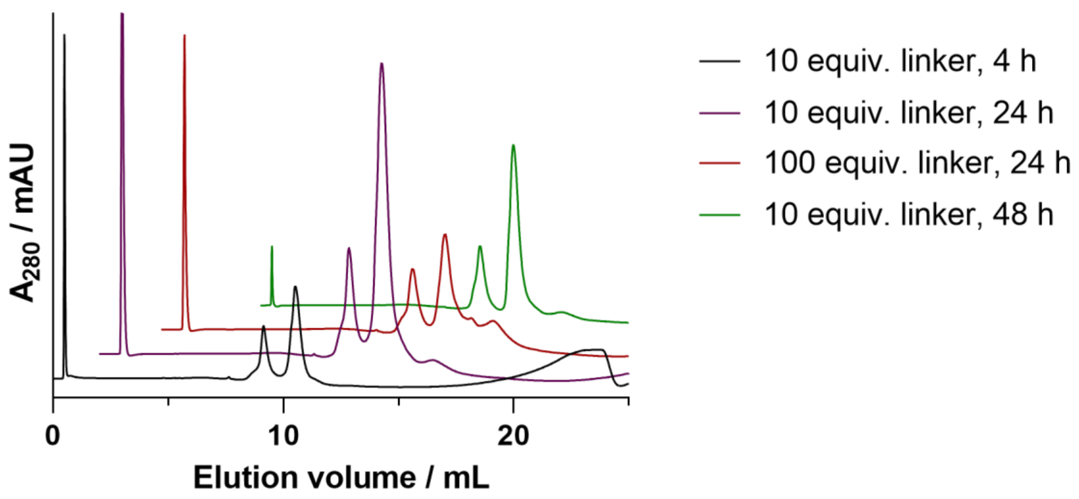

Figure S49: HIC traces of conjugate ADC **6b** from the reaction of ALC **3b** with DBCO-PEG$_5$-Val-Cit-MMAE (**5**). Method A: DBCO reagent (10 equiv.), 4 h; Method B: DBCO reagent (10 equiv.), 24 h; Method C: DBCO reagent (100 equiv.), 24 h; Method D: DBCO reagent (10 equiv.), 48 h.

ADC **6c**

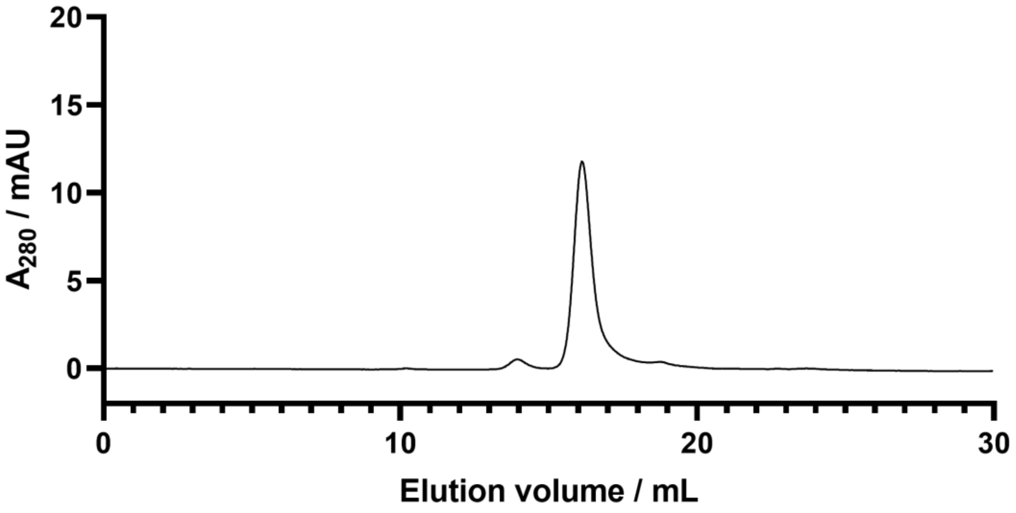

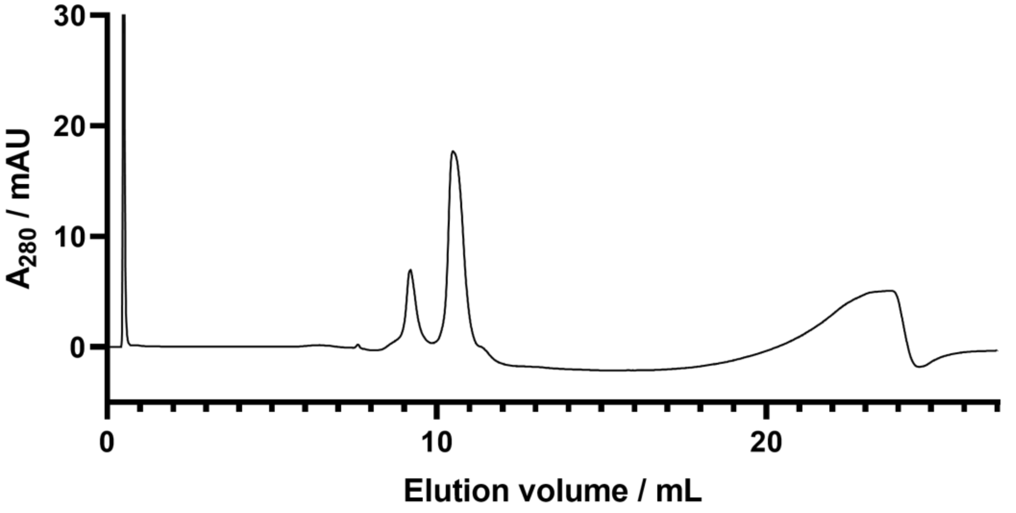

Figure S50: SEC (top) and HIC (bottom) traces of conjugate ADC **6c** from the reaction of ALC **3c** with DBCO-PEG$_5$-Val-Cit-MMAE (**5**). Method: DBCO reagent (10 equiv.), 4 h.

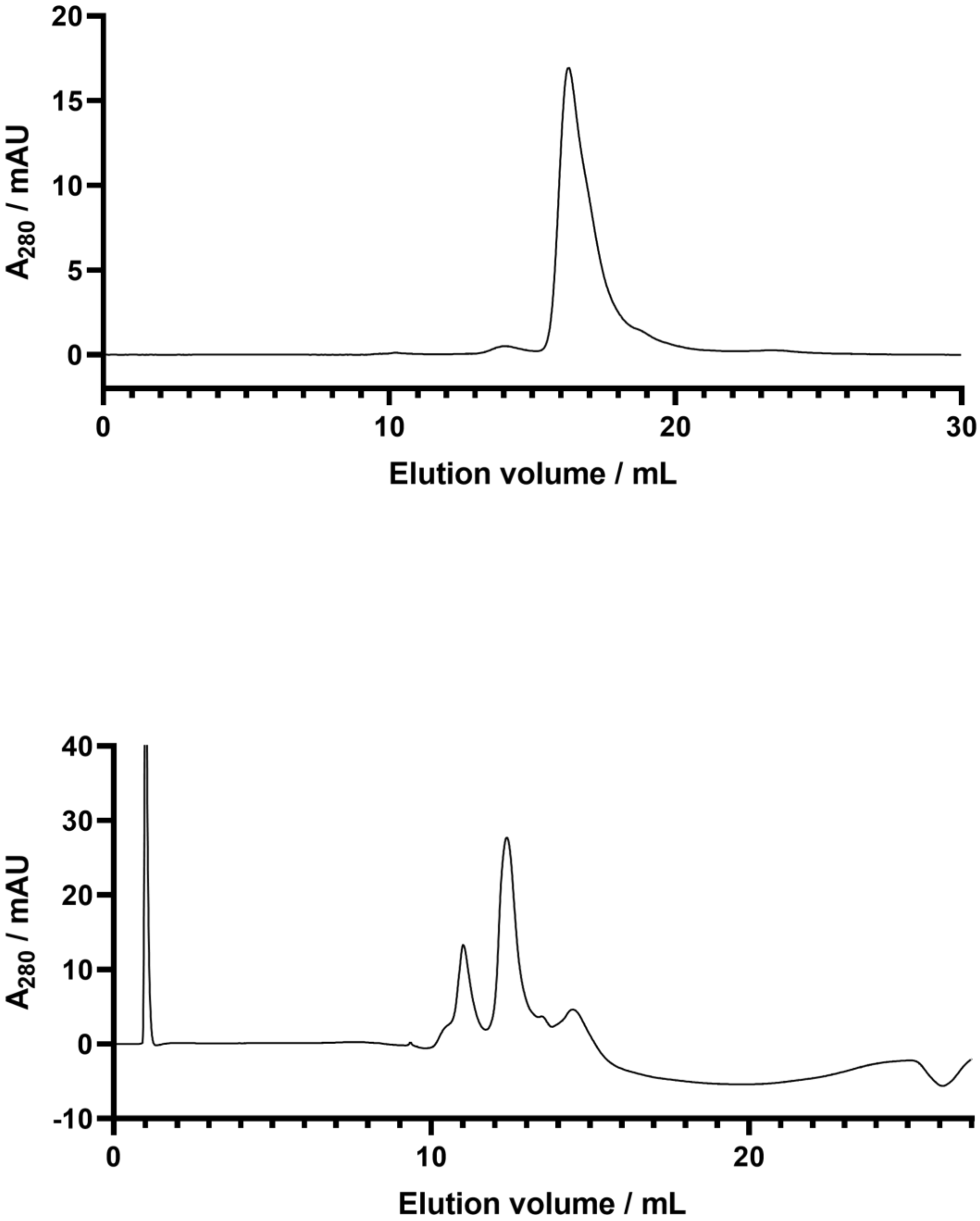

Figure S51: SEC (top) and HIC (bottom) traces of conjugate ADC **6c** from the reaction of ALC **3c** with DBCO-PEG$_5$-Val-Cit-MMAE (**5**). Method: DBCO reagent (100 equiv.), 24 h.

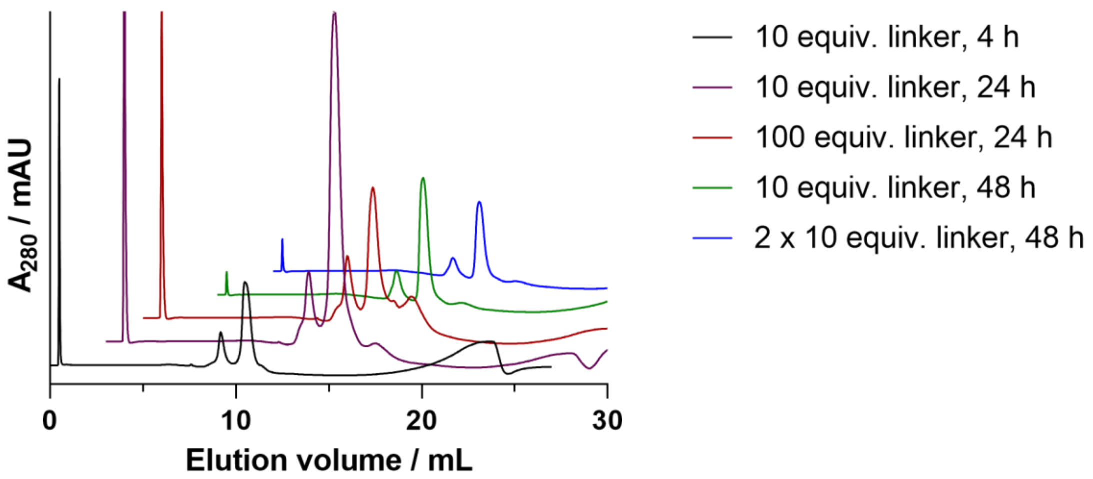

Figure S52: HIC traces of conjugate ADC **6c** from the reaction of ALC **3c** with DBCO-PEG$_5$-Val-Cit-MMAE (**5**). Method A: DBCO reagent (10 equiv.), 4 h; Method B: DBCO reagent (10 equiv.), 24 h; Method C: DBCO reagent (100 equiv.), 24 h; Method D: DBCO reagent (10 equiv.), 48 h; Method E: DBCO reagent (10 equiv.), 24 h then DBCO reagent (10 equiv.), 24 h.

ADC **6d**

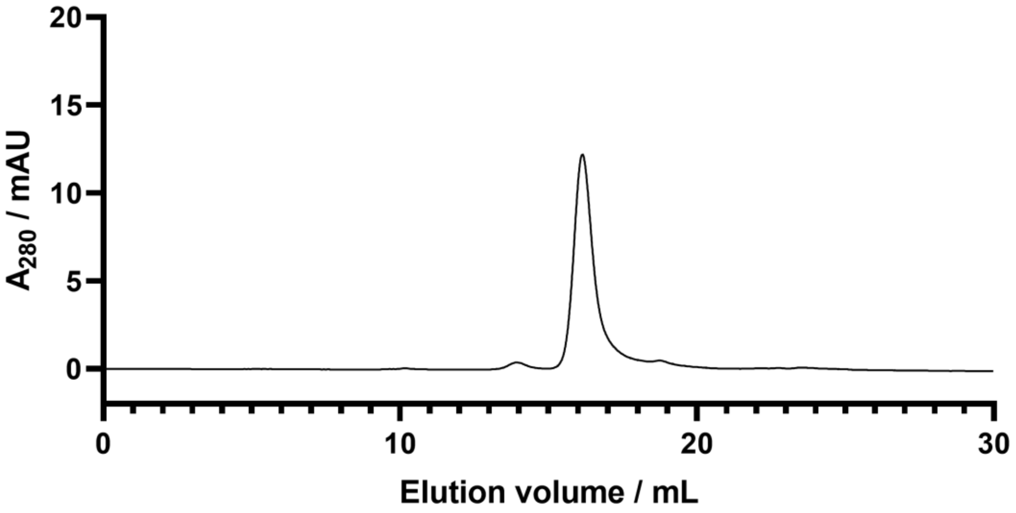

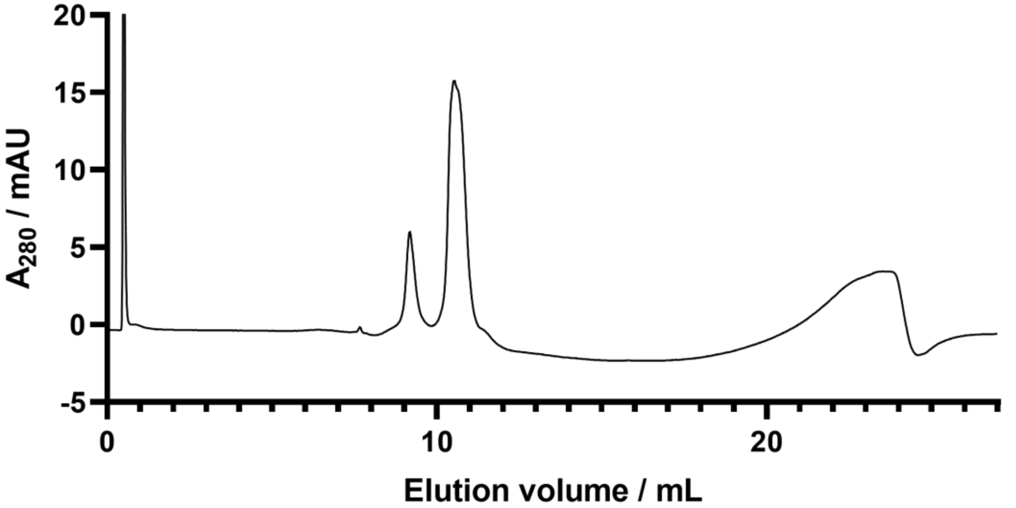

Figure S53: SEC (top) and HIC (bottom) traces of conjugate ADC **6d** from the reaction of ALC **3d** with DBCO-PEG$_5$-Val-Cit-MMAE (**5**). Method: DBCO reagent (10 equiv.), 4 h.

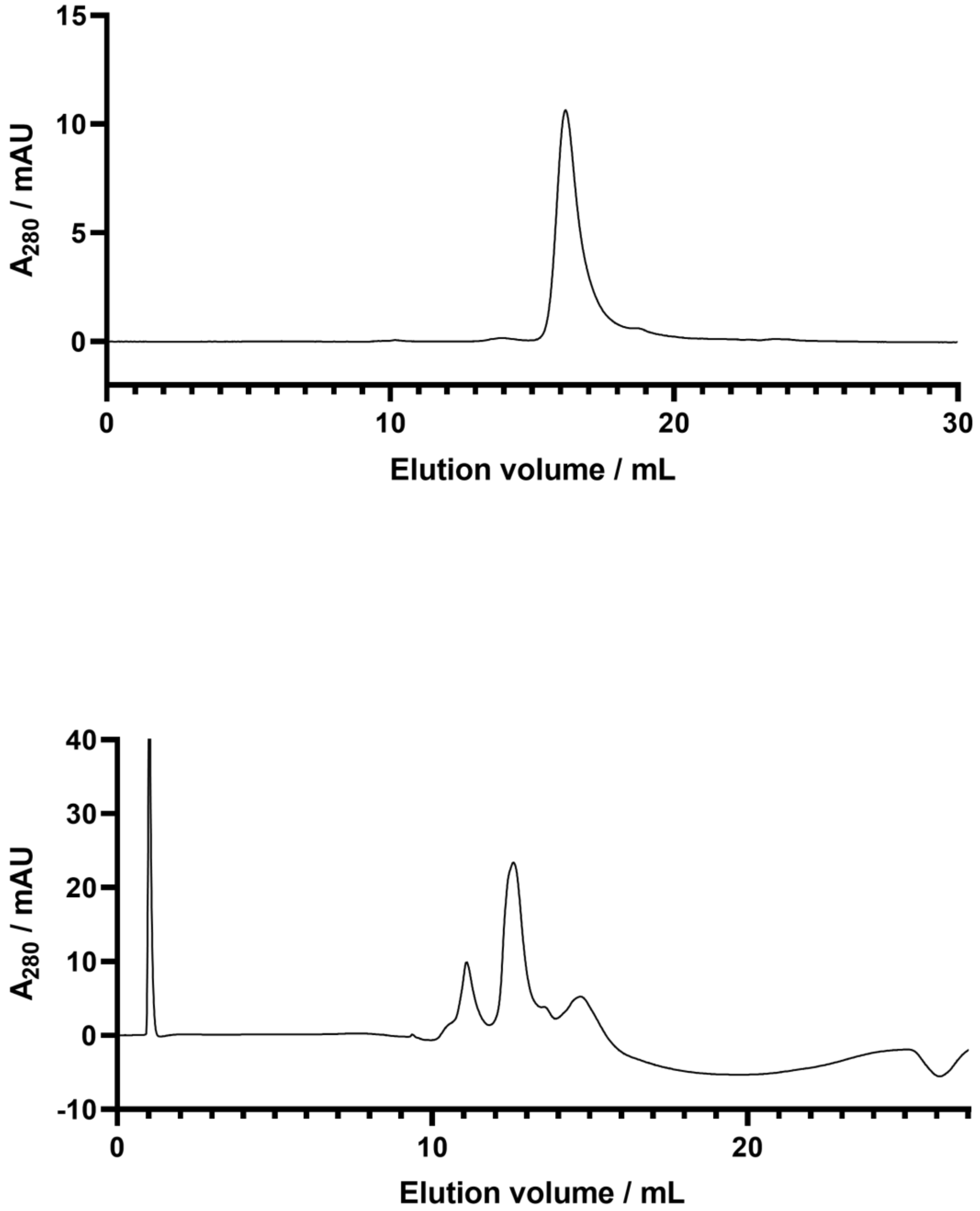

Figure S54: SEC (top) and HIC (bottom) traces of conjugate ADC **6d** from the reaction of ALC **3d** with DBCO-PEG$_5$-Val-Cit-MMAE (**5**). Method: DBCO reagent (100 equiv.), 24 h.

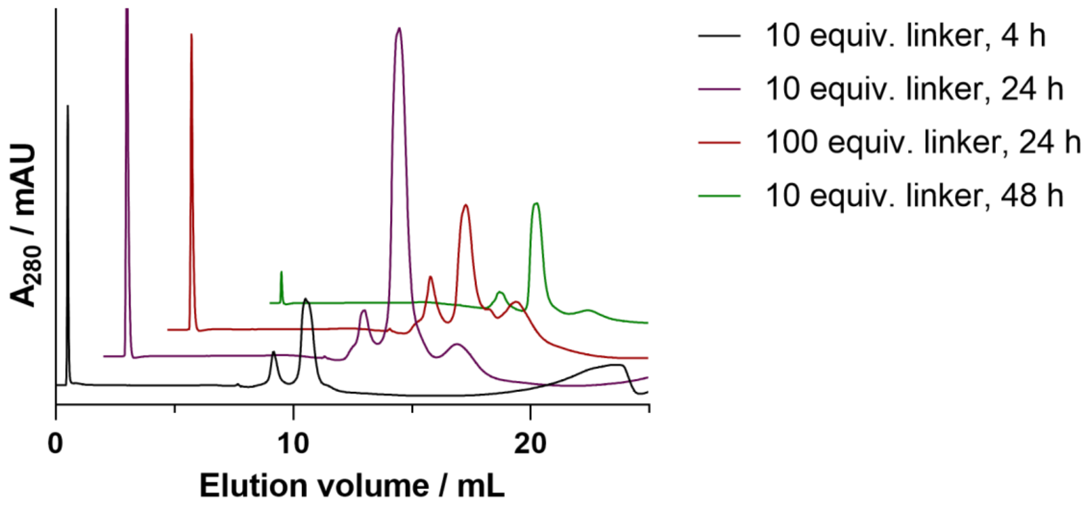

Figure S55: HIC traces of conjugate ADC **6d** from the reaction of ALC **3d** with DBCO-PEG$_5$-Val-Cit-MMAE (**5**). Method A: DBCO reagent (10 equiv.), 4 h; Method B: DBCO reagent (10 equiv.), 24 h; Method C: DBCO reagent (100 equiv.), 24 h; Method D: DBCO reagent (10 equiv.), 48 h.

ADC **7a**

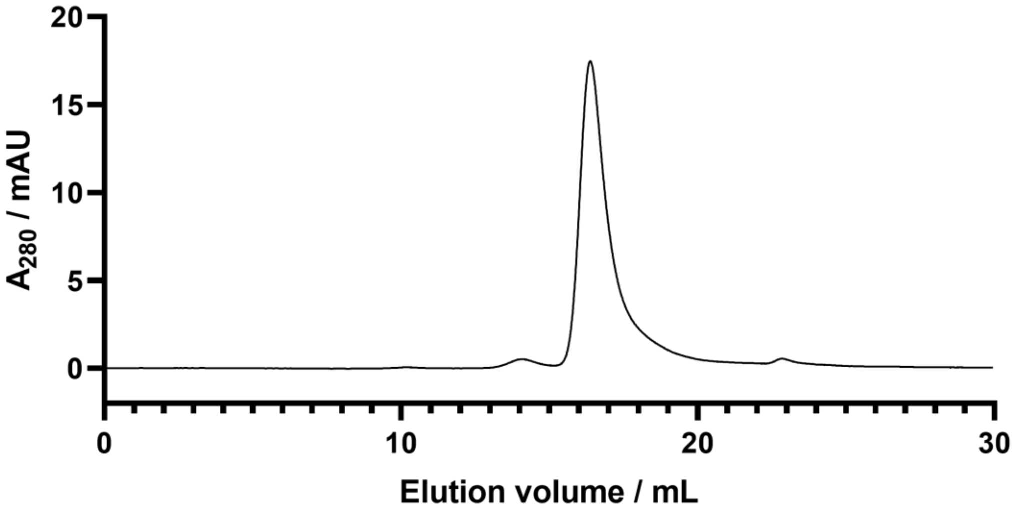

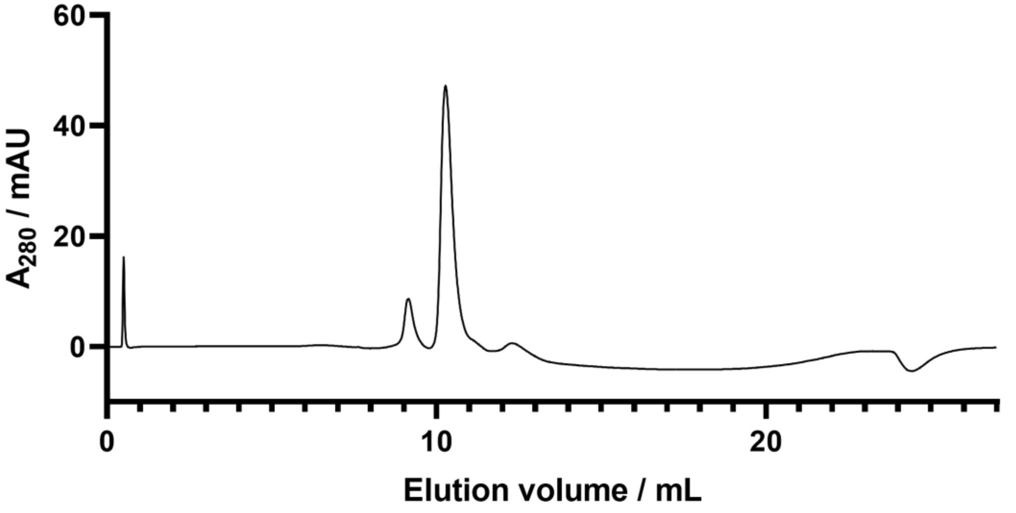

Figure S56: SEC (top) and HIC (bottom) traces of conjugate ADC **7a** from the reaction of ALC **4a** with DBCO-PEG$_5$-Val-Cit-MMAE (**5**). Method: DBCO reagent (10 equiv.), 24 h.

ADC **7b**

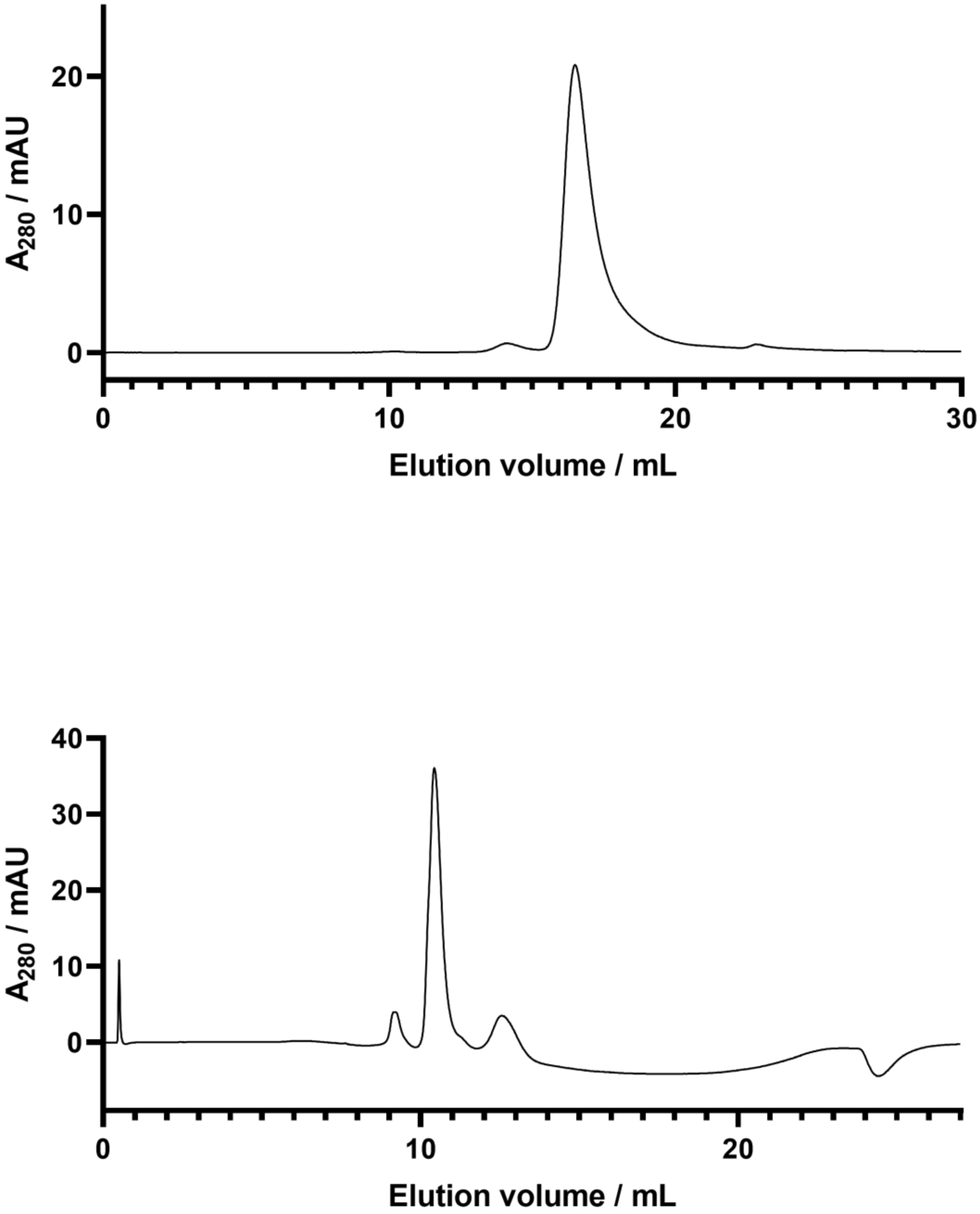

Figure S57: SEC (top) and HIC (bottom) traces of conjugate ADC **7b** from the reaction of ALC **4b** with DBCO-PEG$_5$-Val-Cit-MMAE (**5**). Method: DBCO reagent (10 equiv.), 24 h.

ADC **7c**

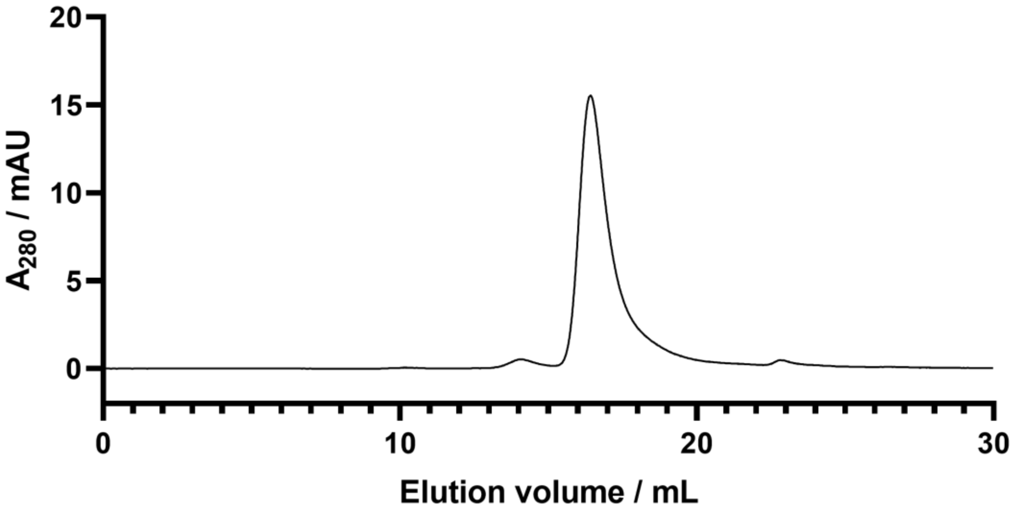

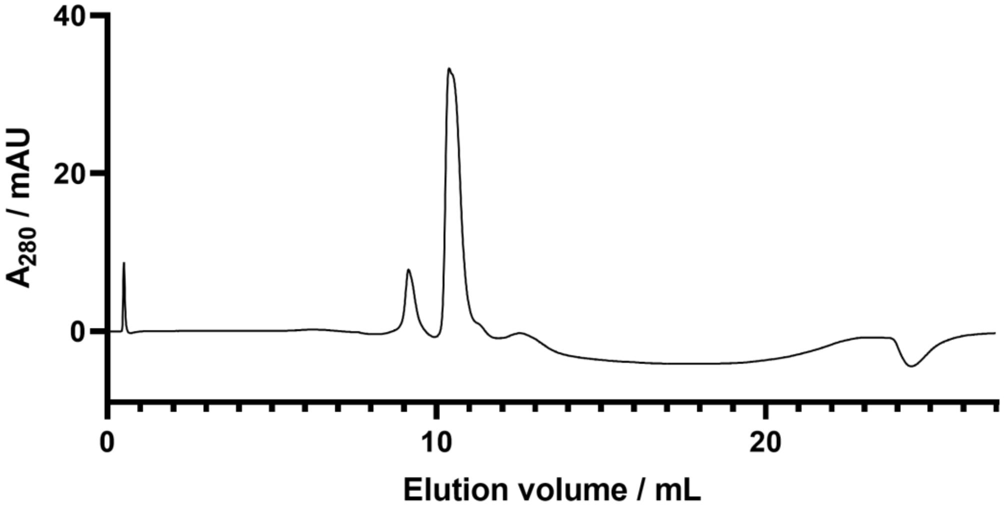

Figure S58: SEC (top) and HIC (bottom) traces of conjugate ADC **7c** from the reaction of ALC **4c** with DBCO-PEG$_5$-Val-Cit-MMAE (**5**). Method: DBCO reagent (10 equiv.), 24 h.

ADC **7d**

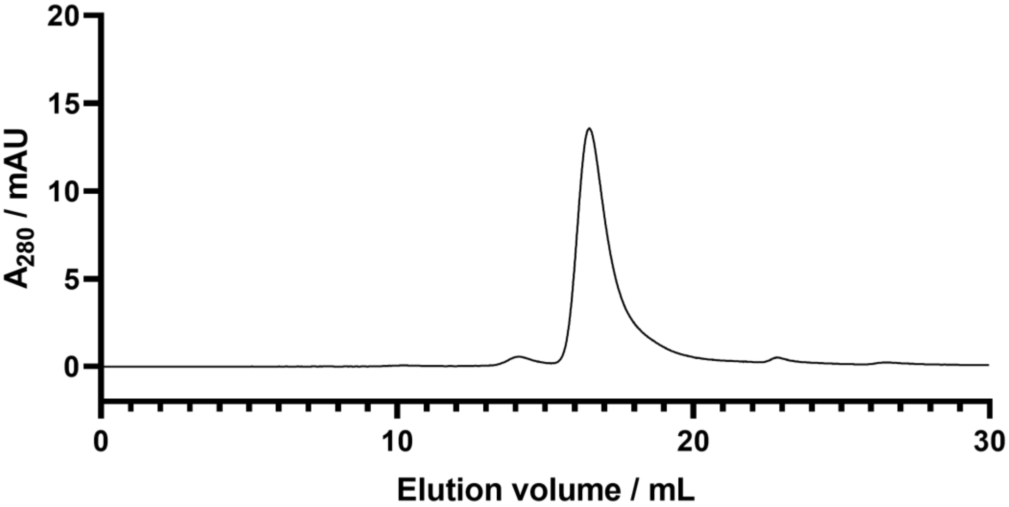

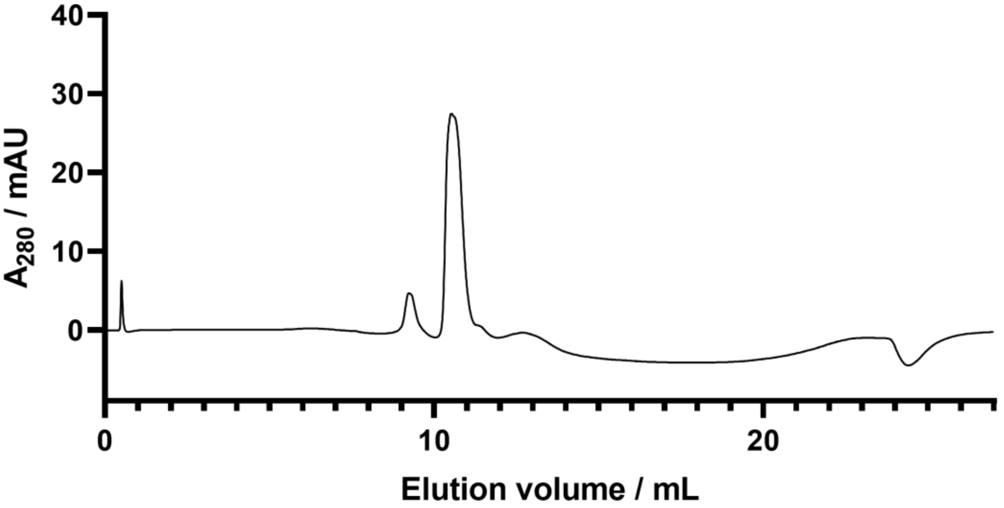

Figure S59: SEC (top) and HIC (bottom) traces of conjugate ADC **7d** from the reaction of ALC **4d** with DBCO-PEG$_5$-Val-Cit-MMAE (**5**). Method: DBCO reagent (10 equiv.), 24 h.

ADC **9a**

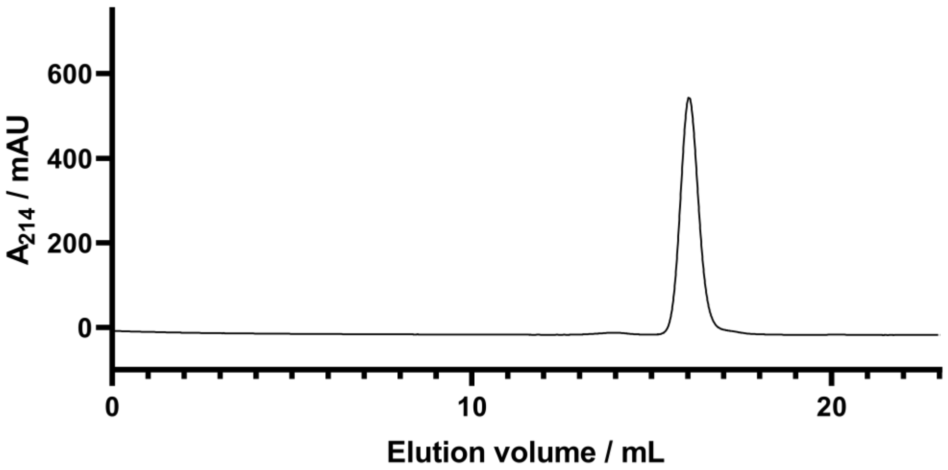

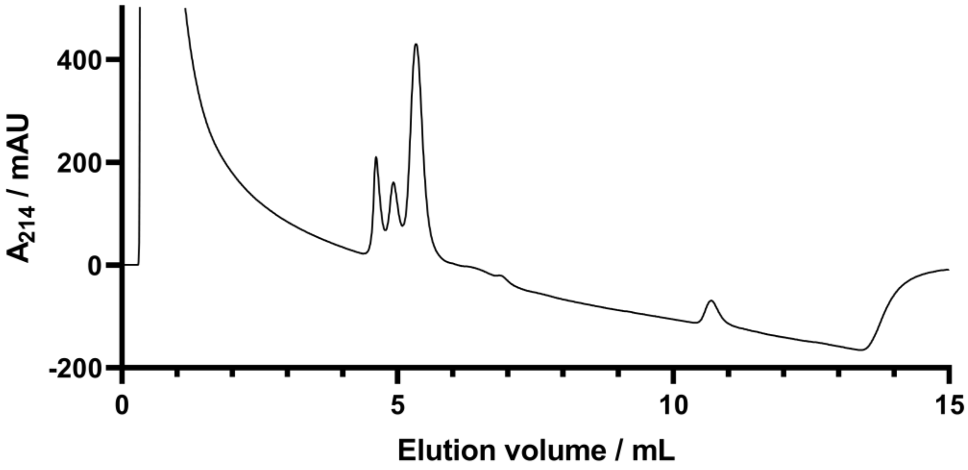

Figure S60: SEC (top) and HIC (bottom) traces of conjugate ADC **9a** from the reaction of ALC **4a** with DBCO-PEG$_8$-Val-Ala-PBD (**8**). Method: DBCO reagent (10 equiv.), 24 h.

ADC **9b**

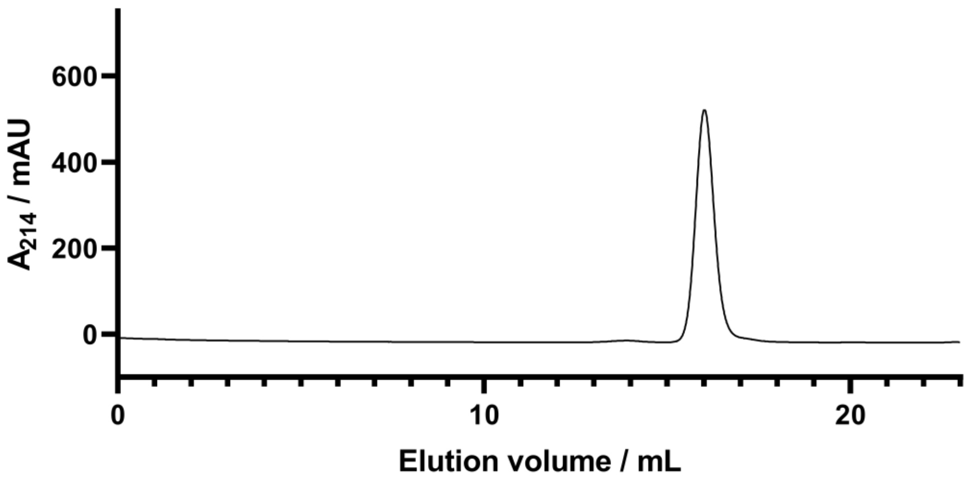

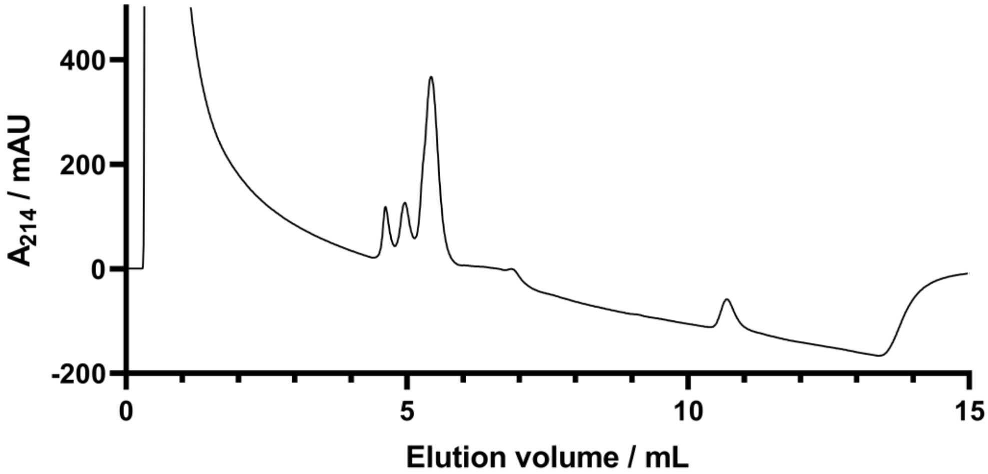

Figure S61: SEC (top) and HIC (bottom) traces of conjugate ADC **9b** from the reaction of ALC **4b** with DBCO-PEG$_8$-Val-Ala-PBD (**8**). Method: DBCO reagent (10 equiv.), 24 h.

ADC **9c**

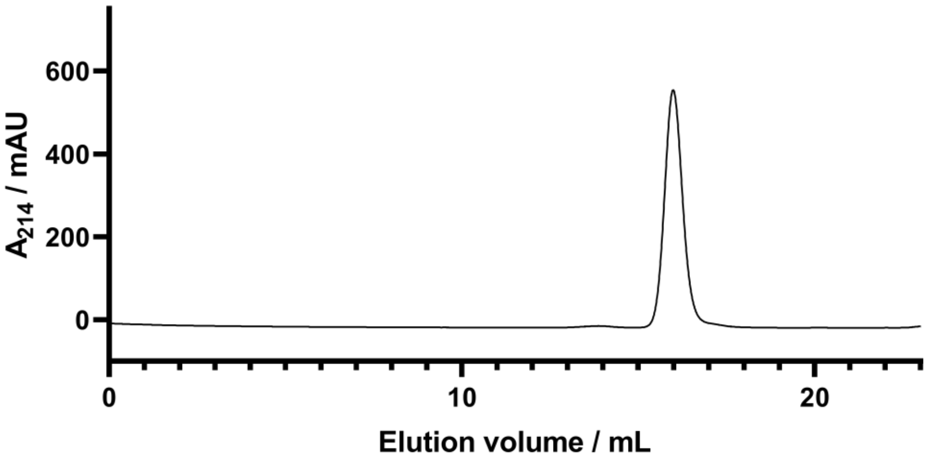

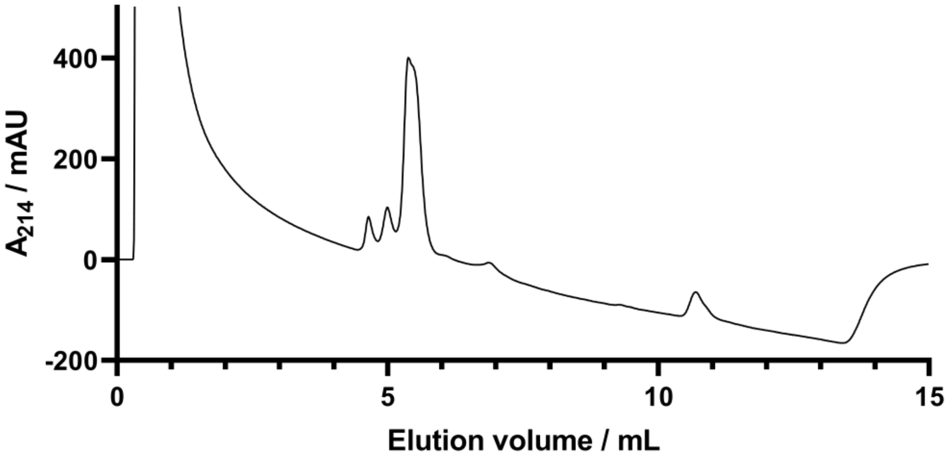

Figure S62: SEC (top) and HIC (bottom) traces of conjugate ADC **9c** from the reaction of ALC **4c** with DBCO-PEG$_8$-Val-Ala-PBD (**8**). Method: DBCO reagent (10 equiv.), 24 h.

ADC **9d**

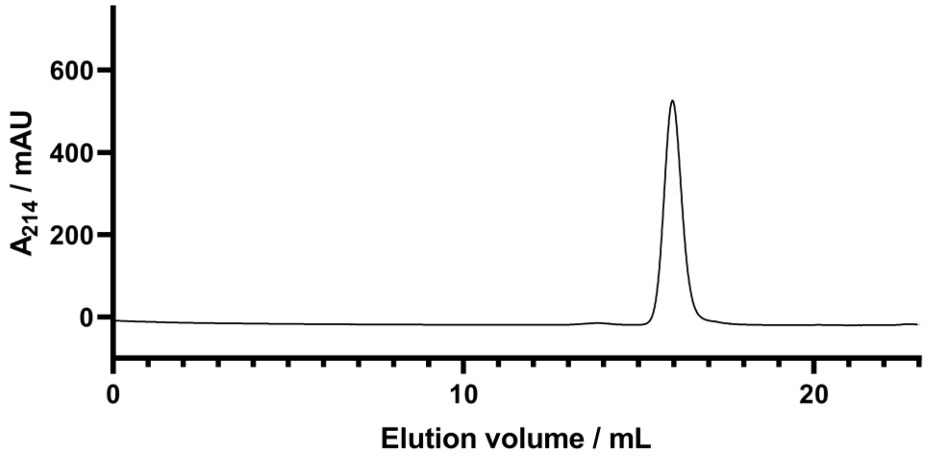

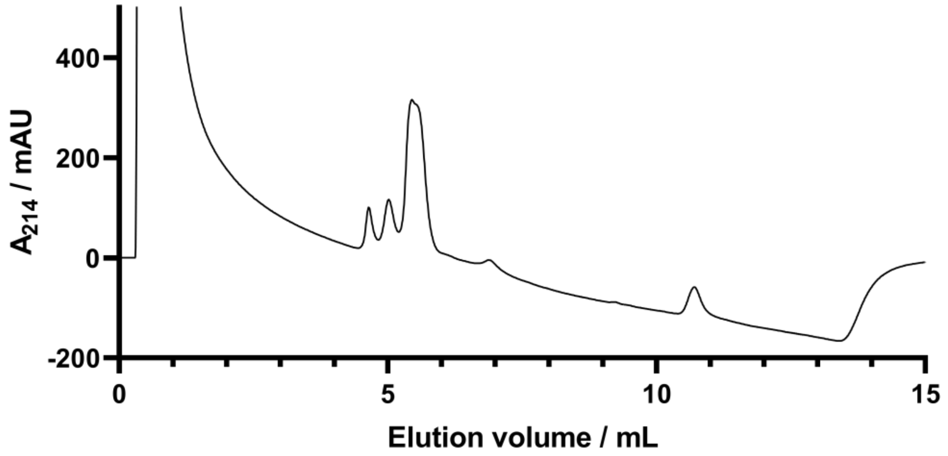

Figure S63: SEC (top) and HIC (bottom) traces of conjugate ADC **9d** from the reaction of ALC **4d** with DBCO-PEG$_8$-Val-Ala-PBD (**8**). Method: DBCO reagent (10 equiv.), 24 h.
